# Synthesis of β‐Diamine Building Blocks by Photocatalytic Hydroamination of Enecarbamates with Amines, Ammonia and N−H Heterocycles

**DOI:** 10.1002/chem.202003562

**Published:** 2020-10-07

**Authors:** Daniel Francis, Adam Nelson, Stephen P. Marsden

**Affiliations:** ^1^ School of Chemistry University of Leeds Leeds LS2 9JT UK; ^2^ Astbury Centre for Structural Molecular Biology University of Leeds Leeds LS2 9JT UK

**Keywords:** (hetero)aromatic amine, ammonia, hydroamination, medicinal chemistry toolkit, photoredox

## Abstract

3‐Amino‐substituted saturated nitrogen heterocycles are an important subclass of β‐diamines, appearing in a number of clinical agents. Herein, we report a unified approach to these products based upon the regioselective photoredox‐mediated hydroamination of enecarbamates. The amine coupling partner can encompass diverse amine types under a single set of reaction conditions, including primary alkyl amines, ammonia, aryl and heteroaryl amines, and N−H heterocycles. The method enables the synthesis of a wide range of pharmaceutically relevant building blocks.

## Graphical abstract

**Medicinally important** 3‐amino‐substituted cyclic amines can be accessed in a single step by photoredox‐catalysed hydroamination of cyclic enecarbamates. A diverse range of drug‐relevant amine substituents can be introduced under unified reaction conditions, including primary alkyl amines, ammonia, (hetero)aromatic amines, and *N*‐heterocycles (see scheme).

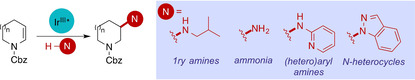

Vicinal diamines (also known as 1,2‐ or β‐diamines) are important subunits of many important organic molecules, including pharmaceutical compounds, natural products and ligands for asymmetric catalysis.^[1]^ A valuable sub‐class of β‐diamines comprises saturated nitrogen heterocycles containing a 3‐amino‐substituent. Examples of this motif in drug molecules include the fluoroquinolone antibiotic besifloxacin **1** (Figure 1);^[2]^ tofacitinib **2** (a JAK inhibitor used to treat arthritis);^[3]^ ibrutinib **3** (a BTK inhibitor used to treat B cell cancers);^[4]^ and the investigational rhinitis treatment SB‐705498 **4**.^[5]^ Significantly, as well as variation (size and substitution) in the saturated nitrogen heterocycle, the nature of the exocyclic nitrogen substituent in medicinally‐relevant structures encompasses a broad range of functionalities, including the parent amino group (e.g. **1**); alkyl and (hetero)arylamino groups (e.g. **2**); *N*‐heterocycles (e.g. **3**); and acyl/sulfonyl derivatives (e.g. **4**). This diversity of functionality presents a challenge in terms of synthesis, and the availability of a single method which facilitates access to all substituent classes from a common class of precursors would be of great value.

**Figure 1:**
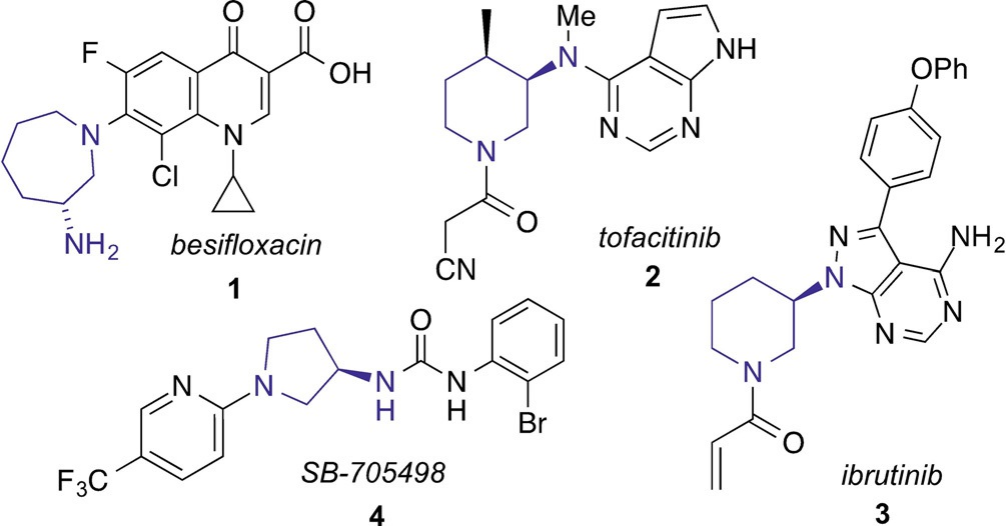
Approved and investigational drugs containing 3‐amino‐substituted nitrogen heterocycles.

Inspired by the challenges associated with the direct elaboration of hits in fragment‐based drug discovery,^[6]^ we have recently developed a method for the β‐elaboration of cyclic amines with functionalized alkyl substituents by regioselective conversion to enecarbamates and subsequent photoredox‐mediated β‐alkylation.^[7]^ We recognized that regioselective hydroamination of the same enecarbamates would offer direct access to the valuable 3‐amino‐substituted azacycles. Regioselective catalytic intermolecular hydroamination of alkenes has been a longstanding synthetic challenge.^[8]^ Studer reported pioneering radical‐based intermolecular hydroaminations of alkene substrates including enamides and enecarbamates,[^9^, ^10^] but this requires *N*‐amino Hantzsch esters^[9]^ or 3‐amino‐1,4‐ cyclohexadienes^[10]^ as the nitrogen coupling partner. More recently, a groundbreaking report by Knowles described the direct photocatalytic intermolecular hydroaminative coupling of secondary alkyl amines with a range of alkenes, including enecarbamates (Scheme 1, panel a).^[11]^

**Scheme 1:**
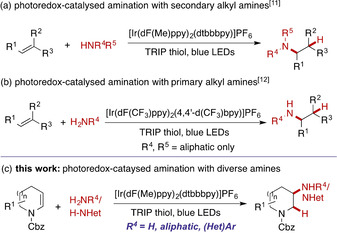
Methods for photoredox‐catalysed hydroamination.

Although it was reported that primary amines did not react under these conditions, we were motivated to investigate whether this reaction could be realised in the context of enecarbamate substrates. Subsequent to the commencement of our efforts, Knowles recently demonstrated that the use of a more oxidizing catalyst can effect the selective hydroamination of primary alkyl amines (Scheme 1, panel b).^[12]^ We have found in the interim, however, that primary alkyl amines and even ammonia can be coupled with enecarbamates using the initial photocatalyst by a mechanism likely distinct from that operating in Knowles’ reactions. This mechanistic divergence allows not only the coupling of aliphatic amines and ammonia but also that of less nucleophilic aromatic and heteroaromatic amines and even N–H azole heterocycles under unified conditions. We describe herein the results of these studies.

We began by investigating the reaction of isobutylamine as a simple primary amine with 5‐ and 6‐membered cyclic enecarbamates **5 a**/**b** (Table 1). Under conditions similar to those previously reported for secondary amines^[11]^ we were pleased to observe that **5 a** underwent an extremely efficient coupling to generate **6 a** in very good yield (entry 1). The 6‐membered substrate **5 b** was somewhat less reactive (entry 2), but while there was little effect of changing catalyst loading or concentration (entries 3–5) or using alternative solvents (Supporting Information), we found that simply employing extended reaction times gave good conversion and yield (entry 6).

**Table 1:** Optimisation of the hydroamination reaction.

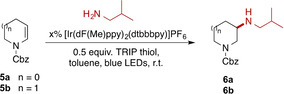						
Entry	SM	*x* % cat.	[c] [mm]	*t* [h]	Prod.	Conv./yield [%]^[a]^
1	**5 a**	2	50	16	**6 a**	>95 (79)
2	**5 b**	2	50	16	**6 b**	39 (33)
3	**5 b**	4	50	16	**6 b**	37
4	**5 b**	2	100	16	**6 b**	33
5	**5 b**	2	200	16	**6 b**	25
6	**5 b**	2	50	40	**6 b**	90 (65)

[a] Conversion measured from crude ^1^H NMR; isolated yields in parentheses.

We next turned our attention to the scope of this transformation (Scheme 2). Five‐, six‐ and seven‐membered enecarbamates could all be successfully aminated, but with a clear decrease in reactivity (**6 a**–**c**) with increasing ring size (all reactions were generally clean and unreacted enecarbamate was observed in the crude ^1^H NMR). The reaction was not limited to enecarbamates: cyclic enamides were also substrates, giving **6 d** in good yield. Ring‐substituted substrates reacted successfully but the products **6 e**–**g** were formed with low diastereoselectivity. The reaction showed excellent tolerance for steric bulk in the enecarbamate partner: the presence of an adjacent spirocyclic centre did not significantly impact the formation of **6 i**. The reaction also allows the preparation of amines bearing tertiary alkyl substituents by addition to tri‐substituted enecarbamates, with ring‐fused amine **6 j** formed as a single (*cis*‐fused) diastereomer.

**Scheme 2:**
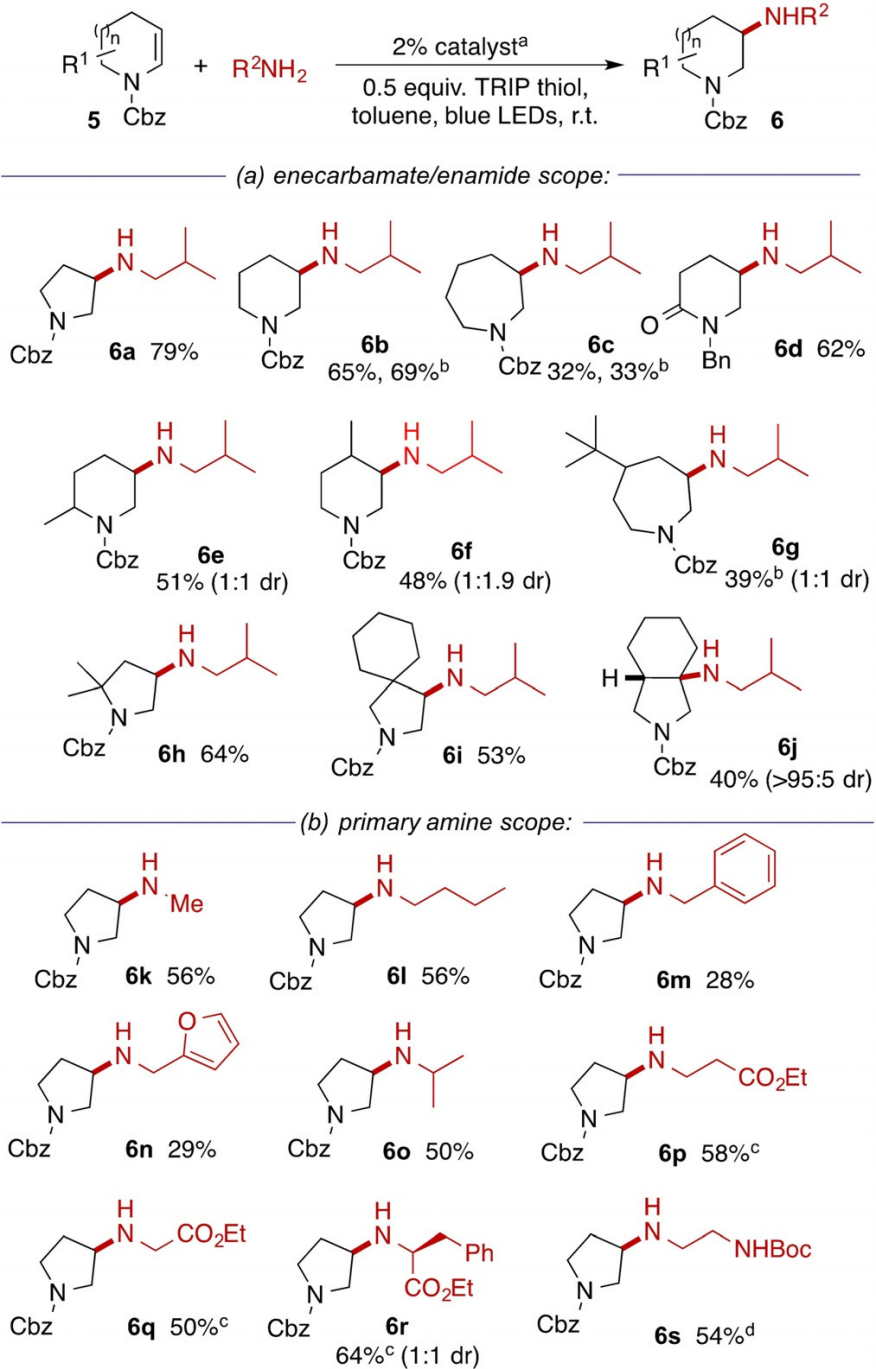
Scope of photoredox hydroamination of cyclic enecarbamates. [a] 2 % [Ir(dF(Me)ppy)_2_(dtbbpy)]PF_6_, 5 equiv amine, 0.5 equiv TRIP thiol, toluene, blue LEDs, r.t., 16 h; [b] using 2 % [Ir(df(CF_3_)ppy)_2_(dtbbpy)]PF_6_; [c] using 2 equiv amine hydrochloride salt, 2 equiv LiOH⋅H_2_O; [d] using 2 equiv amine.

In terms of the scope of the primary amine, examples bearing simple alkyl, (hetero)benzylic (**6 m**,**n**) and branched secondary alkylamines (**6 o**) worked effectively. Importantly, the introduction of functionalized amines such as α‐ and β‐amino esters (**6 p–r**) and monoprotected 1,2‐diamines (**6 s**) was possible. These more complex amines were used as their hydrochloride salts at a 2:1 molar ratio. The facility to introduce functionalized substituents is a valuable feature of the reaction, for example in terms of generating potential bioactive molecules for drug discovery programmes.

Given this success with primary amines, including low molecular weight amines such as methylamine (**6 k**), we were keen to explore whether the reaction would work with ammonia as nucleophile. This would allow access to valuable primary amines (exemplified by **1**) but presents a significant synthetic challenge. Not only is the addition of ammonia to electronically‐unactivated alkenes thermodynamically unfavourable,[^13^, ^14^] but also the initial primary amine products might themselves act as (potentially more reactive) substrates, leading to over‐substitution. For these reasons, the direct hydroamination of alkenes with ammonia is limited to reactions using heterogeneous catalysts under forcing conditions unsuited to functionalized substrates,^[14]^ and recourse has generally been made to the use of protected ammonia surrogates.^[15]^ We were therefore delighted to find that reaction of enecarbamate **5 a** with excess of ammonia in dioxane under our standard conditions returned some of the desired primary amine **7 a**, albeit accompanied by a substantial amount of the disubstitution product formed by reaction of **7 a** with further **5 a**. Optimisation of the stoichiometry (Supporting Information) revealed that selectivity for **7 a** improved with the excess of ammonia but that this was countered by a reduction in reactivity at large excesses. Under the optimum conditions, 3‐aminopyrrolidine **7 a** was formed in 72 % isolated yield using methanolic ammonia (Scheme 3, panel a). The reaction showed similar tolerance to ring‐size and substitution patterns as the reactions of primary amines. The reactions of chiral substituted enecarbamates were again poorly diastereoselective with the exception of the formation of a single diastereomer of the bicyclic tertiary alkyl amine **7 j**. While this rare example of alkene hydroamination is noteworthy in itself, we recognized an opportunity to derive further synthetic utility by directly functionalizing the primary amine in a single pot operation. Thus, following removal of the solvent, addition of a range of electrophiles in the presence of an amine base allowed access to carbamates **8**, amides **9**, ureas **10** and sulfonamides **11** (Scheme 3, panel b). While conditions have been reported for hydroamination of alkenes under photoredox conditions with amides (single intermolecular example)[^16^, ^17^] carbamates[^16^, ^17b^] and ureas[^16^, ^17b^] (all intramolecular), and sulfonamides (inter‐ and intramolecular),^[18]^ these reactions require bespoke reaction conditions for each substrate class. The approach taken here allows the use of a single hydroamination protocol and has the potential to open access to a wide variety of derivatives without the requirement for optimization of individual reactant combinations.

**Scheme 3:**
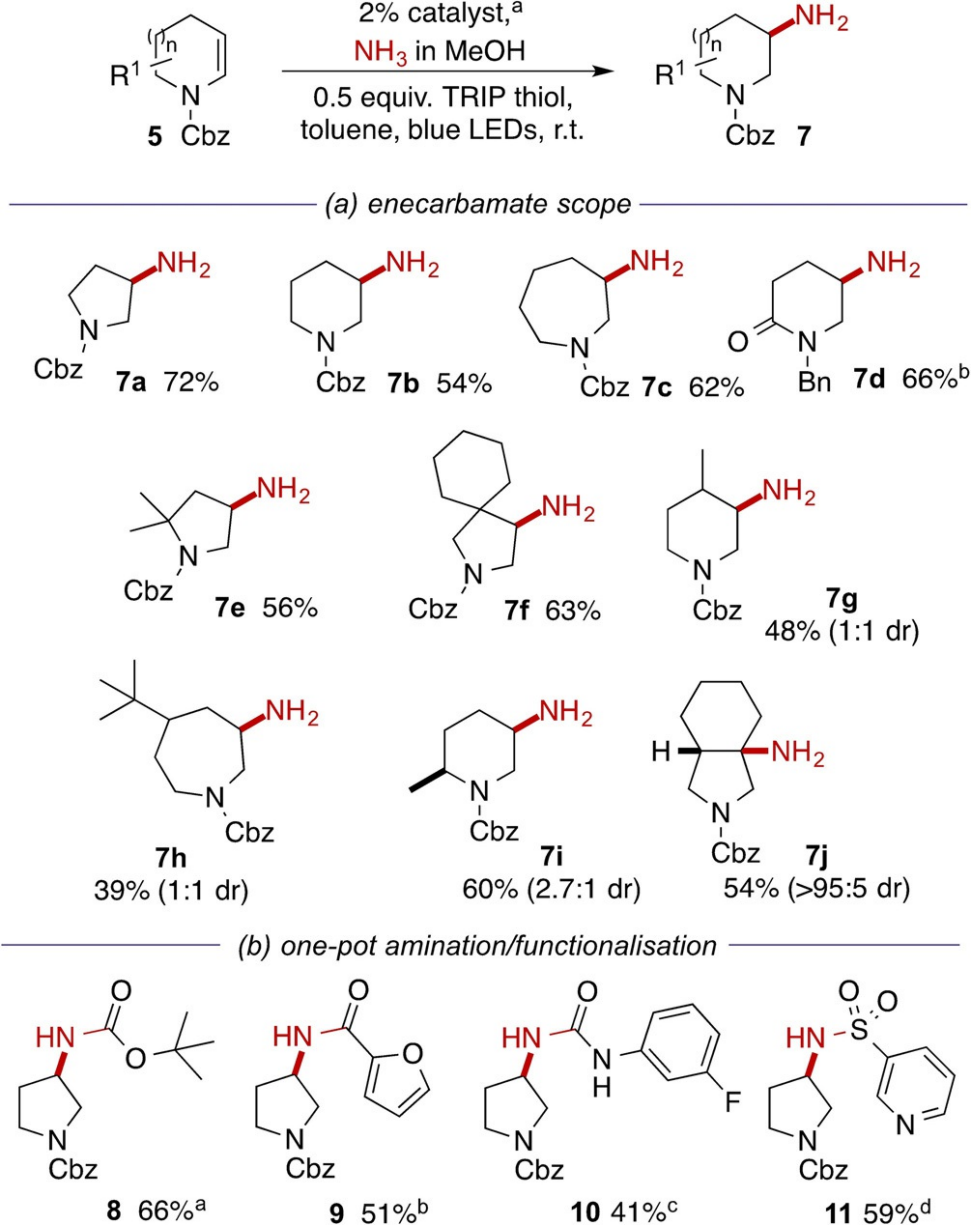
Photoredox‐catalysed hydroamination with ammonia and one‐pot transformations to functionalized derivatives. [a] 2 % [Ir(dF(Me)ppy)_2_(dtbbpy)]PF_6_, 5 equiv amine, 0.5 equiv TRIP thiol, toluene, blue LEDs, r.t., 16 h; [b] using 2 % [Ir(df(CF_3_)ppy)_2_(dtbbpy)]PF_6_; [c] after Boc_2_O, DIPEA, CH_2_Cl_2_; [d] after 2‐furoyl chloride, DIPEA, CH_2_Cl_2_; [e] after (3‐FC_6_H_4_)N=C=O, DIPEA, CH_2_Cl_2_; [f] after 3‐PySO_2_Cl, DIPEA, CH_2_Cl_2_.

We next considered the mechanism of the current transformation. The mechanism proposed by Knowles for the reactions of both secondary and primary amines involves the formation of aminium radicals **A** by reductive quenching of the excited photocatalyst; this then reacts with the alkene in an anti‐Markovnikov fashion to generate a carbon‐centred radical **B** (Scheme 4, pathway a) which abstracts a hydrogen atom from the thiol co‐catalyst.[^11^, ^12^] However, the photocatalyst used here (and in Knowles’ reactions of secondary amines) is not a strong enough oxidant to form aminium radicals from primary amines,^[12]^ and it therefore seemed unlikely that the current reactions are proceeding through this mechanism. Consistent with this, while we were able to couple secondary amines with simple aryl and alkyl‐substituted alkenes (4‐methoxystyrene and α‐pinene, respectively) under Knowles’ reaction conditions, the use of isobutylamine gave no observable reaction (Supporting Information). Stern–Volmer analysis showed that while secondary amines such as piperidine strongly quenched the photoactivated Ir^III^ species, isobutylamine was a much weaker quenching agent and ammonia did not act as a quenching agent at all. However, the enecarbamate **5 b** was found to be a moderate quenching agent, suggesting an alternative mechanism proceeding by activation of the enecarbamate (indeed Knowles suggested such a phenomenon might be occurring in hydroamination of Boc‐dihydropyrrole in reaction with secondary amines^[11]^). A plausible mechanism (Scheme 4, pathway b) could involve reductive quenching of the photoexcited Ir^III^ catalyst by **5 b** ^[19]^ to generate an electrophilic radical cation intermediate **C** which undergoes nucleophilic addition by the amine to generate the same α‐amino radical **B** as that invoked in pathway a: hydrogen atom abstraction from thiol would then deliver the observed products.^[20]^ The intermediacy of alkene‐derived radical cations has also been demonstrated in photoredox‐mediated hydroamination of alkenes by Nicewicz;[^18a^, ^18b^, ^21^] however the highly oxidizing nature of the photocatalysts in those studies meant that the reactions were limited to oxidatively resistant sulfonamide or N–H heterocycle nucleophiles.

**Scheme 4:**
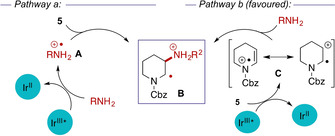
Alternative mechanistic pathways for photocatalysed hydroamination based on photocatalyst quenching by (a) amine or (b, favoured) enecarbamate.

The proposed intermediacy of the reactive radical cation **C** encouraged us to examine the behaviour of less nucleophilic aromatic and heteroaromatic amines. Intramolecular hydroamination reactions of anilines have been reported under photoredox conditions^[22]^ but, to our knowledge, there are no intermolecular examples. We were pleased to observe that under our standard conditions simple aniline derivatives coupled effectively (Scheme 5, panel a). More gratifyingly still, the introduction of the medicinally‐important 2‐aminopyridine and pyrimidine motifs was also possible (**12 c**–**g**). The poor nucleophilicity of (hetero)aryl amines means that synthesis of compounds such as tofacitinib **2** usually relies upon arylation of a pre‐existing 3‐amino‐substituted azacycle:[^3^, ^23^] the current method therefore offers a complementary and convergent approach to this motif.

**Scheme 5:**
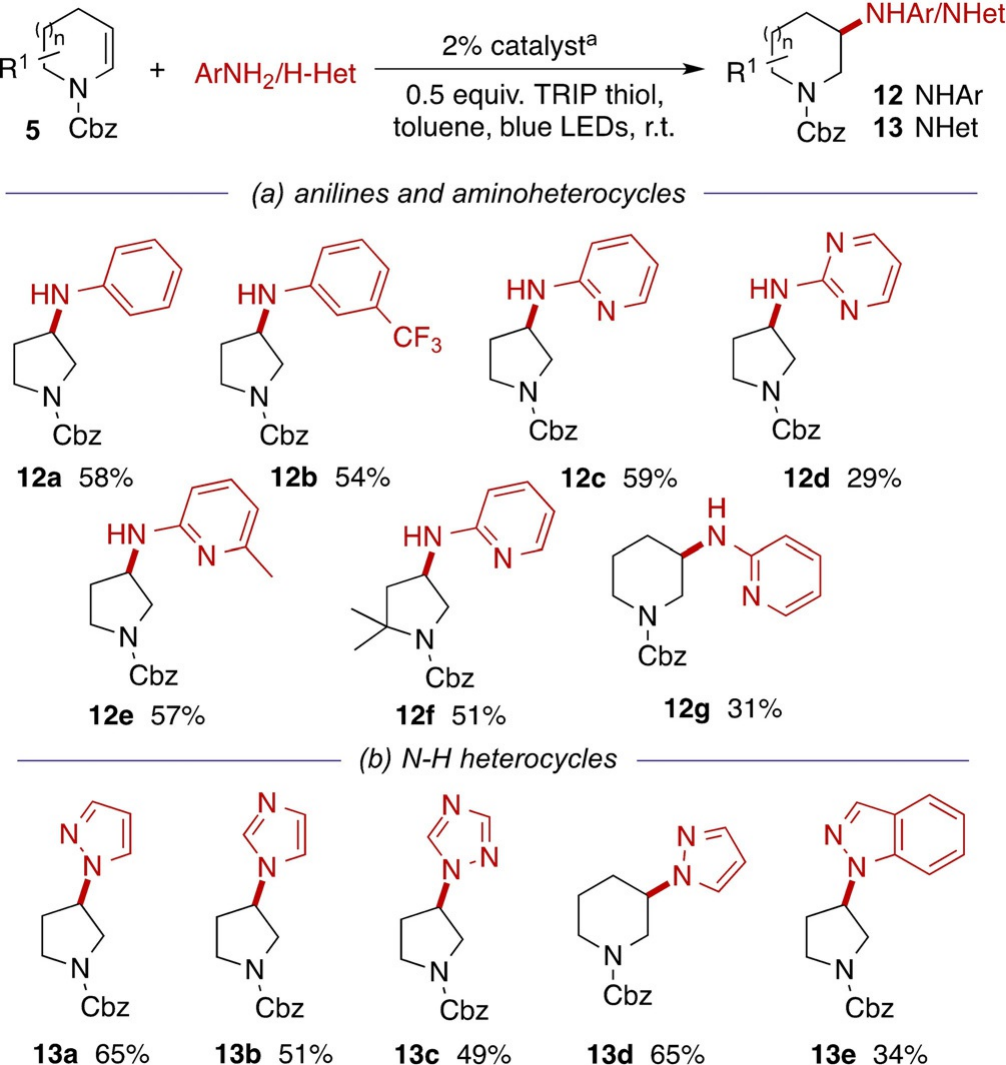
Photoredox‐catalysed hydroamination with (hetero)aromatic amines and *N*‐heterocyclic nucleophiles. [a] 2 % [Ir(dF(Me)ppy)_2_(dtbbpy)]PF_6_, 2 equiv amine, 0.5 equiv TRIP thiol, toluene, blue LEDs, r.t., 16 h.

The introduction of *N*‐heterocyclic motifs would also be extremely beneficial: current routes to compounds such as ibrutinib **3** generally rely upon Mitsunobu substitution of 3‐hydroxyazacycles^[4]^ and S_N_2 reactions of cyclic electrophiles can be highly sensitive to the local environment (e.g. steric factors). Pleasingly, under the standard conditions we were able to introduce diverse N–H heteroaromatic substituents such as pyrazole, imidazole, triazole, and indazole (Scheme 5, panel b).^[18b]^ Although these results are consistent with the proposed involvement of radical cation **C**, we cannot exclude the possibility of alternative pathways for these diverse nucleophiles.

In summary, a unified method for the synthesis of diverse 3‐amino‐substituted saturated nitrogen heterocycles has been reported. The process is mechanistically distinct from prior aminium radical‐based hydroamination reactions and supports the introduction of diverse amines, including ammonia, (hetero)aryl amines and N–H heterocycles under a single reaction regime. Given the biological relevance of diverse 3‐aminoheterocycles, this unified method to access these chemotypes should find ready application in the discovery of novel drugs and useful chemical probes, and contribute to expansion of the toolkit of reactions for medicinal chemistry.^[24]^

## Conflict of interest

The authors declare no conflict of interest.

## Supporting information

As a service to our authors and readers, this journal provides supporting information supplied by the authors. Such materials are peer reviewed and may be re‐organized for online delivery, but are not copy‐edited or typeset. Technical support issues arising from supporting information (other than missing files) should be addressed to the authors.

Supplementary
