# Supplementary material for: Synthesis of β‐Diamine Building Blocks by Photocatalytic Hydroamination of Enecarbamates with Amines, Ammonia and N−H Heterocycles
Source: Chemistry. 2020 Oct 7;26(65):14861–5. doi: 10.1002/chem.202003562 (PMC7756410; doi:10.1002/chem.202003562)

Chemistry–A European Journal

Supporting Information

Synthesis of β-Diamine Building Blocks by Photocatalytic Hydroamination of Enecarbamates with Amines, Ammonia and N–H Heterocycles

Daniel Francis,[a] Adam Nelson,*[a, b] and Stephen P. Marsden*[a]

SUPPORTING INFORMATION

Contents

General experimental 2

General methods 3

Reaction optimisation 5

Synthesis of enecarbamates 7

Aminations with primary amines (scheme 2) 12

Aminations with ammonia (and decoration) (Scheme 3) 25

Aminations with (hetero)aryl amines and N-H heterocycles (Scheme 5) 35

Control reactions 43

Stern Volmer fluorescence quenching experiments 44

Compound Spectra 45

General experimental

Commercially available starting materials were obtained from Sigma−Aldrich, Fluorochem, Acros and Alfa Aesar. All non-aqueous reactions were performed under nitrogen atmosphere unless otherwise stated. Water-sensitive reactions were performed in anhydrous solvents in oven-dried glassware cooled under nitrogen before use. Anhydrous dichloromethane (DCM), anhydrous toluene were obtained from a PureSolv MD5 Purification System. All other solvents used were of chromatography or analytical grade.

Thin layer chromatography (TLC) was performed using aluminium backed silica (Merck silica gel 60 F254) plates obtained from Merck. An ultraviolet lamp (λmax = 254 nm) and $KMnO_4$ stain were used for visualization. Flash column chromatography was performed using silica gel 60 (35-70 µm particles) supplied by Merck. A Bruker Daltonics micrOTOF spectrometer with electrospray (ES) ionisation source was used for high-resolution mass spectrometry (HRMS). Proton (^{1}H) and carbon (^{13}C) NMR data was collected on a Bruker 300, 400 or 500 MHz spectrometer using $CDCl_3$ or d_4-Methanol as solvents. Data was collected at 300 K unless otherwise stated. Chemical shifts (δ) are given in parts per million (ppm) and they are referenced to the residual solvent peak. Coupling constants (*J*) are reported in Hertz (Hz) and splitting patterns are reported in an abbreviated manner: app. (apparent), s (singlet), d (doublet), t (triplet), q (quartet), m (multiplet), br. (broad). Assignments were made using COSY, DEPT, HMQC and NOESY experiments.

General methods

General method A: Hydroamination with primary amines

To an 8mL vial equipped with a septum and stirrer bar was added enecarbamate (0.25 mmol; 1 eq.), 2,4,6-tri*iso*propylthiophenol (0.125 mmol; 50 mol%) and iridium photocatalyst A or B (0.005 mmol; 2 mol%). The vial was purged with nitrogen for 5 minutes followed by the addition of anhydrous toluene (5 mL) and primary amine (1.25 mmol; 5 eq.). The reaction was irradiated with a 32W blue LED for 16 hours. The solvent was removed *in vacuo* and the compound was purified by column chromatography or strong cation exchange chromatography.

General method B: Hydroamination with amine hydrochloride salts

To an 8 mL reaction vial equipped with a septum and stirrer bar was added enecarbamate (0.25 mmol; 1eq.), 2,4,6-tri*iso*propylthiophenol (0.125 mmol; 50 mol%), iridium photocatalyst A or B (0.005 mmol; 2 mol%), amine hydrochloride salt (0.5mmol; 2 eq.) and lithium hydroxide monohydrate (0.50 mmol; 2eq.). The vial was purged with nitrogen for 5 minutes followed by the addition of anhydrous toluene (5 mL). The reaction was irradiated with a 32W blue LED for 16 hours. The solvent was removed *in vacuo* and the compound was purified by column chromatography.

General method C: Hydroamination with heteroaromatic and aromatic amines

To an 8 mL reaction vial equipped with a septum and stirrer bar was added enecarbamate (0.25 mmol; 1eq.), 2,4,6-tri*iso*propylthiophenol (0.125 mmol; 50 mol%), iridium photocatalyst A or B (0.005 mmol; 2 mol%) and (hetero)aromatic amine (0.50 mmol; 2eq.). The vial was purged with nitrogen for 5 minutes followed by the addition of anhydrous toluene (5 mL). The reaction was irradiated with a 32W blue LED for 16 or 40 hours. The solvent was removed *in vacuo* and the compound was purified by column chromatography.

General method D: Hydroamination with ammonia

To an 8mL reaction vial equipped with a septum and stirrer bar was added enecarbamate (0.25 mmol; 1eq.), 2,4,6-tri*iso*propylthiophenol (0.125 mmol; 50 mol%) and iridium photocatalyst A or B (0.005 mmol; 2 mol%). The vial was purged with nitrogen for 5 minutes followed by the addition of anhydrous toluene (4 mL) and ammonia (7M in methanol, 1 mL). The reaction was irradiated with a 32W blue LED for 16 hours. The solvent was removed *in vacuo* and the compound was purified by column chromatography or strong cation exchange chromatography.

General method E: Hydroamination with ammonia: telescoped reactions

To an 8 mL reaction vial equipped with a septum and stirrer bar was added enecarbamate (0.25 mmol; 1eq.), 2,4,6-tri*iso*propylthiophenol (0.125 mmol; 50 mol%) and iridium photocatalyst A or B (0.005 mmol; 2 mol%). The vial was purged with nitrogen for 5 minutes followed by the addition of anhydrous toluene (4 mL) and ammonia (1 mL 7M in methanol). The reaction was irradiated with a 32W blue LED for 16 hours. The solvent was removed *in vacuo* followed by the addition of electrophile (0.5 mmol; 2eq.), DIPEA (0.5 mmol, 2eq.) and DCM (2 mL). The mixture was stirred at room temperate for 4-16 hours. The solvent was removed *in vacuo* followed by purification by column chromatography.

Reaction optimisation

A: Primary amines

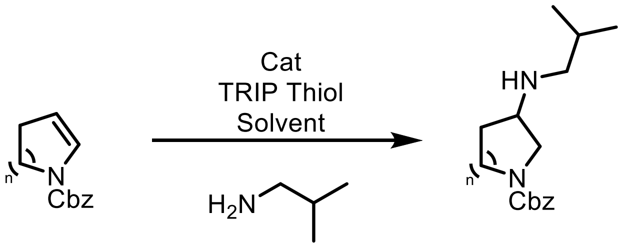

Reactions were set up as for general method A, with deviations from the general method listed in the table below. Conversion was measured by consumption of starting enecarbamate. Isolated yields were recorded after compound purification.

n	Solvent	Loading	[C]	Catalyst	Time (h)	Conversion (yield%)
1	Toluene	2%	50 mM	A	16h	>95% (79%)
2	Toluene	2%	50 mM	A	16h	39% (33%)
2	Toluene	4%	50 mM	A	16h	37%
2	CF_3Tol	2%	50 mM	A	16h	21%
2	DMSO	2%	50 mM	A	16h	15%
2	Methanol	2%	50 mM	A	16h	7%
2	Toluene	2%	100 mM	A	16h	33%
2	Toluene	2%	200 mM	A	16h	25%
2	Toluene	2%	50 mM	A	65h	90% (65%)
2	Toluene	2%	50 mM	B	16h	>95% (69%)

Table 1: . Optimisation of the hydroamination reaction with primary using *iso*butylamine. Conversions were measured from crude ^{1}H NMR. Catalyst A = $[Ir(dF(Me)ppy)_2(dtbbpy)]PF_6$, Catalyst B = $[Ir(df(CF_3)ppy)_2(dtbbpy)]PF_6$

B: Ammonia

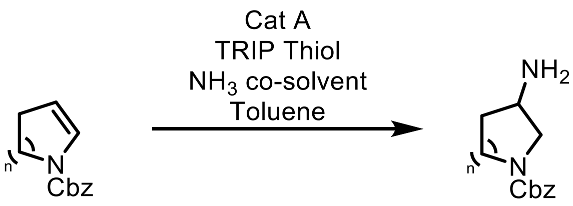

Reactions were set up as for general method D, with deviations from the general method listed in the table below. Conversion was measured by consumption of starting enecarbamate. Isolated yields were recorded after compound purification.

Ammonia equivalence	Co-Solvent	Conversion	Ratio Mono: di alkylated	Yield
4	Dioxane	>10%*	1:2	-
8	Dioxane	-	1:2	33%*
10	Methanol	68%*	2:1	54%*
140	Methanol	0%	-	-
70	Methanol	0%	-	-
35	Methanol	83%	>10:1	72%

Table 1: Optimisation of the hydroamination reaction with ammonia. Conversions were measured from crude ^{1}H NMR. *denotes over alkylation observed by ^{1}H NMR. Ratio of mono:dialkylated amine was qualitatively measured based on uncorrected LC-MS integrations. Catalyst A = $[Ir(dF(Me)ppy)_2(dtbbpy)]PF_6$

Synthesis of enecarbamates

Benzyl 2,3,4,5-tetrahydro-1*H*-azepine-1-carboxylate

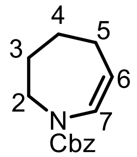

Benzyl 2-oxoazepane-1-carboxylate (2.00 g, 8.00 mmol) was dissolved in anhydrous toluene (15 mL) and cooled to -78 ºC. A solution of Superhydride (1M in THF, 9.6 mL, 9.6 mmol). The mixture was allowed to stir at -78ºC for 45 minutes followed by the sequential dropwise addition of DIPEA (6.9 mL 40.0 mmol), trifluoroacetic anhydride (1.35 mL 9.60 mmol) and DMAP (10mg). The mixture was stirred, allowing to warm from -78ºC to room temperate, for 16 hours. The reaction was diluted with water (50 mL) and ethyl acetate (50 mL), the layers separated and the aqueous further extracted with ethyl acetate (3 x 50 mL). The combined organic layers were dried over $MgSO_4$, evaporated *in vacuo* and the residue purified by column chromatography (20% ethyl acetate in hexane) resulting in a colourless oil (1.568g, 78%) as a mixture of rotamers.

^{1}H NMR (501 MHz, Chloroform-*d*) δ_H 7.33–7.21 (5H, m, Cbz), 6.57–6.38 (1H, m, H7), 5.09 (2H, s, $CbzCH_2$), 5.05–4.92 (1H, m, H6), 3.64 (2H, dd, *J* 6.8, 5.0, H2), 2.16–2.05 (2H, m, H5), 1.76–1.67 (2H, m, H3/H4), 1.67–1.58 (2H, m, H3/H4); ^{13}C NMR (126 MHz, $CDCl_3$) δ 154.4 (C=O), 130.6 (ArC), 129.9 (C7), 128.5 (ArCH), 128.1 (ArCH), 127.9 (ArCH), 116.0 (C6), 115.8 (C6), 67.5 ($CbzCH_2$), 47.7 (C2), 28.1 (C5), 26.3 (C3/4), 26.0 (C3/4), 25.1 (C3/4); 14 signals observed; HRMS: $C_{14}H_{17}NNaO_2$ $[M + Na]^+$ requires 254.1157, found 254.1156.

1-Benzyl-3,4-dihydropyridin-2(1*H*)-one

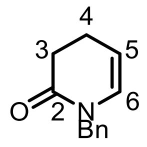

1-Benzylpiperidine-2,6-dione (1.33 g, 6.55 mmol) was dissolved in anhydrous toluene (10 mL) and cooled to -78ºC. A solution of Superhydride (1M in THF, 7.2 mL, 7.2 mmol) was added dropwise. The mixture was allowed to stir at -78ºC for 45 minutes followed by the sequential dropwise addition of DIPEA (6.5 mL 37.0 mmol), trifluoroacetic anhydride (1.0 mL 2.9 mmol) and DMAP (10mg). The mixture was stirred, allowing to warm from -78ºC to room temperate, for 16 hours. The reaction was diluted with water (50 mL) and ethyl acetate (50 mL), the layers separated and the aqueous further extracted with ethyl acetate (3 x 50 mL).

The combined organic layers were dried over $MgSO_4$, evaporated *in vacuo* and the residue purified by column chromatography (15% ethyl acetate in hexane) resulting in a colourless oil (749mg, 67%).

^1H NMR (501 MHz, Chloroform-*d*) δ $_H$ 7.36–7.13 (5H, m, Bn), 6.03–5.98 (1H, m, H6), 5.15–5.09 (1H, m, H5), 4.67 (2H, s, CH_2Bn), 2.34 (2H, t, *J* 8.1, H3), 2.15–2.05 (2H, m, H4); ^{13}C NMR (126 MHz, $CDCl_3$) δ 169.4 (C2), 137.3 (ArC), 129.5 (ArCH), 128.7 (ArCH), 127.6 (ArCH), 127.5 (C6), 106.5 (C5), 48.8 ($BnCH_2$), 31.4 (C3), 20.4 (C4); HRMS $[M + Na]^+$ $C_{12}H_{13}NNaO$ requires 210.0895, found 210.0891.

Benzyl 4-(*tert*-butyl)-2,3,4,5-tetrahydro-1*H*-azepine-1-carboxylate

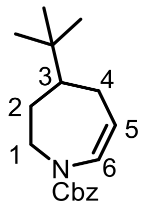

Benzyl 5-(*tert*-butyl)-2-oxoazepane-1-carboxylate(700 mg, 2.30 mmol) was dissolved in anhydrous toluene (10 mL) and cooled to -78ºC. A solution of Superhydride (1M in THF, 2.9 mL, 2.9 mmol) was added dropwise. The mixture was allowed to stir at -78ºC for 45 minutes followed by the sequential dropwise addition of DIPEA (2.9 mL 16.8 mmol), trifluoroacetic anhydride (410µl, 2.90 mmol) and DMAP (10mg). The mixture was stirred, allowing to warm from -78ºC to room temperate, for 16 hours. The reaction was diluted with water (50 mL) and ethyl acetate (50 mL), the layers separated and the aqueous further extracted with ethyl acetate (3 x 50 mL). The combined organic layers were dried over $MgSO_4$, evaporated *in vacuo* and the residue purified by column chromatography (15% ethyl acetate in hexane) resulting in a colourless oil (347mg, 53%) as a mixture of rotamers.

^1H NMR (501 MHz, Chloroform-*d*) δ $_H$ 7.44–7.29 (5H, m, Cbz), 6.69–6.48 (1H, m, H6), 5.27–5.12 (2H, m, $CbzCH_2$), 5.09–4.92 (1H, m, H5), 4.08–3.87 (1H, m, H1), 3.69–3.42 (1H, m, H1'), 2.20–1.92 (3H, m, H2+H2'+H4), 1.74–1.57 (1H, m, H3), 1.52–1.34 (1H, m, H4'), 0.86 (9H, s, *t*Bu); ^{13}C NMR (126 MHz, $CDCl_3$) δ 154.1 (C=O), 136.6 (ArC), 130.3 (C6), 129.5 (C6), 128.5 (ArCH), 128.4 (ArCH), 128.1 (ArCH), 127.9 (ArCH), 114.2 (C5), 114.1 (C5), 67.5 ($CbzCH_2$), 67.3 ($CbzCH_2$), 46.5 (C1), 46.4 (C3), 29.3 (C2/C4), 29.0 (C2/C4), 27.6 (tBu), 27.6 (C2/C4), 27.2 (C2/C4); 19 signals observed; HRMS $[M + H]^+$ $C_{18}H_{26}NO_2$ requires 288.1958, found 288.1961

Benzyl 2,2-dimethyl-2,3-dihydro-1*H*-pyrrole-1-carboxylate

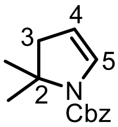

Benzyl 2,2-dimethyl-5-oxopyrrolidine-1-carboxylate (1.45 g, 5.88 mmol) was dissolved in anhydrous toluene (10 mL) and cooled to -78ºC and added Superhydride (1M solution in THF, 7.0 mL, 7.0 mmol). The mixture was allowed to stir at -78 ºC for 45 minutes followed by the sequential dropwise addition of DIPEA (5.1 mL 29.4 mmol), trifluoroacetic anhydride (1.0 mL, 7.0 mmol) and DMAP (10 mg). The mixture was stirred, allowing to warm from -78ºC to room temperate, for 16 hours. The reaction was diluted with water (50 mL) and ethyl acetate (50 mL), the layers separated and the aqueous further extracted with ethyl acetate (3 x 50 mL). The combined organic layers were dried over $MgSO_4$, evaporated *in vacuo* and the residue purified by column chromatography (10% ethyl acetate in hexane) resulting in a colourless oil (720 mg, % 48%) as a mixture of rotamers.

^{1}H NMR (501 MHz, Chloroform-*d*) δ $_H$ 7.32–7.21 (5H, m, Cbz), 6.59–6.35 (1H, m, H4), 5.13–5.02 (2H, m, $CbzCH_2$), 4.90–4.75 (1H, m, H5), 2.56–2.36 (2H, m, H3), 1.44 (3H, s, Me), 1.34 (3H, s, Me); ^{13}C NMR (126 MHz, $CDCl_3$) δ 153.5 (C=O), 151.4 (C=O), 136.8 (ArC), 130.9 (ArC), 128.9 (ArCH), 128.8 (ArCH), 128.51 (C5), 128.49 (C5), 128.1 (ArCH), 127.9 (ArCH), 105.2 (C4), 67.1 ($CbzCH_2$), 66.5 ($CbzCH_2$), 62.3 (C2), 62.1 (C2), 47.8, (C3) 46.4 (C3), 30.4, (Me) 29.0 (Me), 27.5 (Me), 26.4 (Me); 21 signals observed; HRMS: $C_{14}H_{17}NNaO_2$ $[M + Na]^+$ requires 254.1157, found 254.1171.

Benzyl 3-azaspiro[4.5]dec-4-ene-1-carboxylate

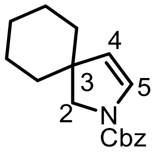

Benzyl 2-oxo-3-azaspiro[4.5]decane-1-carboxylate (3.52 g, 12.2 mmol) was dissolved in anhydrous toluene (20 mL) and cooled to -78ºC. To this was added a solution of Superhydride (1M in THF, 14.7 mL, 14.7 mmol). The mixture was allowed to stir at -78ºC for 45 minutes followed by the sequential dropwise addition of DIPEA (12.0 mL 69.5 mmol), trifluoroacetic anhydride (2.0 mL 14.0 mmol) and DMAP (10mg). The mixture was stirred, allowing to warm from -78ºC to room temperate, for 16 hours. The reaction was diluted with water (50 mL) and ethyl acetate (50 mL), the layers separated and the aqueous further extracted with ethyl acetate (3 x 50 mL). The combined organic layers were dried over

$MgSO_4$, evaporated *in vacuo* and the residue purified by column chromatography (15% ethyl acetate in hexane) resulting in a colourless oil (900mg, 27%) as a mixture of rotamers.

^{1}H NMR (501 MHz, Chloroform-*d*) δ $_H$ 7.42–7.31 (5H, m, Cbz), 6.58 (0.5H, d, *J* 4.3 H5), 6.48 (0.5H, d, *J* 4.3, *H5*), 5.18 (2H, s, $CbzCH_2$), 5.10 (0.5H, d, *J* 4.3, H4), 5.02 (0.5H, d, *J* 4.3, *H4*), 3.55 (1H, s, H2), 3.49 (1H, s, H2'), 1.60–1.34 (10H, m, cyclohexyl); ^{13}C NMR (126 MHz, $CDCl_3$) δ 152.8 (C=O), 152.2 (C=O), 136.6 (ArC), 135.3 (ArC), 128.6 (ArCH), 128.2 (ArCH), 128.1 (ArCH), 127.6 (C5), 127.0 (C5), 118.4 (C4), 118.1 (C4), 67.1 ($CbzCH_2$), 67.0 ($CbzCH_2$), 56.9 (C2), 47.9 (C3), 46.8 (C3), 37.6 (CH_2), 25.6 (CH_2), 22.9 (CH_2), 22.7 (CH_2); 20 signals observed; HRMS $[M + H]^+$ $C_{17}H_{22}NO_2$ requires 272.1645, found 272.1655.

Benzyl hexahydro-2*H*-isoindole-1-carboxylate

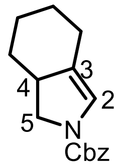

Benzyl octahydro-2*H*-isoindole-2-carboxylate (1.00 g 3.80 mmol) and tetraethylammonium tetrafluoroborate (85mg) were dissolved in anhydrous methanol (8 mL) in an Electrasyn reaction vial. The vial was equipped with graphite electrodes and subjected to 2.5 F mol^{-1} over 8 hours. After the reaction had completed the solvent was removed *in vacuo* and the residue dissolved in ethyl acetate (50ml). This solution was washed with water (2 x 20mL), brine (1 x 20 mL) and dried over $MgSO_4$; evaporation of the solvent *in vacuo* gave the crude aminoacetal. The crude material was dissolved in anhydrous DCM (20 mL) and DIPEA (1.7 mL, 10.0 mmol) was added. The solution was cooled to 0ºC followed by the dropwise addition of TMS triflate (1.88 mL, 10.0 mmol) over 2 minutes. The reaction was immediately quenched by the addition of hexane (20 mL) followed by filtration through celite eluting with hexane , The filtrate was evaporated *in vacuo* and the residue purified by column chromatography (10% ethyl acetate in hexane) and isolated as a colourless oil (358mg, 37%) as a mixture of rotamers which was used immediately in the next step.

^{1}H NMR (501 MHz, Chloroform-*d*) δ $_H$ 7.43–7.27 (5H, m, Cbz), 6.29 (0.5H, s, H2), 6.21 (0.5H, s, *H2*), 5.15 (2H, s, $CbzCH_2$), 4.02–3.89 (1H, m, H5), 3.42–3.20 (1H, m, H5'), 2.83-2.71 (1H, m, H4), 2.48–2.31 (1H, m), 2.01–1.89 (2H, m), 1.84–1.73 (2H, m), 1.40–1.11 (3H, m); ^{13}C NMR (126 MHz, $CDCl_3$) δ 152.7 (C=O), 152.1 (C=O), 137.0 (ArC), 136.9 (ArC), 128.6 (ArCH), 128.1 (ArCH), 128.1 (ArCH), 128.0 (ArCH), 126.7 (C3), 126.5 (C3), 120.3 (C2), 119.7 (C2), 67.0 ($CbzCH_2$), 66.8 ($CbzCH_2$), 52.1 (C5), 51.9 (C5), 43.1 (C4), 42.2 (C4),

34.6 (CH_2), 34.5 (CH_2), 27.48 (CH_2), 27.46 (CH_2), 25.8 (CH_2), 25.7 (CH_2), 25.4 (CH_2); 25 signals observed.

Aminations with primary amines (scheme 2)

Benzyl 3-(*iso*butylamino)pyrrolidine-1-carboxylate (**6a**)

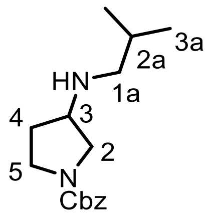

(**6a**) was synthesised using general method A using benzyl 2,3-dihydro-1H-pyrrole-1-carboxylate (50.0 mg, 0.25 mmol), *iso*butylamine (100μL, 1.25 mmol), TRIP thiol (27.0 mg, 0.125 mmol) and catalyst A (5.0 mg, 2 mol%). The reaction was irradiated for 16 hours followed by purification by column chromatography (2:1 to 1:1 hexane/ethyl acetate) and isolated as a colourless oil (55.0 mg, 79% yield) as a mixture of rotamers.

^{1}H NMR (501 MHz, Chloroform-*d*) δ_H 7.42 – 7.26 (5H, m, Cbz), 5.13 (2H, s, $CbzCH_2$), 3.67–3.58 (1 H, m, H2), 3.60–3.49 (1H, m, H5), 3.41 (1H, m, H5'), 3.31 (1H, app h, *J* 5.9, H3), 3.18 (0.5H, dd, *J* 10.9, 5.3, H2'), 3.13 (0.5H, dd, *J* 10.8, 5.7 H2'), 2.47–2.34 (2H, m, H1a), 2.12–2.00 (1H, m, H4), 1.79–1.61 (2H, m, H4' + H2a), 0.90 (6H, d, *J* 6.8, H3a). ^{13}C NMR (126 MHz, $CDCl_3$) δ_C 155.1 (C=O), 137.2 (ArC), 128.6 (ArCH), 128.0 (ArCH) 66.8 ($CbzCH_2$), 58.0 (C3), 57.1 (C3), 56.4 (C1a), 52.3 (C2), 52.0 (C2), 44.8 (C5), 44.5 (C5), 32.2 (C4), 31.5 (C4), 28.7 (C2a), 28.6 (C2a), 20.8 (C3a): 17 signals observed; $C_{16}H_{25}N_2O_2$ $[M + H]^+$ requires 277.1911, found 277.1921.

Benzyl 3-(*iso*butylamino)piperidine-1-carboxylate (**6b**)

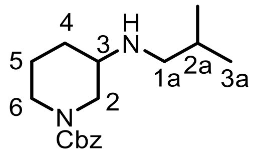

(**6b**) was synthesised using general method A using benzyl 3,4-dihydropyridine-1(2H)-carboxylate (55.0 mg, 0.25 mmol) *iso*butylamine (100μL, 1.25 mmol), TRIP thiol (27.0 mg, 0.125 mmol) and catalyst B (5.0 mg, 2mol%). The reaction was irradiated for 16 hours followed by purification by column chromatography (2:1 to 1:1 hexane/ethyl acetate) and isolated as a colourless oil (50 mg, 69% yield) as a mixture of rotamers.

^{1}H NMR (501 MHz, Chloroform-*d*) δ_H 7.43–7.28 (5H, m, Cbz), 5.13 (2H, s, $CbzCH_2$), 4.05 (1H, br s, H2), 3.88 (1H, s, H6), 2.95 (1H, br s, H6'), 2.81-2.61 (1H, m, H2'), 2.54 (1H, br s, H3), 2.45 (2H, br s, H1a), 1.98–1.89 (1H, m, H4), 1.68 (2H, br s, H2a+H5), 1.52–1.44 (1H, m, H5'), 1.30 (1H, br s, H4'), 0.88 (6H, d, *J* 6.7, H3a). ^{13}C NMR (126 MHz, $CDCl_3$) δ_C 155.4

(C=O), 137.0 (ArC), 128.5 (ArCH), 128.0 (ArCH), 127.8 (ArCH), 67.0 (CbzCH_2), 55.2 (C1a), 54.0 (C3), 49.6 (C2), 44.5 (C6), 31.5 (C4), 28.6 (C2a), 24.0 (C5), 23.4 (C5), 20.6 (C3a), 15 signal observed; HRMS $C_{17}H_{27}N_2O_2$ $[M + H]^+$requires 291.2067, found 291.2080

Benzyl 3-(*iso*butylamino)azepane-1-carboxylate (**6c**)

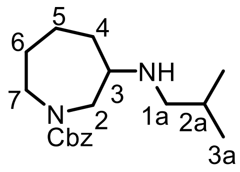

(**6c**) was synthesised using general method A using benzyl 2,3,4,5-tetrahydro-1H-azepine-1-carboxylate (55.0 mg, 0.26 mmol) *iso*butylamine (100 µL, 1.25 mmol), TRIP thiol (27.0 mg, 0.125 mmol) and catalyst B (5.0 mg, 2 mol%). The reaction was irradiated for 16 hours followed by purification by column chromatography (2:1 to 1:1 hexane/ethyl acetate) and isolated as a colourless oil (25mg, 33% yield) as a mixture of rotamers.

^{1}H NMR (501 MHz, Chloroform-*d*) δ_H 7.40–7.28 (5H, m, Cbz), 5.19–5.05 (2H, m, CbzCH_2), 3.85 (0.5H, dd, *J* 14.0, 4.2, H2), 3.78 (0.5H, dd, *J* 14.3, 3.6, *H2*), 3.71 (0.5H, ddd, *J* 13.7, 9.4, 5.9, H7), 3.59 (0.5H, ddd, *J* 13.7, 8.5, 5.1, *H7*), 3.33–3.28 (0.5H H, m, H7'), 3.29–3.21 (0.5H, m, H7'), 2.96 (0.5H, dd, *J* 13.9, 8.7 H2'), 2.86 (0.5H, dd, *J* 14.2, 9.0, H2'), 2.75 (0.5H, tt, *J* 8.6, 4.1, H3), 2.64 (0.5H, tt, *J* 9.1, 4.1, H3'), 2.50 (0.5H, dd, *J* 11.4, 6.8, H1a), 2.46 (0.5H, dd, *J* 11.4, 6.7, H1a), 2.35 (0.5H, dd, *J* 11.6, 6.8, H1a'), 2.31 (0.5H, dd, *J* 11.6, 6.9, H1a'), 1.90–1.66 (4H, m, H6+H5+H4), 1.65–1.55 (1H, m, H2a), 1.4–1.27 (2H, m, H5', H4'), 0.90 (3H, d, *J* 6.6, H3a), 0.81 (1.5H, d, *J* 4.5, H3a), 0.80 (1.5H, d, *J* 4.4, H3a); ^{13}C NMR (126 MHz, $CDCl_3$) δ_C 156.6 (C=O), 156.3 (C=O), 137.2 (ArC), 136.9 (ArC), 128.6 (ArCH), 128.16 (ArCH), 128.12 (ArCH), 128.0 (ArCH), 127.9 (ArCH), 67.3 (CbzCH_2), 67.1 (CbzCH_2), 58.8 (C3), 58.3 (C3), 55.81 (C1a), 55.77 (C1a), 51.6 (C2), 50.6 (C2), 47.6 (C7), 47.5 (C7), 35.3 (C4), 34.6 (C4), 28.8 (C2a), 28.7 (C2a), 27.9 (C5/6), 27.5 (C5/6), 23.0 (C5/6), 22.9 (C5/6), 20.9 (C3a), 20.8 (C3a), 20.7 (C3a); 30 signals observed; HRMS $C_{18}H_{29}N_2O_2$ $[M + H]^+$ requires 305.2224, found 305.2221

1-Benzyl-5-(*iso*butylamino)piperidin-2-one (**6d**)

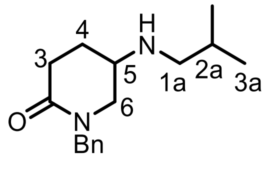

(**6d**) was synthesised using general method A using 1-benzyl-3,4-dihydropyridin-2(1H)-one (48.0 mg, 0.26 mmol) *iso*butylamine (100 µL, 1.25 mmol), TRIP thiol (27.0 mg, 0.125 mmol) and catalyst A (5.0 mg, 2 mol%). The reaction was irradiated for 16 hours followed by purification by column chromatography (2:1 to 1:1 hexane/ethyl acetate) and isolated as a colourless oil (41mg, 62%).

^{1}H NMR 501 MHz, Chloroform-*d*) δ $_H$ 7.35–7.21 (5H, m, Bn), 4.66 (1H, d, *J* 14.7, BnCH$_2$), 4.51 (1H, d, *J* 14.7 BnCH$_2$), 3.32 (1H, ddd, *J* 11.9, 4.5, 1.4, H6), 2.99 (1H, dd, *J* 11.9, 7.4, H6'), 2.91 (1H, dddd, *J* 8.9, 7.6, 4.6, 3.3, H5), 2.61 (1H, dt, *J* 17.8, 5.9, H3), 2.44 (1H, ddd, *J* 17.9, 9.1, 6.4, H3'), 2.37 (1H, dd, *J* 11.2, 6.9, H1a), 2.30 (1H, dd, *J* 11.1, 6.6. H1a), 2.05–1.95 (1H, m, H4), 1.73–1.65 (1H, m, H4'), 1.62 (1H, app sept, 6.7, H2a), 0.87 (3H, d, *J* 6.6, H3a), 0.86 (3H, d, *J* 6.7, H3a); ^{13}C NMR (126 MHz, CDCl$_3$) δ$_C$ 169.6 (C2), 137.1 (ArC), 128.6 (ArCH), 128.1 (ArCH), 127.4 (ArCH), 55.2 (C1a), 52.3 (C6), 52.0 (C5), 50.1 (BnCH$_2$), 29.8 (C3), 28.7 (C2a), 27.4 (C4), 20.6 (C3a), 20.6 (C3a); HRMS $C_{16}H_{25}N_2O$ $[M + H]^+$ requires 261.1961, found 261.1973.

Benzyl-5-(*iso*butylamino)-2-methylpiperidine-1-carboxylate (**6e**)

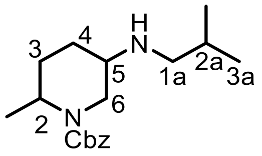

(**6e**) was synthesised using general method A using benzyl 2-methyl-3,4-dihydropyridine-1(2H)-carboxylate (58.0 mg, 0.25 mmol) *iso*butylamine (100 µL 1.25 mmol) TRIP thiol (27.0 mg 0.125 mmol) and catalyst A (5.0 mg, 2 mol%). The reaction was irradiated for 40 hours followed by purification by column chromatography (2:1 to 1:1 hexane/ethyl acetate) and isolated as a colourless oil (39 mg, 51%) as an inseparable 1:1 mixture of diastereomers, each of which presented as rotamers.

^{1}H NMR (501 MHz, Chloroform-*d*) δ $_H$ 7.38–7.28 (5H, m, Cbz), 5.17–5.06 (2H, m, CbzCH$_2$), 4.56–4.35 (1H, m, H2), 4.29–4.09 (0.5H, m, H6), 4.12–4.03 (0.5H, m, H6), 2.99 (0.5H, dd, *J* 13.9, 2.6, H6'), 2.78–2.70 (0.5H, m, H5), 2.56 (0.5 H, t, *J* 11.8, H6'), 2.51–2.38 (2H, m, H5+H1a+H1a'), 2.30 (0.5H, dd, *J* 11.2, 6.5, H1a'), 2.00–1.89 (0.5H, m, H3), 1.84–1.72 (1H, m H3'+ H3), 1.72–1.65 (1H, m, H2a), 1.65–1.52 (1.5H, m, H4+H4+H3'), 1.46–1.34 (0.5H, m (H4'), 1.32–1.22 (0.5H, m, H4'), 1.17 (1.5H, d, *J* 6.9, Me), 1.15 (1.5H, d, *J* 7.0, *Me*), 0.90 (3 H, d, *J* 6.6, H3a), 0.86 (1.5H, d, *J* 6.6, H3a), 0.84 (1.5H, d, *J* 6.6, H3a); ^{13}C NMR (126 MHz, CDCl$_3$) δ$_C$ 156.2 (C=O), 137.2 (ArC), 128.61 (ArCH), 128.59 (ArCH), 128.04 (ArCH), 128.01

(ArCH), 127.94 (ArCH), 127.88 (ArCH), 67.1 ($CbzCH_2$), 55.23 (C1a), 55.22 (C1a), 55.1 (*C5*), 51.6 (C5), 46.6 (C2), 46.1 (*C2*), 44.5 (C6), 41.7 (*C6*), 29.1 (C3), 28.8 (C2a), 28.7 (C2a), 26.7 (*C3*), 24.7 (C4), 24.1 (*C4*), 20.9 (C3a), 20.8 (C3a), 20.7 (C3a), 16.0 (Me); 27 signals observed; HRMS; $C_{18}H_{29}N_2O_2$ $[M + H]^+$requires 305.2224, found 305.2220

Benzyl 3-(*iso*butylamino)-4-methylpiperidine-1-carboxylate (**6f**)

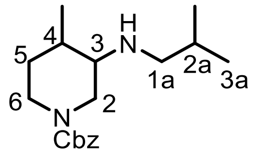

(**6g**) was synthesised using general method A using benzyl 4-methyl-3,4-dihydropyridine-1(2H)-carboxylate (62.0 mg, 0.27 mmol) *iso*butylamine (100 µL, 1.25 mmol), TRIP thiol (27.0 mg, 0.125 mmol) and catalyst A (5.0 mg, 2 mol%). The reaction was irradiated for 40 hours followed. The mixture was purified by SCX chromatography and isolated as a colourless oil as a 1:1.9 mixture of diastereomers (39 mg, 48%). Further purification by column chromatography (2:1 to 1:1 hexane/ethyl acetate) was conducted to separate diastereomers (7mg, 8% (minor); 15mg 18% (major))

Minor diastereomer

^{1}H NMR (501 MHz, Chloroform-*d*) δ_H 7.40–7.27 (5H, m, Cbz), 5.22–5.04 (2H, m, $CbzCH_2$), 4.03–3.64 (2H, m, H2/H6), 3.33–2.89 (2H, m, H2'/H6'), 2.66–2.12 (3H, m, H3+H1a), 1.82 (1H, br s, H4), 1.70–1.37 (3H, m, C5+C2a), 0.97 (3H, d, *J* 6.9, Me), 0.92–0.78 (6H, m, 3a); ^{13}C NMR (126 MHz, $CDCl_3$) δ_C 155.9 (C=O), 137.0 (ArC), 128.6 (ArCH), 128.5 (ArCH), 127.9 (ArCH), 67.0 ($CbzCH_2$), 56.6 (C3), 55.7 (C1a), 46.2 (C2/C6), 45.9 (C2/C6), 43.0 (C2/C6), 42.5 (C2/C6), 33.6 (C4), 33.3 (C4), 29.3 (C5), 28.9 (C5), 28.6 (C2a), 20.7 (C3a), 20.6 (C3a), 16.3 (Me), 15.6 (Me). 21 signals observed; HRMS; $C_{18}H_{29}N_2O_2$ requires 305.2224; found 305.2224.

Major diastereomer

^{1}H NMR (501 MHz, Chloroform-*d*) δ_H 7.34–7.23 (5H, m, Cbz), 5.25–4.81 (2H, m, $CbzCH_2$), 4.21 (1H, br d, H2), 4.03–3.89 (1H, m, H6), 2.74 (1H, t, *J* 12.9, H6'), 2.53–2.26 (3H, m, H2'+H1a), 2.04 (1H, br s, H3), 1.66–1.51 (2H, m, H5+H2a), 1.45–1.28 (2H, m, H5'+H4), 0.94 (3H, d, *J* 6.5, Me), 0.83 (6H, d, *J* 6.6, H3a); ^{13}C NMR (126 MHz, $CDCl_3$) δ_C 155.4 (C=O), 137.1 (ArC), 128.6 (ArCH), 128.1 (ArCH), 127.9 (ArCH), 67.1 ($CbzCH_2$), 60.7 (C3), 55.7 (C1a), 48.8 (C2), 44.1 (C6), 36.5 (C4), 33.3 (C5), 32.9 (C5), 28.8 (C2a), 20.7 (C3a), 20.7 (C3a), 18.5 (Me); 16 signals observed; HRMS $C_{18}H_{29}N_2O_2$ $[M + H]^+$ requires 305.2224; found 305.2218.

Benzyl 5-(*tert*-butyl)-3-(*iso*butylamino)azepane-1-carboxylate (**6g**)

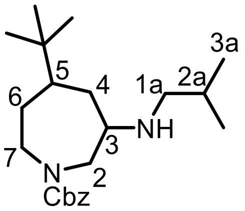

(**6g**) was synthesised using general method A using benzyl 4-(tert-butyl)-2,3,4,5-tetrahydro-1H-azepine-1-carboxylate (35.0 mg, 0.125 mmol) *iso*butylamine (100 µL, 1.25 mmol) TRIP thiol (13.5 mg, 0.125mmol) and catalyst B (2.5 mg, 2 mol%). The reaction was irradiated for 16 hours followed by purification by column chromatography (2:1 to 1:1 hexane/ethyl acetate) and isolated as a colourless oil (18mg, 39%) as a mixture of rotamers.

^{1}H NMR (501 MHz, Chloroform-*d*) δ_H 7.40–7.28 (5H, m, Cbz), 5.16–5.11 (2H, m, $CbzCH_2$), 4.05–3.70 (2H, m, H2+H7), 3.27–2.86 (3H, m, H2+H3+H7), 2.54–2.26 (2H, m, H1a), 1.94–1.57 (3H, m, H4+H5+H6), 1.48–1.12 (3H, m, H4'+H6'+H2a), 1.00–0.70 (15H, m, H3a+ ^{t}Bu); ^{13}C NMR (126 MHz, $CDCl_3$) δ_C 156.5 (C=O), 155.9 (C=O), 137.2 (ArC), 137.1 (ArC), 128.6 (ArCH), 128.1 (ArCH), 128.0 (ArCH), 127.9 (ArCH), 67.1 ($CbzCH_2$), 55.78 (C1a), 55.73 (C1a), 55.2 (C3), 54.9 (C3), 51.7 (C7), 51.4 (C7), 49.9 (C2), 49.1 (C2), 42.2 (C5), 33.64 (C ^{t}Bu), 33.62 (C ^{t}Bu), 33.3 (C4), 30.7 (C6), 30.5 (C6), 28.7 (CH_3), 28.4 (CH_3), 27.6 (CH_3), 20.9 (CH_3), 20.8 (CH_3); 28 signals observed; HRMS $C_{22}H_{37}N_2O_2$ [M+H] requires 361.2850, found 361.2864.

Benzyl 4-(*iso*butylamino)-2,2-dimethylpyrrolidine-1-carboxylate (**6h**)

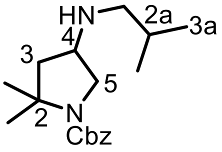

(**6h**) was synthesised using general method A using benzyl 2,2-dimethyl-2,3-dihydro-1H-pyrrole-1-carboxylate (61.0 mg, 0.27 mmol) *iso*butylamine (100 µL, 1.25 mmol), TRIP thiol (27.0 mg, 0.125 mmol) and catalyst A (5.0 mg, 2 mol%). The reaction was irradiated for 16 hours followed by purification by column chromatography (1:1 hexane/ethyl acetate) and isolated as a colourless oil (52.0 mg, 64%) as a mixture of rotamers.

^{1}H NMR (501 MHz, Chloroform-*d*) δ_H 7.38–7.28 (5H, m, Cbz), 5.16–4.97 (2H, m, $CbzCH_2$), 3.92–3.79 (1H, m, H5), 3.28 (1H, p, *J* 7.7, H4), 3.17–3.06 (1H, m, H5'), 2.50–2.31 (2H, m,

C1a), 2.07 (1H, dd, *J* 12.6, 6.2, H3), 1.73–1.59 (2H, m, H3'+H2a), 1.48–1.26 (6H, m, 2xMe), 0.90 (6H, d, *J* 6.7, H3a); ^{13}C NMR (126 MHz, $CDCl_3$) δ_C 153.6, (C=O) 137.3 (ArC), 128.5 (ArCH), 128.0 (ArCH), 127.8 (ArCH), 127.7 (ArCH), 66.9 ($CbzCH_2$), 66.1 ($CbzCH_2$), 60.6 (C2), 60.0 (C2), 56.6 (C1a), 54.6 (C4), 54.5 (C4), 54.0 (C5), 53.7 (C5), 49.4 (C3), 48.4 (C3), 28.7 (C2a), 28.6 (C2a), 27.6, (CH_3) 27.4 (CH_3), 26.3 (CH_3), 20.7 (C3a); 23 signals observed; HRMS $C_{18}H_{29}N_2O_2$ $[M + H]^+$requires 305.2226; found 305.2235.

Benzyl 4-(*iso*butylamino)-2-azaspiro[4.5]decane-2-carboxylate (**6i**)

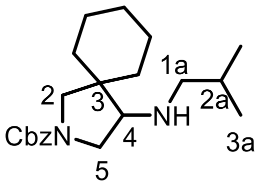

(**6i**) was synthesised using general method A using benzyl 2-azaspiro[4.5]dec-3-ene-2-carboxylate (70.0 mg, 0.26 mmol) *iso*butylamine (100 µL, 1.25mmol), TRIP thiol (27.0 mg, 0.125 mmol) and catalyst A (5.0 mg, 2 mol%). The reaction was irradiated for 16 hours followed by purification by column chromatography (2:1 to 1:3 hexane/ethyl acetate) and isolated as a colourless oil (47.0 mg, 53%) as a mixture of rotamers.

^{1}H NMR (500 MHz, Chloroform-*d*) δ_H 7.42–7.28 (5H, m, Cbz), 5.16–5.09 (2H, m, $CbzCH_2$), 3.69 (0.5H, dd, *J* 11.0, 6.4, H5), 3.66 (0.5H, dd, *J* 13.5, 6.5, H5), 3.56 (0.5H, d, *J* 10.9, H2), 3.47 (0.5H, d, *J* 10.8, H2), 3.18 (0.5H, dd, *J* 11.0, 6.2, H5'), 3.15-3.08 (1.5H, m, H2'+*H5'*), 2.85–2.71 (1H, m, H4), 2.46 (0.5H, dd, *J* 11.4, 6.8, H1a), 2.42 (0.5H, dd, *J* 11.2, 6.7, *H1a*), 2.33 (1H, dd, *J* 11.4, 6.6, H1a'), 1.80-1.73 (1H, m, H2a), 1.72–1.55 (4H, m, spyro), 1.55-1.48 (2H, m, cyclohexyl), 1.36–1.20 (4H, m, cyclohexyl), 0.89 (6H, dd, *J* 6.7, 2.4, H3a); ^{13}C NMR (126 MHz, $CDCl_3$) δ_C 155.4 (C=O), 155.3 (C=O), 137.2 (ArC), 128.6 (ArCH), 128.0 (ArCH), 66.8 ($CbzCH_2$), 65.6 (C4), 64.5 (C4), 57.2 (C1a), 57.1 (C1a), 54.3 (C2), 54.0 (C2), 50.6 (C5), 50.2 (C5), 44.9 (C3), 44.1 (C3), 36.4 (cyclohexyl), 35.2 (cyclohexyl), 35.1 (cyclohexyl), 28.8 (C2a), 28.7 (C2a), 28.1 (cyclohexyl), 27.9 (cyclohexyl), 26.3 (cyclohexyl), 26.2 (cyclohexyl), 23.6 (cyclohexyl), 23.5 (cyclohexyl), 22.9 (cyclohexyl), 22.8 (cyclohexyl), 20.8 (C3a), 20.7 (C3a); 31 signals observed; HRMS $C_{21}H_{33}N_2O_2$ $[M + H]^+$requires 345.2537, found 345.2544.

Benzyl 3a-(*iso*butylamino)octahydro-2*H*-isoindole-1-carboxylate (**6j**)

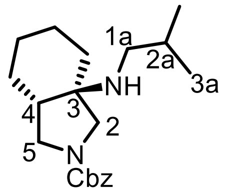

(**6j**) was synthesised using general method A using (ENECARB) (32.0 mg, 0.125 mmol) *iso*butylamine (100 µL, 1.25 mmol), TRIP thiol (13.0 mg, 0.0625 mmol) and catalyst A (2.5 mg, 2 mol%). The reaction was irradiated for 16 hours followed by purification by column chromatography (2:1 hexane/ethyl acetate) and isolated as a colourless oil (16.0 mg, 40%) as a mixture of rotamers. The stereochemistry was assigned through positive NOESY interaction between H1a and H4.

^{1}H NMR (501 MHz, Chloroform-*d*) δ_H 7.62–7.23 (5H, m, Cbz), 5.22–4.90 (2H, m, CbzCH$_2$), 3.57 (1H, app dt, *J* 10.5, 7.7, H5), 3.39 (0.5H, d, *J* 10.8, H2), 3.36–3.26 (1.5H, m, H2+H5', 3.24 (0.5H, dd, *J* 9.7, H2'), 3.22 (0.5H, d, *J* 10.8, H2'), 2.41–2.32 (1H, m, H1a), 2.30–2.18 (1H, m, H1a'), 2.05–1.94 (1H, m, H4), 1.69–1.30 (9H, m, H2a+ 4xCH$_2$), 0.94–0.85 (6H, m, H3a); ^{13}C NMR (126 MHz, CDCl$_3$) δ_C 155.8, (C=O) 155.7 (*C=O*), 137.2 (ArC), 128.6 (ArCH), 128.0 (ArCH), 127.97 (ArCH), 127.9 (*ArCH*), 66.8 (CbzCH$_2$), 66.8 (CbzCH$_2$), 60.5 (C3), 59.8 (C3), 54.9 (C5), 54.7 (C5), 50.5 (C1a), 50.4 (C1a), 49.6 (C2), 49.4 (C2), 42.3 (C4), 41.3 (C4), 29.6 (C2a), 29.5 (CH$_2$), 25.3 (CH$_2$), 22.4 (CH$_2$), 22.3 (CH$_2$), 22.1 (CH$_2$), 22.0 (CH$_2$), 20.9 (C3a); 27 signals observed; $C_{20}H_{31}N_2O_2$ requires 331.2382; found 331.2379

Benzyl 3-(methylamino)pyrrolidine-1-carboxylate (**6k**)

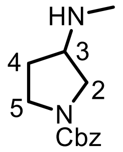

(**6k**) was synthesised using general method A benzyl 2,3-dihydro-1H-pyrrole-1-carboxylate (51.0 mg, 0.125 mmol) methylamine (100 µL, 2 mmol) TRIP thiol (27 mg, 0.25mmol) and catalyst A (5.0 mg, 2 mol%). The reaction was irradiated for 16 hours followed by purification by column chromatography (2:1 to 1:1 hexane/ethyl acetate) and isolated as a colourless oil (38mg, 56%) as a mixture of rotamers.

^{1}H NMR (501 MHz, Chloroform-*d*) δ_H 7.57 – 7.20 (5H, m, Cbz), 5.13 (2H, d, *J* 3.5, CbzCH$_2$), 3.63 – 3.49 (2H, m, H2+H5), 3.49 – 3.39 (1H, m, H5'), 3.29 – 3.16 (2H, m, H3+H2'), 2.46-

2.40 (3H, m, NMe), 2.12 – 2.00 (1 H, m, H4), 1.81 – 1.70 (1 H, m, H4'); ^{13}C NMR (126 MHz, $CDCl_3$) δ_C 155.1 (C=O), 137.1 (ArC), 128.6 (ArCH), 128.1 (ArCH), 128.0 (ArCH), 66.9 ($CbzCH_2$), 59.7 (C3), 58.8 (C3), 51.8 (C5), 51.5 (C5), 44.7 (C2) 44.4 (C2), 34.8 (NMe), 31.8 (C4), 31.1 (C4); 15 signals observed; HRMS $C_{13}H_{19}N_2O_2$ [M + H] requires 235.1441; find 235.1440

Benzyl 3-(butylamino)pyrrolidine-1-carboxylate (**6l**)

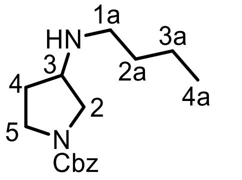

(**6l**) was synthesised using general method A benzyl 2,3-dihydro-1H-pyrrole-1-carboxylate (51.0 mg, 0.125 mmol), butylamine (100 µL, 1.25 mmol) TRIP thiol (27 mg, 0.25mmol) and catalyst A (5.0 mg, 2 mol%). The reaction was irradiated for 16 hours followed by purification by column chromatography (2:1 to 1:1 hexane/ethyl acetate) and isolated as a colourless oil (38mg, 56%) as a mixture of rotamers.

^{1}H NMR (501 MHz, Chloroform-*d*) δ_H 7.40 – 7.27 (5H, m, Cbz), 5.16 – 5.01 (2H, m, $CbzCH_2$), 3.68 – 3.61 (1H, m, H2), 3.60–3.50 (1H, m, H5), 3.45 – 3.38 (1H, m, H5'), 3.39 – 3.30 (1H, m, H3), 3.23 (0.5H, dd, *J* 10.9, 5.4, H2'), 3.17 (0.5H, dd, *J* 10.8, 6.1, *H2'*), 2.65 – 2.42 (2H, m, H2a), 2.14 – 2.03 (1H, m, H4), 1.88 – 1.68 (1H, m, H4'), 1.53 – 1.22 (4H, m. H3a+H4a), 0.91 (3H, t, *J* 7.3, H5a); ^{13}C NMR (126 MHz, $CDCl_3$) δ_C 155.1 (C=O), 137.1 (ArC), 128.7 (ArCH), 128.6 (ArCH), 128.1 (ArCH), 128.0 (ArCH), 66.9 ($CbzCH_2$), 57.9 (C3), 57.1 (C3), 51.9 (C2), 51.6 (C2), 48.1 (C2a), 44.8 (C5), 44.4 (C5), 32.2 (CH_2), 32.1 (CH_2), 32.0 (C4), 31.3 (C4), 20.6 (CH_2), 14.1 (C5a); 20 signals observed, HRMS $C_{16}H_{25}N_2O_2$ [M + H]$^+$ requires 277.1911, found 277.1911.

Benzyl 3-(benzylamino)pyrrolidine-1-carboxylate (**6m**)

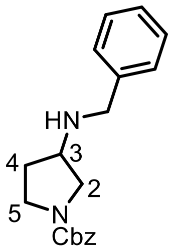

(**6m**) was synthesised using general method A using benzyl 2,3-dihydro-1H-pyrrole-1-carboxylate (52.0 mg, 0.25 mmol), benzylamine (107 mg, 1.00 mmol), TRIP thiol (27.0 mg,

0.125 mmol) and catalyst A (5.0 mg, 2 mol%). The reaction was irradiated for 16 hours followed by purification by column chromatography (2:1 to 1:1 hexane/ethyl acetate) and isolated as a colourless oil (22.0 mg, 28%) as a mixture of rotamers.

^{1}H NMR (501 MHz, Chloroform-*d*) δ $_H$ 7.50–7.05 (10H, m, ArCH), 5.13 (2H, s, CbzCH$_2$), 3.85 – 3.77 (2H, m, BnCH$_2$), 3.59 (2H, m, H2+H5), 3.40 (2H, m, H2'+H3), 3.23 (0.5H, dd, J 11.0, 5.1, H2'), 3.20 (0.5H, dd, J 10.8, 5.4, H2'), 2.10–1.98 (1H, m, H4) 1.85–1.66 (1H, m, H4'); ^{13}C NMR (126 MHz, CDCl$_3$) δ$_C$ 155.0 (C=O), 140.0 (ArC), 139.9 (ArC), 137.0 (ArCH), 128.5 (ArCH), 128.5 (ArCH), 128.12 (ArCH), 128.08 (ArCH), 127.99 (ArCH), 127.94 (ArCH), 127.2 (ArCH), 66.7 (CbzCH$_2$), 57.1 (C3), 56.2 (C3), 52.2 (BnCH$_2$), 52.1 (C2), 51.8 (C2) , 44.7 (C5), 44.3 (C5), 32.1 (C4), 31.4 (C4); 21 signals observed; HRMS; $C_{19}H_{22}N_2NaO_2$ [M + Na]$^+$ requires 333.1573, found 333.1578.

Benzyl 3-((furan-2-ylmethyl)amino)pyrrolidine-1-carboxylate (**6n**)

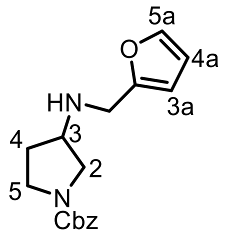

(**6n**) was synthesised using general method A using benzyl 2,3-dihydro-1H-pyrrole-1-carboxylate (51.0 mg, 0.25 mmol), 2-aminomethylfuran (120 mg, 1.25 mmol), TRIP thiol (27.0 mg, 0.125 mmol) and catalyst A (5.0 mg, 2 mol%). The reaction was irradiated for 16 hours followed by purification by column chromatography (2:1 to 1:1 hexane/ethyl acetate) and isolated as a colourless oil (22.0 mg, 29%) as a mixture of rotamers.

^{1}H NMR (501 MHz, Chloroform-*d*) δ $_H$ 7.42–7.28 (6H, m, H5a+Cbz), 6.31 (1H, dd, *J* 3.2, 1.9, H4a), 6.18 (1H, br s, H3a), 5.18-5.12 (2H, m, CbzCH$_2$), 3.82 (1H, d, *J* 14.6, BnCH$_2$), 3.79 (1H, d *J* 14.6, BnCH$_2$), 3.63–3.51 (2H, m H2+ H5), 3.46–3.38 (1H, m, H5), 3.39–3.29 (1H, m, H3), 3.23 (0.5H, dd, *J* 10.9, 5.2, H2') 3.17 (0.5H, dd, *J* 10.9, 5.2, H2'), 2.10–1.97 (1H, m, H4), 1.82–1.67 (1H, m, H4'); ^{13}C NMR (126 MHz, CDCl$_3$) δ$_C$ 155.0 (C=O), 153.4 (ArC), 142.2 (C5a), 137.1 (ArC), 128.6 (ArCH), 128.0 (ArCH), 128.0 (ArCH), 110.4 (C4a), 107.4 (C3a), 107.3 (C3a), 66.8 (CbzCH$_2$), 56.8 (C3), 55.9 (C3), 52.0 (C2), 51.7 (C2), 44.8 (BnCH$_2$), 44.7 (BnCH$_2$), 44.6 (C2), 44.4 (C2), 32.1 (C4), 31.4 (C4); 21 signals observed; HRMS $C_{17}H_{21}N_2O_3$ [M + H]$^+$ requires 301.1547, found 301.1545.

Benzyl 3-(*iso*propylamino)pyrrolidine-1-carboxylate (**6o**)

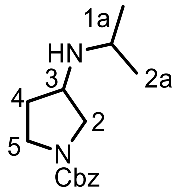

(**6o**) was synthesised using general method A using benzyl 2,3-dihydro-1H-pyrrole-1-carboxylate (58.0 mg, 0.29 mmol), *iso*propyl amine (100 µL, 1.25 mmol), TRIP thiol (27.0 mg, 0.125 mmol) and catalyst A (5.0 mg, 2 mol%). The reaction was irradiated for 16 hours followed by purification by column chromatography (2:1 to 1:1 hexane/ethyl acetate) and isolated as a colourless oil (37.0 mg, 50%) as a mixture of rotamers.

^{1}H NMR (501 MHz, Methanol-*d*$_4$) δ $_H$ 7.41–7.20 (5H, m, Cbz), 5.11 (2H, s CbzCH$_2$), 3.67 (0.5H, dd, J 12.6, 6.7, H2). 3.67 (0.5H, dd, J 12.6, 6.7, *H2*), 3.61–3.50 (1 H, m, H5), 3.45 (1 H, app h, *J* 6.9, H3), 3.41–3.32 (1H, m, H5'), 3.14–3.06 (1H, m, H1'), 2.94–2.81 (1H, m, H1a), 2.20–2.07 (1H, m, H4), 1.80–1.65 (1H, m, H4'), 1.12–1.04 (6H, m, H6); δ $_C$ (126 MHz, Methanol-*d*$_4$) 156.7 (C=O), 138.2 (ArC), 129.5 (ArCH), 129.1 (ArCH), 128.9 (ArCH), 68.0 (CbzCH$_2$), 55.8 (C3), 55.1 (C3), 52.6 (C2), 52.3 (C2), 48.0 (C6), 47.9 (C6), 45.7 (C5), 45.4 (C5), 32.5 (C4), 31.7 (C4), 22.7 (C7), 22.6 (C7); 18 signals observed; HRMS $C_{15}H_{23}N_2O_2$ [M + H]$^+$ requires 263.1754, found 263.1755.

Benzyl 3-((3-ethoxy-3-oxopropyl)amino)pyrrolidine-1-carboxylate (**6p**)

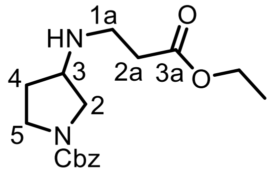

(**6p**) was synthesised using general method B using benzyl 2,3-dihydro-1H-pyrrole-1-carboxylate (51.0 mg, 0.25 mmol), ethyl 3-aminopropionate hydrochloride (75.0 mg, 0.49 mmol), TRIP thiol (27.0 mg, 0.125 mmol), lithium hydroxide monohydrate (21.0 mg, 0.50 mmol) and catalyst A (5.0 mg, 2 mol%). The reaction was irradiated for 16 hours followed by purification by column chromatography (2:1 to 1:1 hexane/ethyl acetate) and isolated as a colourless oil (46mg, 58%) as a mixture of rotamers.

^{1}H NMR (501 MHz, Chloroform-*d*) δ $_H$ 7.52–7.33 (5H, m, Cbz), 5.14 (2H, app d, *J* 3.4, CbzCH$_2$), 4.15 (2H, q, *J* 7.2, OCH$_2$), 3.68–3.57 (1H, m, H2), 3.58–3.52 (1H, m, H5), 3.48–3.41 (1H, m, H5'), 3.37-3.31 (1H, m, H3), 3.21 (0.5H, dd, J 11.0, 5.1, *H2'*), 3.16 (0.5H, dd, J 10.8, 5.5, *H2'*), 2.95–2.81 (2H, m, H1a), 2.55–2.47 (2H, m, H2a), 2.13–2.02 (1H, m, H2),

1.82–1.67 (1H, m, H2'), 1.27 (3H, t, *J* 7.2, CH_3); ^{13}C NMR (126 MHz, $CDCl_3$) δ_C 172.8 (C=O), 155.1 (C=O), 137.1 (ArC), 128.6 (ArCH), 128.04 (ArCH), 128.00 (ArCH), 66.8 ($CbzCH_2$), 60.6 (OCH_2), 57.8 (C3), 56.9 (C3), 52.2 (C2), 51.9 (C2), 44.8 (C5), 44.4 (C5), 43.5 (C1a), 34.9 (C2a), 32.1 (C4), 31.4 (C4), 14.3 (CH_3): 19 signals observed; HRMS $C_{17}H_{25}N_2O_4$ $[M + H]^+$ requires 321.1809, find 321.1817.

Benzyl 3-((2-ethoxy-2-oxoethyl)amino)pyrrolidine-1-carboxylate (**6q**)

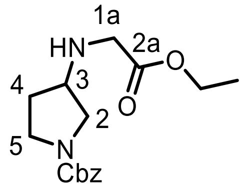

(**6q**) was synthesised using general method B using benzyl 2,3-dihydro-1H-pyrrole-1-carboxylate (52.0 mg, 0.25 mmol) glycine ethyl ester hydrochloride (70 mg, 0.50 mmol), TRIP thiol (27.0 mg, 0.125 mmol), lithium hydroxide monohydrate (21 mg, 0.50 mmol) and catalyst A (5.0 mg, 2 mol%). The reaction was irradiated for 16 hours followed by purification by column chromatography (2:1 to 1:1 hexane/ethyl acetate) and isolated as a colourless oil (38.0 mg, 50%) as a mixture of rotamers.

^1H NMR (501 MHz, Chloroform-*d*) δ_H 7.43–7.28 (5H, m, Cbz), 5.22–5.00 (2H, m, $CbzCH_2$), 4.19 (2H, q, *J* 7.2, OCH_2), 3.62–3.50 (2H, m, H2+H5), 3.49–3.40 (1H, m, H1'), 3.42–3.38 (2H, m, H5), 3.38–3.31 (1H, m, H3), 3.25 (0.5H, dd, J 11.1, 4.5), 3.19 (0.5H, dd, J 10.9, 5.0), 2.10–1.96 (1H, m, H4), 1.81–1.72 (1H, m, H4'), 1.30–1.25 (3H, m, CH_3); ^{13}C NMR (126 MHz, $CDCl_3$) δ_C 172.4 (C2a), 155.1 (C=O), 155.0 (C=O), 137.1 (ArC), 128.6 (ArCH), 128.1 (ArCH), 128.0 (ArCH), 66.9 ($CbzCH_2$), 61.1 (OCH_2), 57.4 (C3), 56.5 (C3), 52.0 (C2), 51.7 (C2), 49.3 (C1a), 49.3 (C1a), 44.7 (C5), 44.3 (C5), 32.1 (C4), 31.4 (C4), 14.3 (CH_3): 20 signals observed; HRMS $C_{16}H_{22}N_2O_4$ $[M + H]^+$ requires 307.1652, found 307.1652.

Benzyl 3-(((S)-1-ethoxy-1-oxo-3-phenylpropan-2-yl)amino)pyrrolidine-1-carboxylate (**6r**)

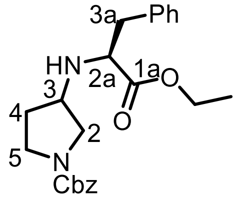

(**6r**) was synthesised using general method B using benzyl 2,3-dihydro-1H-pyrrole-1-carboxylate (53.0 mg, 0.26 mmol) L-phenylalanine ethyl ester hydrochloride (118 mg, 0.50 mmol), TRIP thiol (27.0 mg, 0.125 mmol), lithium hydroxide monohydrate (21.0 mg, 0.50 mmol) and catalyst A (5.0 mg, 2 mol%). The reaction was irradiated for 16 hours followed by purification by column chromatography (2:1 to 1:1 hexane/ethyl acetate) and isolated as a colourless oil (66.0 mg, 64%) as a 1:1 mixture of diastereomers which presented as rotamers.

^{1}H NMR (501 MHz, Chloroform-*d*) δ_H 7.42–7.28 (5H, m, Cbz), 7.24–7.14 (5H, m, Ph), 5.15–5.06 (2H, m, $CbzCH_2$), 4.26–4.03 (2H, m, OCH_2), 3.61–3.44 (2H, m, H2+H5), 3.44–3.30 (2H, m, H2a + H5'), 3.29–3.19 (1H, m, H3), 3.19–2.98 (1H, m, H2'), 2.97–2.84 (2H, m, H3a), 2.04–1.94 (0.5H, m, H4), 1.87 (0.5H, m, H4), 1.71–1.64 (0.5H, m, H4'), 1.60–1.50 (0.5H, m, H4'), 1.26–1.05 (3H, m, CH_3); ^{13}C NMR (126 MHz, $CDCl_3$) δ_C 175.0 (C1a), 174.9 (C1a), 174.8 (C1a), 174.7 (C1a), 155.0 (C=O), 137.5 (ArC), 137.3 (ArC), 137.2 (ArC), 137.1 (ArC), 129.41 (ArCH), 129.36 (ArCH), 129.3 (ArCH), 128.59 (ArCH), 128.57 (ArCH), 128.55 (ArCH), 128.52 (ArCH), 128.50 (ArCH), 128.1 (ArCH), 128.06 (ArCH), 128.03 (ArCH), 126.9 (ArCH), 66.8 ($CbzCH_2$), 61.6 (C2a), 61.44 (C2a), 61.41 (C2a), 61.3 (C2a), 60.97 (OCH_2), 60.93 (OCH_2), 60.9 (OCH_2), 56.4 (C3), 56.2 (C3), 55.4 (C3), 55.2 (C3), 52.8 (C2), 52.4 (C2), 51.7 (C2), 51.4 (C2), 44.9 (C5), 44.6 (C5), 44.4 (C5), 44.2 (C5), 40.3 (C3a), 40.2 (C3a), 40.2 (C3a), 32.9 (C4), 32.2 (C4), 31.4 (C4), 30.8 (C4), 14.3 (CH_3): 49 signals observed; HRMS $C_{23}H_{28}N_2O_4$ $[M + H]^+$ requires 397.2122, found 397.2138.

Benzyl 3-((2-((*tert*-butoxycarbonyl)amino)ethyl)amino)pyrrolidine-1-carboxylate (**6s**)

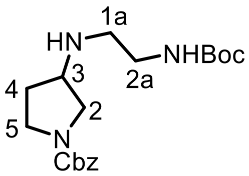

(**6s**) was synthesised using general method A using benzyl 2,3-dihydro-1H-pyrrole-1-carboxylate (52.0 mg, 0.25 mmol), *N*-Boc-ethylenediamine (75.0 mg, 0.49 mmol), TRIP thiol (27.0 mg, 0.125 mmol) and catalyst A (5.0 mg, 2 mol%). The reaction was irradiated for 16 hours followed by purification by column chromatography (2:1 to 1:1 hexane/ethyl acetate) and isolated as a colourless oil (49mg, 54%) as a mixture of rotamers.

^{1}H NMR (501 MHz, Chloroform-*d*) δ_H 7.38–7.28 (5H, m, Cbz), 5.14-5.10 (2H, m $CbzCH_2$), 3.65–3.50 (2H, m, H2 + H5), 3.45–3.23 (2H, m, H3 + H5'), 3.22–2.98 (3H, m, H2' + H2a), 2.78–2.52 (2H, m H1a), 2.09–1.93 (1H, m, H2), 1.8 –1.64 (1H, m, H2'), 1.44 (9H, s, Boc); ^{13}C NMR (126 MHz, $CDCl_3$) δ_C 156.2 (C=O), 155.1 (C=O), 149.2 (C=O), 148.1 (C=O), 137.1

(ArC), 137.0 (ArC), 128.64 (ArCH), 128.61 (ArCH), 128.24 (ArCH), 128.19 (ArCH), 128.1 (ArCH), 128.0 (ArCH), 79.5 (OBoc), 66.9 ($CbzCH_2$), 60.8 (C3), 59.8 (C3), 57.6 (C3), 56.7 (C3), 52.2 (C2), 51.9 (C2), 48.3 (C2), 48.0 (C1a), 47.5 (C1a), 40.8 (C5), 40.6 (C5), 32.2 (C2a), 31.4 (C2a), 28.5 (Boc): 28 signals observed; HRMS $C_{19}H_{30}N_3O_4$ $[M + H]^+$ requires 364.2231, found 364.2229.

Aminations with ammonia (and decoration) (Scheme 3)

Benzyl 3-aminopyrrolidine-1-carboxylate (**7a**)

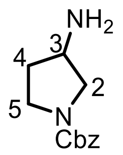

(**7a**) was synthesised using general method D using benzyl 2,3-dihydro-1H-pyrrole-1-carboxylate (51.0 mg, 0.25 mmol), ammonia (7M solution in methanol, 1mL), TRIP thiol (27.0 mg, 0.125 mmol) and catalyst A (5.0 mg, 2mol%). The reaction was irradiated for 16 hours followed by purification by strong cation exchange chromatography (1g benzene sulfonic acid bonded resin H^+ form pre equilibrated with 3x column volumes of methanol. Sample loaded in methanol (ca 2ml) and washed with 10x column volumes of methanol. Compound was eluted with 10x column volumes of methanolic ammonia (7M)) and isolated as a colourless oil (50mg, 72%) as a mixture of rotamers.

^{1}H NMR (501 MHz, Chloroform-*d*) δ_H 7.34–7.19 (5H, m, Cbz), 5.11 (2H, s, $CbzCH_2$), 3.63–3.49 (3H, m, H5+H3+H2), 3.50–3.36 (1H, m, H2'), 3.23–3.06 (1H, m, H5'), 2.16–2.04 (1H, m, H4), 1.80–1.67 (1H, m, H4'); ^{13}C NMR (126 MHz, $CDCl_3$) δ_C 156.8 (C=O), 138.3 (ArC), 129.5 (ArCH), 129.1 (ArCH), 128.9 (ArCH), 68.0 ($CbzCH_2$), 54.8 (C5), 54.4 (C5), 52.1 (C3), 51.4 (C3), 45.7 (C2), 45.4 (C2), 34.6 (C4), 34.0 (C4):14 signals observed; HRMS; $C_{12}H_{17}N_2O_2$ [M + H] requires 221.1285, found 221.1279.

Benzyl 3-aminopiperidine-1-carboxylate (**7b**)

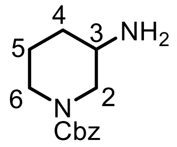

(**7b**) was synthesised using general method D using benzyl 3,4-dihydropyridine-1(2H)-carboxylate (55.0 mg, 0.25 mmol), ammonia (7M solution in methanol, 1 mL), TRIP thiol (27.0 mg, 0.125 mmol) and catalyst A (5.0 mg, 2 mol%). The reaction was irradiated for 16 hours followed by purification by strong cation exchange chromatography (1g benzene sulfonic acid bonded resin H^+ form pre equilibrated with 3x column volumes of methanol. Sample loaded in methanol (ca 2ml) and washed with 10x column volumes of methanol. Compound was eluted with 10x column volumes of methanolic ammonia (7M)) and isolated as a colourless oil (32mg, 54%) as a mixture of rotamers.

^{1}H NMR (501 MHz, Methanol-d_4) δ_H 7.39–7.21 (5H, m, Cbz), 5.11 (2H, s, CH_2Cbz), 4.10-4.01 (1H, m, H2), 3.92–3.85 (1H, m, H6), 2.91 (1H, br s, H6'), 2.71 (2H, br s, H2+H3), 1.99–1.89 (1H, m, H4), 1.77–1.67 (1H, m, H5), 1.51–1.43 (1H, m, H5'), 1.38–1.25 (1H, m, H4'); ^{13}C NMR (126 MHz, MeOD) δ_C 155.6 (C=O), 136.8 (ArC), 128.2 (ArCH), 127.7 (ArCH), 127.5 (ArCH), 67.0 (CbzCH_2), 50.7 (C2), 48.3 (C3), 43.7 (C6), 32.3 (C4), 23.4 (C5); HRMS $C_{13}H_{19}N_2O_2$ $[M + H]^+$ requires 235.1441, found 235.1428

Benzyl 3-aminoazepane-1-carboxylate (**7c**)

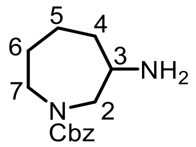

(**7c**) was synthesised using general method D using benzyl 2,3,4,5-tetrahydro-1H-azepine-1-carboxylate (58.0 mg, 0.25 mmol), ammonia (7M solution in methanol, 1 mL), TRIP thiol (27.0 mg, 0.125 mmol) and catalyst B (5.0 mg, 2 mol%). The reaction was irradiated for 16 hours followed by purification by strong cation exchange chromatography (1g benzene sulfonic acid bonded resin H^+ form pre equilibrated with 3x column volumes of methanol. Sample loaded in methanol (ca 2ml) and washed with 10x column volumes of methanol. Compound was eluted with 10x column volumes of methanolic ammonia (7M)) and isolated as a colourless oil (38mg, 62%) as a mixture of rotamers.

^{1}H NMR (400 MHz, Methanol-d_4) δ_H 7.41–7.24 (5H, m, Cbz), 5.23-5.14 (2H, m, CbzCH_2), 3.86–3.70 (1H, m, H2), 3.71–3.51 (1H, m, H7), 3.41–3.18 (1H, m, H7'), 3.10–2.90 (2H, m, H3+H2'), 1.94–1.70 (3H, m, H6+H5+H4), 1.70–1.49 (1H, m, H6'), 1.44–1.21 (2H, m, H5'+H4'); ^{13}C NMR (101 MHz, MeOD) δ_C 156.4 (C=O), 136.8 (ArC), 128.2 (ArCH), 128.2 (ArCH), 127.8 (ArCH), 127.7 (ArCH), 127.6 (ArCH), 127.5 (ArCH), 66.9 (CbzCH_2), 53.1 (C2), 52.9 (C2), 51.1 (C3), 51.0 (C3), 48.5 (C7), 48.2 (C7), 36.1 (C4), 35.6 (C4), 27.5 (C6) , 27.1 (C6), 22.1 (C5), 21.9 (C5): 21 signals observed; HRMS $C_{14}H_{21}N_2O_2$ $[M + H]^+$ requires 249.1598; found 249.1592.

5-Amino-1-benzylpiperidin-2-one (**7d**)

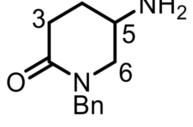

(**7d**) was synthesised using general method D using 1-benzyl-3,4-dihydropyridin-2(1H)-one (47.0 mg, 0.25 mmol), ammonia (7M solution in methanol, 1 mL), TRIP thiol (27.0 mg, 0.125 mmol) and catalyst B (5.0 mg, 2 mol%). The reaction was irradiated for 16 hours followed by purification by strong cation exchange chromatography (1g benzene sulfonic acid bonded resin H^+ form pre equilibrated with 3x column volumes of methanol. Sample loaded in methanol (ca 2ml) and washed with 10x column volumes of methanol. Compound was eluted with 10x column volumes of methanolic ammonia (7M)) and isolated as a colourless oil (34.0 mg, 66%) as a mixture of rotamers.

^{1}H NMR (501 MHz, Chloroform-*d*) δ_H 7.44–6.86 (5H, m, Bn), 4.62 (1H, d, *J* 14.6, $BnCH_2$), 4.51 (1H, d, *J* 14.6, $BnCH_2$), 3.28 (1H, ddd, *J* 11.8, 4.9, 1.7, H6), 3.17 (1H, dddd, *J* 9.8, 8.3, 4.9, 3.4, H5), 2.91 (1H, dd, *J* 11.8, 8.3, H6'), 2.60 (1H, ddd, *J* 17.9, 6.1, 4.6, H3), 2.47 (1H, ddd, *J* 18.0, 10.1, 6.5, H3'), 1.96 (1H, ddddd, *J* 13.0, 6.5, 4.9, 3.5, 1.8, H4), 1.65 (1H, dtd, *J* 13.1, 9.9, 6.1, H4'); ^{13}C NMR (126 MHz, $CDCl_3$) δ_C 169.3 (C2), 137.1 (ArC), 128.7 (ArCH), 128.3 (ArCH), 127.6 (ArCH), 54.8 (C6), 50.2 ($BnCH_2$), 46.0 (C5), 30.7 (C4), 30.1 (C3); HRMS $C_{12}H_{17}N_2O$ $[M + H]^+$ requires 205.1335, found 205.1332.

Benzyl 4-amino-2,2-dimethylpyrrolidine-1-carboxylate (**7e**)

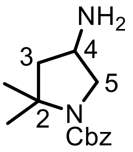

(**7e**) was synthesised using general method D using benzyl 2,2-dimethyl-2,3-dihydro-1H-pyrrole-1-carboxylate (61mg, 0.26mmol), ammonia (1mL 7M in methanol), TRIP thiol (29mg 0.125mmol) and catalyst A (5mg, 2mol%). The reaction was irradiated for 16 hours followed by purification by strong cation exchange chromatography (1g benzene sulfonic acid bonded resin H^+ form pre equilibrated with 3x column volumes of methanol. Sample loaded in methanol (ca 2ml) and washed with 10x column volumes of methanol. Compound was eluted with 10x column volumes of methanolic ammonia (7M)) and isolated as a colourless oil (36mg, 56%) as a mixture of rotamers.

^{1}H NMR (501 MHz, Methanol-*d*$_4$) δ_H 7.42–7.22 (5H, m, Cbz), 5.12 (0.75H, s, $CbzCH_2$), 5.05 (1.25H, s $CbzCH_2$), 3.88–3.71 (1H, m, H5), 3.54–3.40 (1H, m, H4), 3.10–3.04 (1H, m, H5'), 2.13–1.99 (1H, m, H3), 1.80–1.61 (1H, m, H3'), 1.50 (2H, s Me), 1.42 (1H, s, Me), 1.36 (2H, s Me'), 1.31 (1H, s, Me'); ^{13}C NMR (126 MHz, MeOD) δ_C 156.6 (C=O), 155.3 (C=O), 138.4 (ArC), 138.0 (ArC), 129.5 (ArCH), 129.3 (ArCH), 129.2 (ArCH), 129.0 (ArCH), 128.7 (ArCH), 68.1 ($CbzCH_2$), 67.3 ($CbzCH_2$), 62.1 (C5), 61.7 (C5), 56.7 (C2), 55.9 (C2), 51.9 (C4), 51.1

(C4), 48.5 (C3), 48.0 (C3), 28.8 (Me), 27.7 (Me), 27.3 (Me), 26.2 (Me); 23 signals observed; HRMS $C_{14}H_{21}N_2O_2$ $[M + H]^+$ requires 249.1598, found 249.15981

Benzyl 4-amino-3-azaspiro[4.5]decane-1-carboxylate (**7f**)

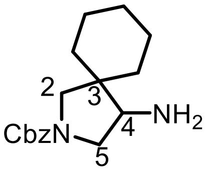

(**7f**) was synthesised using general method D using benzyl 2-azaspiro[4.5]dec-3-ene-2-carboxylate (68.0 mg, 0.25 mmol), ammonia (7M solution in methanol, 1 mL), TRIP thiol (27.0 mg, 0.125 mmol) and catalyst A (5.0 mg, 2 mol%). The reaction was irradiated for 16 hours followed by purification by strong cation exchange chromatography (1g benzene sulfonic acid bonded resin H^+ form pre equilibrated with 3x column volumes of methanol. Sample loaded in methanol (ca 2ml) and washed with 10x column volumes of methanol. Compound was eluted with 10x column volumes of methanolic ammonia (7M)) and isolated as a colourless oil (42 mg, 63%) as a mixture of rotamers.

^1H NMR (501 MHz, Chloroform-*d*) δ_H 7.39–7.28 (5H, m, Cbz), 5.16–5.11 (2H, m, $CbzCH_2$), 3.79–3.65 (1H, m, H5), 3.52 (0.5H, d, *J* 11.0, H2), 3.45 (0.5H, d, *J* 10.8, H2), 3.17 (0.5H, d, *J* 14.4, H2’), 3.15 (0.5H, d, *J* 14.4, H2’), 3.12 (1H, dd, *J* 11.2, 5.9, H5’), 3.08–3.02 (1H, m, H4), 1.66 – 1.55 (3H, m, cyclohexyl), 1.44–1.28 (9H, m, cyclohexyl+NH_2); ^{13}C NMR (126 MHz, $CDCl_3$) δ_C 155.3 (C=O), 155.2 (C=O), 137.2 (ArC), 137.1 (ArC), 128.6 (ArCH), 128.6 (ArCH), 128.0 (ArCH), 128.0 (ArCH), 127.9 (ArCH), 66.9 ($CbzCH_2$), 66.8 ($CbzCH_2$), 59.0 (C3), 58.0 (C3), 53.8 (C1), 53.6 (C1), 52.8 (C4), 52.2 (C4), 44.9 (C3), 44.0 (C3), 34.5 (cyclohexyl), 34.4 (cyclohexyl), 27.8 (cyclohexyl), 27.7 (cyclohexyl), 26.24 (cyclohexyl), 26.21 (cyclohexyl), 23.4 (cyclohexyl), 23.3 (cyclohexyl), 22.92 (cyclohexyl), 22.88 (cyclohexyl); 29 signals observed; HRMS $C_{17}H_{25}N_2O_2$ $[M + H]^+$ requires 289.1911; find 289.1907

Benzyl 3-amino-4-methylpiperidine-1-carboxylate (**7g**)

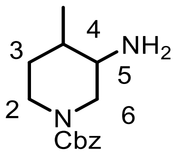

(**7g**) was synthesised using general method D using benzyl 4-methyl-3,4-dihydropyridine-1(2H)-carboxylate (59.0 mg, 0.25 mmol), ammonia (7M solution in methanol, 1mL), TRIP

thiol (29.0 mg, 0.125 mmol) and catalyst A (5.0 mg, 2 mol%). The reaction was irradiated for 16 hours followed by purification by strong cation exchange chromatography (1g benzene sulfonic acid bonded resin H^+ form pre equilibrated with 3x column volumes of methanol. Sample loaded in methanol (ca 2ml) and washed with 10x column volumes of methanol. Compound was eluted with 10x column volumes of methanolic ammonia (7M)) and isolated as a colourless oil (30mg, 48%) as a 1:1 mixture of diastereomers which presented as rotamers.

^{1}H NMR (501 MHz, Methanol-d_4) δ_H 7.40–7.25 (5 H, m, Cbz), 5.11 (2 H, s, $CbzCH_2$), 4.17 (0.5 H, dd, *J* 12.9, 4.6, H6), 4.07 (0.5 H, ddt, *J* 13.3, 4.4, H6), 3.95–3.84 (1 H, m, H2+H2), 3.26–3.13 (0.5 H, m, H2'), 3.01 (0.5 H, br s, H2'), 2.95–2.69 (1 H, m, H5+H6), 2.53 (0.5 H, br s, H6'), 2.28 (1 H, br s, H5), 1.83 (0.5 H, br s, H4), 1.71–1.65 (0.5H, m, H3), 1.58–1.41 (1 H, m, H3), 1.42–1.23 (1 H, m, H3+H4), 1.26–1.11 (0.5H, m, H3), 1.03 (1.3 H, d, *J* 6.5 Me), 0.97 (1.3 H, d, *J* 7.0, Me), 0.93 (0.2 H, d, *J* 6.5, Me), 0.89 (0.2 H, d, *J* 6.7 Me); ^{13}C NMR (126 MHz, MeOD) δ 157.6 (C=O), 156.8 (C=O), 138.14(ArC), 138.12 (ArC), 129.54 (ArCH), 129.53 (ArCH), 129.12 (ArCH), 129.11 (ArCH), 129.0 (ArCH), 128.91 (ArCH), 68.35 ($CbzCH_2$), 68.32 ($CbzCH_2$), 55.0 (C5), 51.3 (C5), 51.2 (C6), 50.3 (C2), 45.1 (C6), 44.1 (C2), 39.9 (C4), 34.8 (C4), 33.8 (C3), 29.0 (C3), 22.4 (Me), 20.3 (Me), 18.5 (Me), 16.8 (Me); 26 signals observed; HRMS $C_{14}H_{21}N_2O_2$ $[M + H]^+$ requires 249.1598, found 249.1593.

Benzyl 3-amino-5-(*tert*butyl)azepane-1-carboxylate (**7h**)

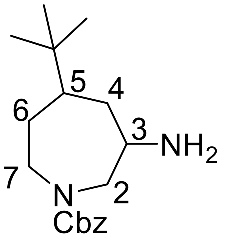

(**7h**) was synthesised using general method D using benzyl 4-(tert-butyl)-2,3,4,5-tetrahydro-1H-azepine-1-carboxylate (75mg, 0.26mmol), ammonia (1mL 7M in methanol), TRIP thiol (29mg 0.125mmol) and catalyst A (5mg, 2mol%). The reaction was irradiated for 16 hours followed by purification by strong cation exchange chromatography (1g benzene sulfonic acid bonded resin H^+ form pre equilibrated with 3x column volumes of methanol. Sample loaded in methanol (ca 2ml) and washed with 10x column volumes of methanol. Compound was eluted with 10x column volumes of methanolic ammonia (7M)) and isolated as a colourless oil (31.0mg, 39%) as a mixture of rotamers.

^{1}H NMR δ $_H$ (501 MHz, Methanol-d_4) δ $_H$ 7.51 – 7.24 (5H, m), 5.26 – 5.04 (2H, m), 3.96 – 3.85 (0.6H, m), 3.85 – 3.75 (0.4H, m), 3.72 – 3.55 (1H, m), 3.41 – 3.34 (1H, m), 3.29 – 3.15 (0.5H, m), 3.15 – 3.01 (0.5H, m), 2.97 – 2.87 (0.5H, m), 2.87 – 2.75 (0.5H, m), 2.10 – 1.76 (2H, m), 1.45 – 1.25 (2H, m), 1.20 – 1.05 (1H, m), 0.97 – 0.80 (9H, m); 13C NMR (126 MHz, MeOD) δ$_C$ 13C NMR (126 MHz, MeOD) δ 158.0 (C=O), 157.6 (C=O), 138.3 (ArC), 129.6 (ArCH), 129.2 (ArCH), 129.1 (ArCH), 129.0 (ArCH), 68.30 (CbzCH_2), 68.26 (CbzCH_2), 68.21 (CbzCH_2), 55.3 (CH_2), 54.9 (CH_2), 53.6 (CH_2), 53.5 (CH_2), 53.1 (C3), 52.8 (C3), 49.0 (C3), 48.9 (C3) 47.9 (CH_2), 47.7 (CH_2), 46.0 (C5), 45.9 (C5), 42.6 (C5), 42.5 (C5), 39.3 (CH_2), 38.7 (CH_2), 37.9 (CH_2), 37.4 (CH_2), 34.3 (C^tBu), 34.1 (C^tBu), 31.5 (CH_2), 31.4 (CH_2), 29.9 (CH_2), 29.3 (CH_2), 27.8 (CH_3), 27.7 (CH_3), 27.6 (CH_3); 37 signals observed; HRMS C18H29N2O2 [M + H]+ requires 305.2224, found 305.2231

Benzyl 5-amino-2-methylpiperidine-1-carboxylate (**7i**)

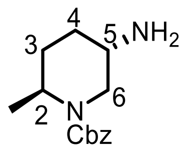

(**7h**) was synthesised using general method D using benzyl 2-methyl-3,4-dihydropyridine-1(2H)-carboxylate (58.0 mg, 0.25 mmol), ammonia (7M solution in methanol, 1mL), TRIP thiol (29.0 mg, 0.125 mmol) and catalyst A (5.0 mg, 2 mol%). The reaction was irradiated for 16 hours followed by purification by strong cation exchange chromatography (1g benzene sulfonic acid bonded resin H^+ form pre equilibrated with 3x column volumes of methanol. Sample loaded in methanol (ca 2ml) and washed with 10x column volumes of methanol. Compound was eluted with 10x column volumes of methanolic ammonia (7M)) and isolated as a colourless oil (37.0 mg, 60%) as an inseparable 1:2.7 mixture of diastereomers, which presented as rotamers. Major diastereomer was assigned through large vicinal coupling between H6 axial and H5.

Signals for minor diastereomer in italics and underlined

^{1}H NMR (400 MHz, Methanol-d_4) δ $_H$ 7.36–7.24 (5H, m, Cbz), 5.12 (2H, app d, *J* 4.4, CbzCH_2), 4.48–4.35 (1H, m, H2+*H2*), 4.15–4.00 (0.25H, m, *H6*), 3.92–3.83 (0.75H, m, H6), 3.19 (0.75H, dd, *J* 13.9, 2.7, H6'), 3.09–2.99 (0.75H, m, H5), 2.69–2.52 (0.5H, m, *H5*+*H6'*), 2.10–1.84 (1.5H, m, H3+H4), 1.80–1.56 (0.75H, m, *H3*+*H3'*+*H4*), 1.55–1.39 (1H, m H4'+*H4'*), 1.39–1.25 (0.75H, m, H3'), 1.17 (2.25H, d, *J* 6.9, Me), 1.16 (0.75H, d, *J* 7.1 *Me*); ^{13}C NMR (101 MHz, MeOD) δ$_C$ 157.9 (C=O), 156.9 (*C=O*), 138.2 (ArC), 129.5 (ArCH), 129.1 (ArCH), 128.89 (ArCH), 128.85 (ArCH), 68.3 (CbzCH_2), 49.3 (*C5*), 48.0 (C2), 47.1 (*C2*), 46.8

(*<u>C6</u>*), 46.2 (C5), 45.5 (C5), 30.0 (*<u>C3)</u>*, 28.9 (*<u>C4</u>*), 25.9 (C4), 24.7 (C3), 15.9 (Me); 19 signals observed; HRMS $C_{14}H_{21}N_2O_2$ [M + H]$^+$ requires 249.1598, found 249.1593

Benzyl 3a-aminooctahydro-2*H*-isoindole-2-carboxylate (**7j**)

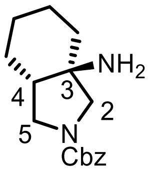

(**7j**) was synthesised using general method D using Benzyl hexahydro-2*H*-isoindole-1-carboxylate (32.0 mg, 0.125 mmol), ammonia (7M solution in methanol, 1mL), TRIP thiol (14.0 mg, 0.125 mmol) and catalyst A (2.5 mg, 2 mol%). The reaction was irradiated for 16 hours followed by purification by strong cation exchange chromatography (1g benzene sulfonic acid bonded resin H$^+$ form pre equilibrated with 3x column volumes of methanol. Sample loaded in methanol (ca 2ml) and washed with 10x column volumes of methanol. Compound was eluted with 10x column volumes of methanolic ammonia (7M)) and isolated as a colourless oil (19.0 mg, 54%).

^{1}H NMR (501 MHz, Chloroform-*d*) δ_H 7.44–7.29 (5H, m, Cbz), 5.21–5.07 (2H, m, $CbzCH_2$), 3.66 (0.5H, ddd, *J* 10.8, 6.9, H1), 3.64 (0.5H, dd *J* 10.8 6.9, H5) 3.55 (0.5 H, d, *J* 10.9, H2), 3.50 (0.5H, d, *J* 10.8, H2), 3.34 (0.5H, dd, *J* 10.9, 5.0, H5’), 3.29 (0.5H, dd, *J* 10.7, 4.8 H5’), 3.13 (0.5H, d, *J* 11.3, H2’), 3.13 (0.5H, d, *J* 11.3, H2’), 1.90–1.80 (1 H, m H4), 1.80–1.38 (8H, m, $4xCH_2$); ^{13}C NMR (126 MHz, $CDCl_3$) δ_C 155.5 (C=O), 137.0 (ArC), 137.0 (ArC), 128.5 (ArCH), 127.9 (ArCH), 127.9 (ArCH), 127.9 (ArCH), 66.8 ($CbzCH_2$), 57.5 (C3), 56.9 (C2), 56.72 (C3), 56.69 (C2), 50.3 (C5), 50.1 (C5), 45.1 (C4), 44.6 (C4), 34.6 (CH_2), 34.4 (CH_2), 26.6 (CH_2), 26.4 (CH_2), 23.1 (CH_2), 23.0 (CH_2), 22.7 (CH_2), 22.6 (CH_2); 24 signals observed; HRMS $C_{16}H_{23}N_2O_2$ [M + H]$^+$ requires 275.1754, found 275.1749.

Benzyl 3-((*tert*-butoxycarbonyl)amino)pyrrolidine-1-carboxylate (**8**)

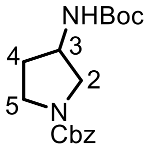

(**8**) was synthesised using general method E using benzyl 2,3-dihydro-1H-pyrrole-1-carboxylate (52.0 mg, 0.25 mmol), ammonia (7M solution in methanol, 1 mL), TRIP thiol (27.0 mg, 0.125 mmol) and catalyst A (5.0 mg, 2 mol%). The reaction was irradiated for 16 hours. Following removal of solvent *in vacuo*, di-*tert*-butyl dicarbonate (110 mg, 0.50 mmol),

DIPEA (87 µL, 0.50 mmol) and DCM (2 mL) were added. The solution was stirred for 16 hours followed by purification by column chromatography (3:1 to 2:1 hexane/ethyl acetate) and isolated as a colourless oil (56.0 mg, 66%) as a mixture of rotamers.

^{1}H NMR (501 MHz, Chloroform-*d*) δ $_H$ 7.39–7.28 (5H, m, Cbz), 5.13 (2H, s, $CbzCH_2$), 4.62 (1H, br s, NH), 4.21 (1H, br s, H3), 3.71–3.61 (1H, m, H2), 3.56–3.42 (2H, m, H5), 3.33–3.19 (1H, m, H2'), 2.19–2.05 (1H, m, H4), 1.92–1.73 (1H, m, H4'), 1.44 (9H, s, Boc); ^{13}C NMR (126 MHz, $CDCl_3$) δ$_C$ 155.3 (C=O), 155.04 (C=O), 155.00 (C=O), 136.9 (ArC), 128.6 (ArCH), 128.15 (ArCH), 128.11 (ArCH), 80.0 (Boc q), 67.0 ($CbzCH_2$), 52.1 (C2), 51.9 (C2), 50.6 (C3), 49.9 (C3), 44.4 (C5), 44.0 (C5), 32.2 (C4), 31.3 (C4), 28.5 (Boc): 18 signals observed; HRMS $C_{17}H_{25}N_2O_4$ [M + H]$^+$ requires 343.1628, found 343.1644.

Benzyl 3-(furan-2-carboxamido)pyrrolidine-1-carboxylate (**9**)

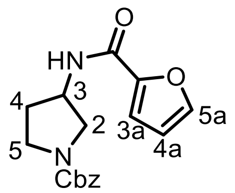

(**9**) was synthesised using general method E using benzyl 2,3-dihydro-1H-pyrrole-1-carboxylate (52.0 mg, 0.25 mmol), ammonia (7M solution in methanol, 1 mL), TRIP thiol (27.0 mg 0.125 mmol) and catalyst A (5.0 mg, 2 mol%). The reaction was irradiated for 16 hours. Following removal of solvent *in vacuo*, 2 furoyl chloride (50 µL, 0.51 mmol), DIPEA (87 µL, 0.50 mmol) and DCM (2 mL) were added. The solution was stirred for 16 hours followed by purification by column chromatography (2:1 hexane/ethyl acetate) and isolated as colourless oil (40.0 mg, 51%).

^{1}H NMR (501 MHz, Chloroform-*d*) δ $_H$ 7.45–7.42 (1H, m, H5a), 7.36-7.28 (5H, m, Cbz), 7.12 (1H, d, *J* 3.5, H3a), 6.50 (1H, dd, *J* 3.5, 1.8, H4a), 6.40 (1H, d, *J* 7.4, NH), 5.19–5.08 (2H, m, $CbzCH_2$), 4.72–4.58 (1H, m, H3), 3.77 (1H, dd, *J* 11.6, 6.3, H2), 3.65-3.58 (2H, m, H5), 3.42 (0.5H, dd, *J* 11.8, 4.4, H2'), 3.39 (0.5H, dd, *J* 11.8, 4.4, H2'), 2.31–2.19 (1H, m, H4), 2.06–1.87 (1H, m, H4'); ^{13}C NMR (126 MHz, $CDCl_3$) δ$_C$ 158.3 (C=O), 155.0 (C=O), 147.7 (ArC), 144.1 (C5a), 136.8 (ArC), 128.6 (ArCH), 128.2 (ArCH), 128.1 (ArCH), 114.8 (C3a), 112.4 (C4a), 67.1 ($CbzCH_2$), 51.8 (C3), 51.7 (C3), 49.3 (C2), 48.6 (C2), 44.4 (C5), 44.1 (C5), 32.1 (C4), 31.2 (C4): 19 signals observed; HRMS : $C_{17}H_{18}N_2NaO_4$ [M + Na]$^+$ requires 337.1164, found 337.1173.

Benzyl 3-(3-(3-fluorophenyl)ureido)pyrrolidine-1-carboxylate (**10**)

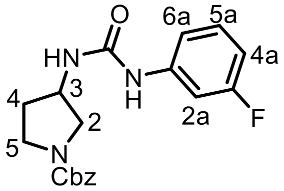

(**10**) was synthesised using general method E using benzyl 2,3-dihydro-1H-pyrrole-1-carboxylate (56mg, 0.28mmol) ammonia (7M solution in methanol, 1 mL) TRIP thiol (27mg 0.125mmol) and catalyst A (5mg, 2mol%). The reaction was irradiated for 16 hours. Following removal of solvent invacuo, 3 fluorophenyl isocyanate (57 µL, 0.5mmol), DIPEA (87 µL,0.5mmol) and DCM (2mL). The solution was stirred for 4 hours followed by purification by column chromatography (1:2 to 0:1 hexane ethyl acetate) and isolated as an off white solid (38mg 41%).

^{1}H NMR (501 MHz Methanol-d_4) δ_H 7.39–7.27 (6H, m, Cbz + H2a), 7.26–7.17 (1H, m, H5a), 6.99 (1H, dd, *J* 8.2, 1.1, H6a), 6.68 (1H, td, *J* 8.4, 2.5, H4a), 5.13 (2H, s, CbzCH$_2$), 4.33–4.25 (1H, m, H3), 3.71–3.62 (1H, m, H2), 3.56–3.44 (2H, m, H5), 3.37–3.27 (1H, m, H2'), 2.30–2.07 (1H, m, H4), 1.97–1.82 (1H, m, H4'); ^{13}C NMR (126 MHz, MeOD) δ_C 164.1 (C=O), 155.9 (C=O), 155.4 (C=O), 141.4 (ArC), 136.8 (ArC), 129.7 (C2a), 129.6 (C2a), 128.1 (ArCH), 127.7 (ArCH), 127.6 (ArCH), 127.5 (ArCH), 113.8 (C6a), 113.7 (C6a), 108.3 (C4a), 108.1 (C4a), 105.4 (C5a), 105.2 (C5a), 66.7 (CbzCH$_2$), 51.6 (C2), 51.5 (C2), 49.8 (C3), 49.0 (C3), 44.0 (C5), 43.7 (C5), 31.3 (C4), 30.4 (C4): 26 signals observed; HRMS $C_{19}H_{20}FN_3NaO_3$ [M + Na]$^+$ requires 380.1386, found 380.1392.

Benzyl 3-(pyridine-3-sulfonamido)pyrrolidine-1-carboxylate (**11**)

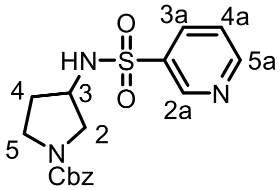

(**11**) was synthesised using general method E using benzyl 2,3-dihydro-1H-pyrrole-1-carboxylate (57.0 mg, 0.27 mmol) ammonia (7M solution in methanol, 1 mL), TRIP thiol (27.0 mg, 0.125 mmol) and catalyst A (5.0 mg, 2 mol%). The reaction was irradiated for 16 hours. Following removal of solvent *in vacuo*, pyridine-3-sulfonyl chloride (60 µL, 0.50 mmol), DIPEA (87 µL,0.50 mmol) and DCM (2 mL) were added. The solution was stirred for 16 hours followed by purification by column chromatography (1:1 hexane/ethyl acetate) and isolated as colourless oil (53mg, 59%) as a mixture of rotamers.

^{1}H NMR (501 MHz, Chloroform-*d*) δ_H 9.08 (1H, d, *J* 2.4, H2a), 8.81 (1H, s, H5a), 8.14 (1H, d, *J* 8.1, H5a), 7.50–7.42 (1H, m, H3a), 7.38-7.30 (5H, m, Cbz), 5.44 (1H, d, *J* 7.3, NH), 5.09 (2H, s, $CbzCH_2$), 3.96–3.85 (1H, m, H3), 3.60–3.50 (1H, m, H2), 3.52–3.43 (1H, m, H5), 3.45–3.37 (1H, m, H5'), 3.29–3.19 (1H, m, H2'), 2.13–1.98 (1H, m, H4), 1.97–1.81 (0.5H, m, H2'), 1.78-1.69 (0.5H, m, H2'); ^{13}C NMR (126 MHz, $CDCl_3$) δ_C 154.9 (C=O), 153.6 (C2a), 148.1 (C5a), 137.3 (ArC), 136.6 (ArC), 134.8 (C4a), 128.7 (ArCH), 128.3 (ArCH), 128.14 (ArCH), 127.98 (ArCH), 124.0 (C3a), 67.3 ($CbzCH_2$), 53.2 (C3), 52.5 (C3), 51.8 (C2), 44.0 (C5), 43.7 (C5), 32.6 (C4), 31.7 (C4); 19 signals observed; HRMS $C_{17}H_{19}N_3NaO_4S$ $[M + Na]^+$ requires 384.0994, found 384.1006.

Aminations with (hetero)aryl amines and N-H heterocycles (Scheme 5)

Benzyl 3-(phenylamino)pyrrolidine-1-carboxylate (**12a**)

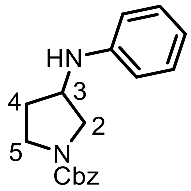

(**12a**) was synthesised using general method C using benzyl 2,3-dihydro-1H-pyrrole-1-carboxylate (51.0 mg, 0.25 mmol), aniline (47.0 mg, 0.50 mmol), TRIP thiol (27.0 mg, 0.125 mmol) and catalyst A (5.0 mg, 2 mol%). The reaction was irradiated for 40 hours followed by purification by column chromatography (2:1 to 1:1 hexane/ethyl acetate) and isolated as a colourless oil (43mg, 58%) as a mixture of rotamers.

^{1}H NMR (501 MHz, Chloroform-*d*) δ $_H$ 7.32–7.23 (5H, m, Cbz), 7.12 (2H, t, *J* 7.9, 2 x ArH), 6.67 (1H, t, *J* 7.3, ArH), 6.59–6.49 (2H, m, 2 x ArH), 5.08 (2H, s, CbzCH$_2$), 4.11–3.88 (1H, m, H3), 3.71 (1H, app ddd, *J* 19.8, 11.2, 5.7, H2+H2), 3.57–3.39 (2 H, m, H5), 3.30 (0.5H, dd, *J* 11.3, 4.0, H1'), 3.25 (0.5 H, dd, 11.3, 4.0, H1'), 2.14 (1H, td, *J* 13.2, 7.8, H4), 1.96–1.73 (1H, m, H4); ^{13}C NMR (126 MHz, CDCl$_3$) δ$_C$ 155.0 (C=O), 146.7 (ArC), 129.4 (ArCH), 128.5 (ArCH), 128.0 (ArCH), 128.0 (ArCH), 118.1 (ArCH), 113.3 (ArCH), 66.9 (CbzCH$_2$), 53.4 (C3), 52.4 (C2), 52.3 (C3), 51.9 (C2), 44.4 (C5), 44.0 (C5), 31.9 (C4), 31.2 (C4); 17 signals observed; HRMS $C_{18}H_{21}N_2O_2$ [M + H]$^+$ requires 297.1598, found 297.1592.

Benzyl 3-((3-(trifluoromethyl)phenyl)amino)pyrrolidine-1-carboxylate (**12b**)

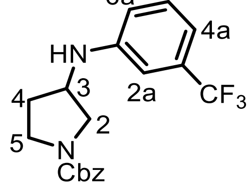

(**12b**) was synthesised using general method C using benzyl 2,3-dihydro-1H-pyrrole-1-carboxylate (52.0 mg, 0.26 mmol), 3-(trifluoromethyl)aniline (80.0 mg, 0.50 mmol), TRIP thiol (27.0 mg, 0.125 mmol) and catalyst A (5.0 mg, 2 mol%). The reaction was irradiated for 40 hours followed by purification by column chromatography (2:1 to 1:1 hexane/ethyl acetate) and isolated as a colourless oil (49.0 mg, 54%).

^{1}H NMR (400 MHz, Chloroform-*d*) δ $_H$ 7.42–7.29 (5H, m, Cbz), 7.27 (1H, t, *J* 7.9, H5a), 6.97 (1H, d, *J* 7.7, H4a), 6.79 (1H, d, *J* 2.0, H2a), 6.73 (1H, d, *J* 6.0, H6a), 5.27–4.99 (2H, m,

CbzCH$_2$), 4.07 (1 H, br s, H3), 3.97 (1H, s, NH), 3.85–3.69 (1H, m, H2), 3.62–3.51 (2H, m, H5+H5'), 3.39 (0.5H, dd, J 11.8, 3.6, H2'), 3.33 (0.5H, dd, J 11.3, 4.0, H2'), 2.30–2.13 (1H, m, H4), 2.03–1.82 (1H, m, H4'); ^{13}C NMR (101 MHz, CDCl$_3$) δ 155.1 (C=O), 147.0 (ArC), 130.0 (C5a), 128.6 (ArCH), 128.2 (ArCH), 128.1 (ArCH), 116.2 (C6a), 114.5 (C4a), 109.4 (C2a), 67.1 (CbzCH$_2$), 53.0 (C3), 52.4 (C3), 52.1 (C2), 51.9 (C2), 44.4 (C5), 44.1 (C5), 31.8 (C4), 31.1 (C4): 18 signals observed CF$_3$ quartet is not observed; HRMS; $C_{19}H_{20}F_3N_2O_2$ [M + H]$^+$requires 365.1471, found 365.1470.

Benzyl 3-(pyridin-2-ylamino) pyrrolidine-1-carboxylate (**12c**)

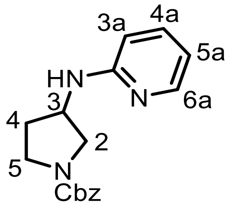

(**12c**) was synthesised using general method C using benzyl 2,3-dihydro-1H-pyrrole-1-carboxylate (50.0 mg, 0.25 mmol), 2-aminopyridine (47.0 mg, 0.50 mmol), TRIP thiol (27.0 mg, 0.125 mmol) and catalyst A (5.0 mg, 2 mol%). The reaction was irradiated for 40 hours followed by purification by column chromatography (2:1 to 1:1 hexane/ethyl acetate) and isolated as a colourless oil (44mg, 59%) as a mixture of rotamers.

^{1}H NMR (501 MHz, Chloroform-*d*) δ_H 8.09 (1H, d, *J* 3.6, H6a), 7.42 (1H, t, *J* 7.7, H4a), 7.39–7.27 (5H, m, Cbz), 6.60 (1H, dd, *J* 7.0, 5.1, H5a), 6.42–6.36 (1H, m, H3a), 5.19–5.07 (2H, m, CbzCH$_2$), 4.64–4.49 (1H, m, NH), 4.45–4.32 (1H, m, H3), 3.80 (1H, dd, *J* 11.2, 6.0, H2), 3.63–3.50 (2H, m H5), 3.38 (0.5H, dd, *J* 11.3, 4.0 H2'), 3.30 (0.5H, dd, *J* 11.1, 4.4 *H2'*), 2.31–2.19 (1H, m, H4), 1.99–1.86 (1H, m, H4'); ^{13}C NMR (126 MHz, CDCl$_3$) δ_C 157.7 (C=O), 155.0 (C2a), 148.2 (C6a), 137.6 (C4a), 136.9 (ArC), 128.6 (ArCH), 128.1 (ArCH), 128.0 (ArCH), 113.5 (C5a), 107.7 (C3a), 107.6 (C3a), 66.9 (CbzCH$_2$), 52.3 (C2), 52.1 (C2), 51.5 (C3), 50.8 (C3), 44.5 (C5), 44.1 (C5), 32.1 (C4), 31.4 (C4); 20 signals observed; HRMS $C_{17}H_{19}N_3O_2Na$ [M + Na]$^+$ requires 320.1375, found 320.1374.

Benzyl 3-(pyrimidin-2-ylamino)pyrrolidine-1-carboxylate (**12d**)

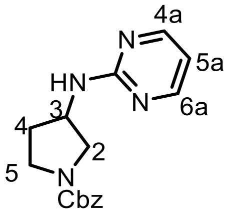

(**12d**) was synthesised using general method C using benzyl 2,3-dihydro-1H-pyrrole-1-carboxylate (50.0 mg, 0.25 mmol), 2-aminopyrimidine (47.0 mg, 0.50 mmol), TRIP thiol (27.0 mg, 0.125 mmol) and catalyst A (5.0 mg, 2 mol%). The reaction was irradiated for 40 hours followed by purification by column chromatography (2:1 to 1:1 hexane/ethyl acetate) and isolated as a colourless oil (22.0 mg, 29%) as a mixture of rotamers.

^{1}H NMR (501 MHz, Chloroform-*d*) δ_H 8.28 (2H, d, *J* 4.8, H4a+H6a), 7.40–7.29 (5H, m, Cbz), 6.57 (1H, t, *J* 4.8, H5a), 5.21 (1H, d, *J* 7.0, NH), 5.15 (2H, s, CbzCH$_2$), 4.53 (1H, m, H3), 3.81 (1H, dd, *J* 11.3, 6.3, H2), 3.65–3.48 (2 H, m, H5), 3.41 (0.5H, dd, *J* 11.3, 4.6, H2'), 3.32 (0.5H, dd, *J* 11.3, 4.6, *H2'*) 2.31–2.21 (1 H, m, H4), 2.00–1.86 (1 H, m, H4); ^{13}C NMR (126 MHz, CDCl$_3$) δ_C 161.9 (2a), 158.2 (C4a+C6a), 155.1 (C=O), 136.9 (ArC), 128.6 (ArCH), 128.1 (ArCH), 128.0 (ArCH), 111.4 (C5a), 66.9 (CbzCH$_2$), 52.2 (C2), 51.9 (C2), 51.3 (C3), 50.5 (C3), 44.6 (C5), 44.2 (C5), 32.0 (C4), 31.3 (C4): 17 signals observerd; HRMS $C_{16}H_{19}N_4O_2$ [M + H]$^+$ requires 299.1503, found 299.1514.

Benzyl 3-((6-methylpyridin-2-yl)amino)pyrrolidine-1-carboxylate (**12e**)

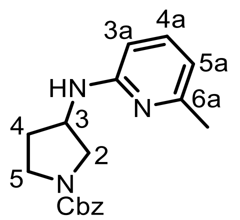

(**12e**) was synthesised using general method C using benzyl 2,3-dihydro-1H-pyrrole-1-carboxylate (49.0 mg, 0.24 mmol), 2-amino-5-methyl pyridine (54.0 mg, 0.50 mmol), TRIP thiol (27.0 mg, 0.125mmol) and catalyst A (5.0 mg, 2 mol%). The reaction was irradiated for 40 hours followed by purification by column chromatography (2:1 to 1:1 hexane/ethyl acetate) and isolated as a colourless oil (44.0 mg, 57%) as a mixture of rotamers.

^{1}H NMR (501 MHz, Chloroform-*d*) δ_H 7.52–7.29 (6H, m, Cbz+H3a), 6.48 (1H, d, *J* 7.3, H4a), 6.19 (1H, dd, *J* 8.2, 6.3, H5a), 5.16–5.08 (2H, m, CbzCH$_2$), 4.55-4.51 (1H, t, *J* 6.5, NH), 4.37-4.25 (1H, m, H3), 3.79 (0.5H, dd, *J*, 7.8, 5.9, H2), 3.76 (0.5H, dd, *J*, 7.8, 5.9, *H2*), 3.66–3.47 (2H, m, H5+H5'), 3.36 (0.5H, dd, *J* 11.3, 4.3, H2), 3.29 (0.5H, dd, *J* 11.1, 4.6, H2'), 2.36 (3H, s, Me), 2.29–2.16 (1H, m, H4), 1.97–1.84 (1H, m, H4'); ^{13}C NMR (126 MHz, CDCl$_3$) δ_C 157.4 (C=O), 157.4 (C=O), 155.1 (ArC), 138.1 (C3a), 138.1 (ArC), 136.9 (ArC), 136.9 (ArC), 128.6 (ArCH), 128.14 (ArCH), 128.11 (ArCH), 128.1 (ArCH), 113.0 (C5a), 113.0 (C5a), 103.4 (C4a), 103.3 (C4a), 67.0 (CbzCH$_2$), 52.3 (C2), 52.0 (C2), 51.7 (C3), 50.9 (C3), 44.5 (C5), 44.1 (C5), 32.0 (C4), 31.3 (C4), 24.54 (Me), 24.51 (Me); 26 signals observed; HRMS $C_{18}H_{22}N_3O_2$ [M + H]$^+$ requires 312.1707, found 312.1728.

Benzyl 2,2-dimethyl-4-(pyridin-2-ylamino)pyrrolidine-1-carboxylate (**12f**)

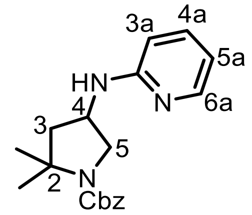

(**12f**) was synthesised using general method C using benzyl 2,2-dimethyl-2,3-dihydro-1H-pyrrole-1-carboxylate (55.0 mg, 0.25 mmol), 2-aminopyridine (54.0 mg, 0.50 mmol), TRIP thiol (27.0 mg, 0.125 mmol) and catalyst A (5.0 mg, 2 mol%). The reaction was irradiated for 40 hours followed by purification by column chromatography (2:1 to 1:1 hexane/ethyl acetate) and isolated as a colourless oil (42.0 mg, 51%) as a mixture of rotamers.

^{1}H NMR (501 MHz, Chloroform-*d*) δ_H 8.08 (1H, ddd, *J* 5.1, 2.0, 0.9, H6a), 7.41 (1H, ddd, *J* 8.7, 7.1, 1.9, H4a), 7.39 – 7.28 (5H, m, Cbz), 6.60 (1H, ddd, *J* 7.1, 5.0, 0.9, H5a), 6.43–6.36 (1 H, m, H3a), 5.25–4.98 (2 H, m, CbzCH$_2$), 4.44 (1 H, br s, NH), 4.40–4.25 (1 H, m, H4), 4.16–4.01 (1 H, m, H5), 3.30 (0.3 H, dd, J 11.3, 7.2, H5’), 3.21 (0.7 H, dd, J 11.1, 7.4, H5’), 2.33–2.21 (1 H, m, H3), 1.92–1.76 (1 H, m, H3’), 1.46 (2 H, s, Me), 1.39 (1 H, s, Me), 1.26 (2 H, d, *J* 6.9, Me’), 1.15 (1 H, d, *J* 6.9, Me’); ^{13}C NMR (126 MHz, CDCl$_3$) δ_C 158.1 (C=O), 153.8 (ArC), 153.4 (ArC), 148.3 (C6a), 146.1 (C6a), 137.6 (C4a), 137.2 (C4a), 137.0 (ArC), 128.6 (ArCH), 128.2 (ArCH), 128.1 (ArCH), 128.0 (ArCH), 113.6 (C5a), 109.2 (C3a), 107.6 (C3a), 67.2 (CbzCH$_2$), 66.4 (CbzCH$_2$), 60.4 (C2), 59.9 (C2), 54.3 (C5), 53.6 (C5), 49.0 (C3), 48.4 (C4), 48.1 (C3), 27.6 (Me), 26.4 (Me), 24.4 (Me), 24.1 (Me): 28 signals observed; HRMS $C_{19}H_{24}N_3O_2$ [M + H]$^+$ requires 326.1863, found 326.1878.

Benzyl 3-(pyridin-2-ylamino)piperidine-1-carboxylate (**12g**)

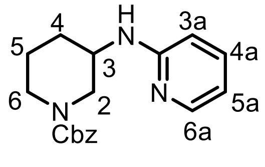

(**12g**) was synthesised using general method C using benzyl 3,4-dihydropyridine-1(2H)-carboxylate (56.0 mg, 0.25 mmol), 2-aminopyridine (54.0 mg, 0.5mmol), TRIP thiol (27.0 mg, 0.125 mmol) and catalyst B (5.0 mg, 2 mol%). The reaction was irradiated for 40 hours followed by purification by column chromatography (2:1 to 1:1 hexane/ethyl acetate) and isolated as a colourless oil (24.0 mg, 31%) as a mixture of rotamers.

^{1}H NMR (501 MHz, Chloroform-*d*) δ_H 8.00 (1H, dd, *J* 5.1, 1.9, H6a), 7.32–7.23 (6H, m, Cbz+H4a), 6.51 (1H, dd, *J* 7.1, 5.1, H5a), 6.41–6.17 (1H, m, H3a), 5.14–5.01 (2H, m,

CbzCH$_2$), 4.48–4.35 (1H, m, NH), 4.11–3.87 (1H, m, H6), 3.71–3.61 (2H, m, H2+H3), 3.26–2.85 (2H, m, H2'+H6'), 1.98-1.95 (1H, m, H4), 1.76–1.56 (3H, m, H5+H5'+H3'); ^{13}C NMR (126 MHz, CDCl$_3$) δ_C 157.5 (C=O), 155.4 (ArC), 148.3 (C6a), 137.6 (ArC), 136.7 (C5a), 128.6 (ArCH), 128.0 (ArCH), 127.8 (ArCH), 113.2 (C6a), 107.0 (C4a), 67.2 (CbzCH$_2$), 49.1 (C6), 47.3 (C3), 44.4 (C2), 30.5 (C4), 29.7 (C4); 16 signals observed; HRMS; $C_{18}H_{22}N_3O_2$ [M + H]$^+$ requires 312.1707; found 312;1722.

Benzyl 3-(1*H*-pyrazol-1-yl)pyrrolidine-1-carboxylate (**13a**)

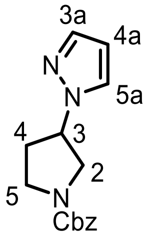

(**13a**) was synthesised using general method C using benzyl 2,3-dihydro-1H-pyrrole-1-carboxylate (53.0 mg, 0.26 mmol), pyrazole (34.0 mg, 0.50 mmol), TRIP thiol (27.0 mg, 0.125mmol) and catalyst A (5.0 mg, 2 mol%). The reaction was irradiated for 16 hours followed by purification by column chromatography (2:1 to 1:1 hexane/ethyl acetate) and isolated as a colourless oil (46.0 mg, 65%) as a mixture of rotamers.

^{1}H NMR (501 MHz, Chloroform-*d*) δ_H 7.46 (1H, d, *J* 1.8, ArCH), 7.36–7.22 (6H, m, Cbz+ ArCH), 6.19 (1H, d, *J* 2.2, ArCH), 5.15–5.01 (2H, m, CbzCH$_2$), 4.91–4.80 (1H, m, H3), 3.85 (1H, dd, *J* 11.6, 7.0, H2), 3.77 (0.5H, dd, J 11.9, 4.6, H2'), 3.72 (0.5H, dd, J 11.5, 5.4, H2'), 3.68–3.49 (2H, m, H5), 2.41–2.24 (2H, m, H4); ^{13}C NMR (126 MHz, CDCl$_3$) δ_C 154.7 (C=O), 139.7 (ArCH), 136.7 (ArC), 128.5 (ArCH), 128.1 (ArCH), 128.0 (ArCH), 127.6 (ArCH), 105.8 (ArCH), 67.0 (CbzCH$_2$), 60.4 (C3), 59.6 (C3), 51.5 (C2), 51.1 (C2), 44.7 (C5), 44.4 (C5), 32.1 (C4), 31.2 (C4); 17 signals observed; HRMS $C_{15}H_{17}N_3NaO_2$ [M + Na]$^+$ requires 294.1218, found 294.1216.

Benzyl 3-(1*H*-imidazol-1-yl)pyrrolidine-1-carboxylate (**13b**)

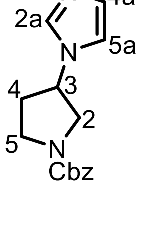

(**13b**) was synthesised using general method C using benzyl 2,3-dihydro-1H-pyrrole-1-carboxylate (51.0 mg, 0.25 mmol), imidazole (35.0 mg, 0.51 mmol), TRIP thiol (27.0 mg, 0.125mmol) and catalyst A (5.0 mg, 2 mol%). The reaction was irradiated for 16 hours followed by purification by column chromatography (2:1 to 1:1 hexane/ethyl acetate) and isolated as a colourless oil (36.0 mg, 51%) as a mixture of rotamers.

^{1}H NMR (501 MHz, Methanol-d_4) δ_H 7.91–7.77 (1H, m, H2a), 7.47–7.03 (7H, m, Cbz+H4a+H5a), 5.15 (2H, s, $CbzCH_2$), 4.93 (1H, t, J 6.7, H3), 3.99–3.72 (1H, m, H2), 3.70–3.52 (3H, m, H5+H5'+H2'), 2.51–2.41 (1H, m, H4), 2.35-2.24 (1H, m, H4'); ^{13}C NMR (126 MHz, MeOD) δ_C 156.5 (C=O), 151.4 (C2a), 149.6 (C2a), 138.1 (ArC), 129.6 (ArCH), 129.2 (ArCH), 129.0 (ArCH), 127.1 (C5a), 123.4 (C4a), 68.3 ($CbzCH_2$), 57.6 (C3), 56.9 (C3), 52.9 (C2), 52.6 (C2), 45.7 (C5), 45.4 (C5), 33.3 (C4), 32.5 (C4); 18 signals observed; HRMS $C_{15}H_{17}N_3O_2Na$ [M + Na]$^+$ requires 294.1213, found 294.1222.

Benzyl 3-(1*H*-1,2,4-triazol-1-yl)pyrrolidine-1-carboxylate (**13c**)

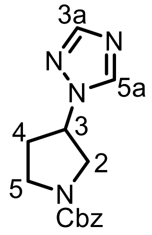

(**13c**) was synthesised using general method C using benzyl 2,3-dihydro-1H-pyrrole-1-carboxylate (53.0 mg, 0.26 mmol), 1,2,4-triazole (69.0 mg, 0.50 mmol), TRIP thiol (27.0 mg, 0.125 mmol) and catalyst A (5.0 mg, 2 mol%). The reaction was irradiated for 16 hours followed by purification by column chromatography (2:1 to 1:1 hexane/ethyl acetate) and isolated as a colourless oil (37.0 mg, 49%) as a mixture of rotamers.

^{1}H NMR (501 MHz, Methanol-d_4) δ_H 8.52 (1H, d, J 3.9, H5a), 7.99 (1H, s, H3a), 7.39–7.27 (5H, m, Cbz), 5.19–5.11 (3H, m, $CbzCH_2$ + H3), 3.95–3.84 (1H, m, H2), 3.84–3.74 (1H, m, H2'), 3.76–3.65 (1H, m, H5), 3.67–3.55 (1H, m, H5'), 2.53–2.34 (2H, m, H4); ^{13}C NMR (126 MHz, MeOD) δ_C 156.5 (C=O), 152.5, (C3a) 144.3 (C5a), 138.1 (ArC), 129.5 (ArCH), 129.1 (ArCH), 128.9 (ArCH), 68.2 ($CbzCH_2$), 60.0 (C3), 59.2 (C3), 52.4 (C2), 52.1 (C2), 45.9 (C5), 45.6 (C5), 32.6 (C4), 31.8 (C4); 16 signals observed; HRMS $C_{14}H_{16}N_4NaO_2$ [M + Na]$^+$ requires 295.1171, found 295.1162.

Benzyl 3-(1*H*-pyrazol-1-yl)piperidine-1-carboxylate (**13d**)

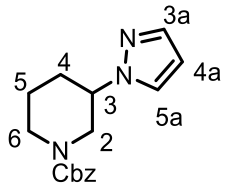

(**13d**) was synthesised using general method C using benzyl 3,4-dihydropyridine-1(2H)-carboxylate (56.0 mg, 0.26 mmol), pyrazole (34.0 mg, 0.51 mmol), TRIP thiol (27.0 mg, 0.125 mmol) and catalyst A (5.0 mg, 2 mol%). The reaction was irradiated for 16 hours followed by purification by column chromatography (2:1 to 1:1 hexane/ethyl acetate) and isolated as a colourless oil (48.0 mg, 65%) as a mixture of rotamers.

^{1}H NMR (500 MHz, Chloroform-*d*) δ_H 7.52 (1H, d, *J* 1.8, H5a), 7.43 (1H, br s, H3a), 7.38 – 7.30 (5H, m, Cbz), 6.24 (1H, t, *J* 2.1, H4a), 5.16-5.08 (2H, m, $CbzCH_2$), 4.39-4.31 (1H, dd, *J* 13.2, 4.4, H2), 4.24-4.18 (1H, m, H3), 4.16–4.01 (1H, m, H5), 3.29 (1H, dd, *J* 13.1, 10.0, H2'), 2.97 (1H, br s, H5'), 2.27–2.01 (2H, m, H4+H4'), 1.89–1.73 (1H, m, H5), 1.62 (1H, br s H5'); ^{13}C NMR (126 MHz, $CDCl_3$) δ_C 155.3 (C=O), 139.4 (C3a), 136.7 (ArC), 128.6 (ArCH), 128.2 (ArCH), 128.1 (C5a), 105.3 (C4a), 67.5 ($CbzCH_2$), 57.4 (C3), 49.1 (C3), 44.1 (C5), 30.9 (C4), 24.1 (C2); 13 signals observed; HRMS $C_{16}H_{19}N_3O_2Na$ $[M + Na]^+$ requires 308.1375, found 308.1386.

Benzyl 3-(1*H*-indazol-1-yl)pyrrolidine-1-carboxylate (**13e**)

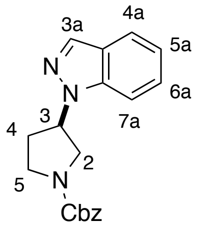

(**13e**) was synthesised using general method C using benzyl 2,3-dihydro-1H-pyrrole-1-carboxylate (50.0 mg, 0.26 mmol), indazole (64.0 mg, 0.50 mmol), TRIP thiol (27.0 mg, 0.125 mmol) and catalyst A (5.0 mg, 2 mol%). The reaction was irradiated for 16 hours followed by purification by column chromatography (2:1 to 1:1 hexane/ethyl acetate) and isolated as a colourless oil (27.0 mg, 34%) as a mixture of rotamers.

^{1}H NMR (501 MHz, Chloroform-*d*) δ_H 7.94 (1H, d, *J* 3.6, H3a), 7.70 (1H, d, *J* 8.8, H4a), 7.63 (1H, d, *J* 8.4, H7a), 7.42–7.27 (6H, m, Cbz+H5a), 7.09 (1H, dd, *J* 8.4, 6.9, H6a), 5.32–5.01 (3H, m, $CbzCH_2$+H3), 4.12–3.92 (2H, m, H2+H2'), 3.85–3.60 (2H, m, H5+H5'), 2.73–2.43 (2H, m H4+H4'); ^{13}C NMR (126 MHz, $CDCl_3$) δ_C 154.8 (C=O), 149.0 (ArC), 136.8 (ArC),

136.7 (ArC), 128.7 (ArCH), 128.2 (ArCH), 128.1 (ArCH), 126.4 (C5a), 126.3 (C5a), 122.1 (C6a), 121.9 (ArC), 121.3 (C3a), 120.3 (C7a), 117.7 (C4a), 67.2 (CbzCH_2), 62.1 (C3), 61.3 (C3), 51.8 (C2), 51.6 (C2), 44.9 (C5), 44.5 (C5), 32.7 (C4), 31.7 (C4); 23 signals observed; HRMS $C_{19}H_{19}N_3NaO_2$ $[M + Na]^+$ requires 344.1375, found 344.1380.

Control reactions

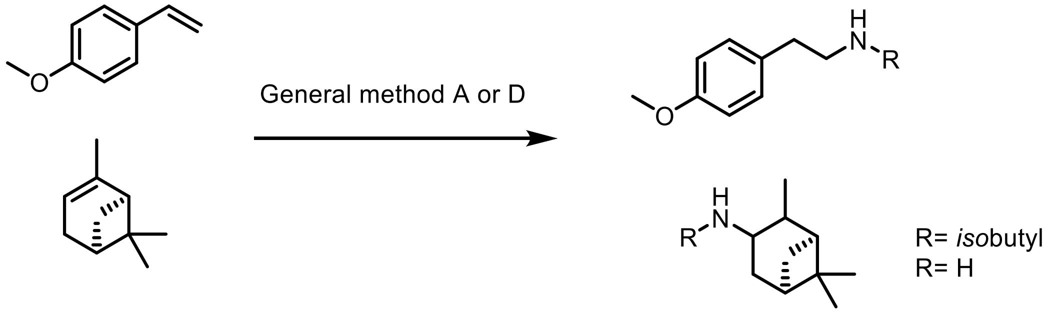

Both reactions with 4-methoxystyrene and α pinene were carried out under identical conditions to general method A or D. Analysis of the LCMS and crude NMR showed no formation of product.

Stern Volmer fluorescence quenching experiments

Stern–Volmer fluorescence quenching experiments were run with freshly prepared solutions of Cat A (4.2 x10^{-4} M) in toluene at ambient temperature under a nitrogen atmosphere. Stock solutions of quencher were made up in anhydrous toluene to a final concentration of 360mM. 3.6ml of photocatalyst was added to the cuvettes which were irradiated at 380nm and luminescence recorded at 465nm. Sequential additions of 20 µL (2mM increase in quencher concentration) of quencher were made and irradiation and luminescence recorded twice per sample.

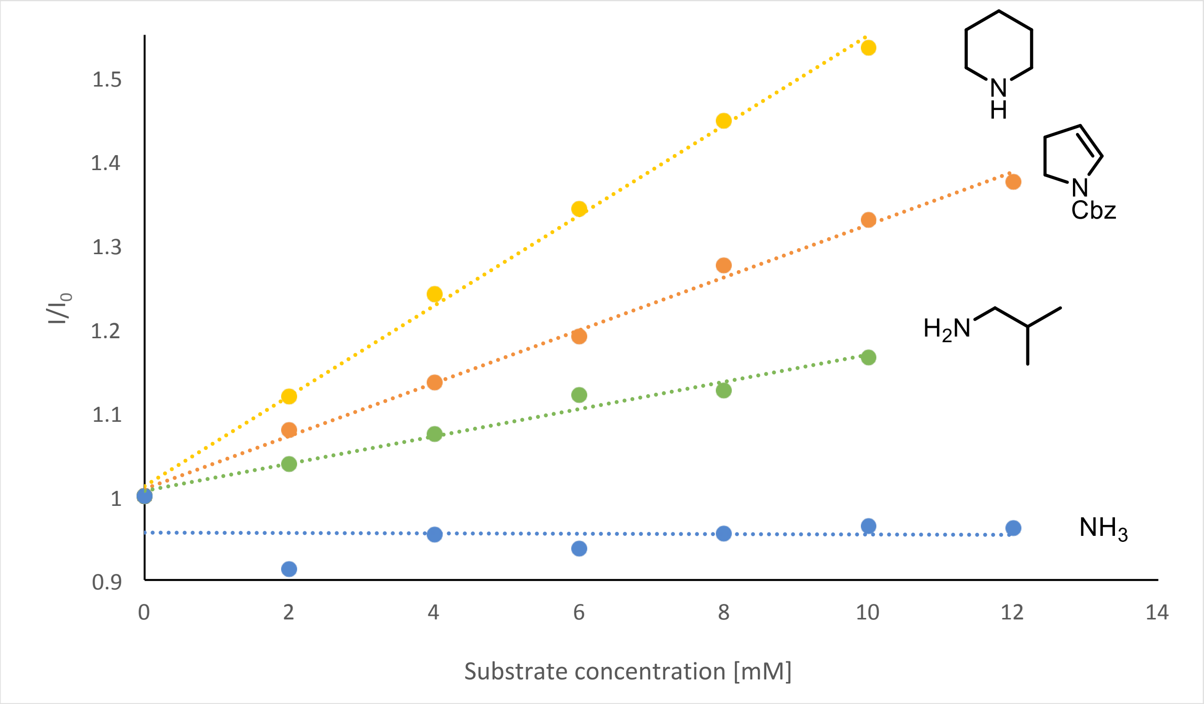

Electrochemical data: the oxidation potential of *N*-Cbz-dihydropyrrole is not known, but the corresponding Boc derivative has a value of 0.98 V vs Ag/Ag^+ 0.01M in MeCN (C. Adouama, R. Keyrouz, G. Pilet, C. Monnereau, D. Gueyrard, T. Noël, M. Médebielle, *Chem. Commun*., **2017**, *53*, 5653-5656) which corrects to ca. 0.90 V versus Fc/Fc^+ (using the correction factors in V. V. Pavlishchuk, A. W. Addison, *Inorg. Chim. Acta*, **2000**, *298*, 97-102). This compares with values of 0.59 V for the photoexcited Ir catalyst (S. Ladouceur, D. Fortin, E. Zysman-Colman, *Inorg. Chem*. **2011**, *50*, 11514−11526) and 1.14 V for isobutylamine (J. L. Bourdelande, I. Gallardo, G. Guirado, *J. Am. Chem. Soc.* **2007**, *129*, 2817−2821) (all values corrected to values *vs* Fc/Fc^+ as before). These figures would suggest formation of only a very small equilibrium concentration of oxidation products from interaction with the photoexcited catalyst (K_{eq} ca. 5.9 x 10^{-6} and 2.0 x 10^{-9} respectively), with electron transfer to enecarbamate favoured by a factor of ca. 3000. This supports the observed weak quenching of photoactivated catalyst by the enecarbamate and the even weaker quenching by isobutylamine.

Compound Spectra

Benzyl 2,3,4,5-tetrahydro-1*H*-azepine-1-carboxylate

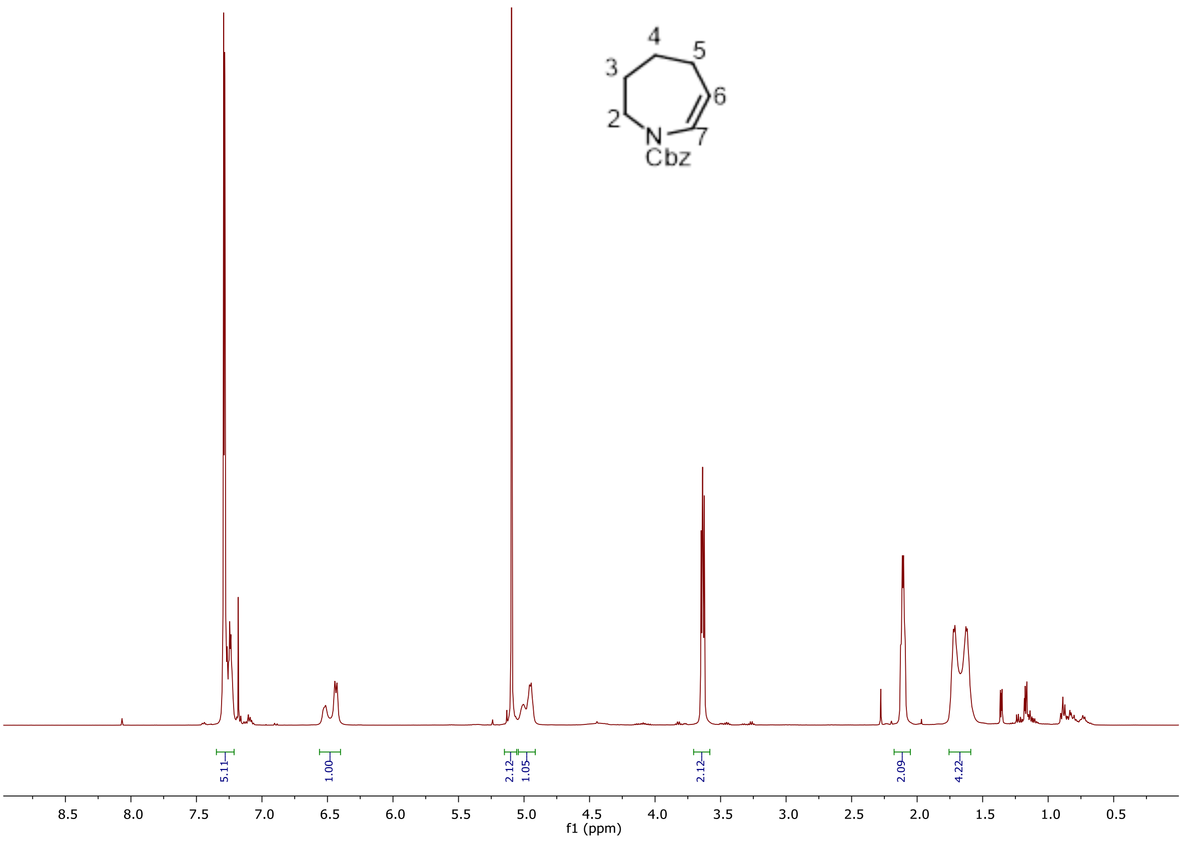

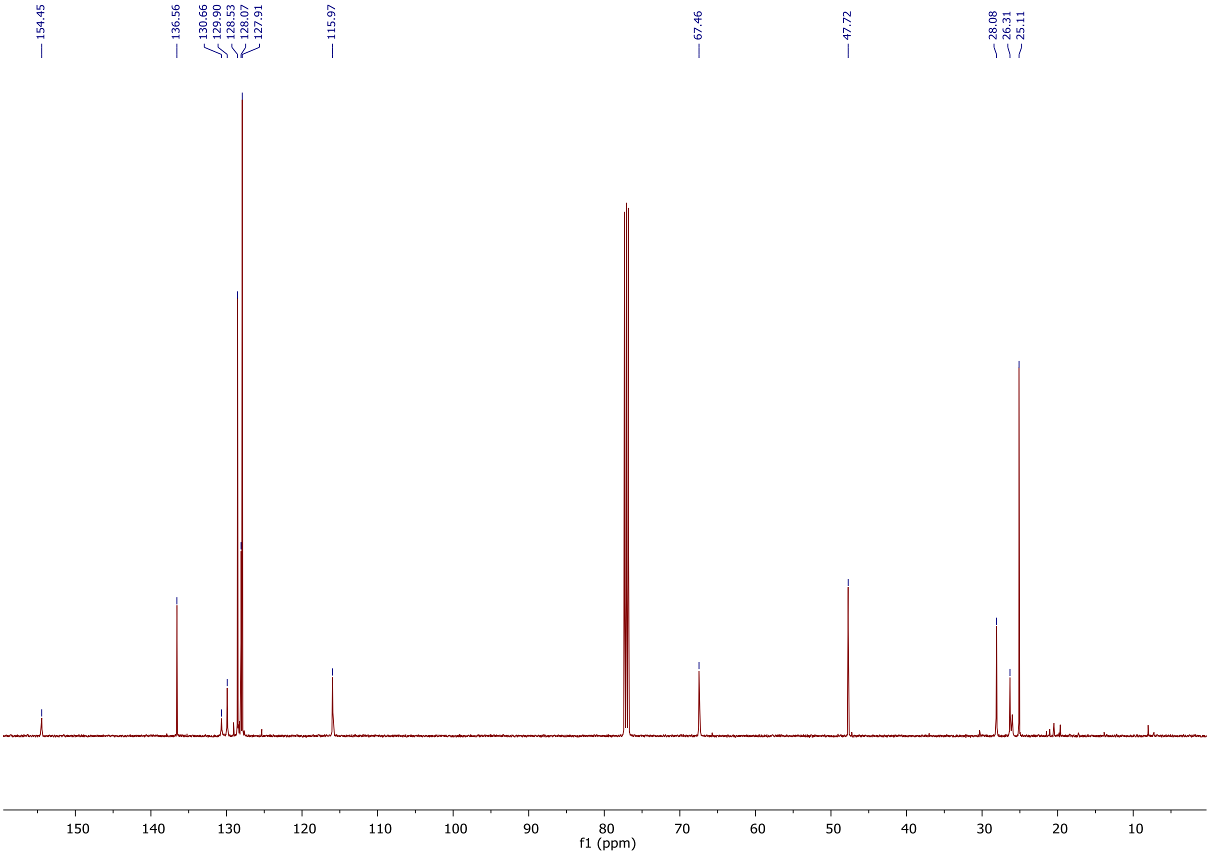

1-Benzyl-3,4-dihydropyridin-2(1*H*)-one

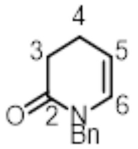

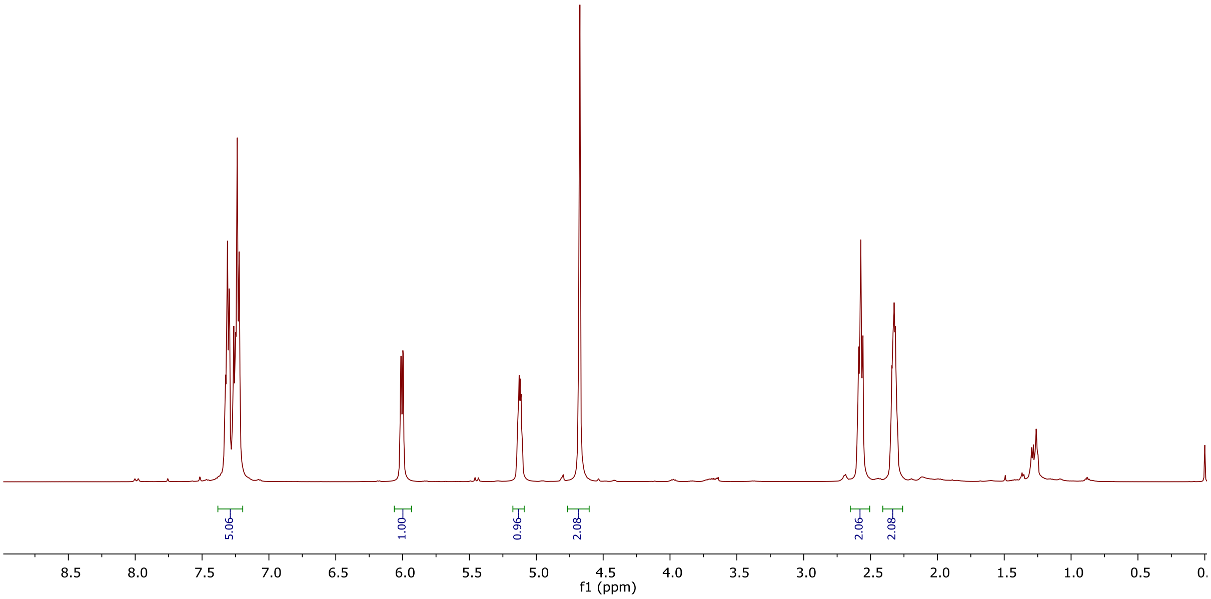

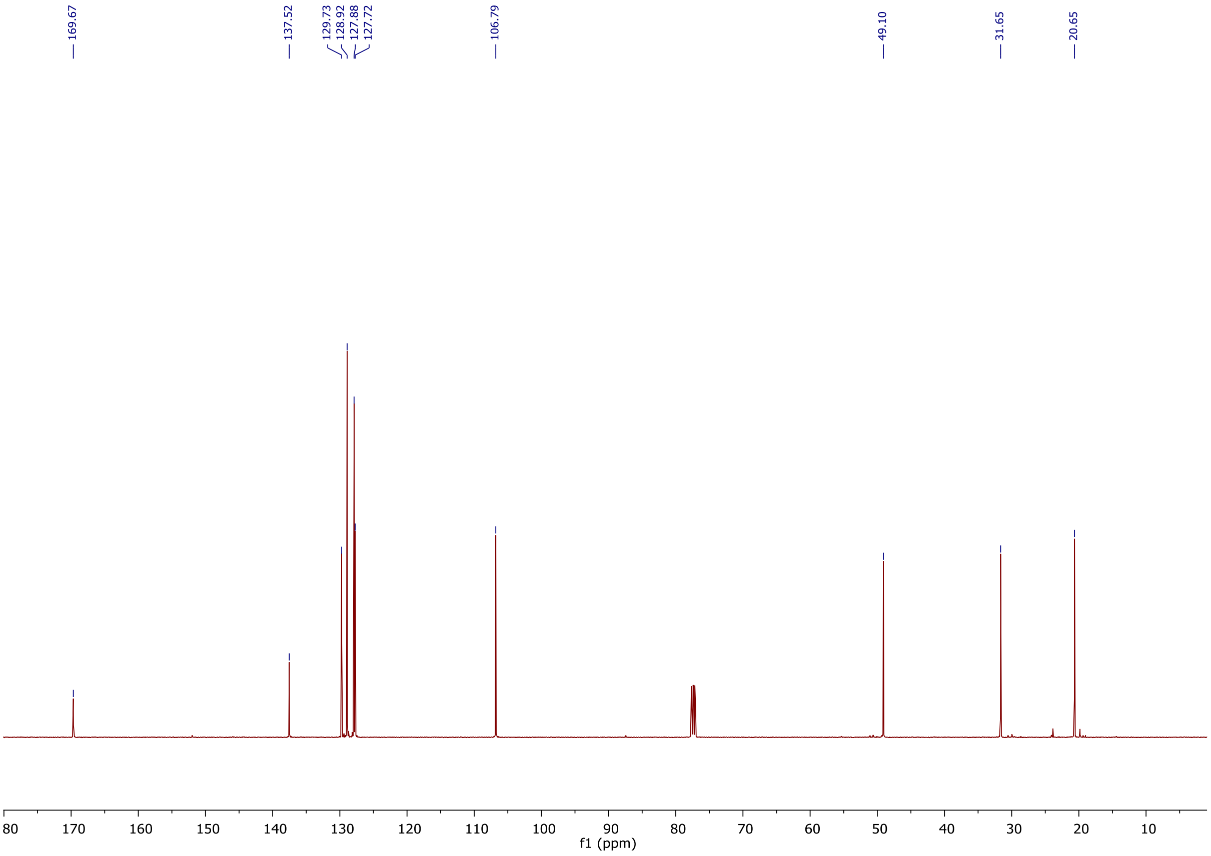

Benzyl 4-(*tert*-butyl)-2,3,4,5-tetrahydro-1*H*-azepine-1-carboxylate

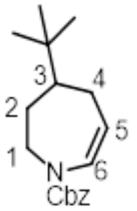

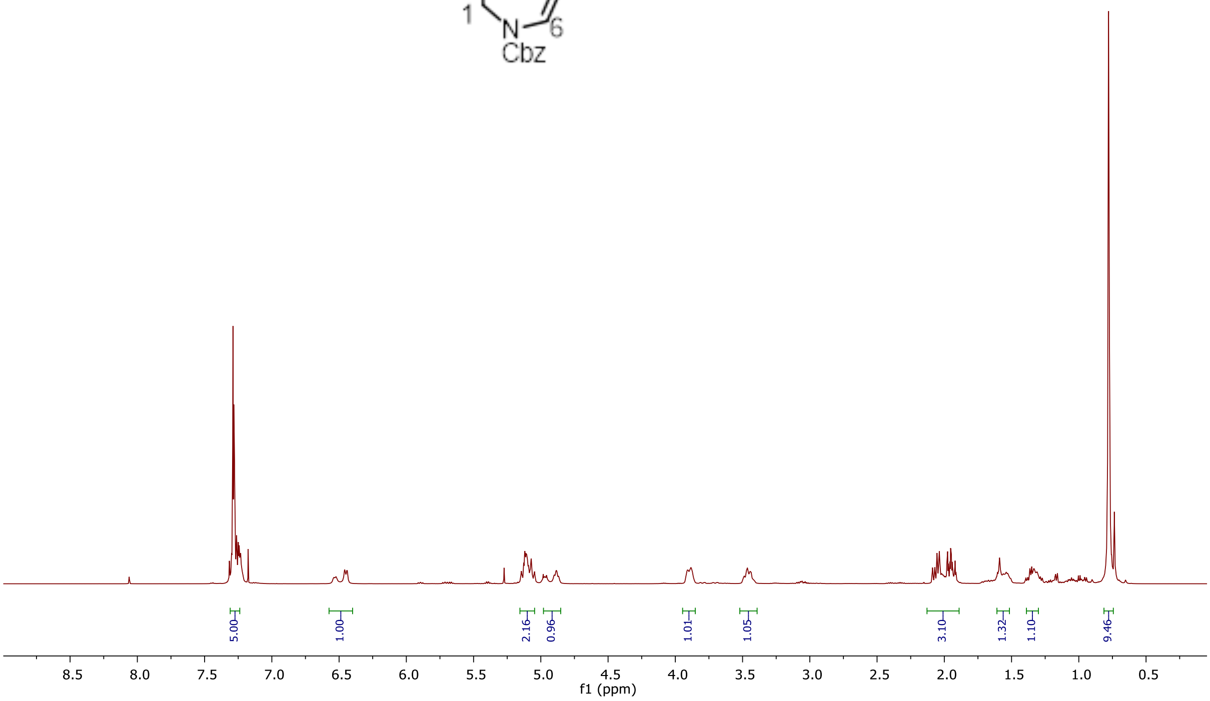

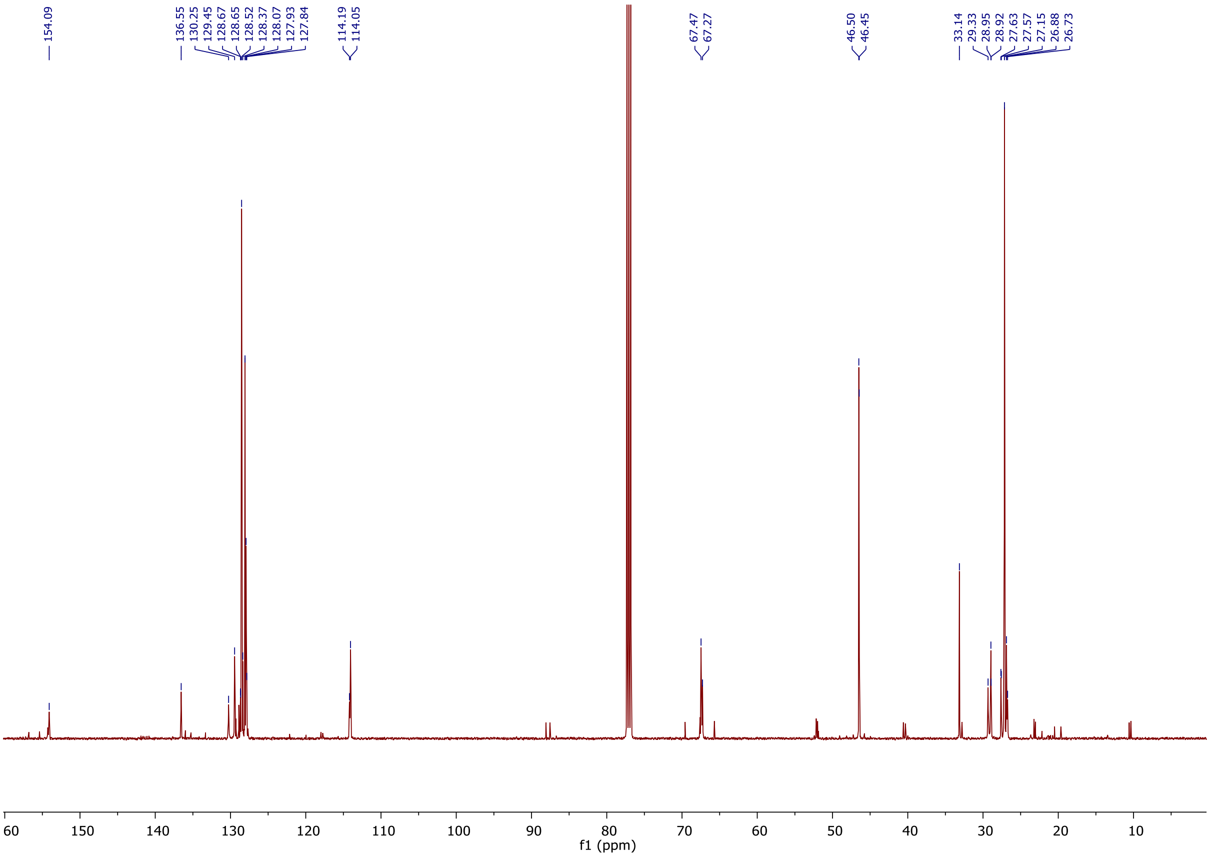

Benzyl 2,2-dimethyl-2,3-dihydro-1*H*-pyrrole-1-carboxylate

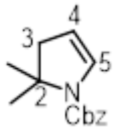

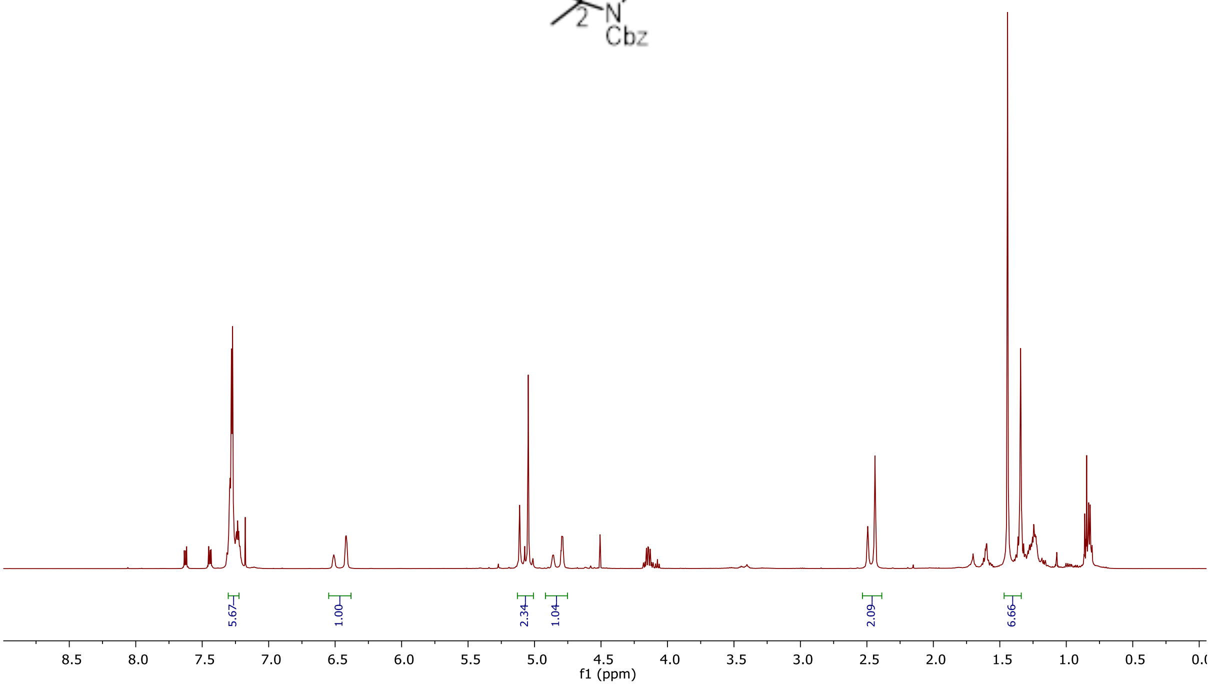

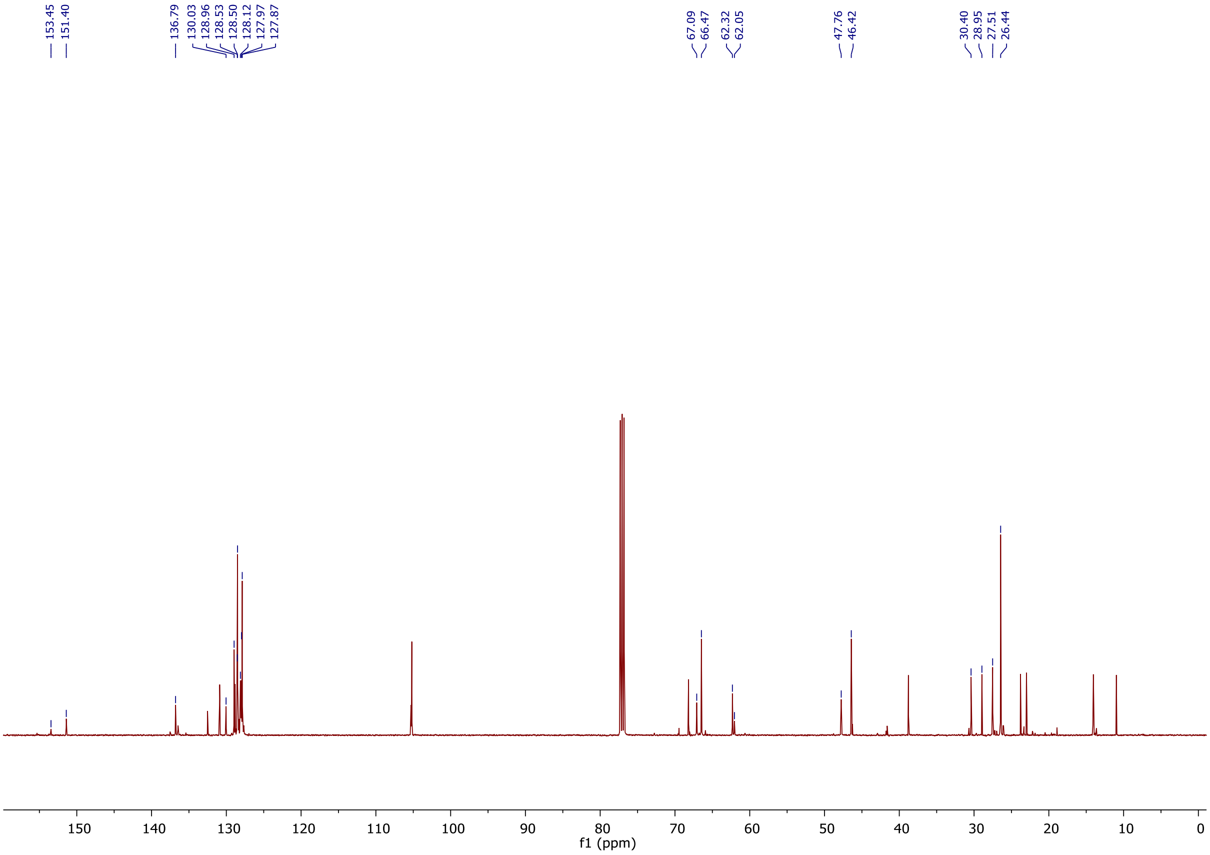

Benzyl 3-azaspiro[4.5]dec-4-ene-1-carboxylate

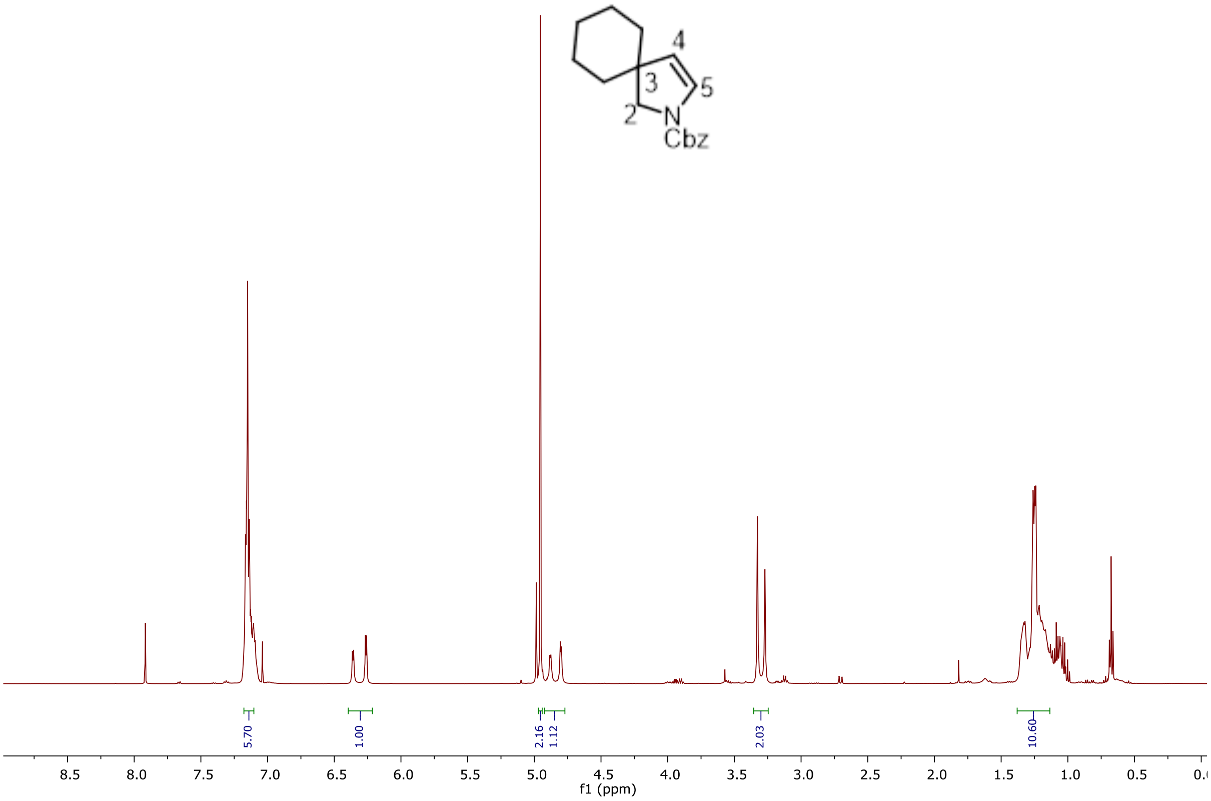

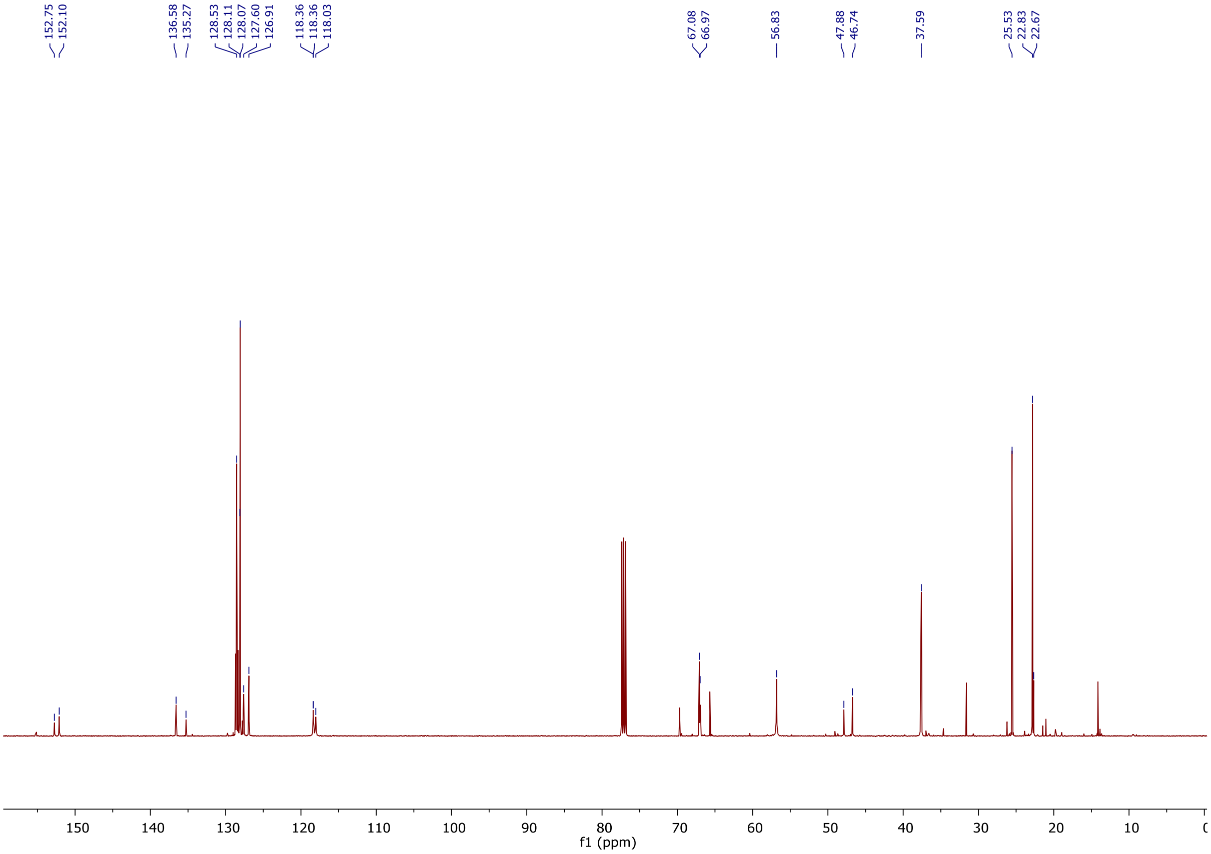

Benzyl hexahydro-2*H*-isoindole-1-carboxylate

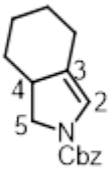

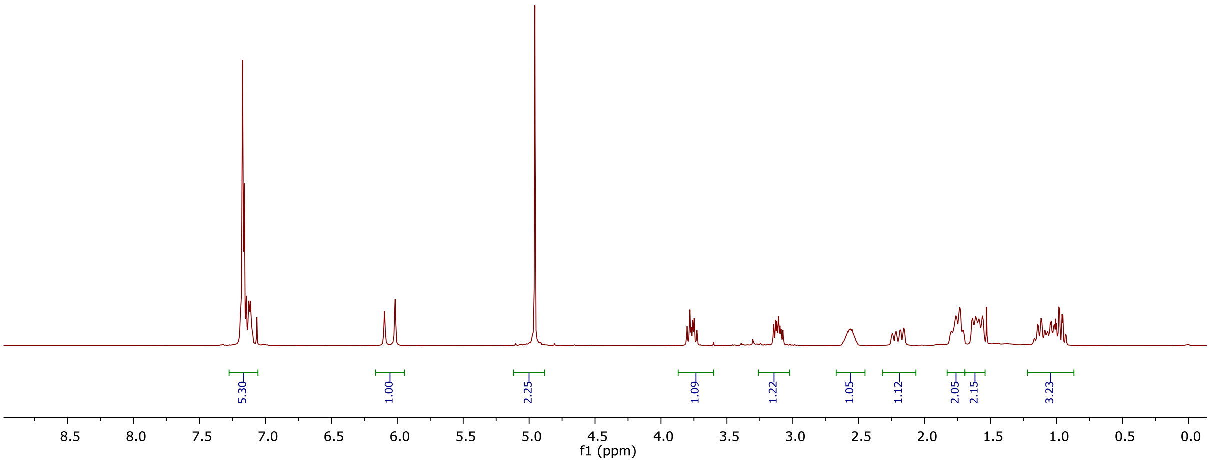

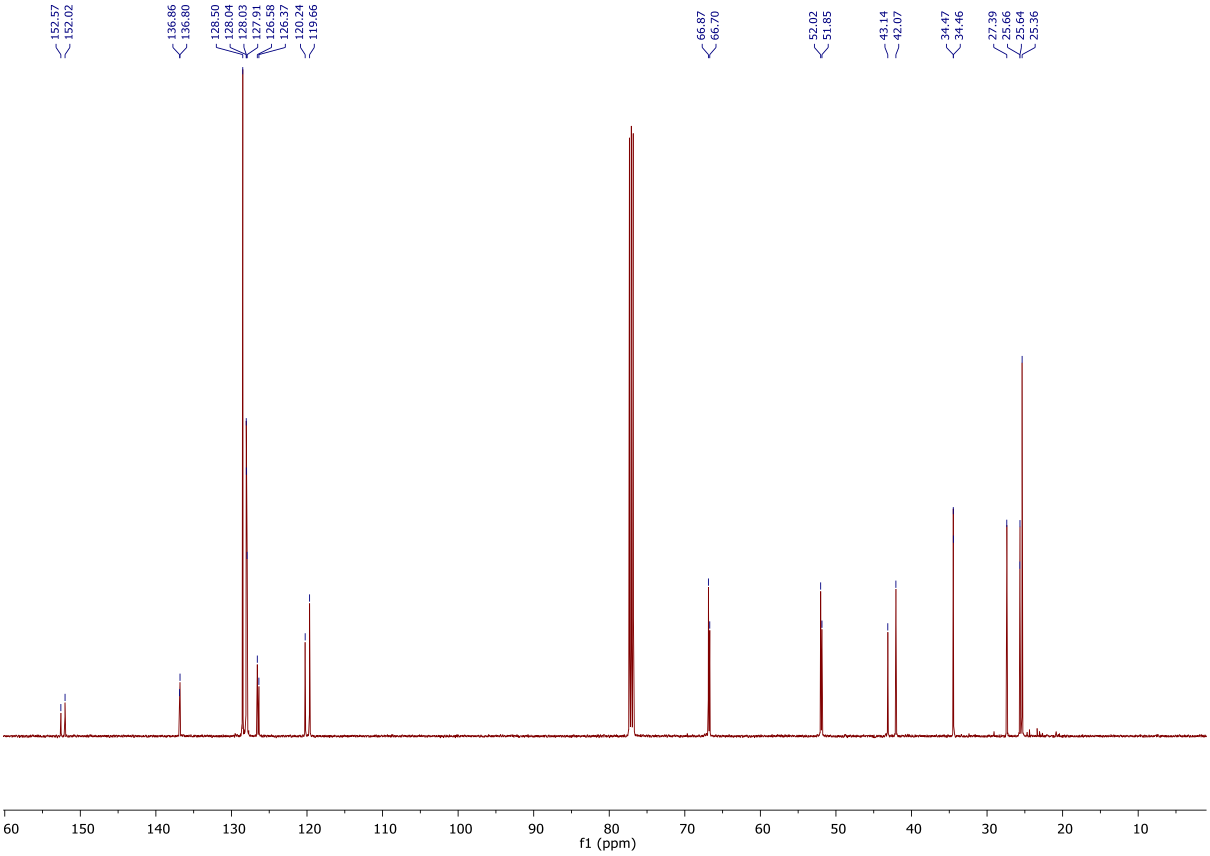

Benzyl 3-(*iso*butylamino)pyrrolidine-1-carboxylate (**6a**)

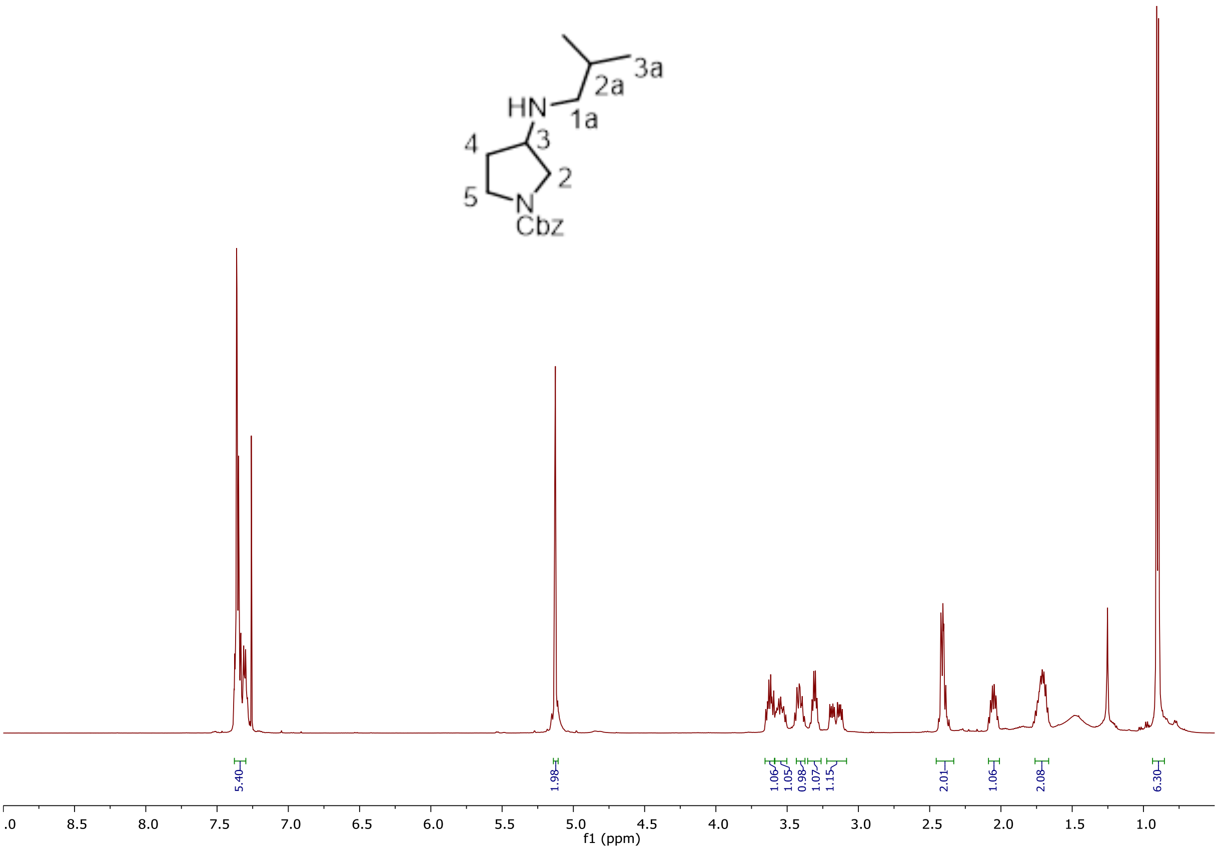

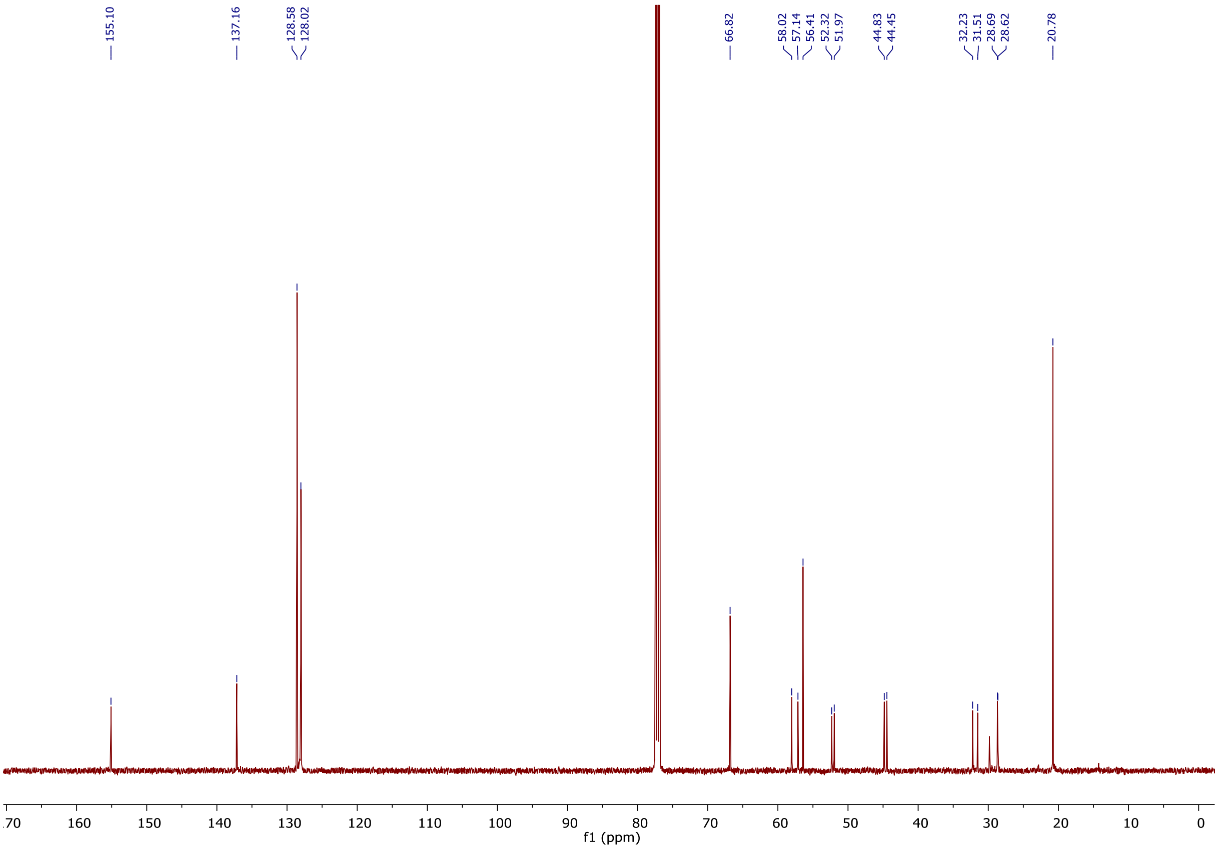

Benzyl 3-(*iso*butylamino)piperidine-1-carboxylate (**6b**)

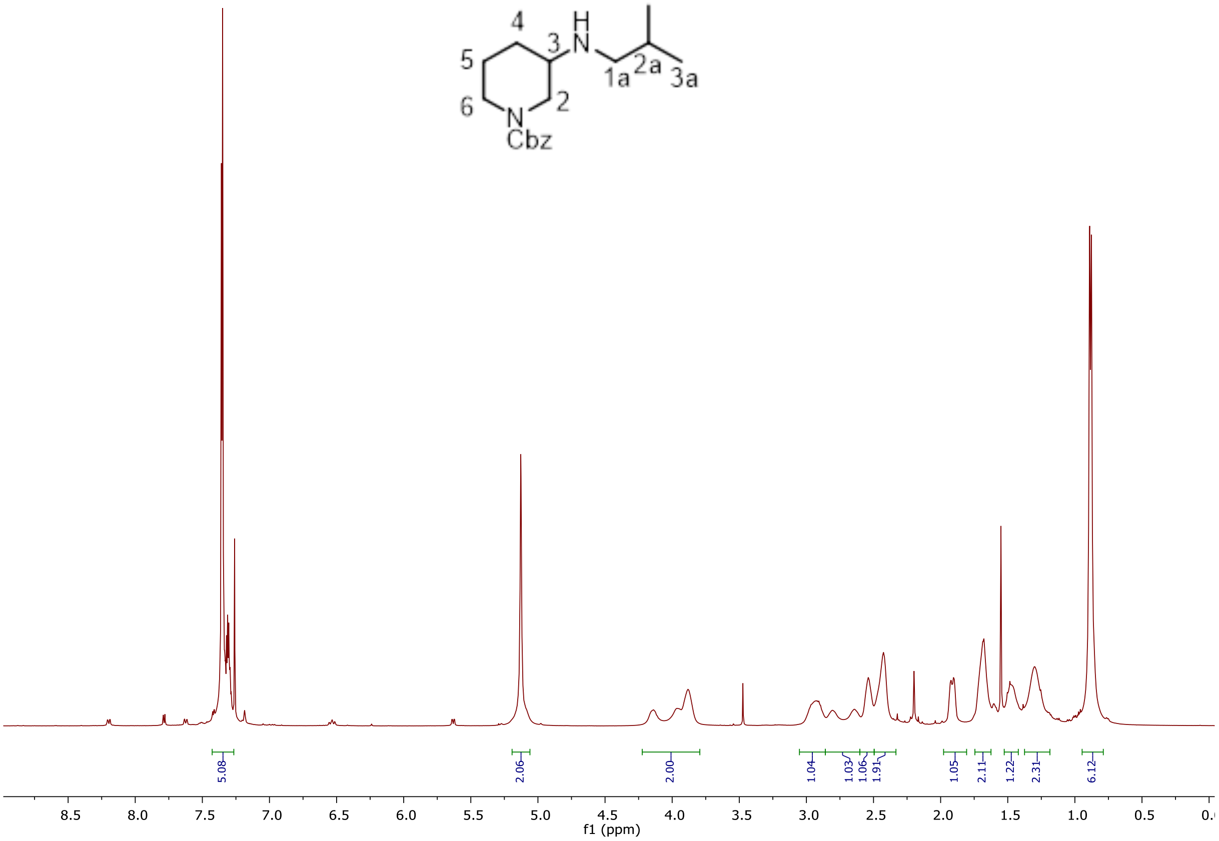

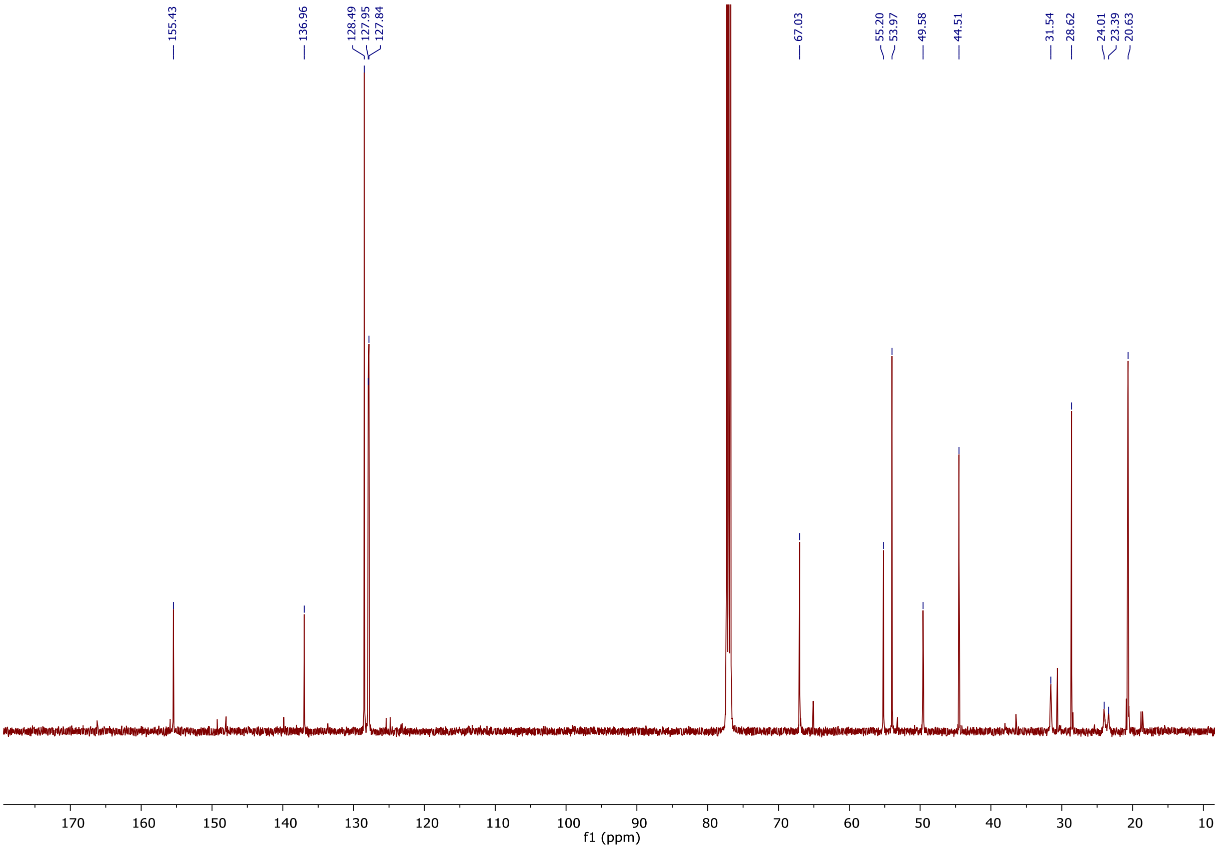

Benzyl 3-(*iso*butylamino)azepane-1-carboxylate (**6c**)

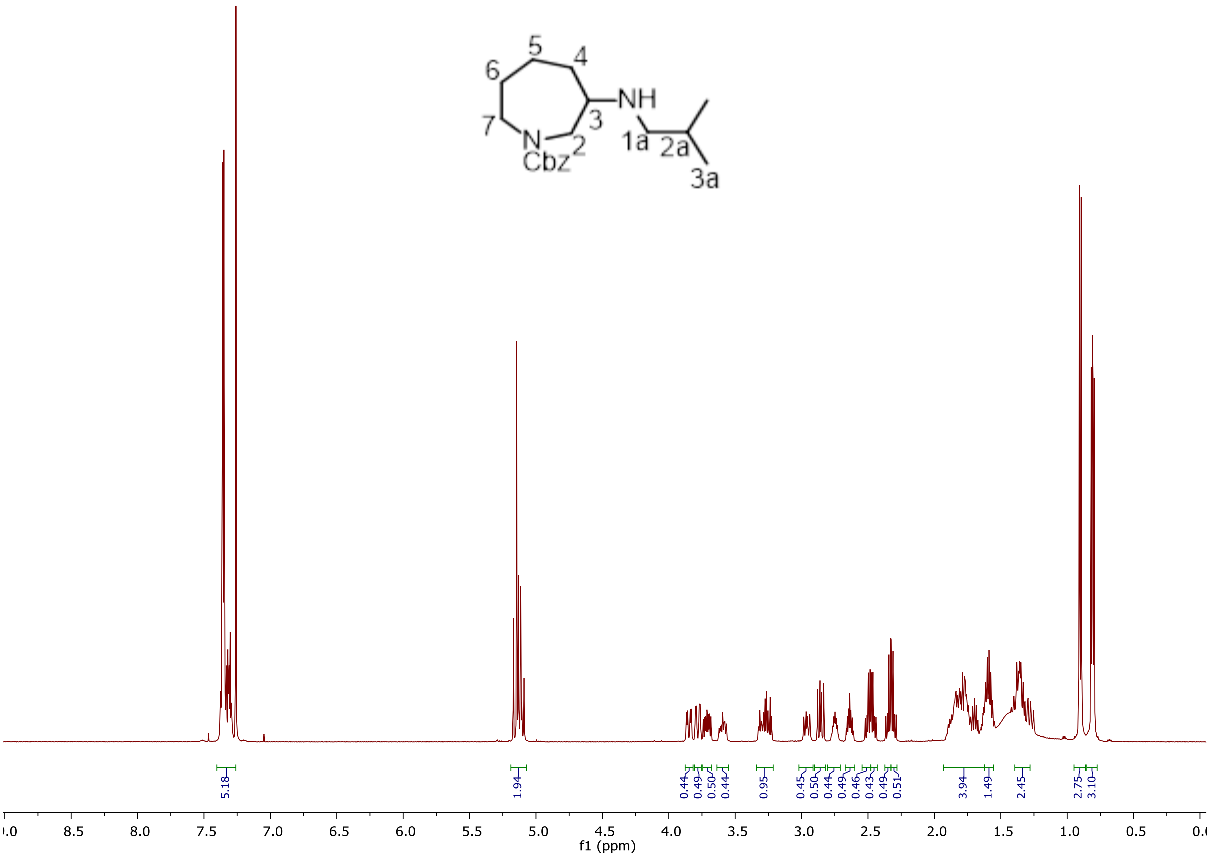

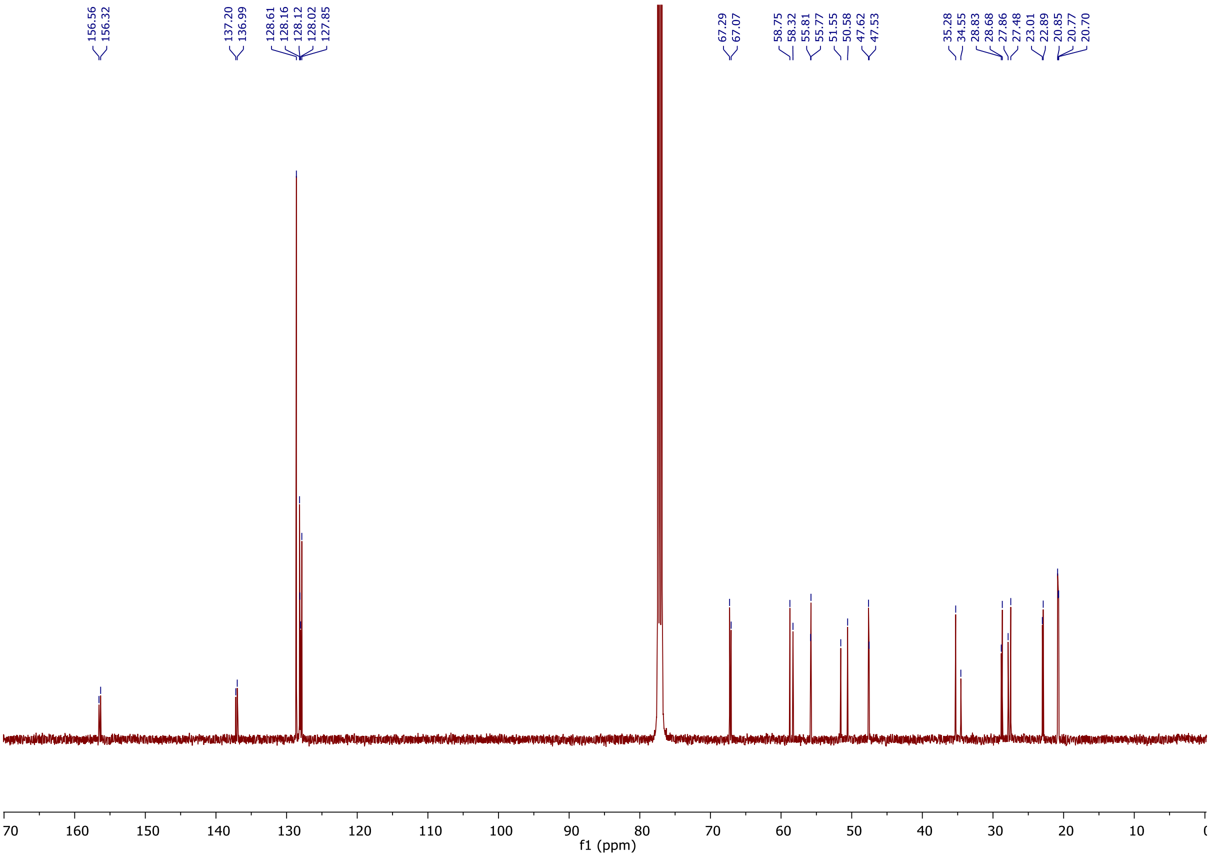

1-Benzyl-5-(*iso*butylamino)piperidin-2-one (**6d**)

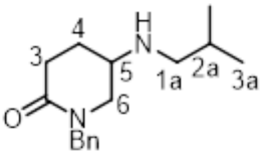

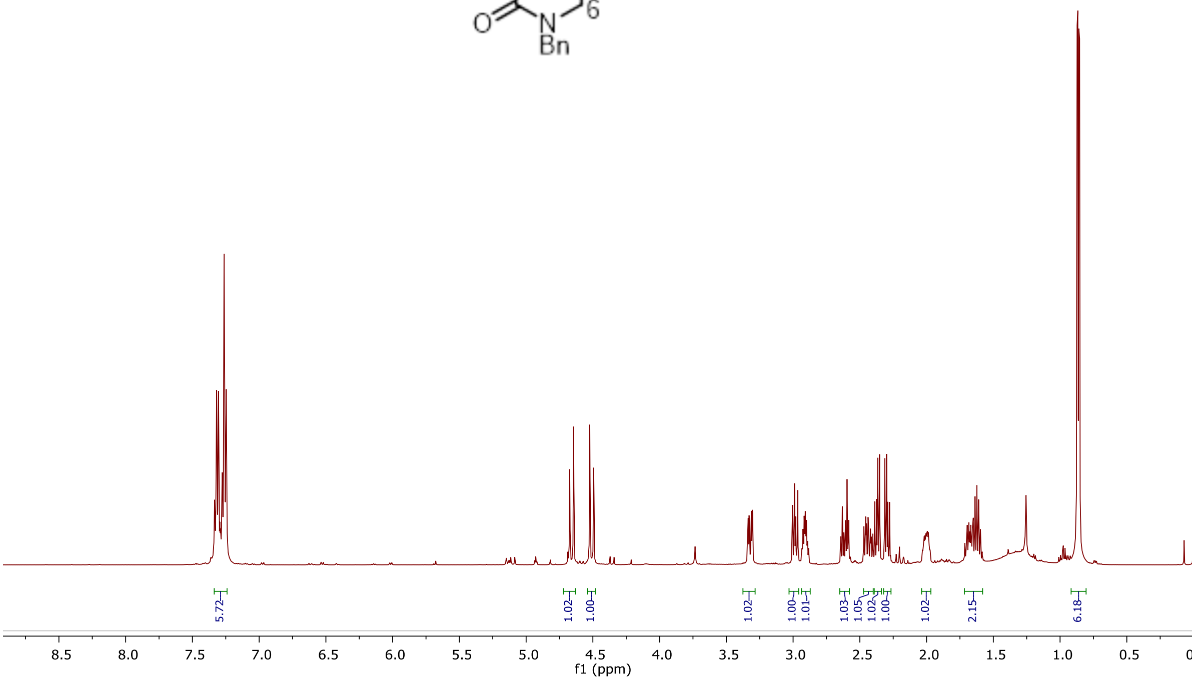

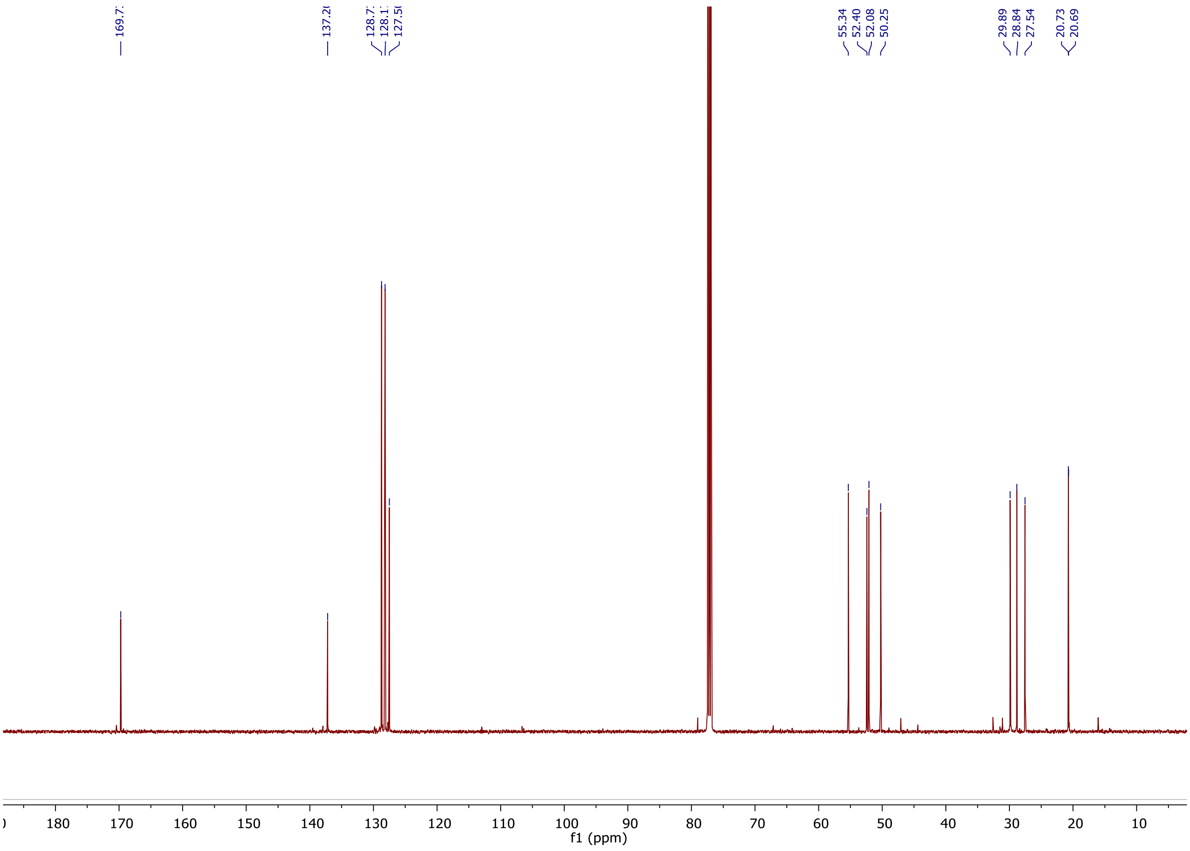

Benzyl-5-(*iso*butylamino)-2-methylpiperidine-1-carboxylate (**6e**)

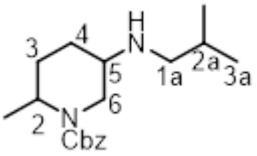

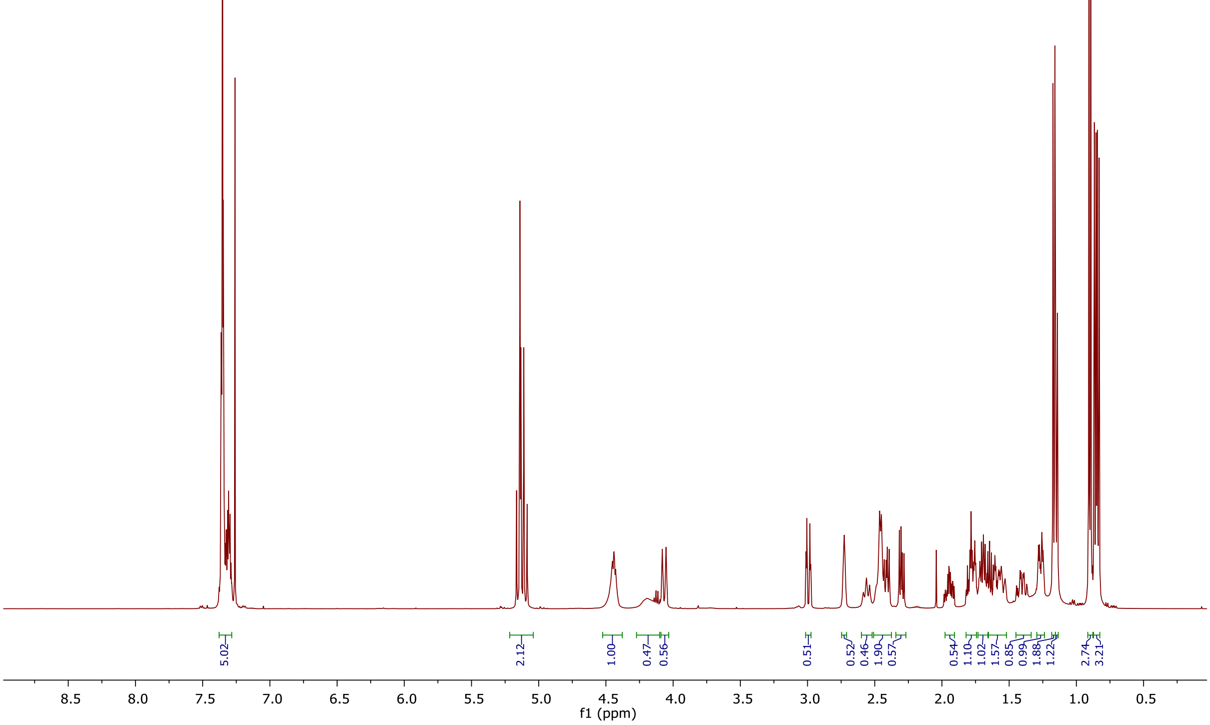

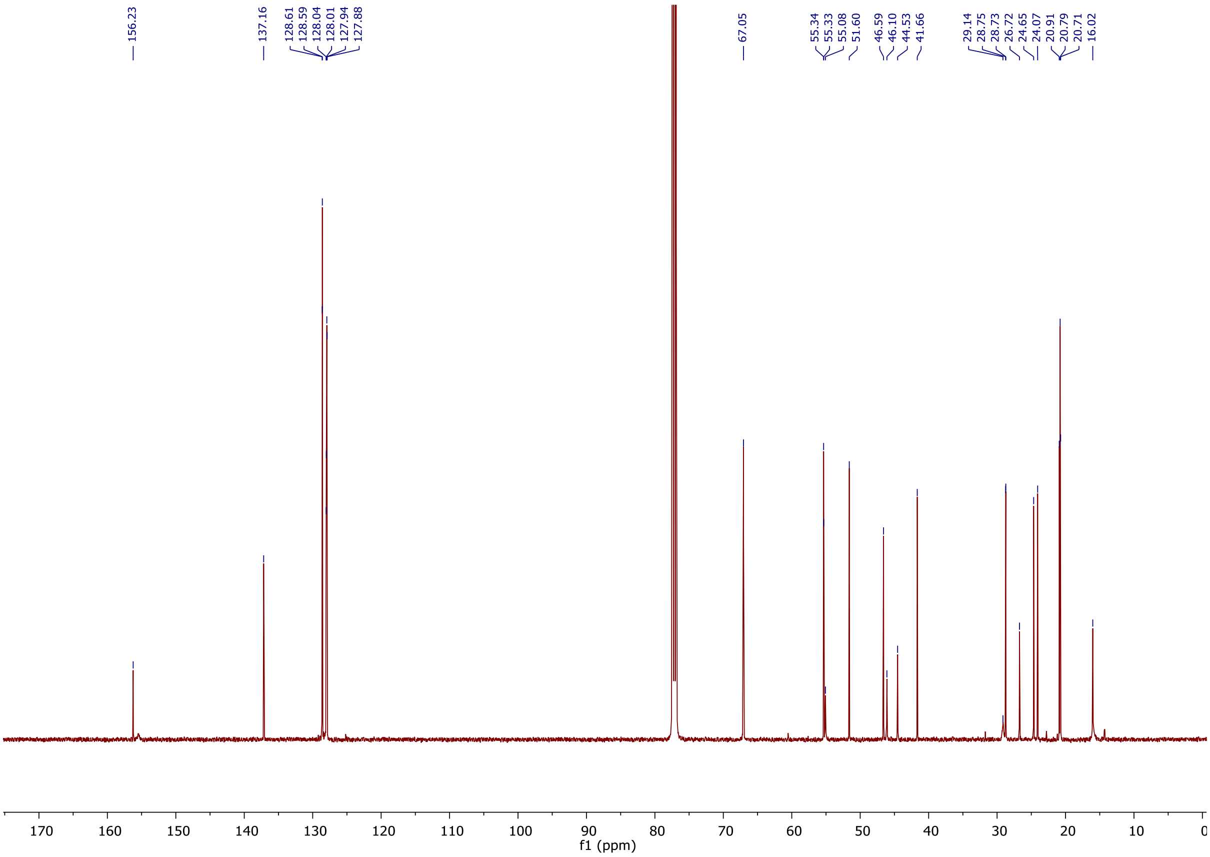

Benzyl 3-(*iso*butylamino)-4-methylpiperidine-1-carboxylate minor (**6f**)

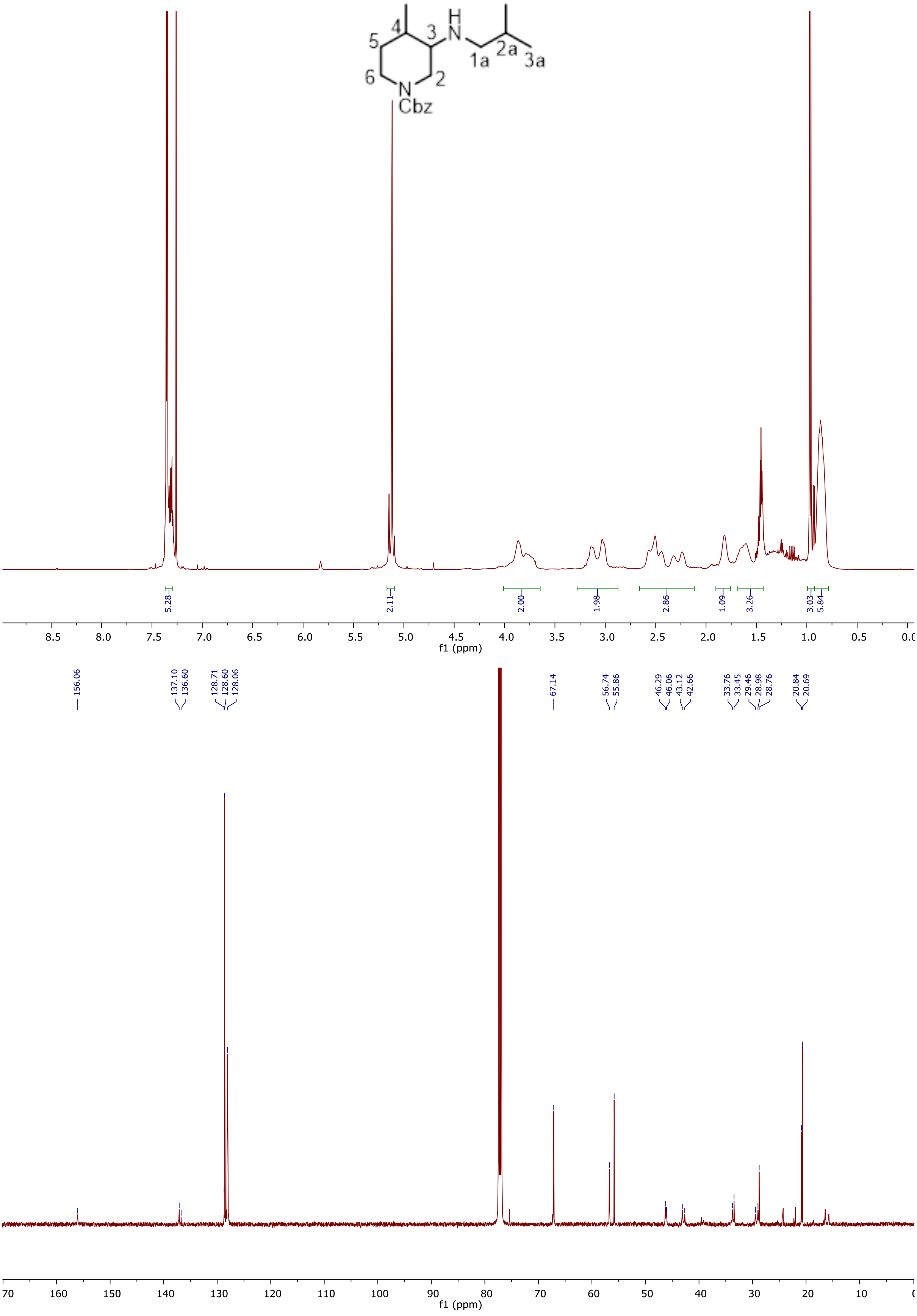

Benzyl 3-(*iso*butylamino)-4-methylpiperidine-1-carboxylate (**6f**)

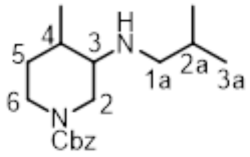

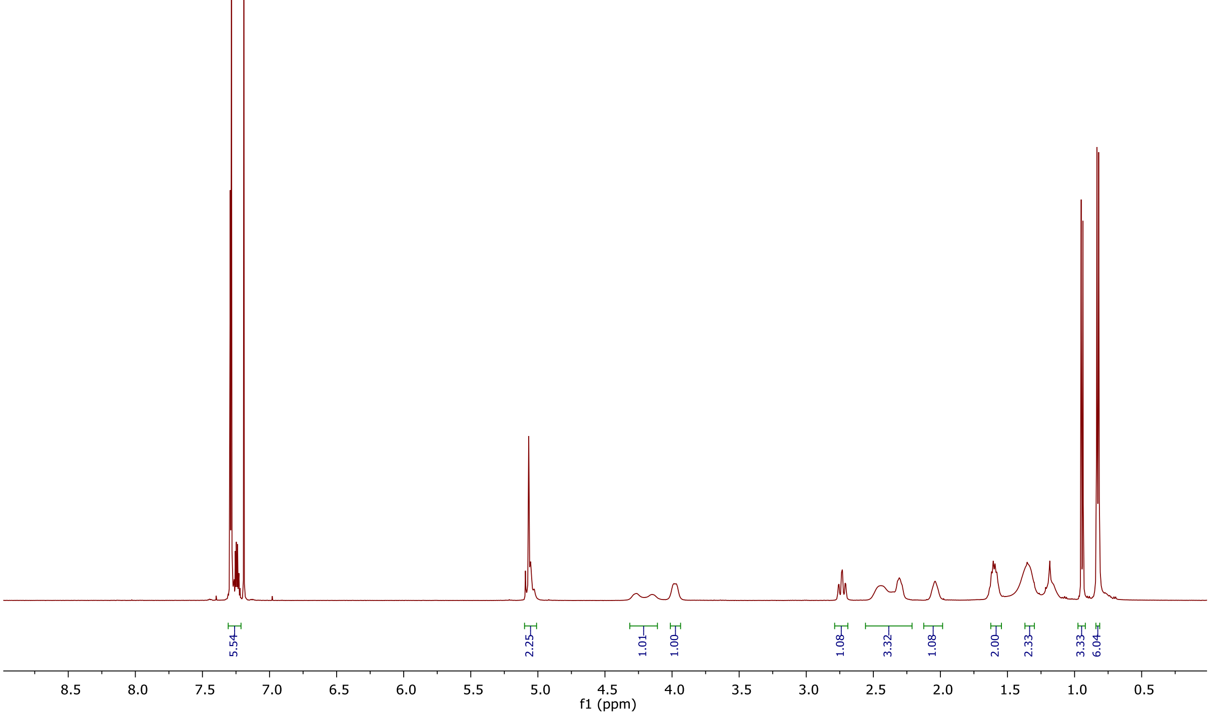

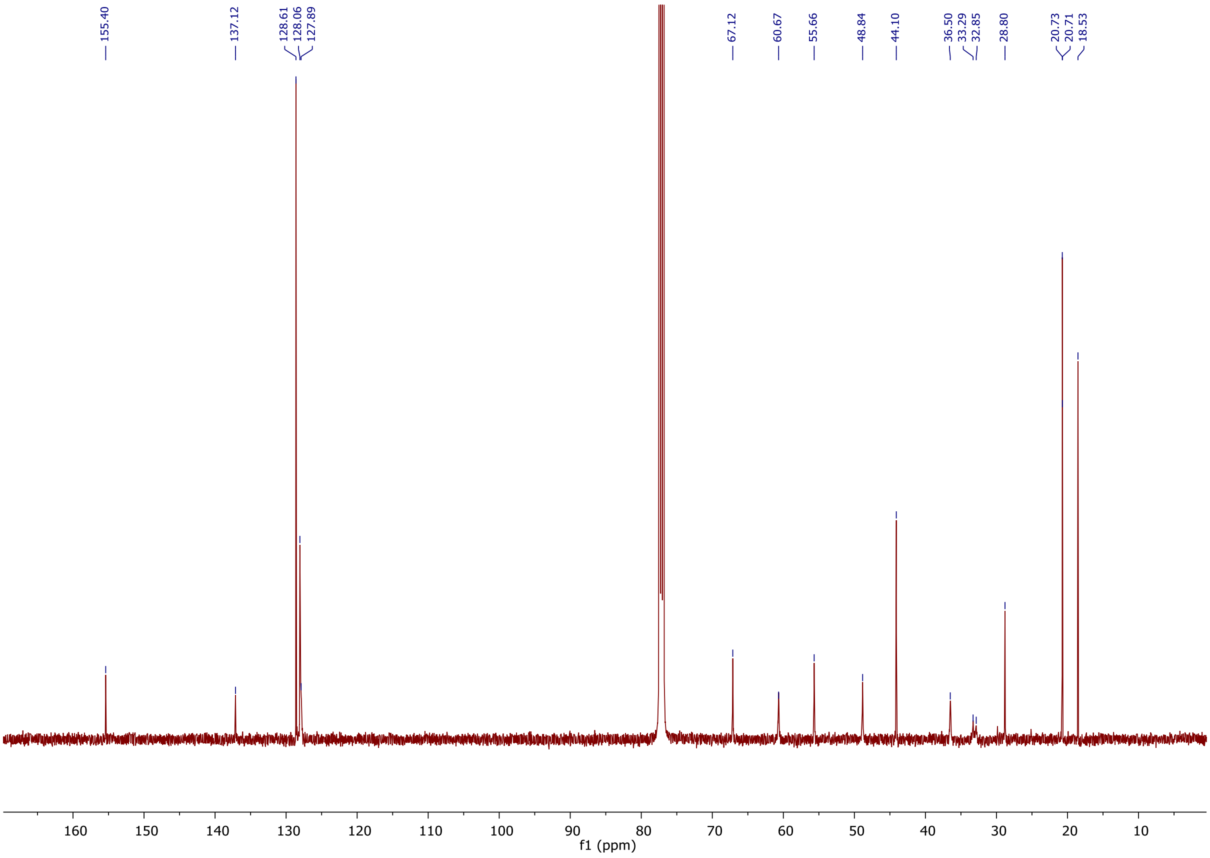

Benzyl 5-(*tert*-butyl)-3-(*iso*butylamino)azepane-1-carboxylate (**6g**)

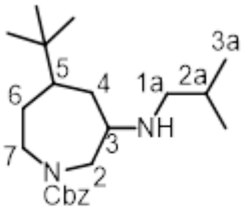

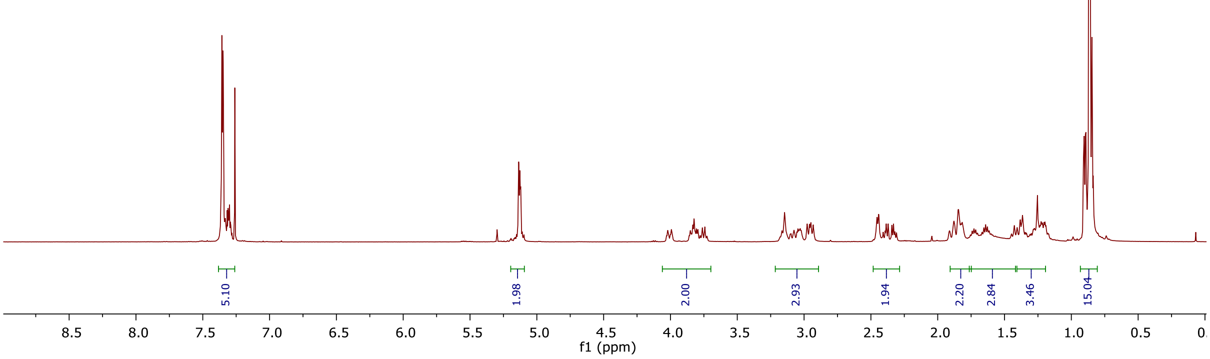

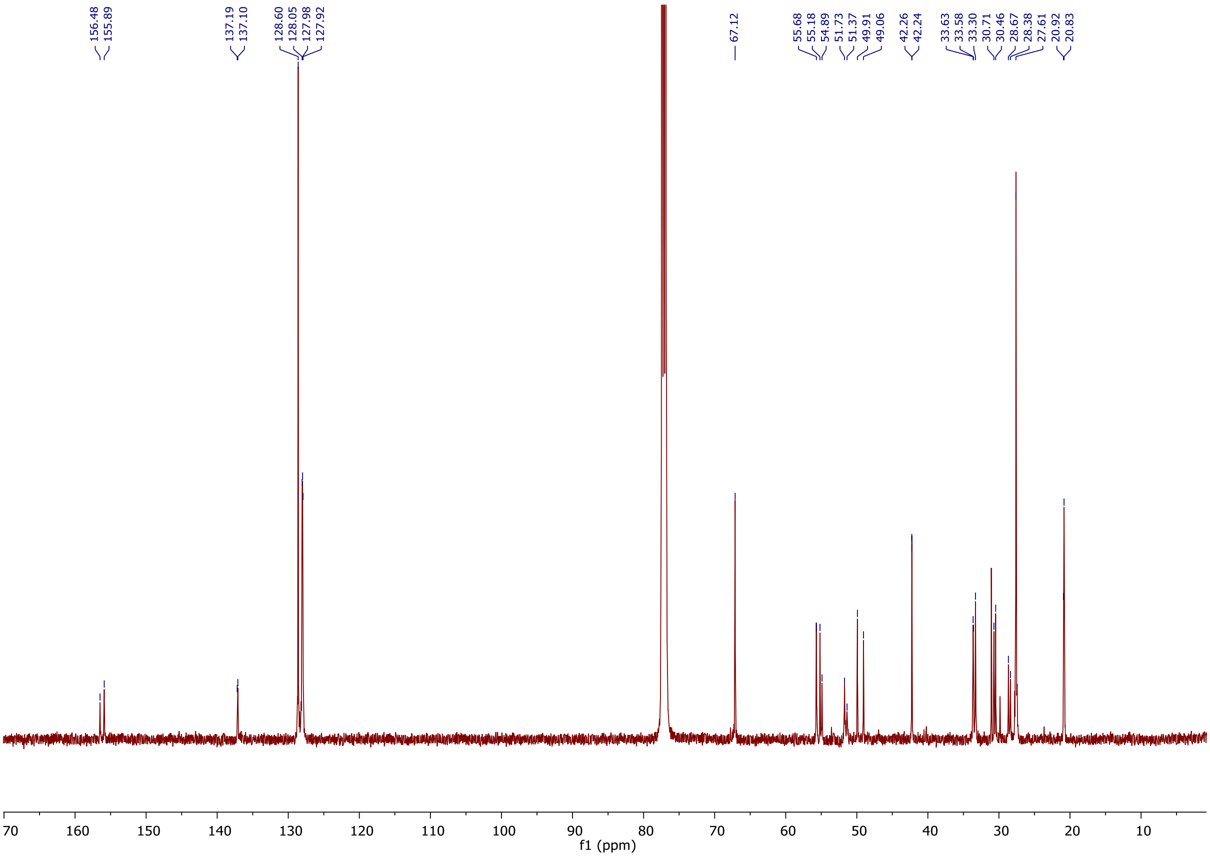

Benzyl 4-(*iso*butylamino)-2,2-dimethylpyrrolidine-1-carboxylate (**6h**)

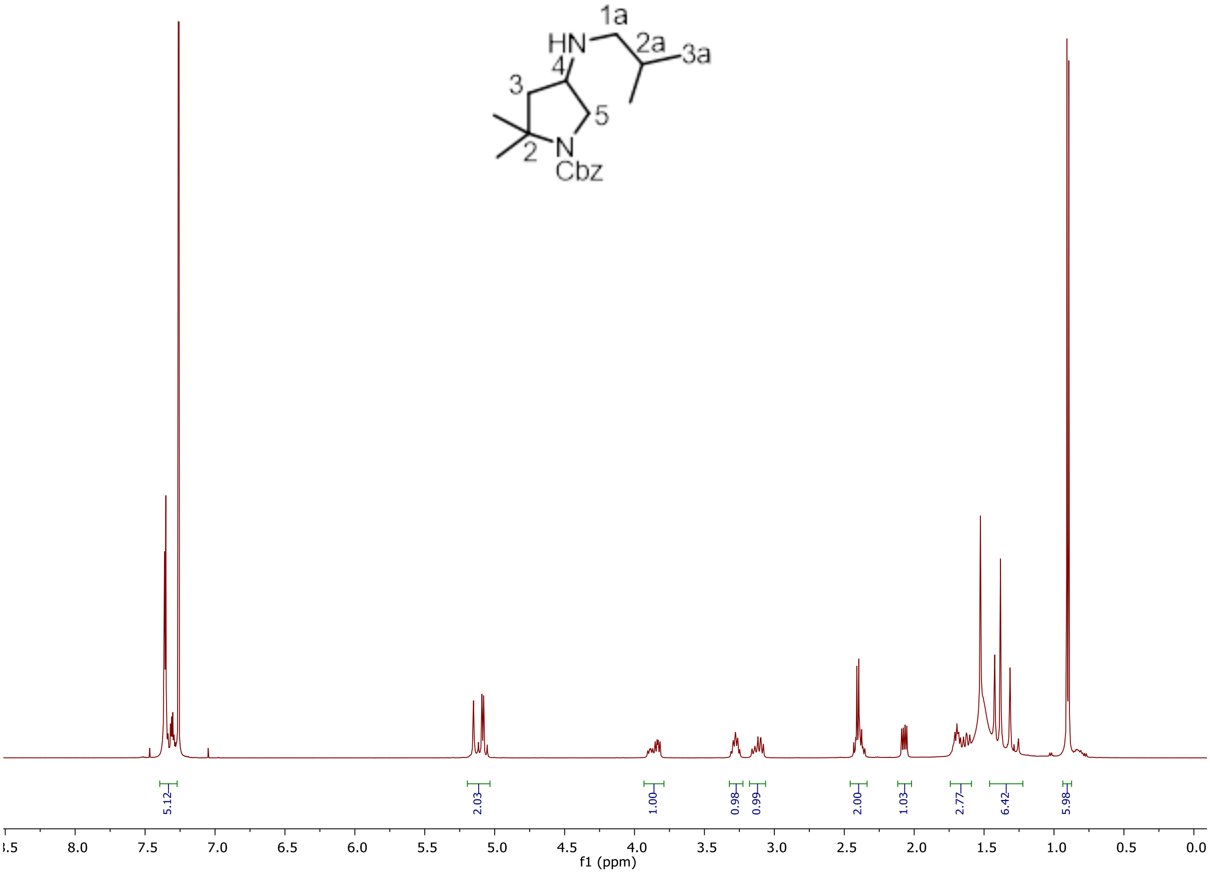

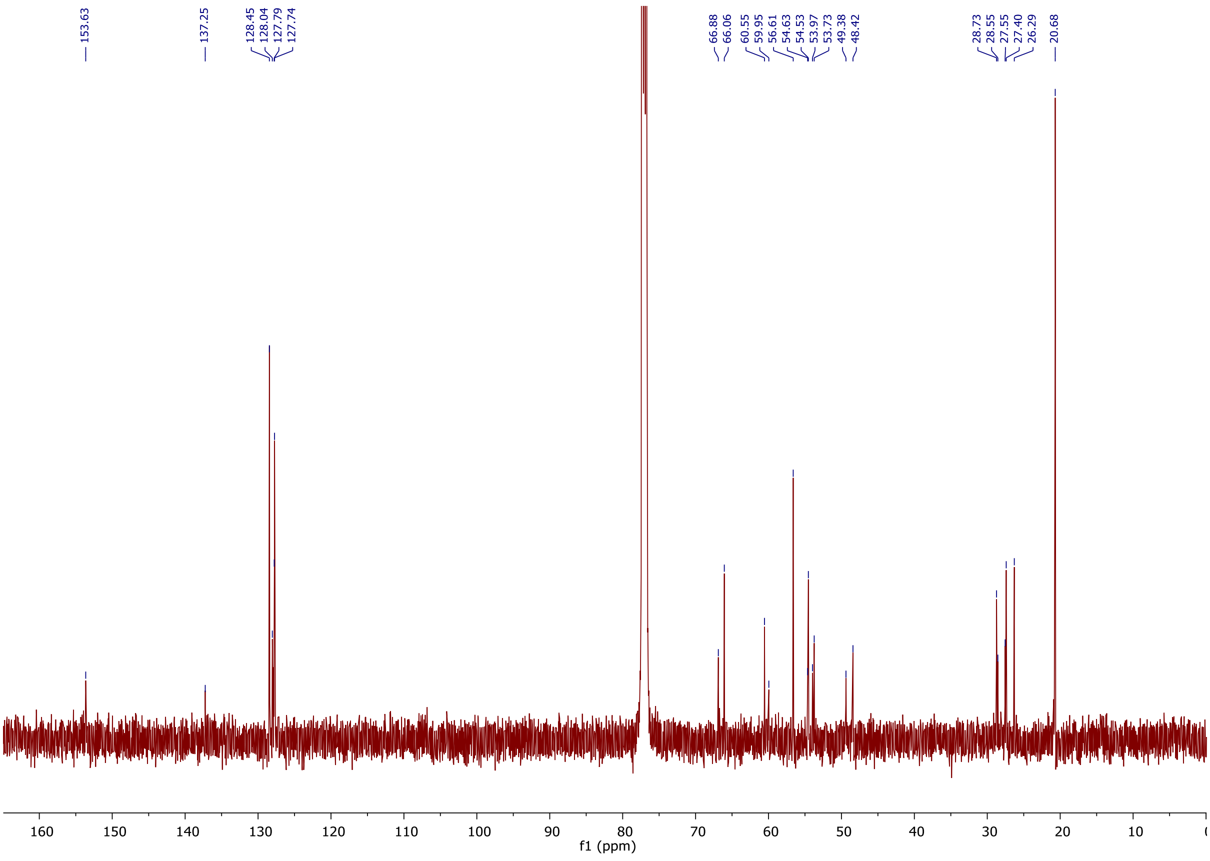

Benzyl 4-(*iso*butylamino)-2-azaspiro[4.5]decane-2-carboxylate (**6i**)

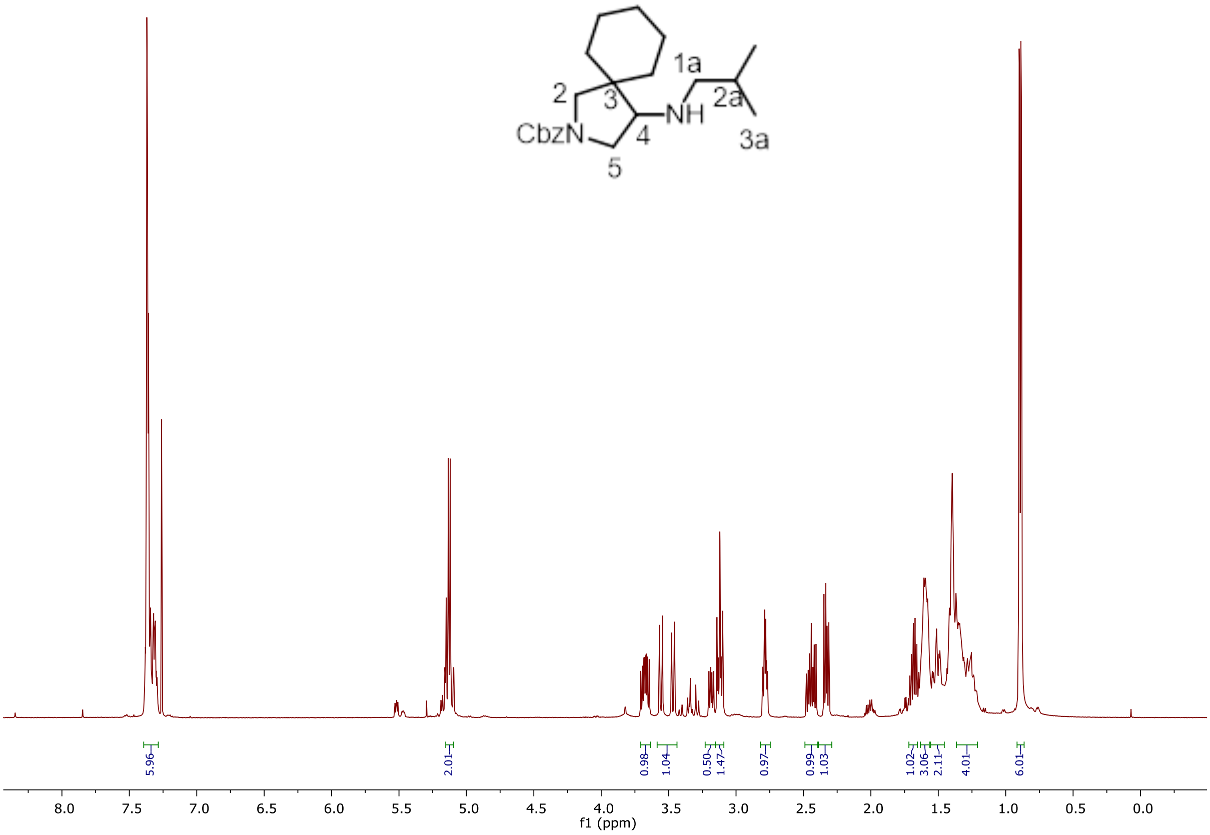

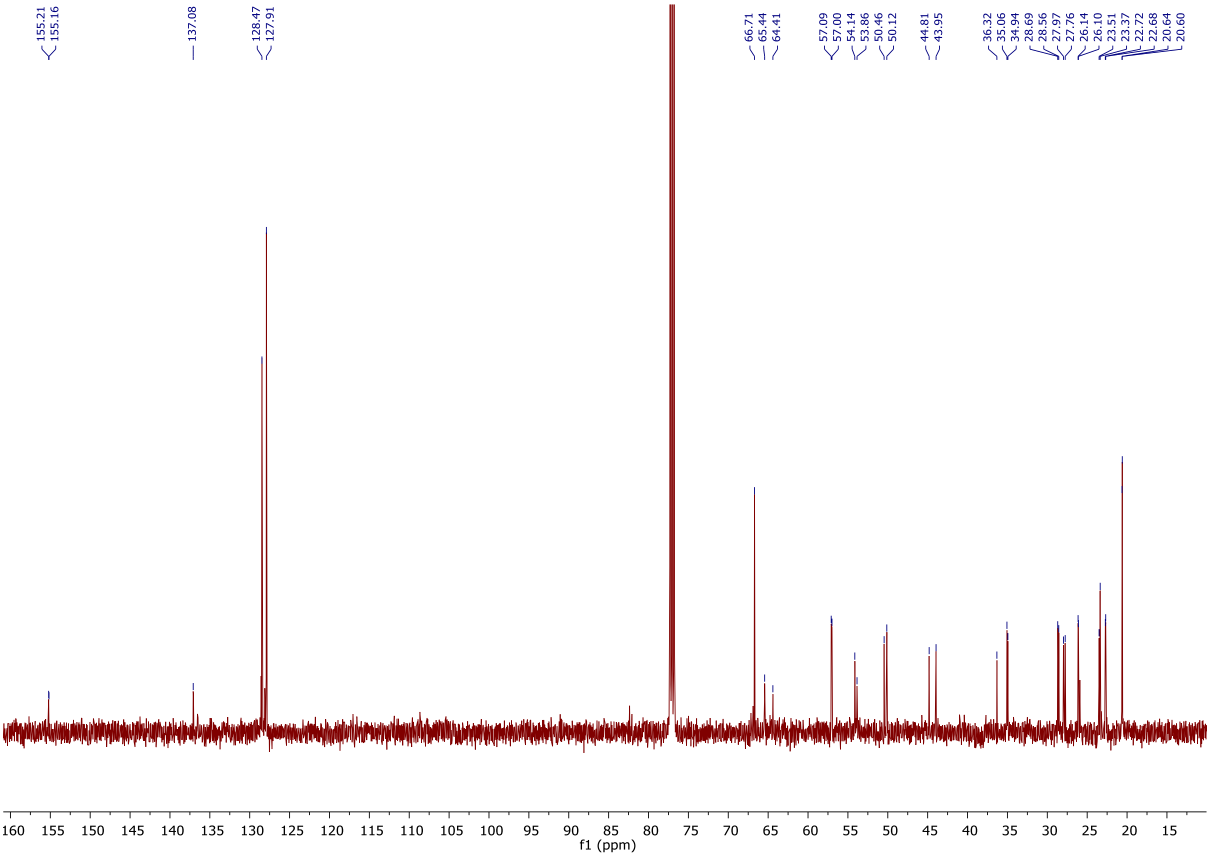

Benzyl 3a-(*iso*butylamino)octahydro-2*H*-isoindole-1-carboxylate (**6j**)

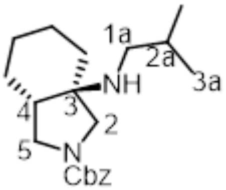

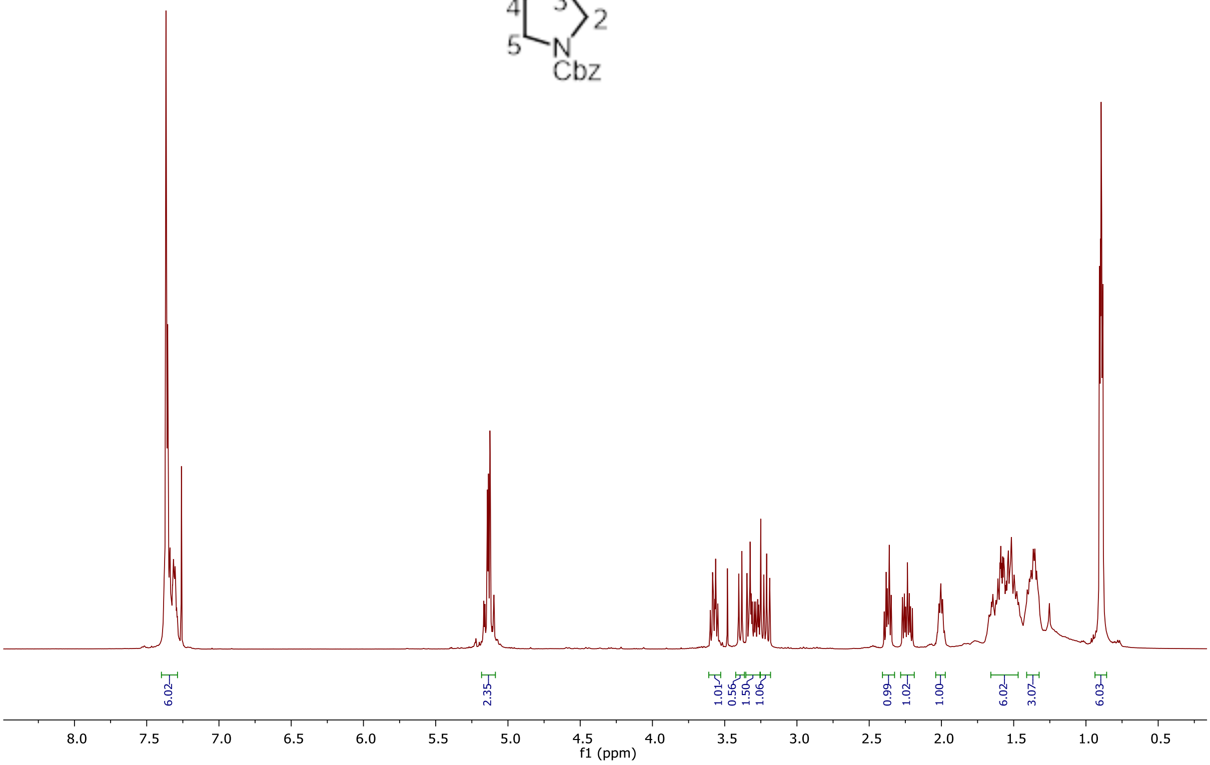

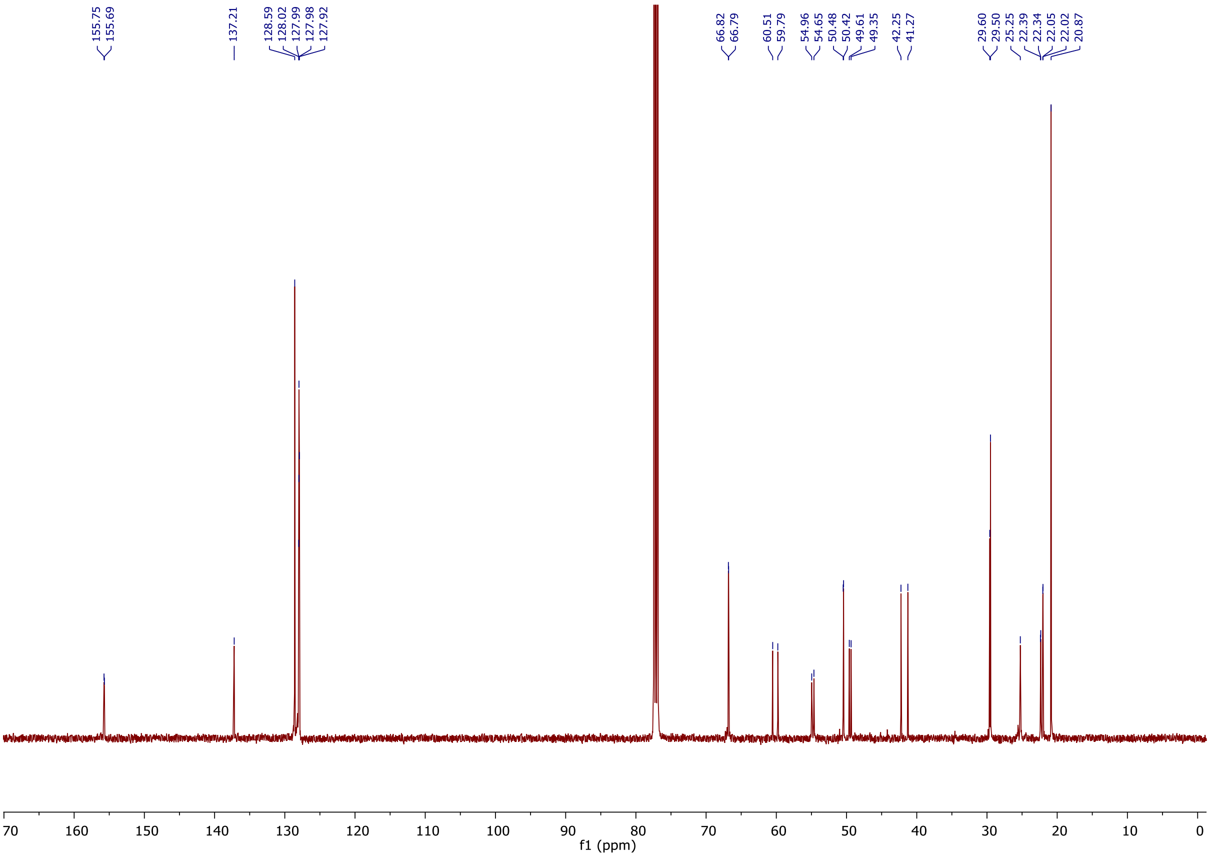

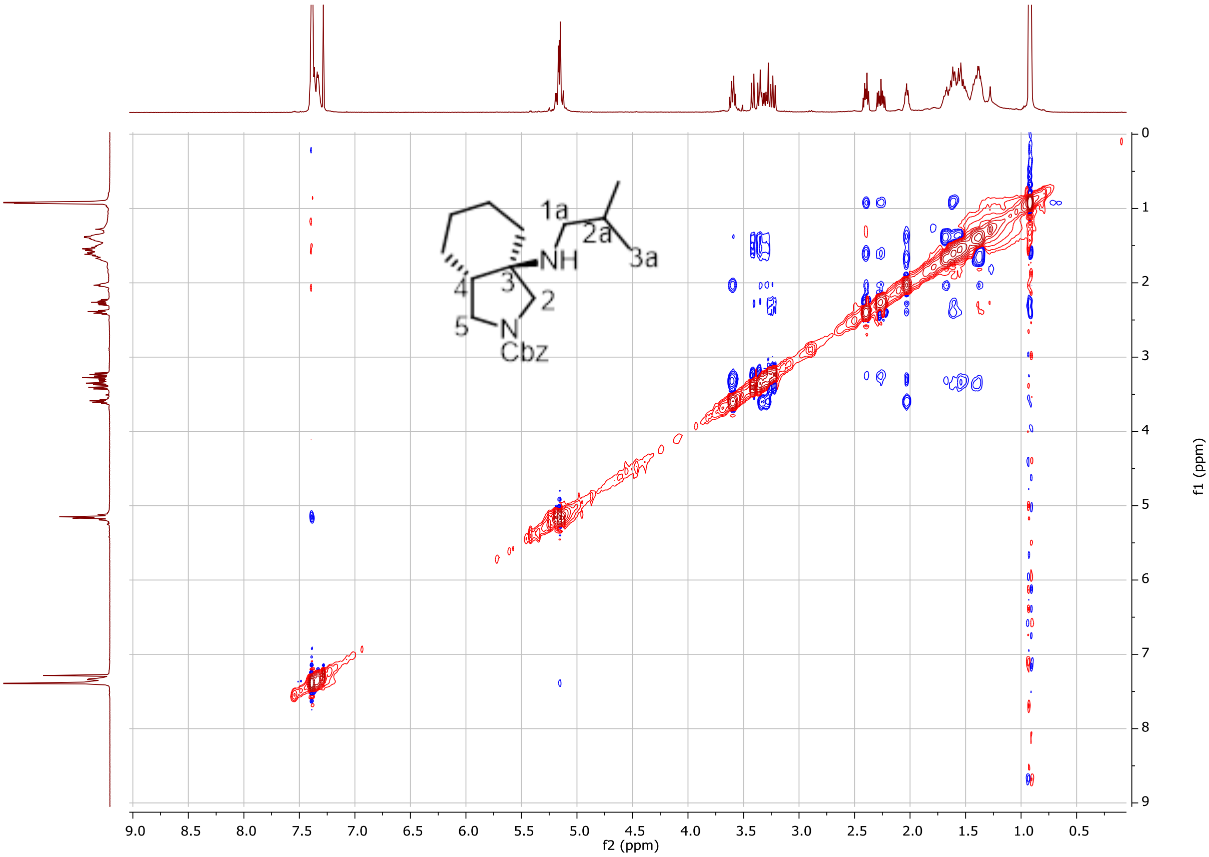
1a
2a
NH
3a
4
3
2
5
N
Cbz
f2 (ppm)
f1 (ppm)

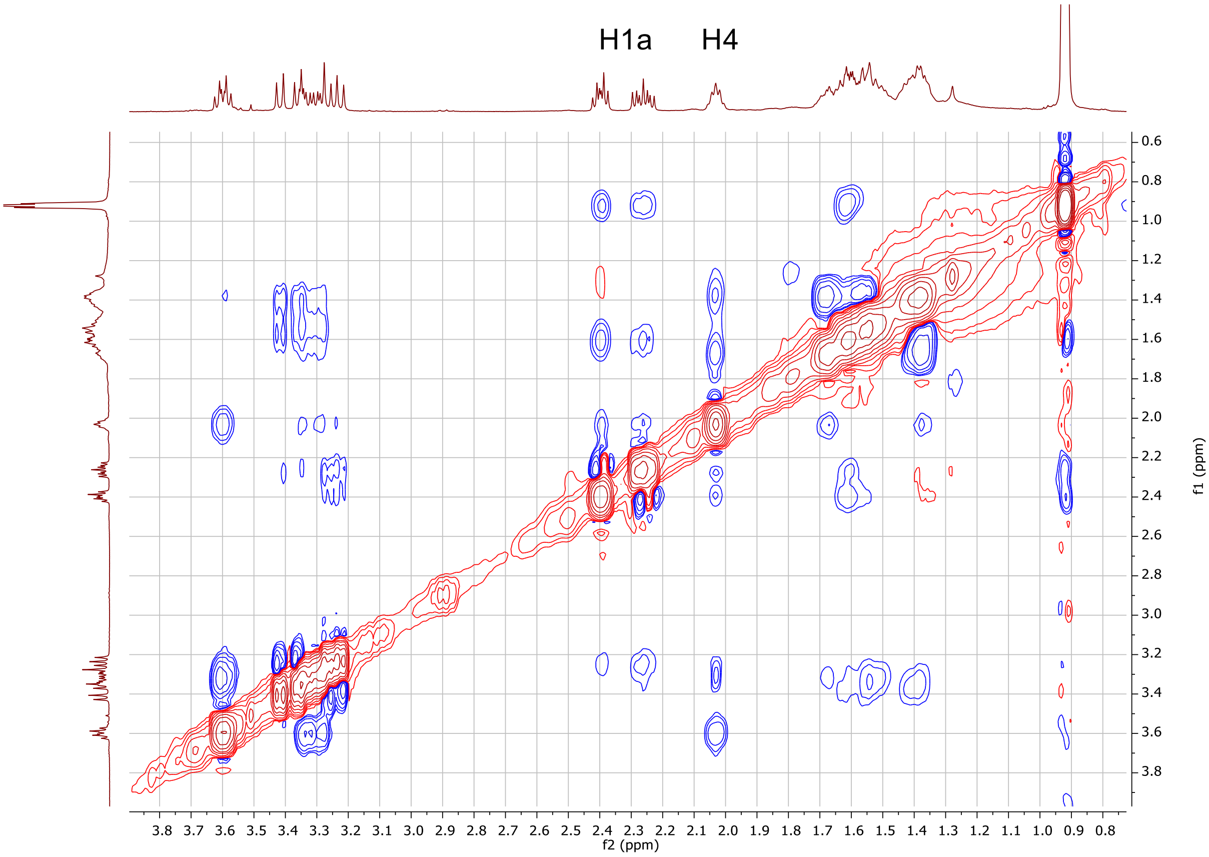
H1a
H4
f2 (ppm)
f1 (ppm)

Benzyl 3-(methylamino)pyrrolidine-1-carboxylate (**6k**)

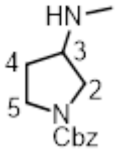

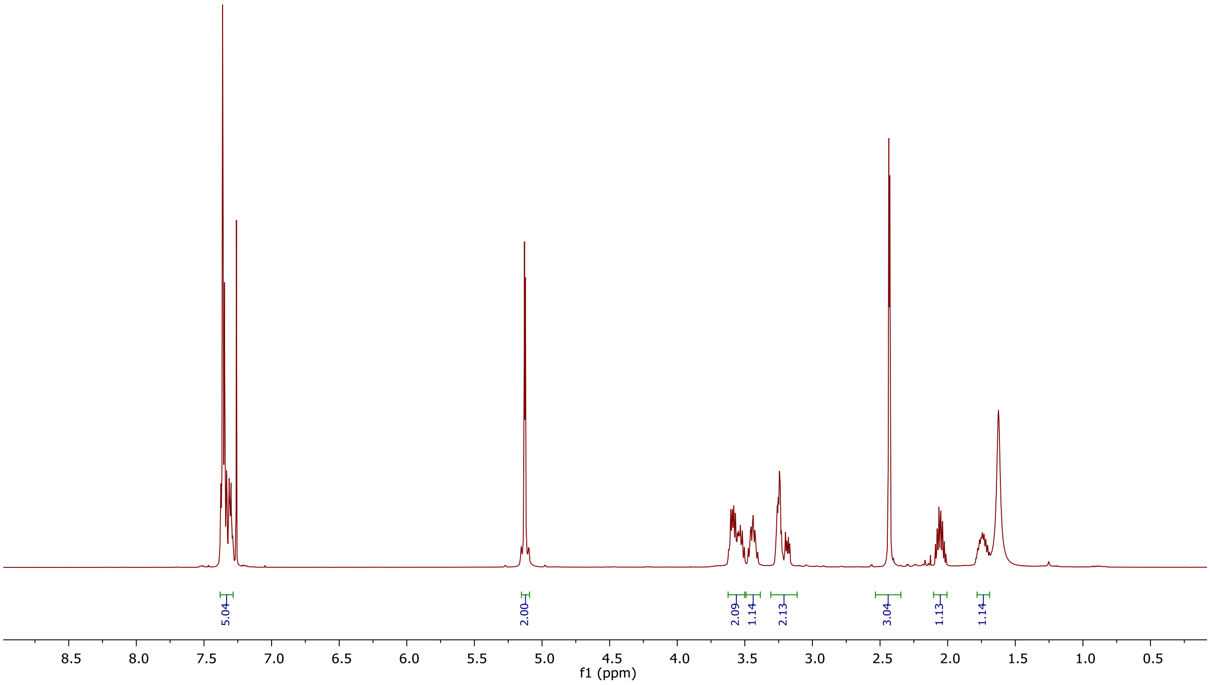

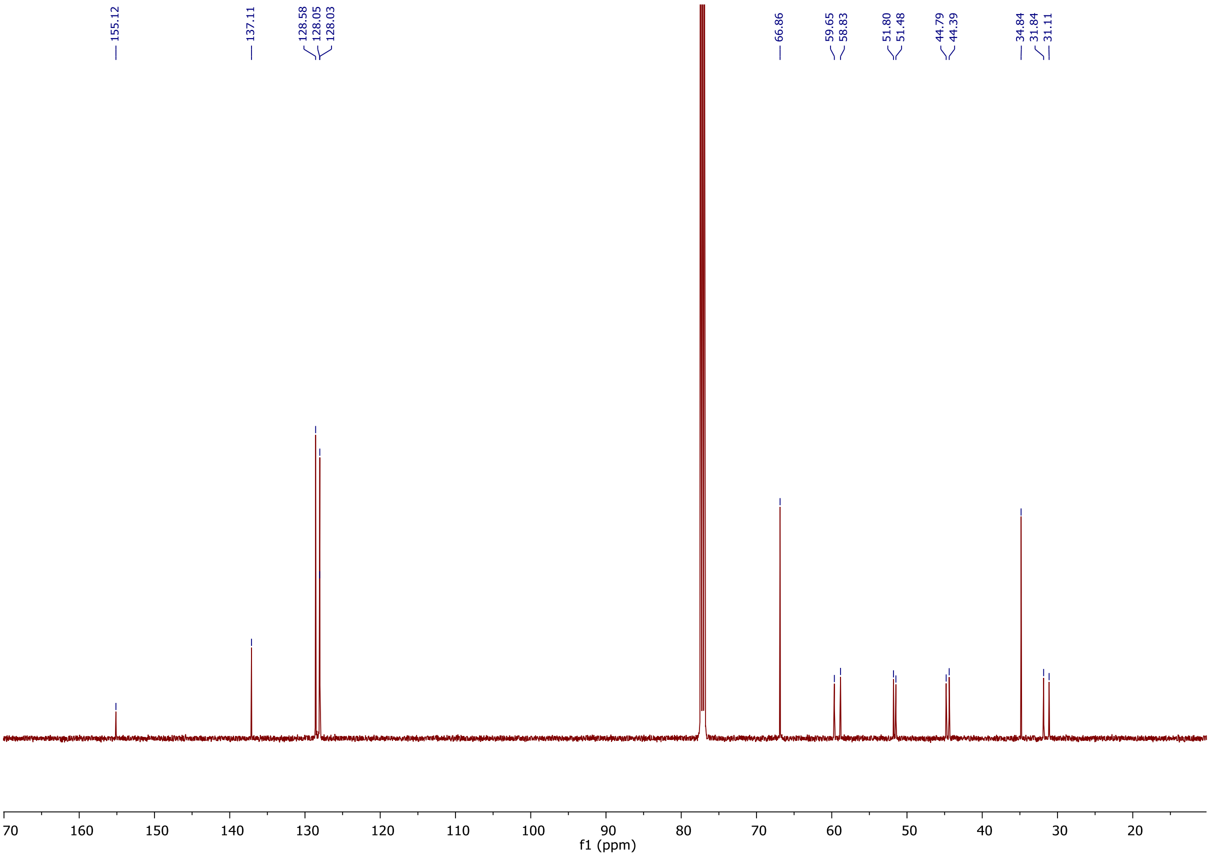

Benzyl 3-(butylamino)pyrrolidine-1-carboxylate (**6l**)

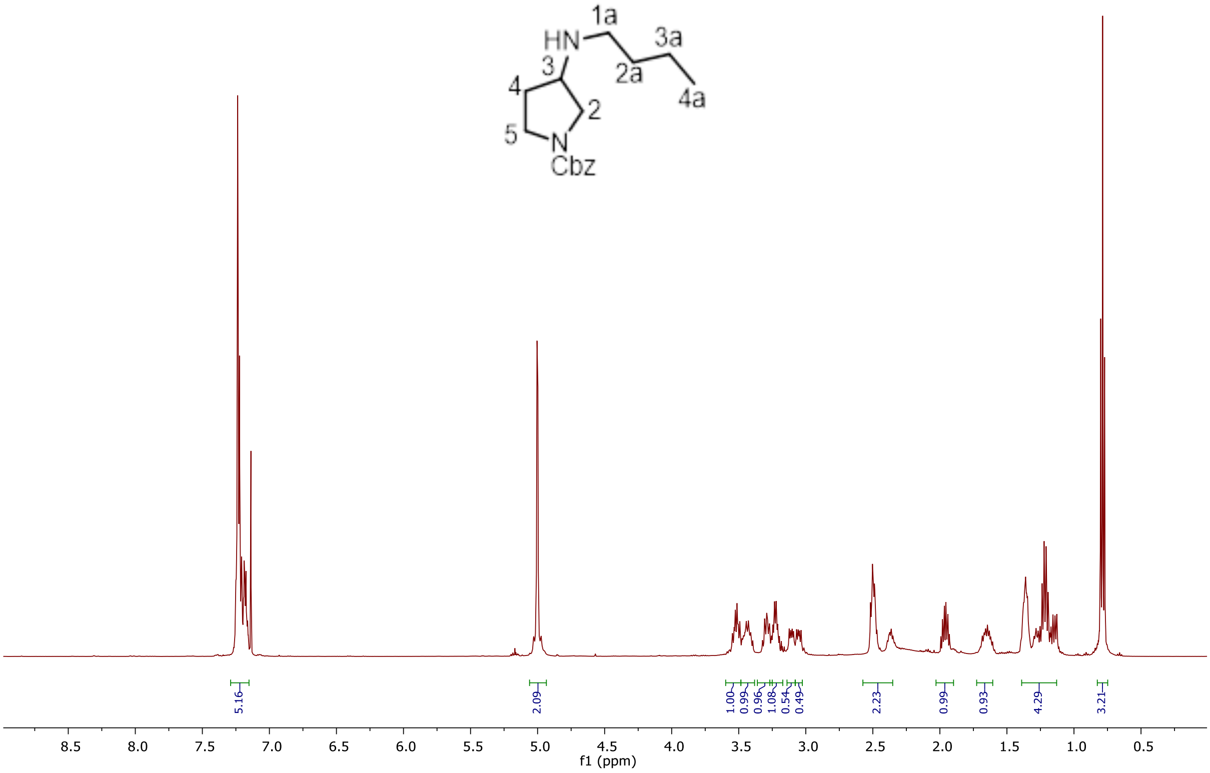

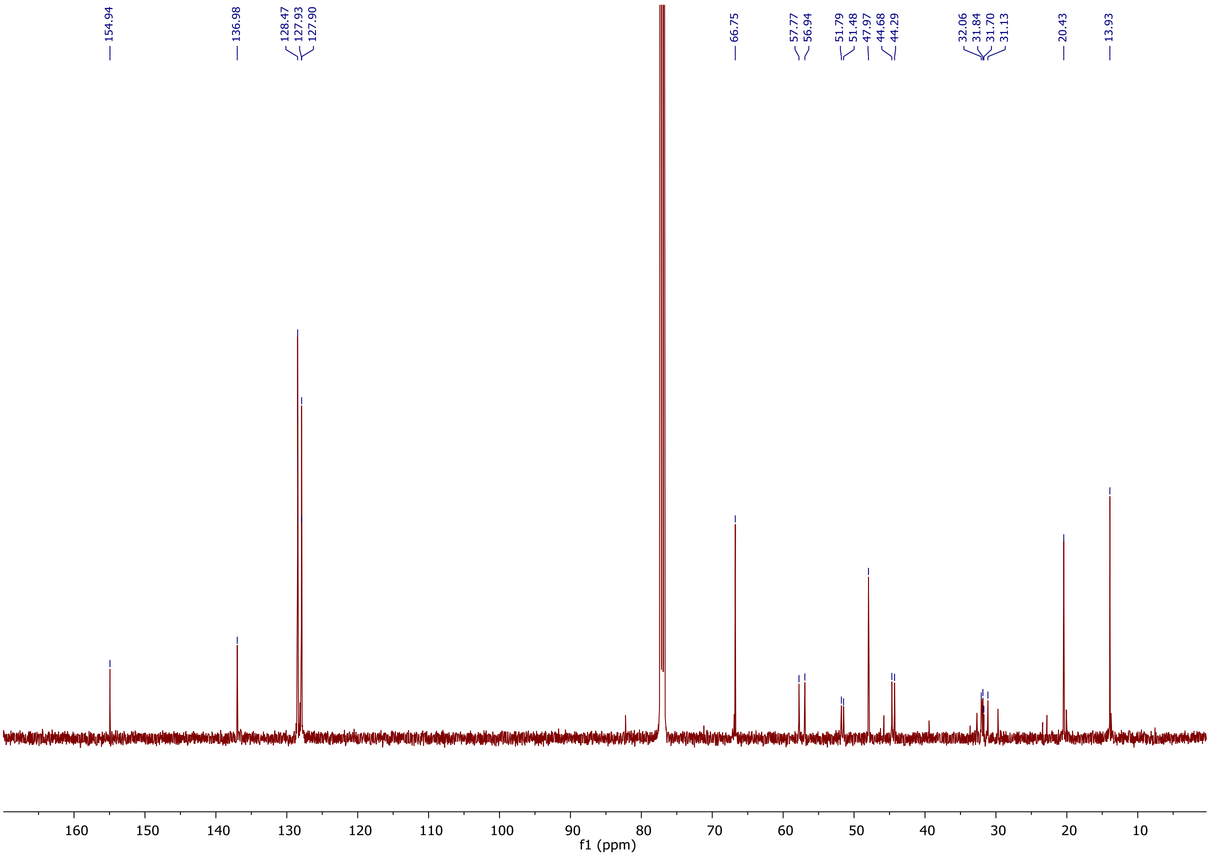

Benzyl 3-(benzylamino)pyrrolidine-1-carboxylate (**6m**)

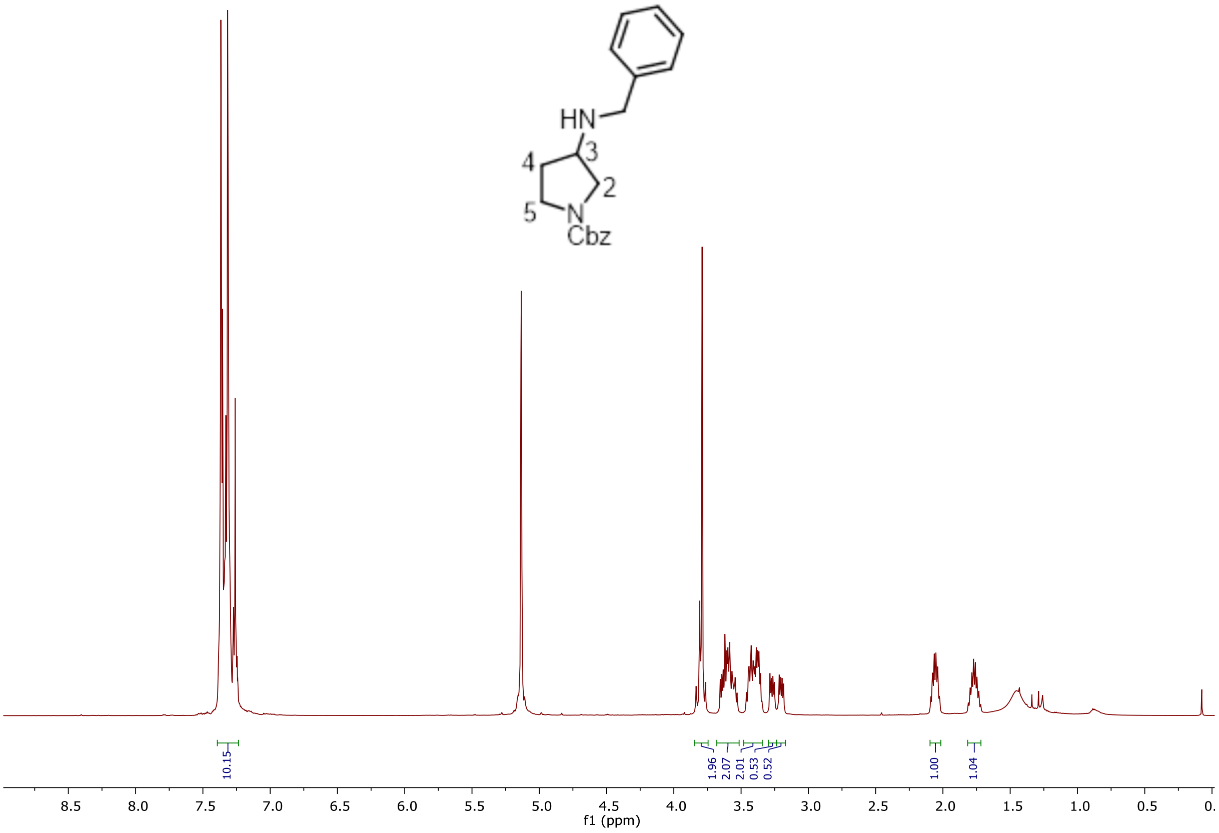

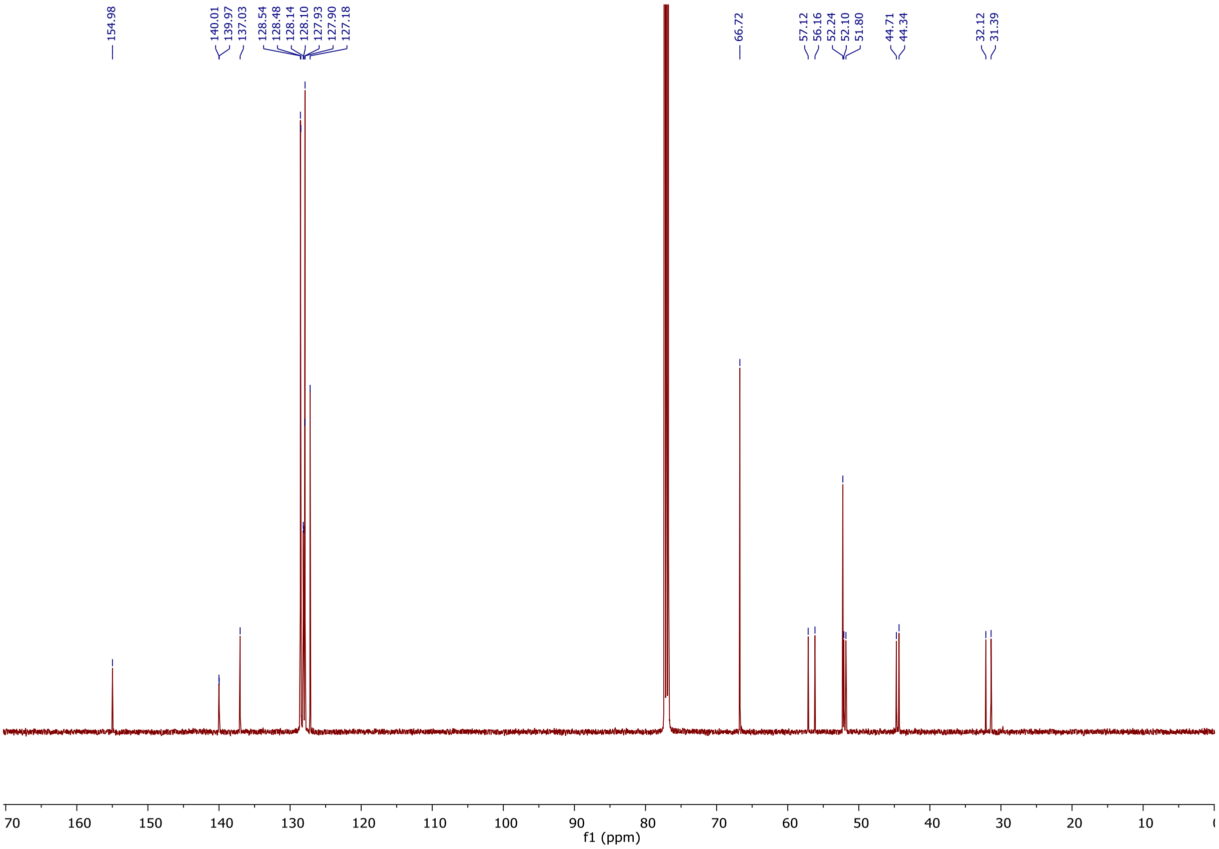

Benzyl 3-((furan-2-ylmethyl)amino)pyrrolidine-1-carboxylate (**6n**)

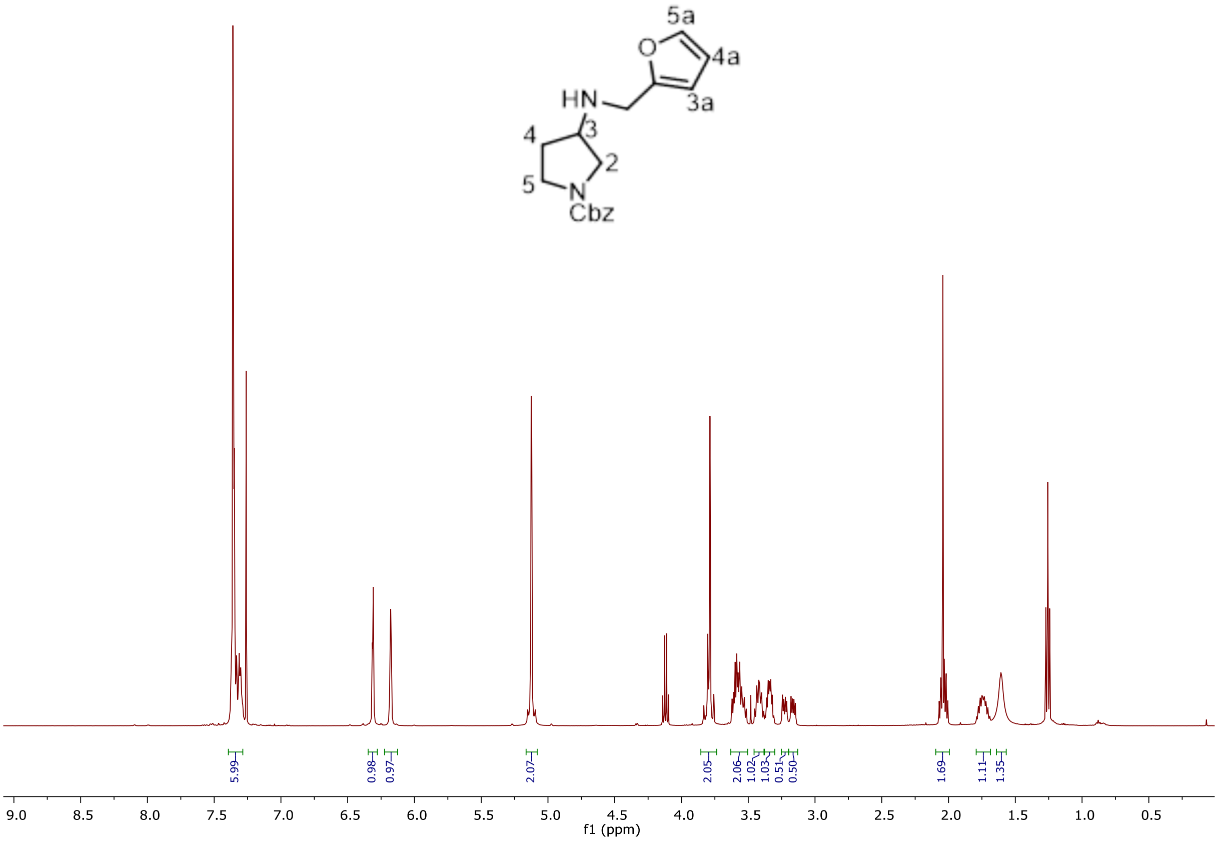

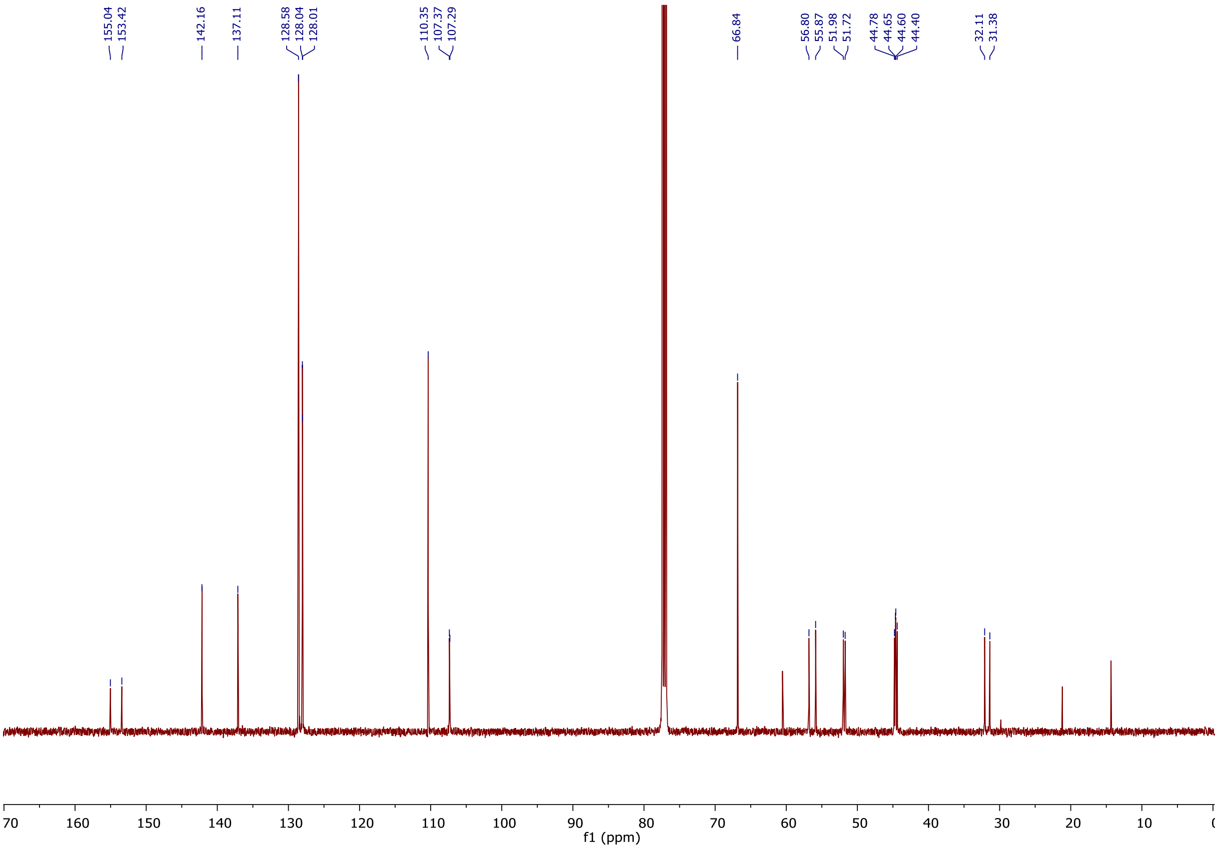

Benzyl 3-(*iso*propylamino)pyrrolidine-1-carboxylate (**6o**)

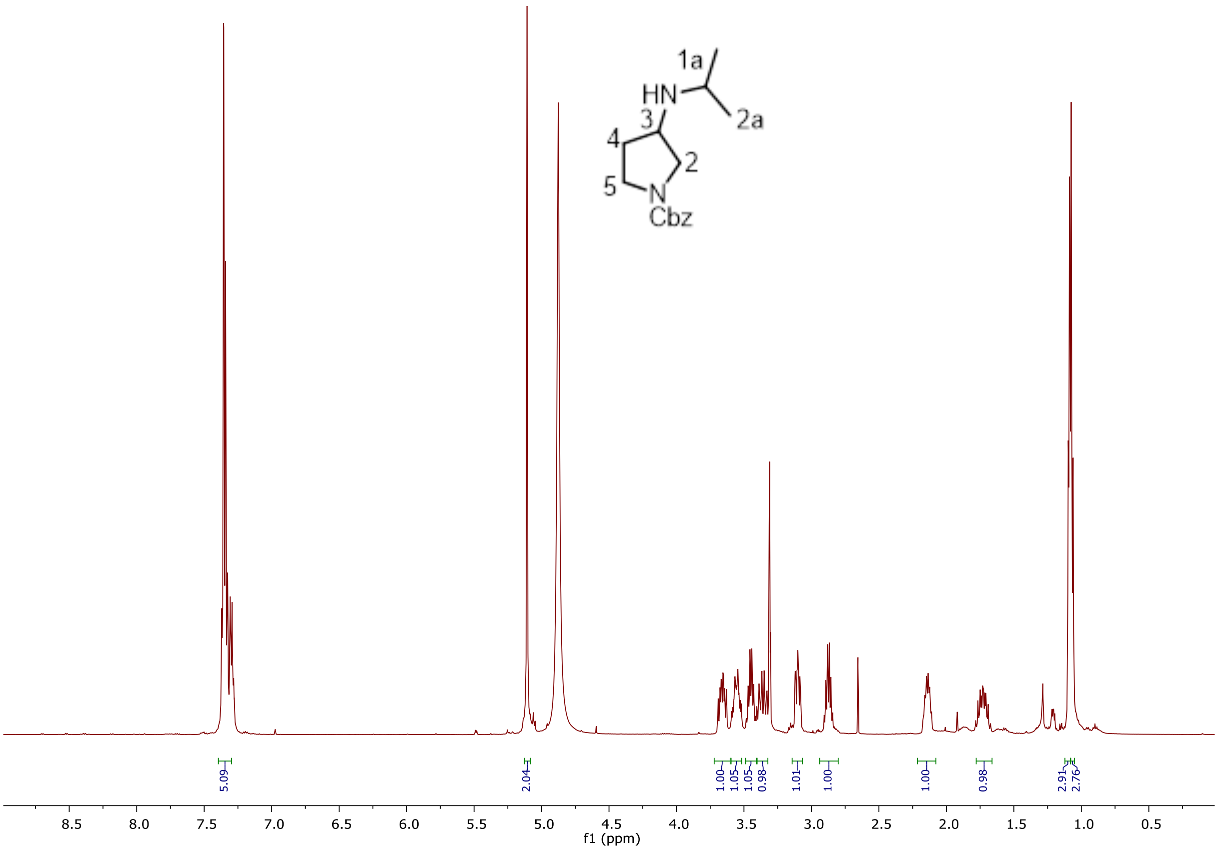

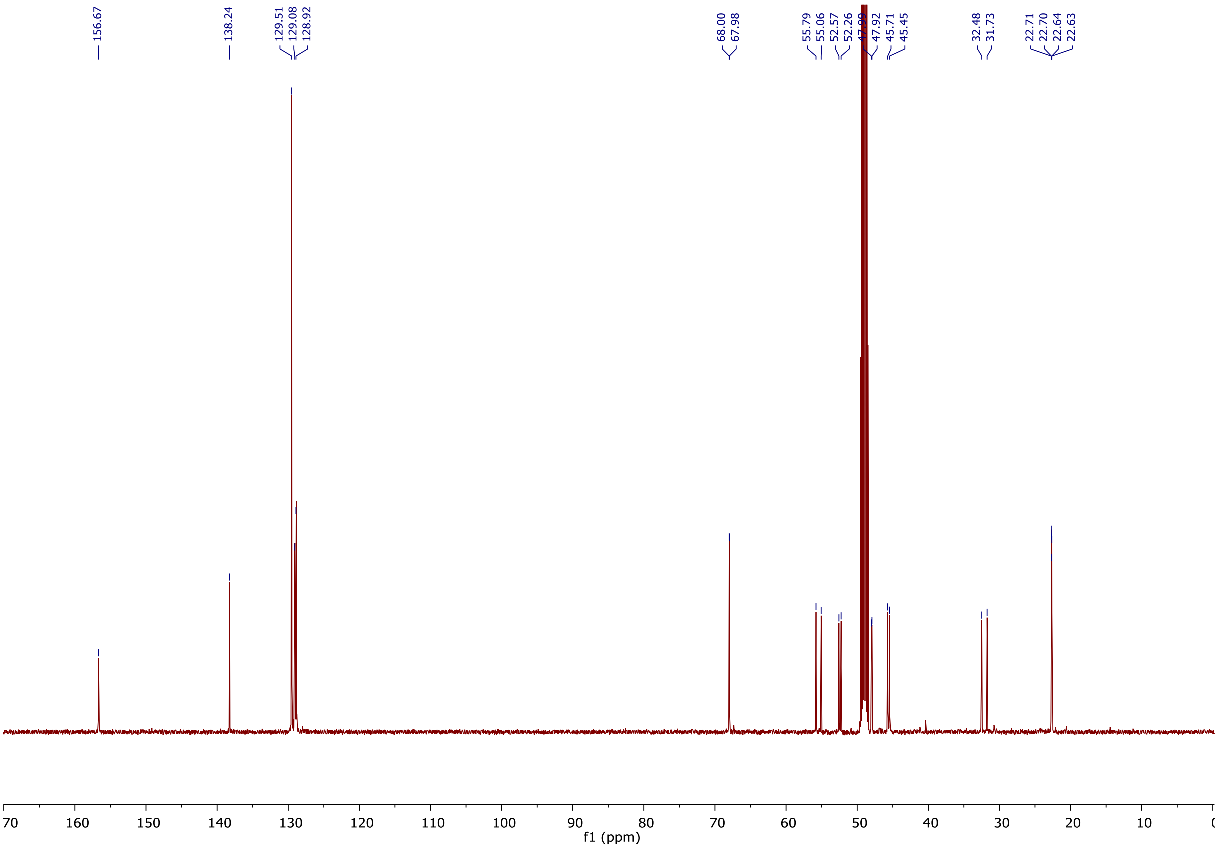

Benzyl 3-((3-ethoxy-3-oxopropyl)amino)pyrrolidine-1-carboxylate (**6p**)

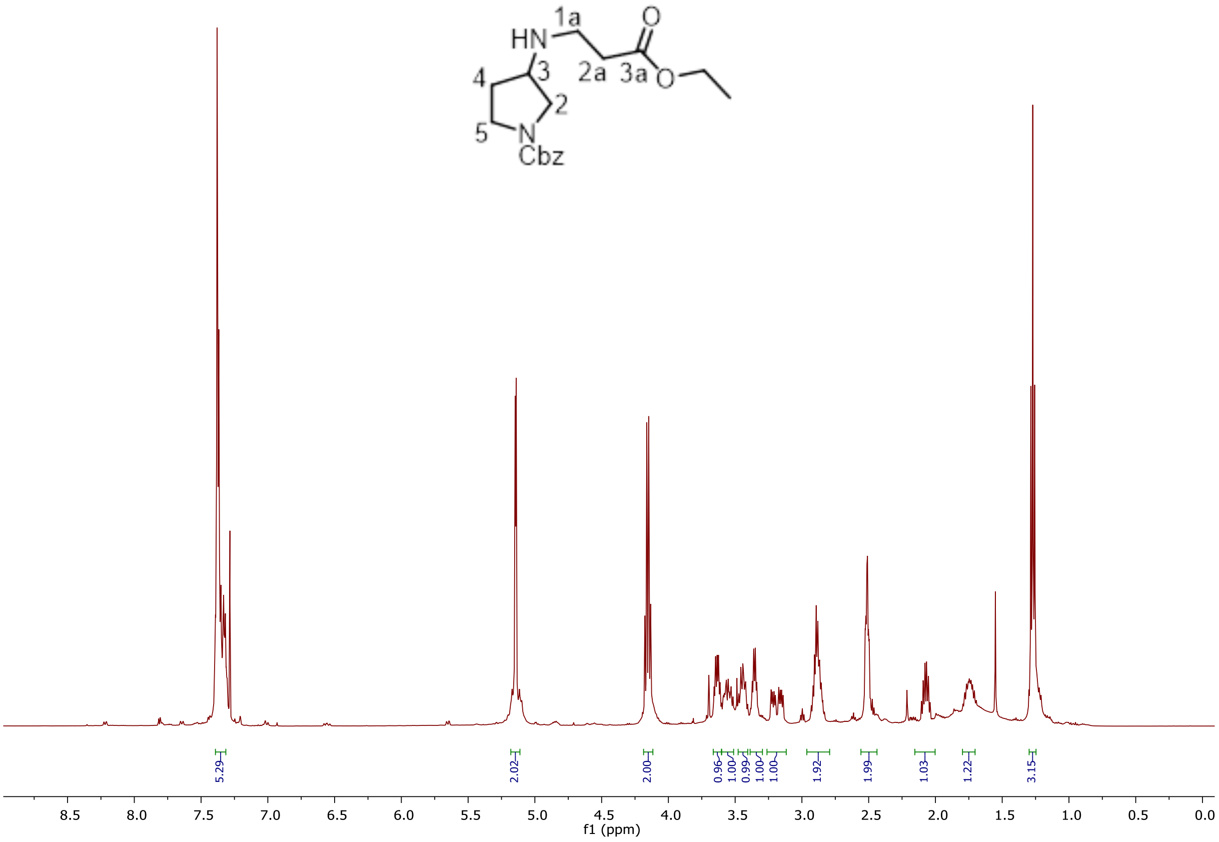

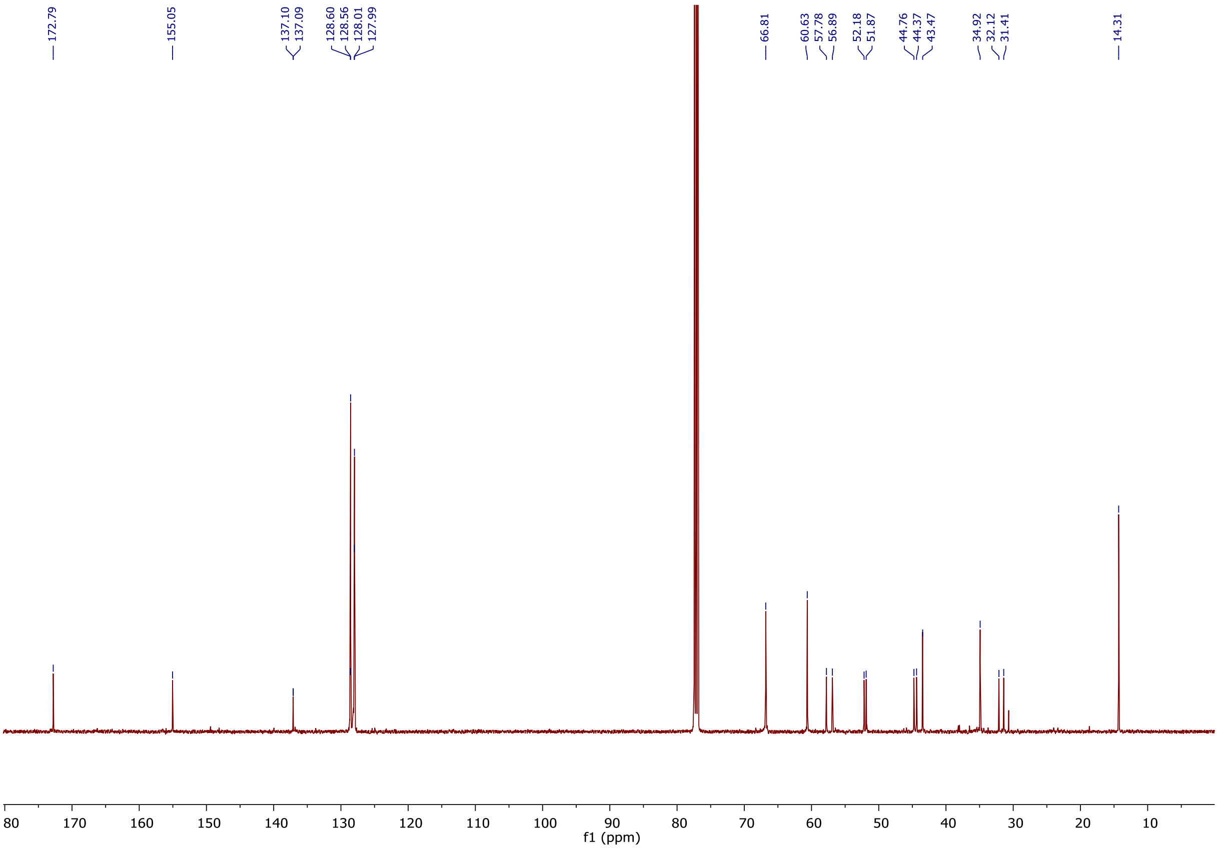

Benzyl 3-((2-ethoxy-2-oxoethyl)amino)pyrrolidine-1-carboxylate (**6q**)

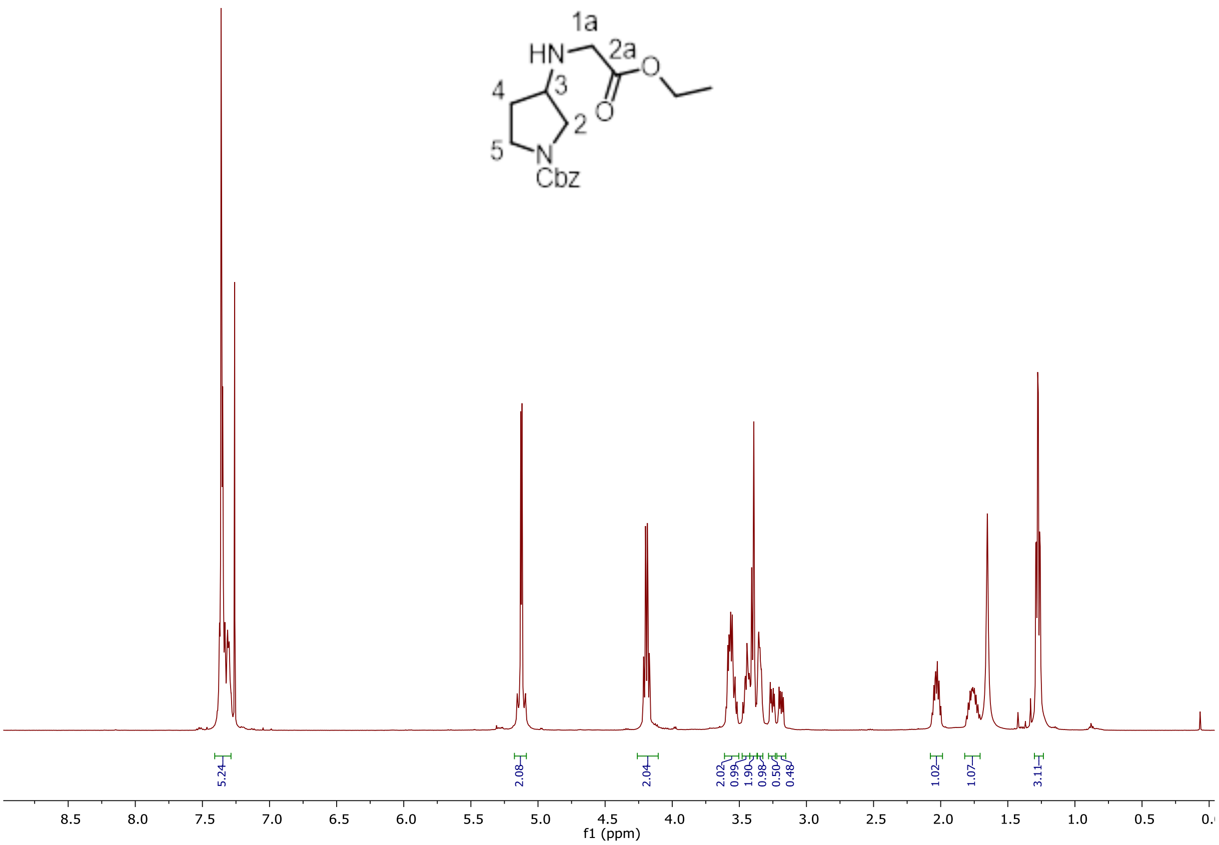

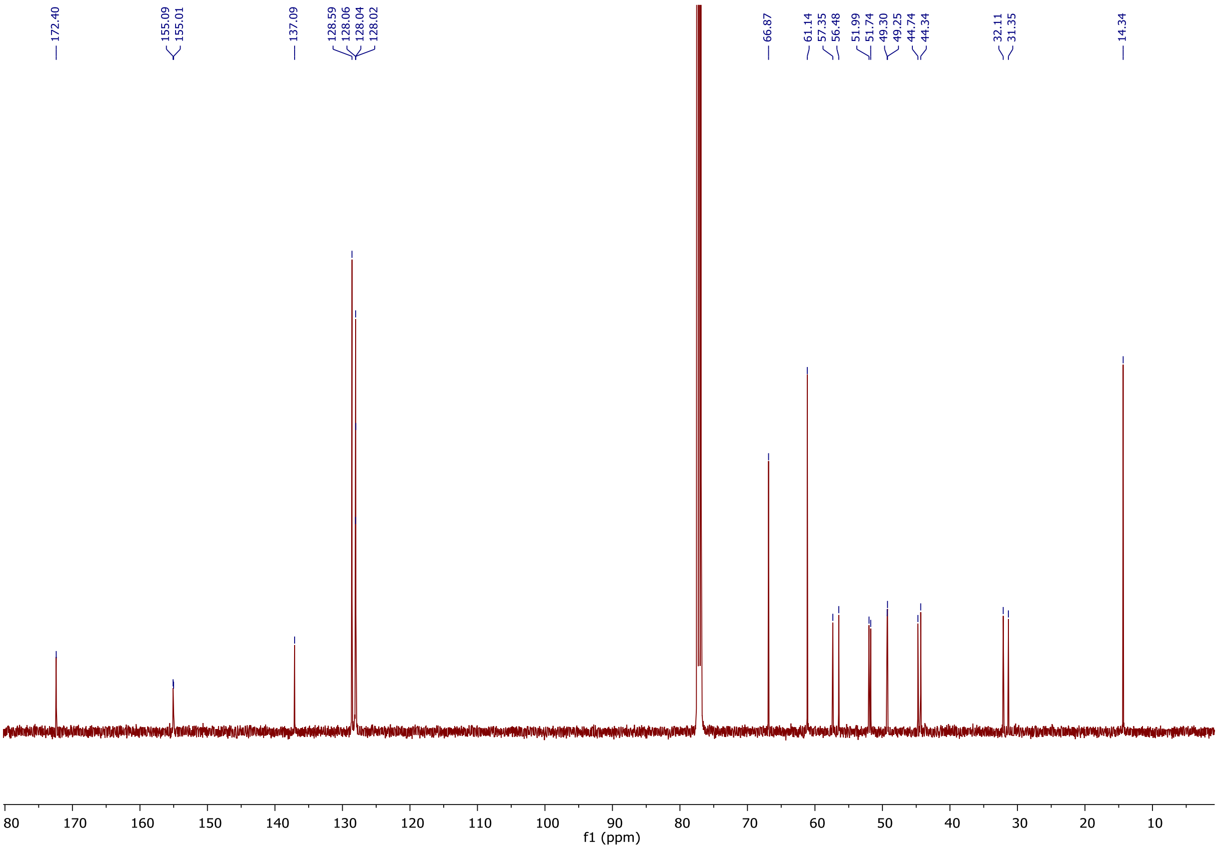

Benzyl 3-(((S)-1-ethoxy-1-oxo-3-phenylpropan-2-yl)amino)pyrrolidine-1-carboxylate (**6r**)

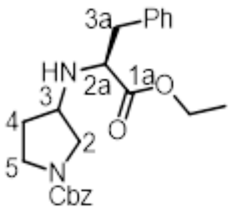

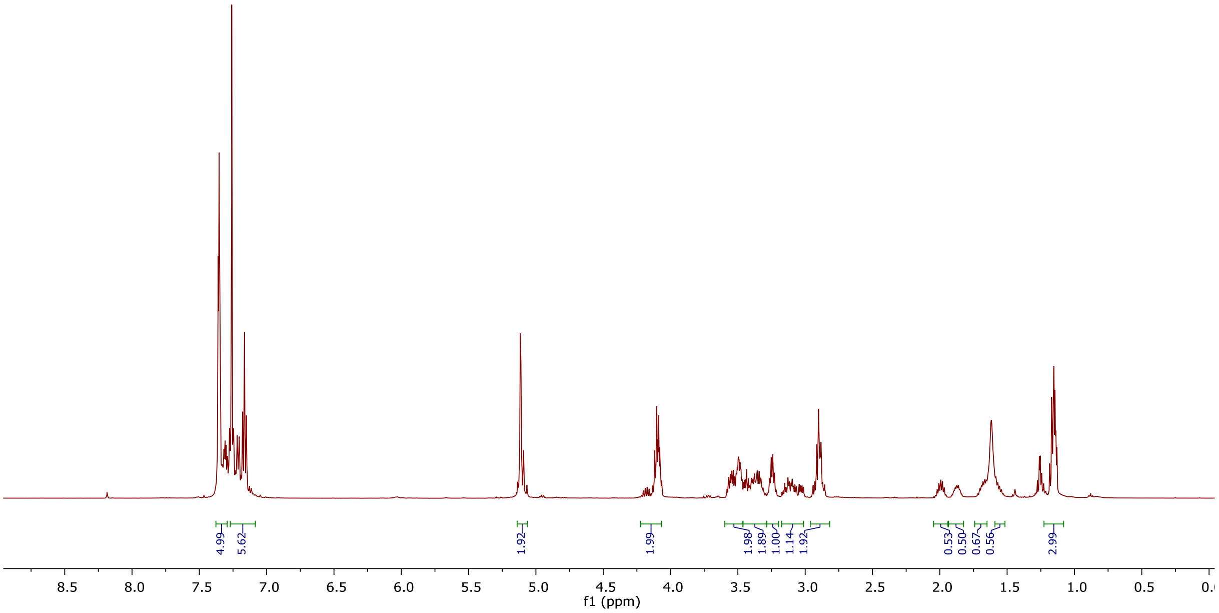

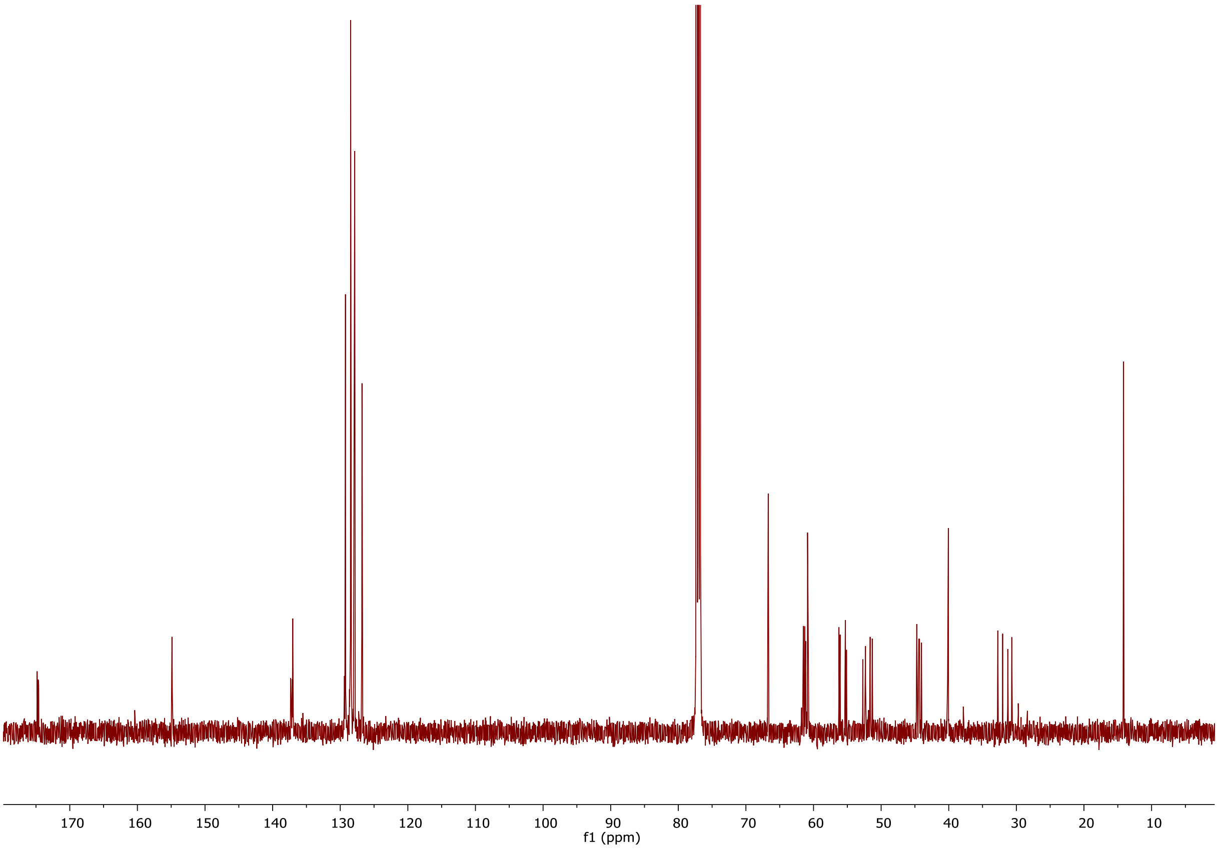

Benzyl 3-((2-((*tert*-butoxycarbonyl)amino)ethyl)amino)pyrrolidine-1-carboxylate (**6s**)

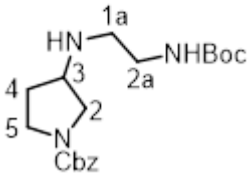

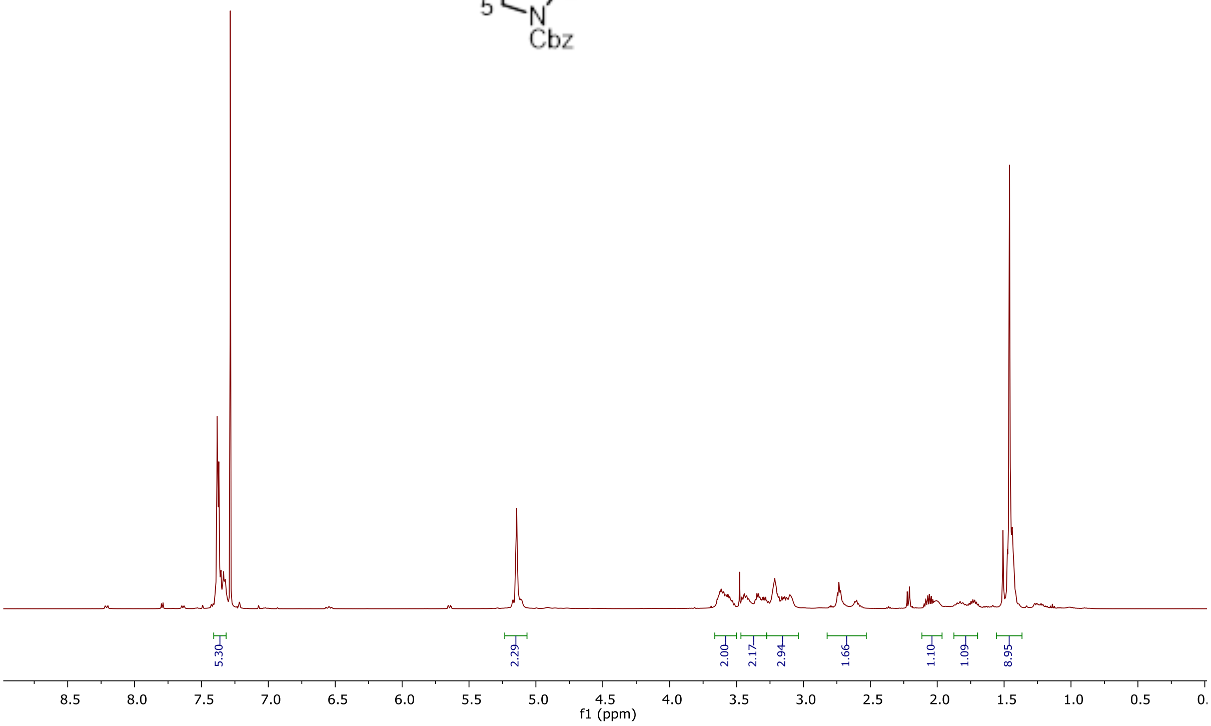

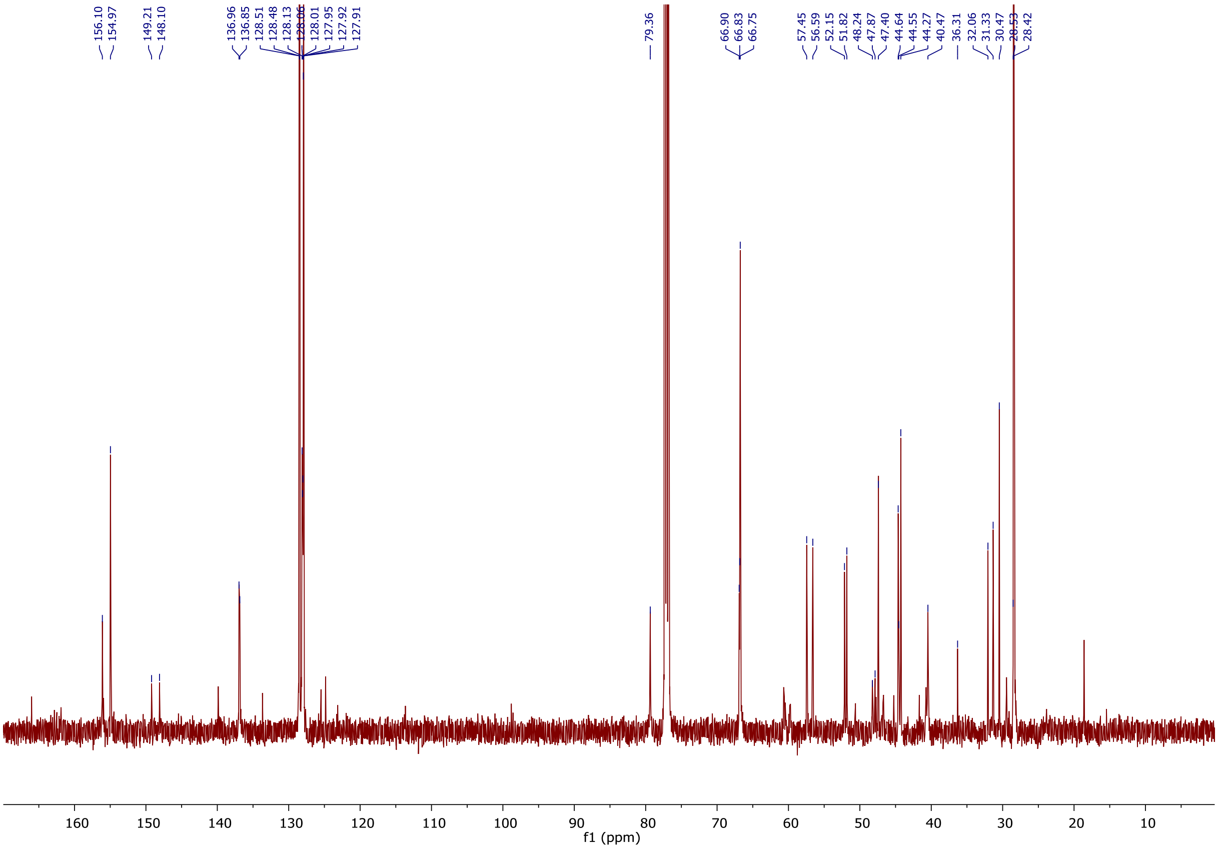

Benzyl 3-aminopyrrolidine-1-carboxylate (**7a**)

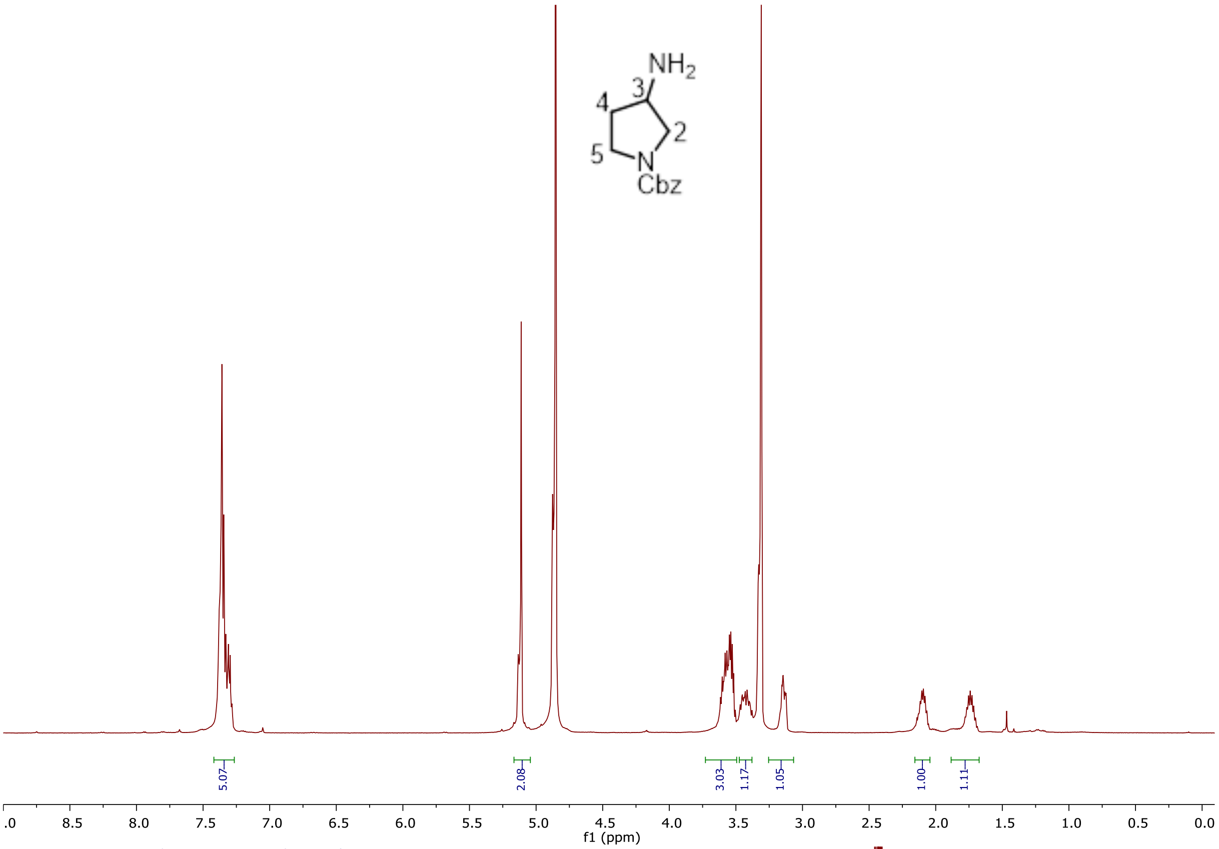

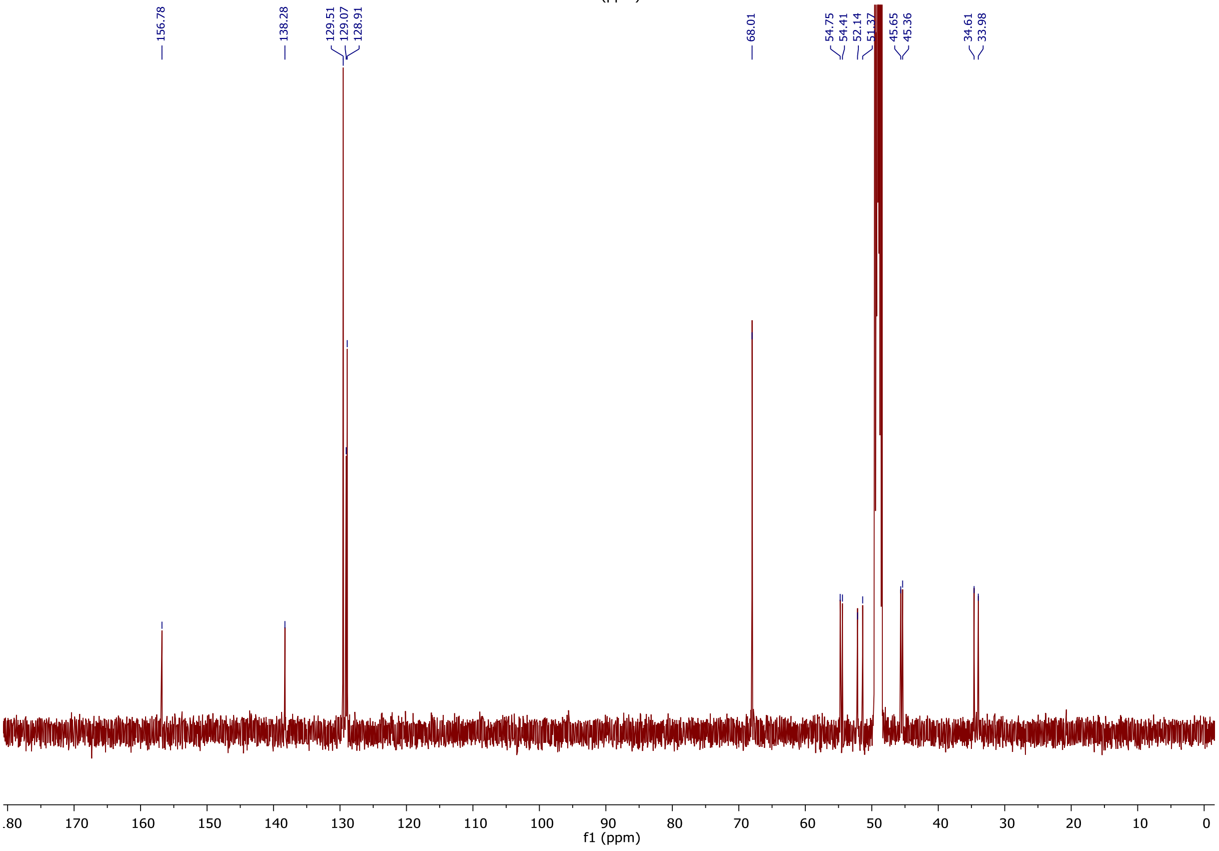

Benzyl 3-aminopiperidine-1-carboxylate (**7b**)

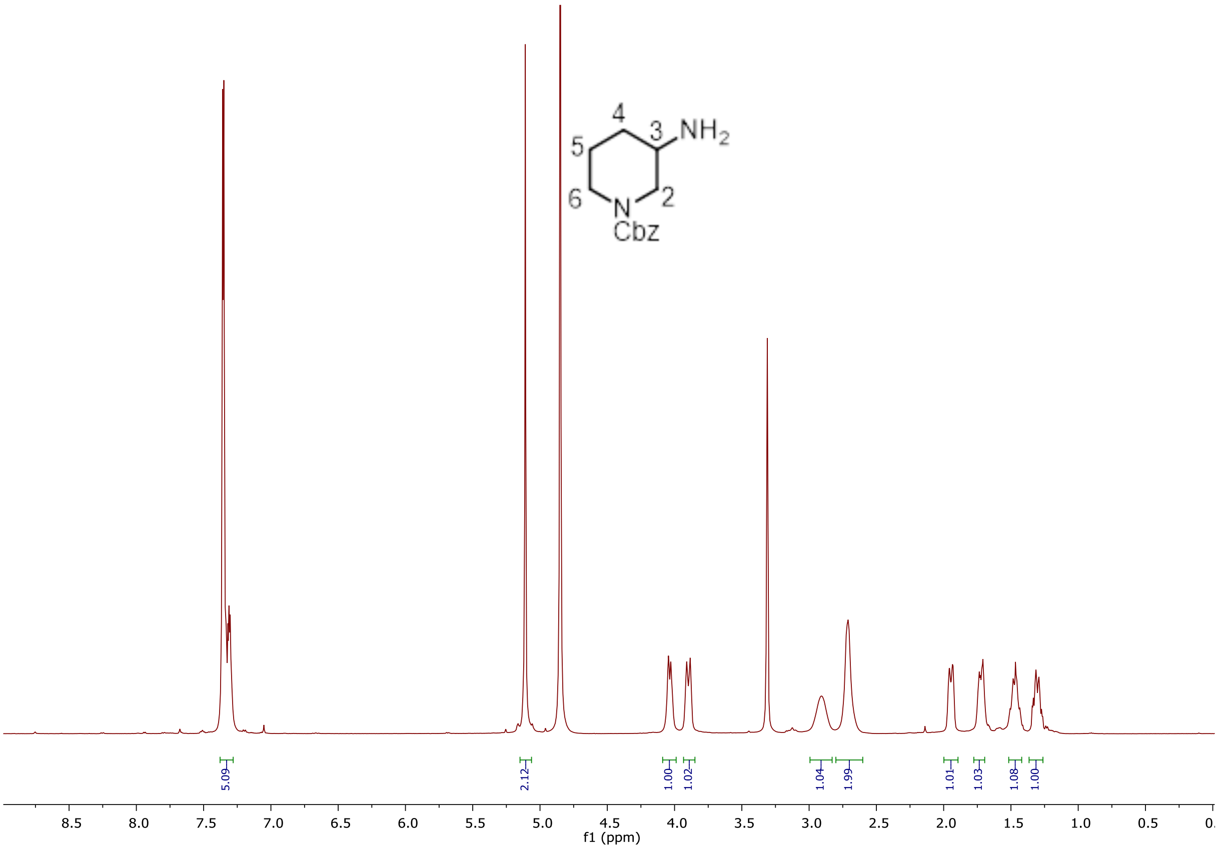

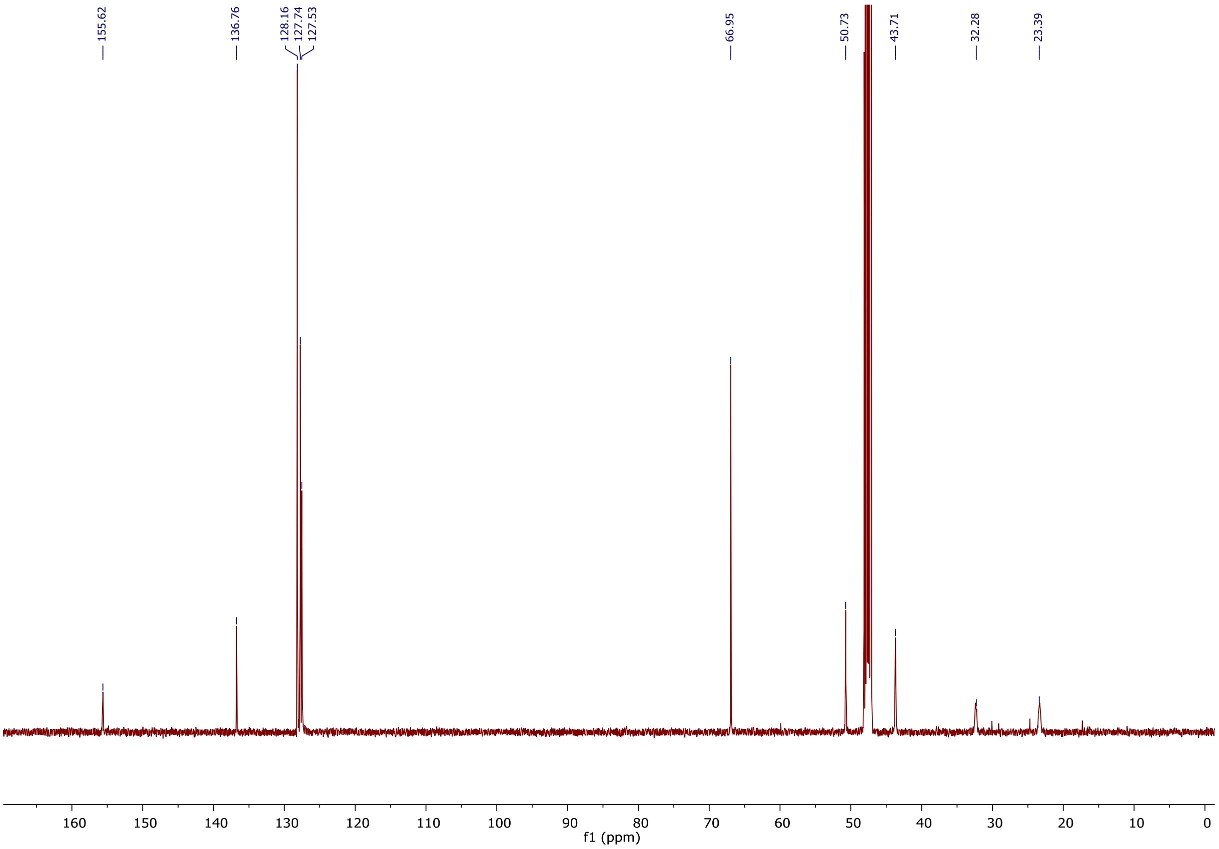

Benzyl 3-aminoazepane-1-carboxylate (**7c**)

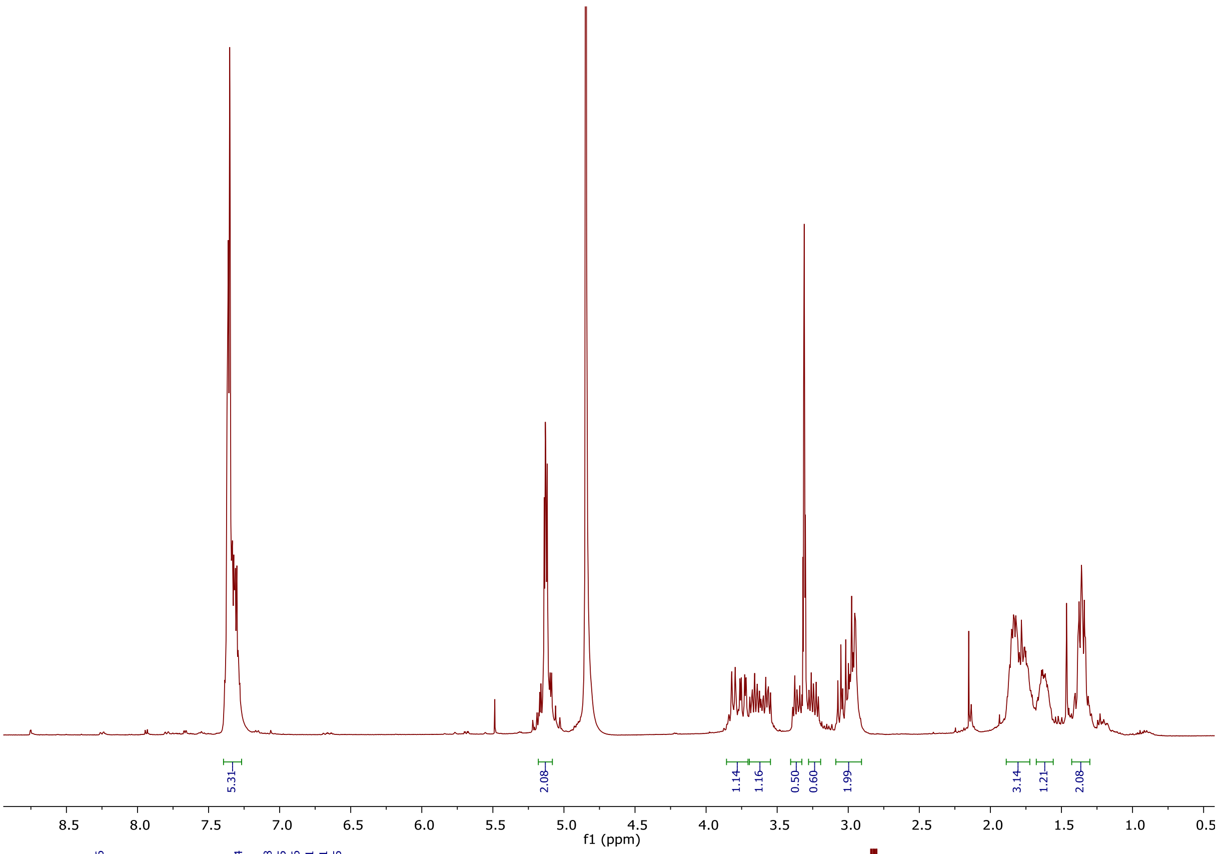

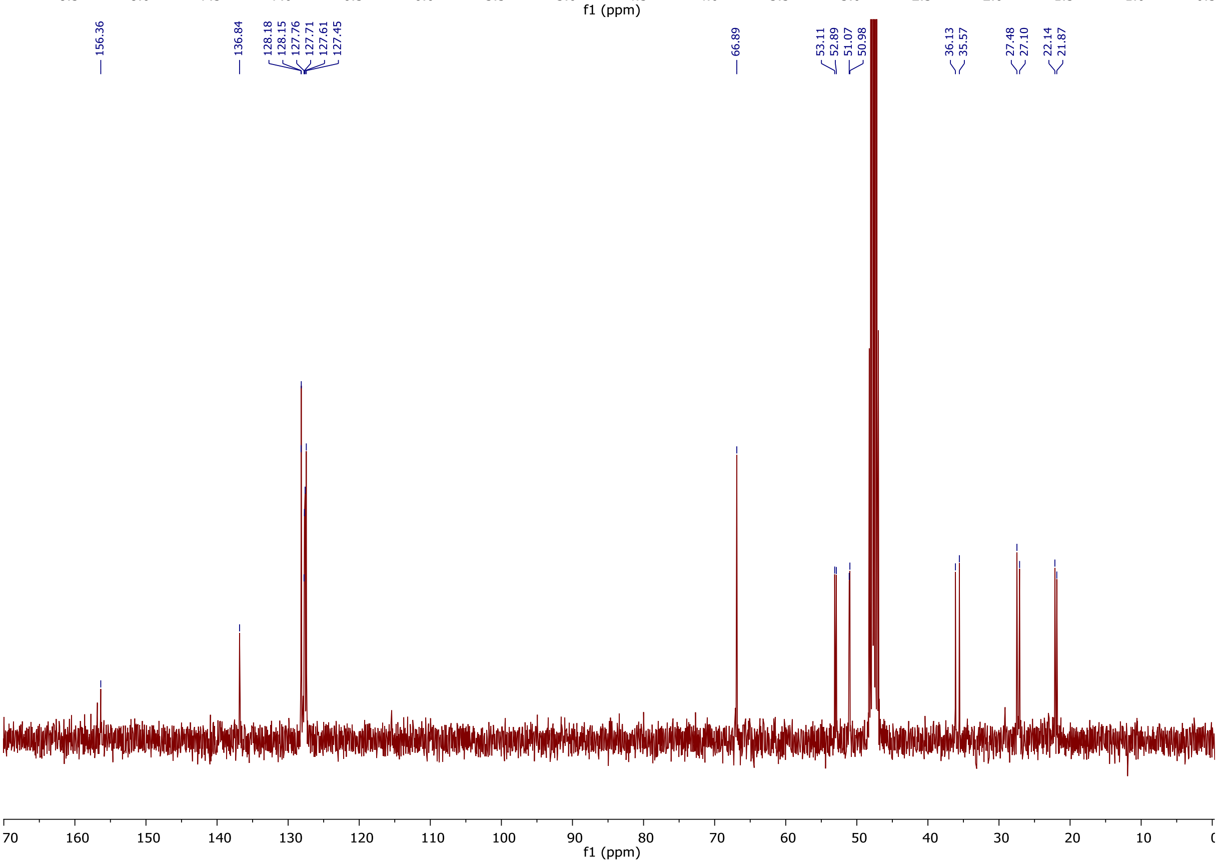

5-Amino-1-benzylpiperidin-2-one (**7d**)

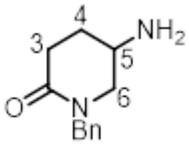

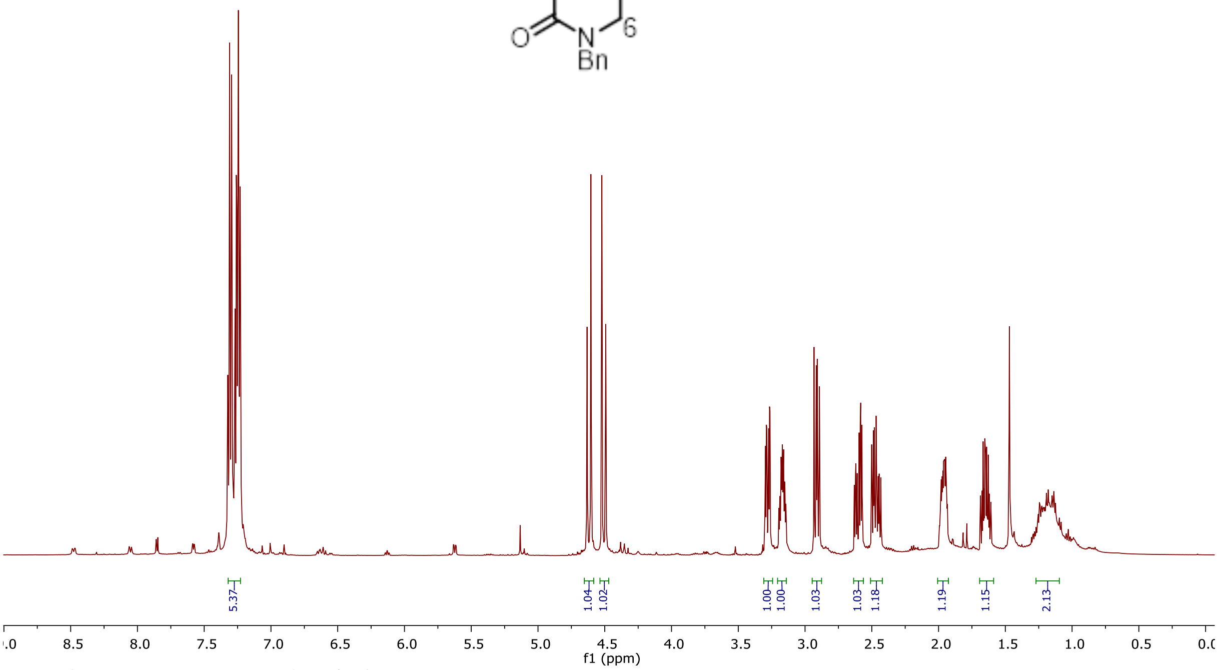

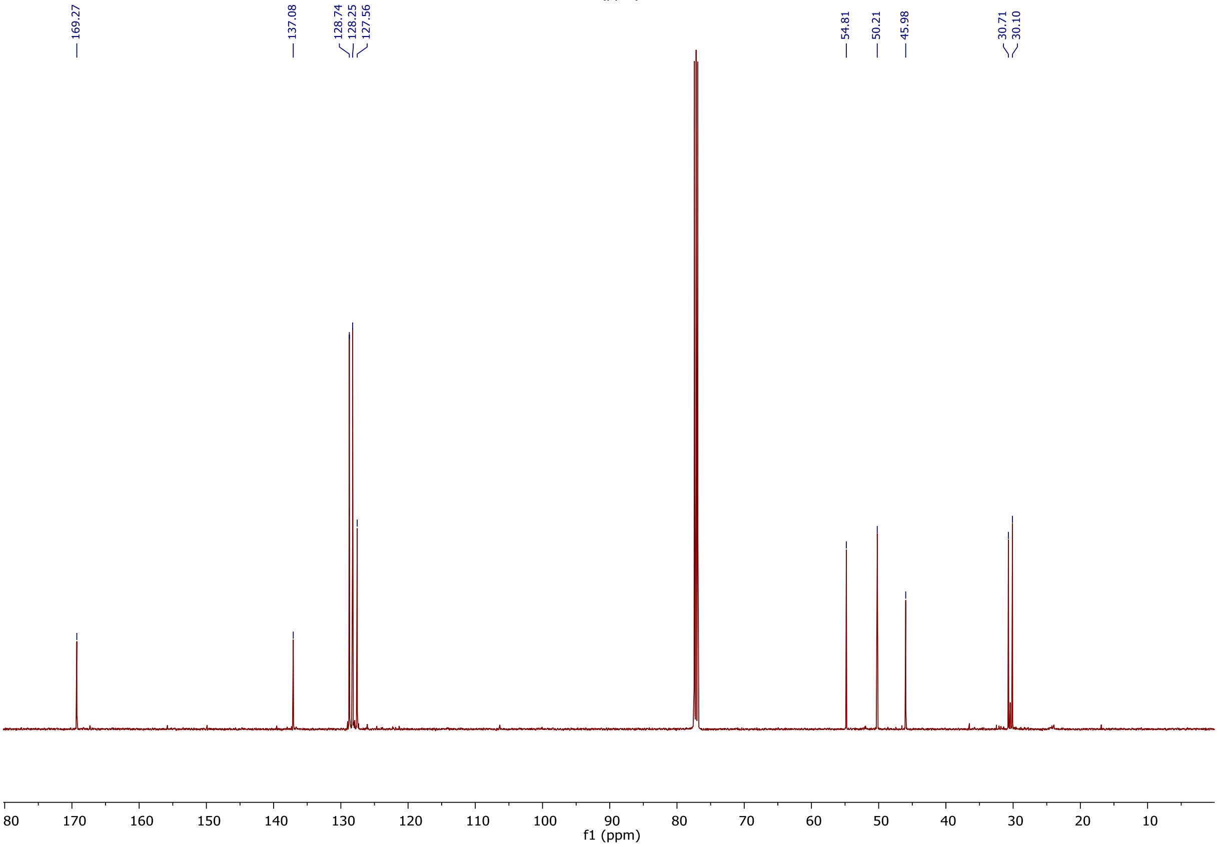

Benzyl 4-amino-2,2-dimethylpyrrolidine-1-carboxylate (**7e**)

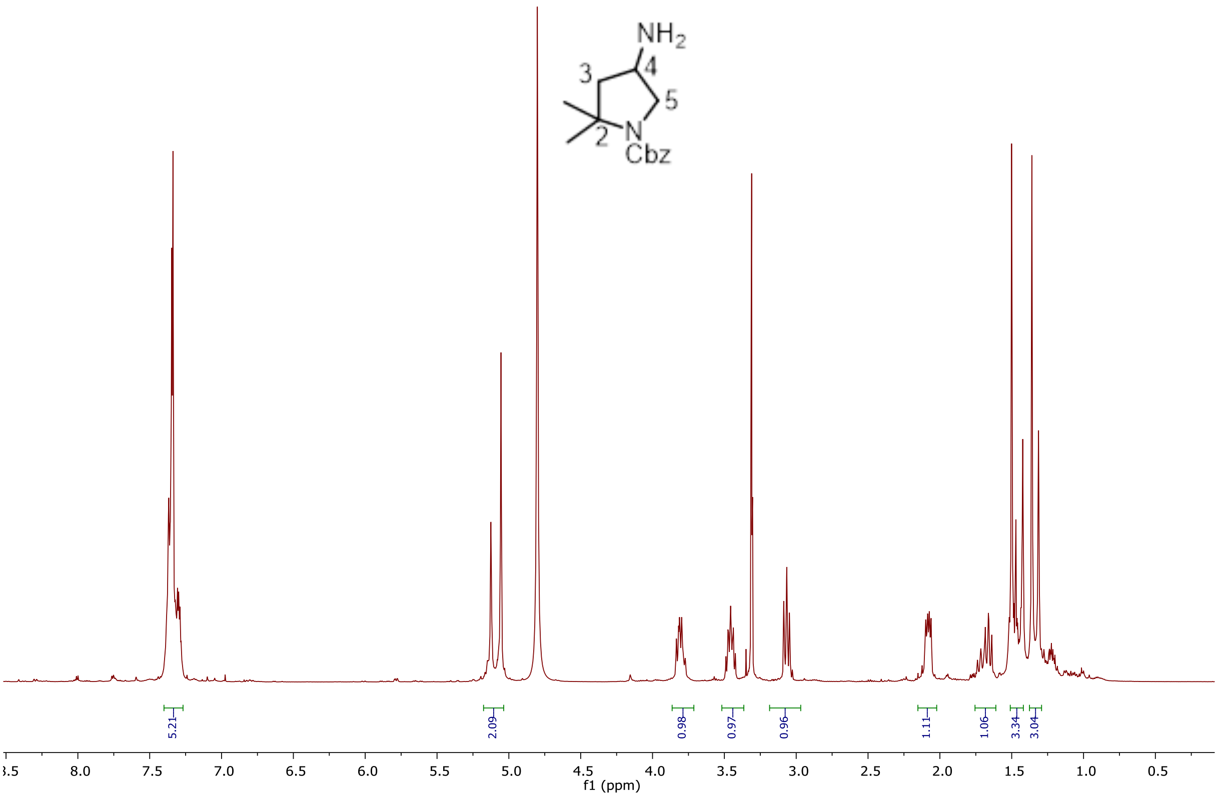

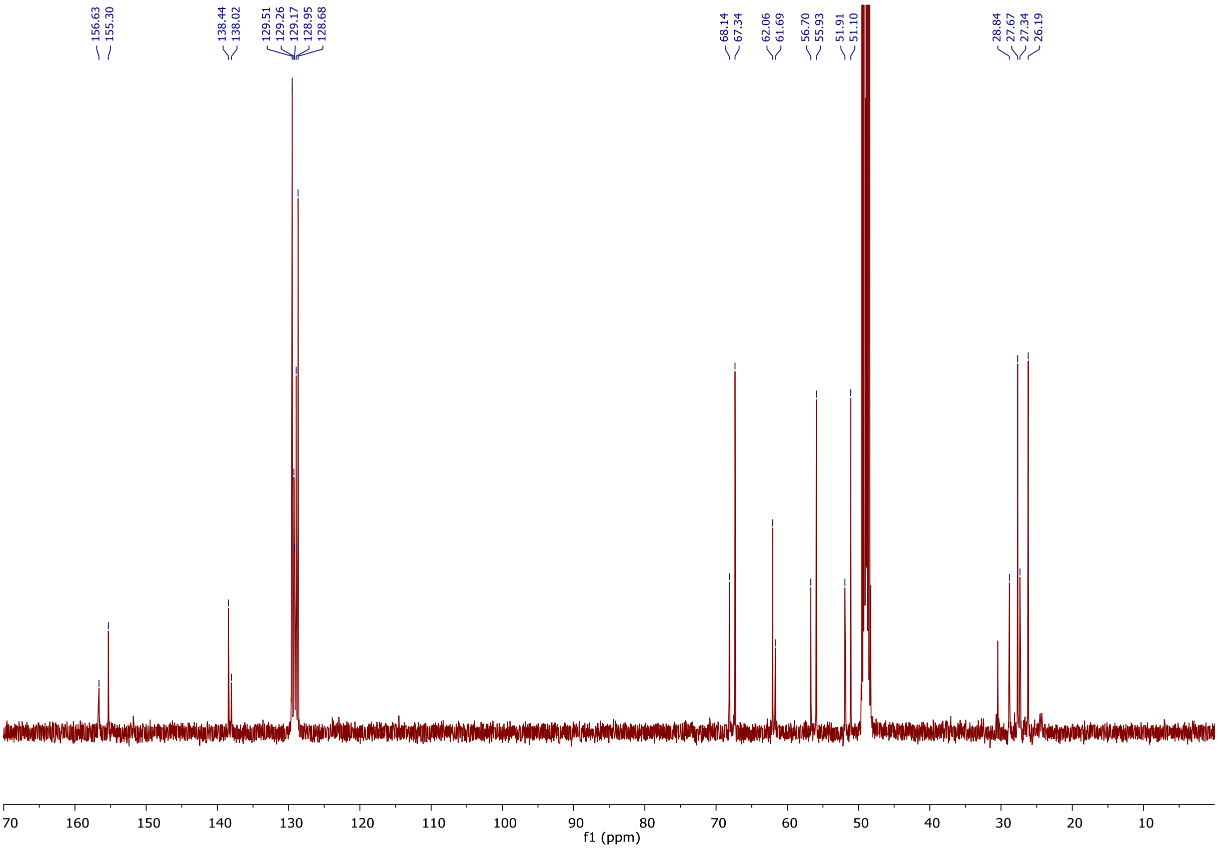

Benzyl 4-amino-3-azaspiro[4.5]decane-1-carboxylate (**7f**)

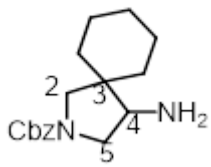

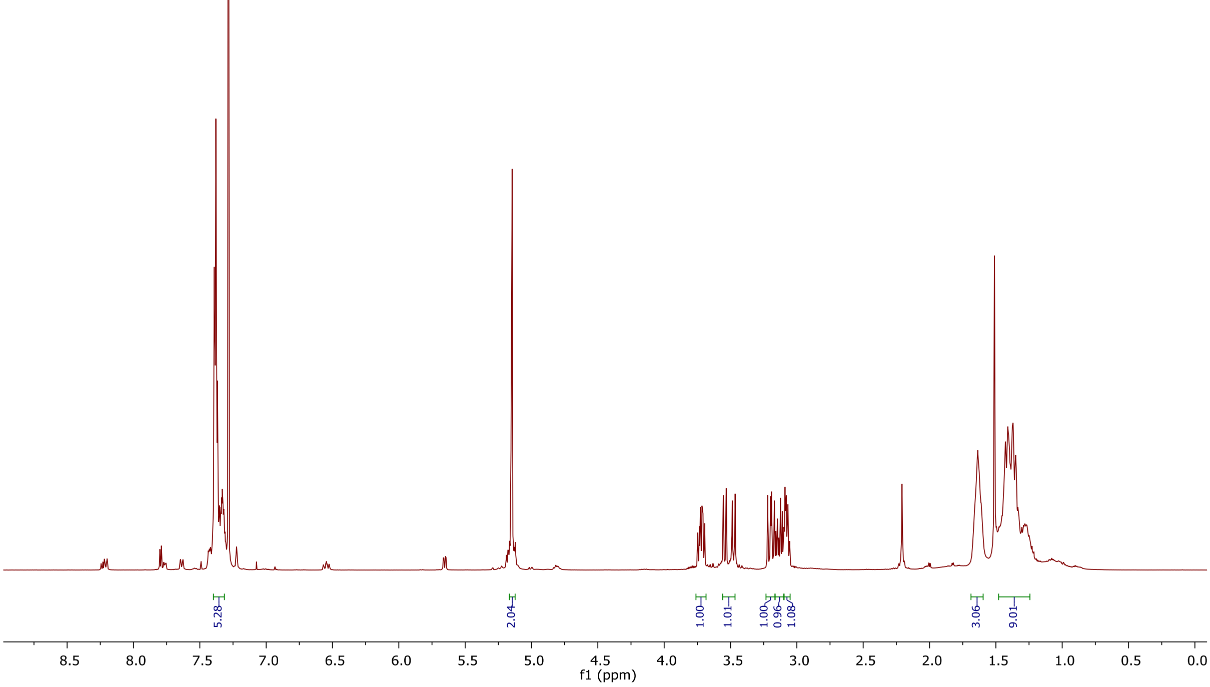

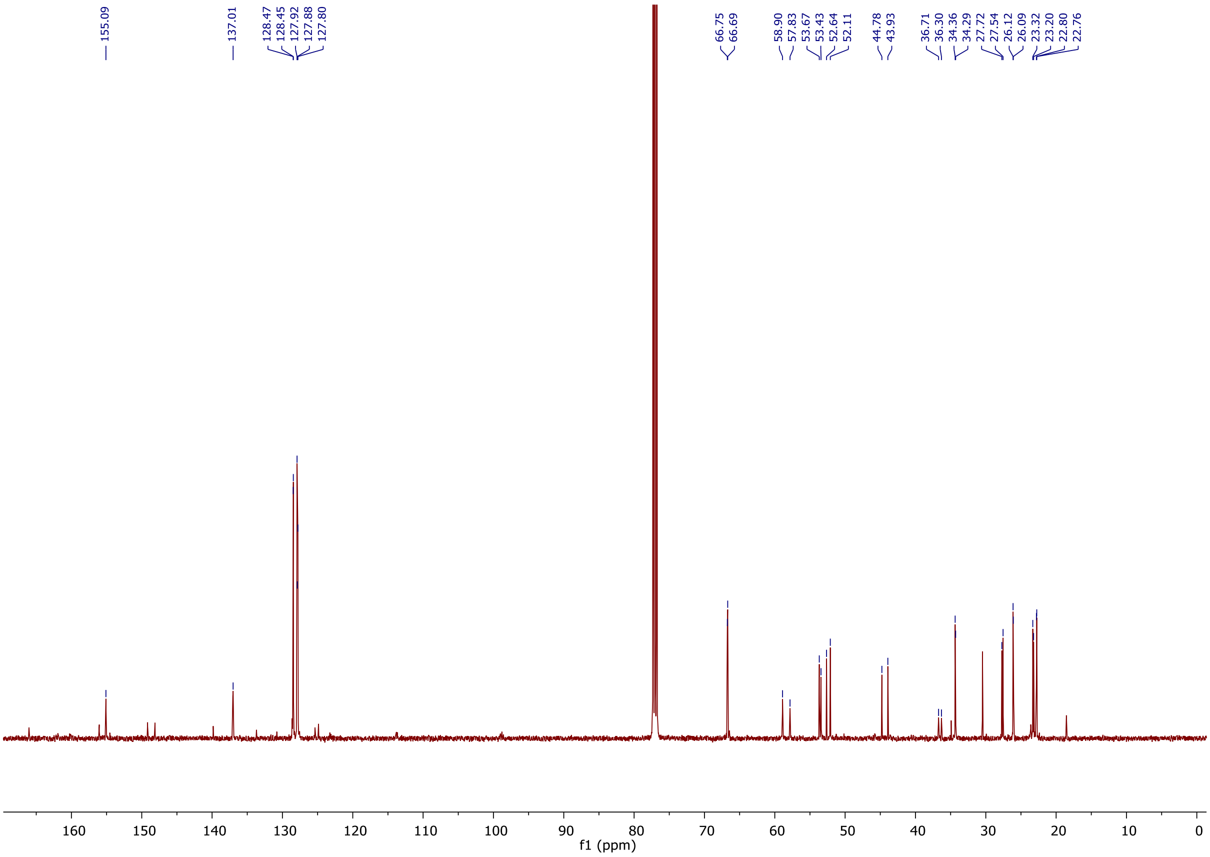

Benzyl 3-amino-4-methylpiperidine-1-carboxylate (**7g**)

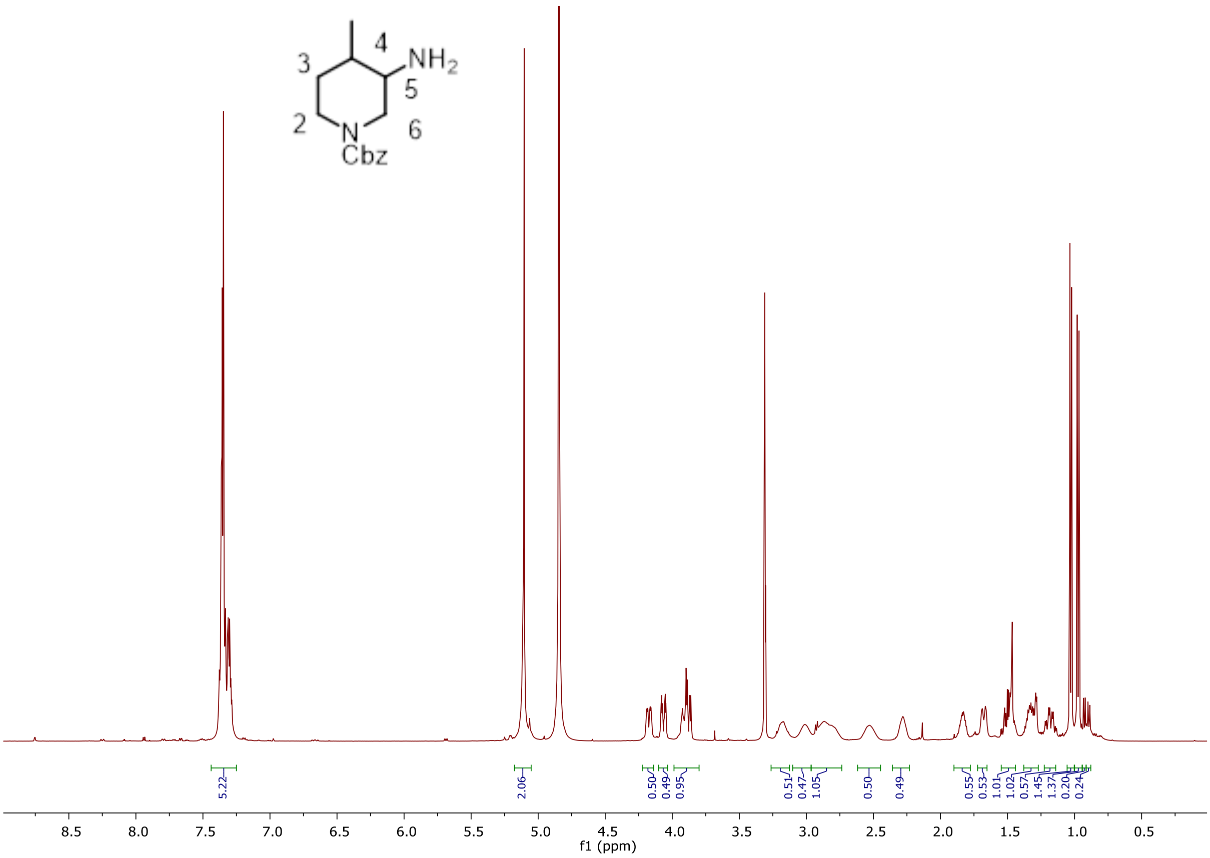

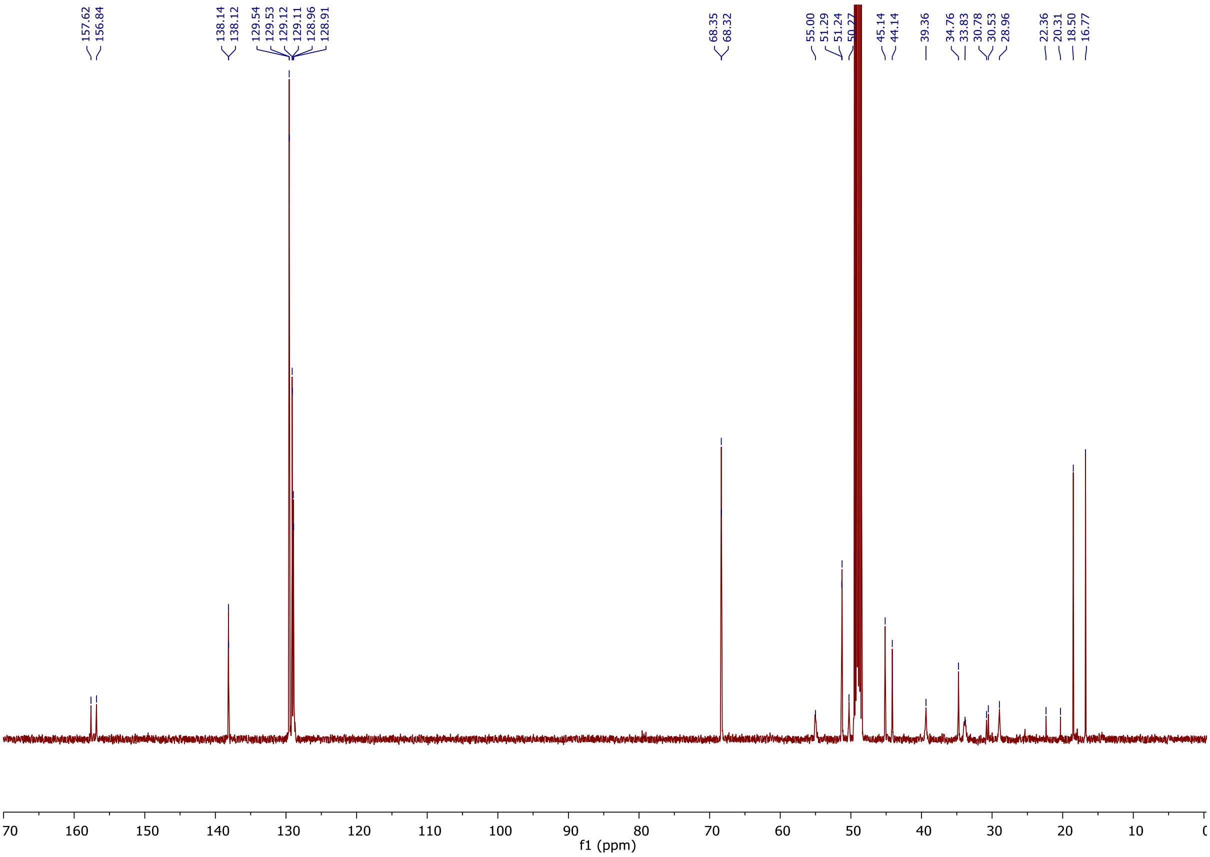

Benzyl 3-amino-5-(*tert*butyl)azepane-1-carboxylate (**7h**)

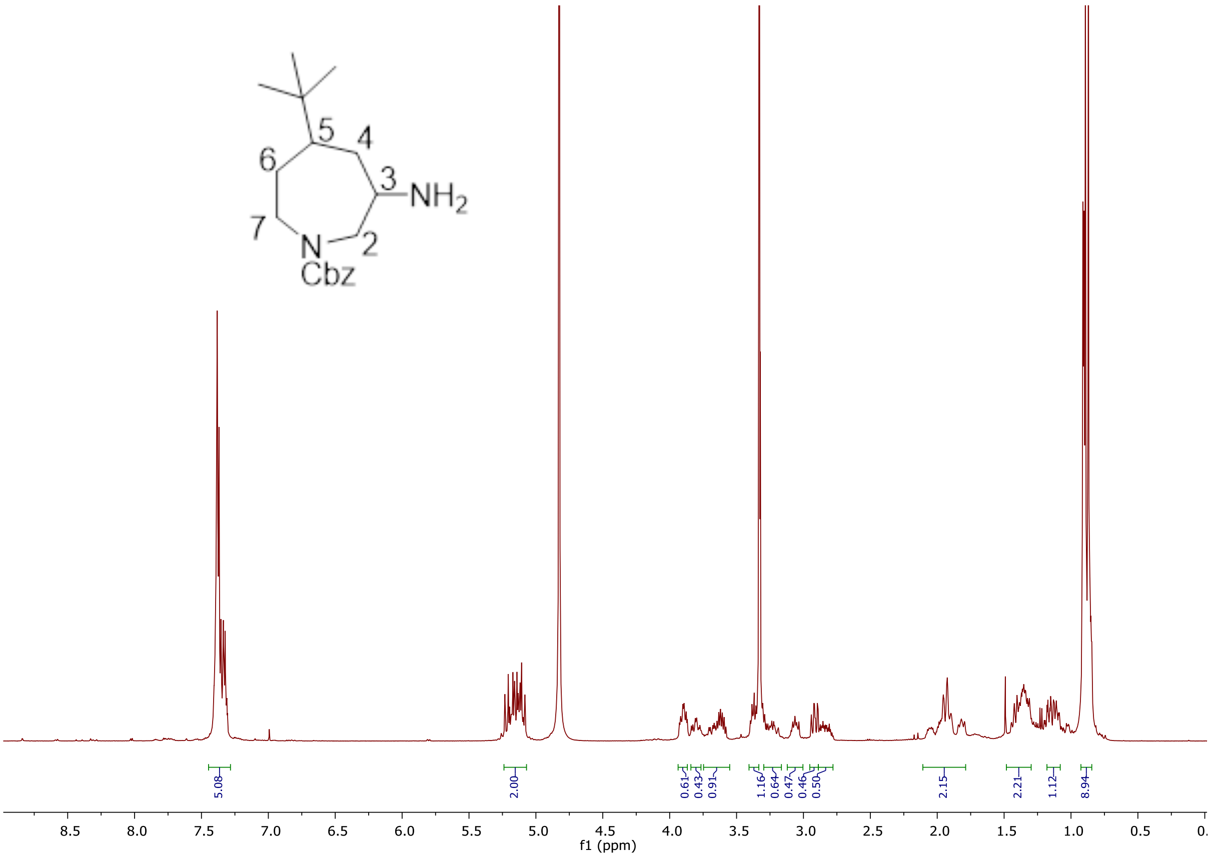

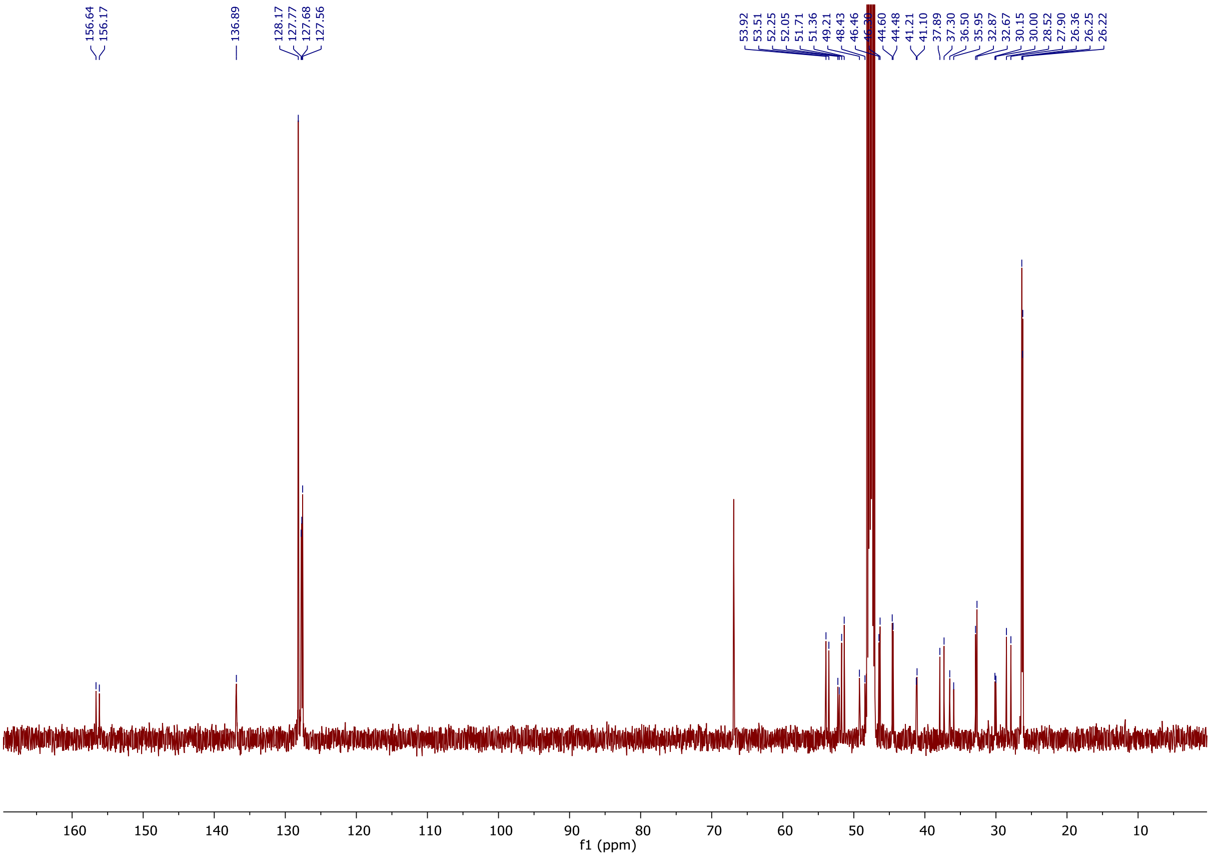

Benzyl 5-amino-2-methylpiperidine-1-carboxylate (**7i**)

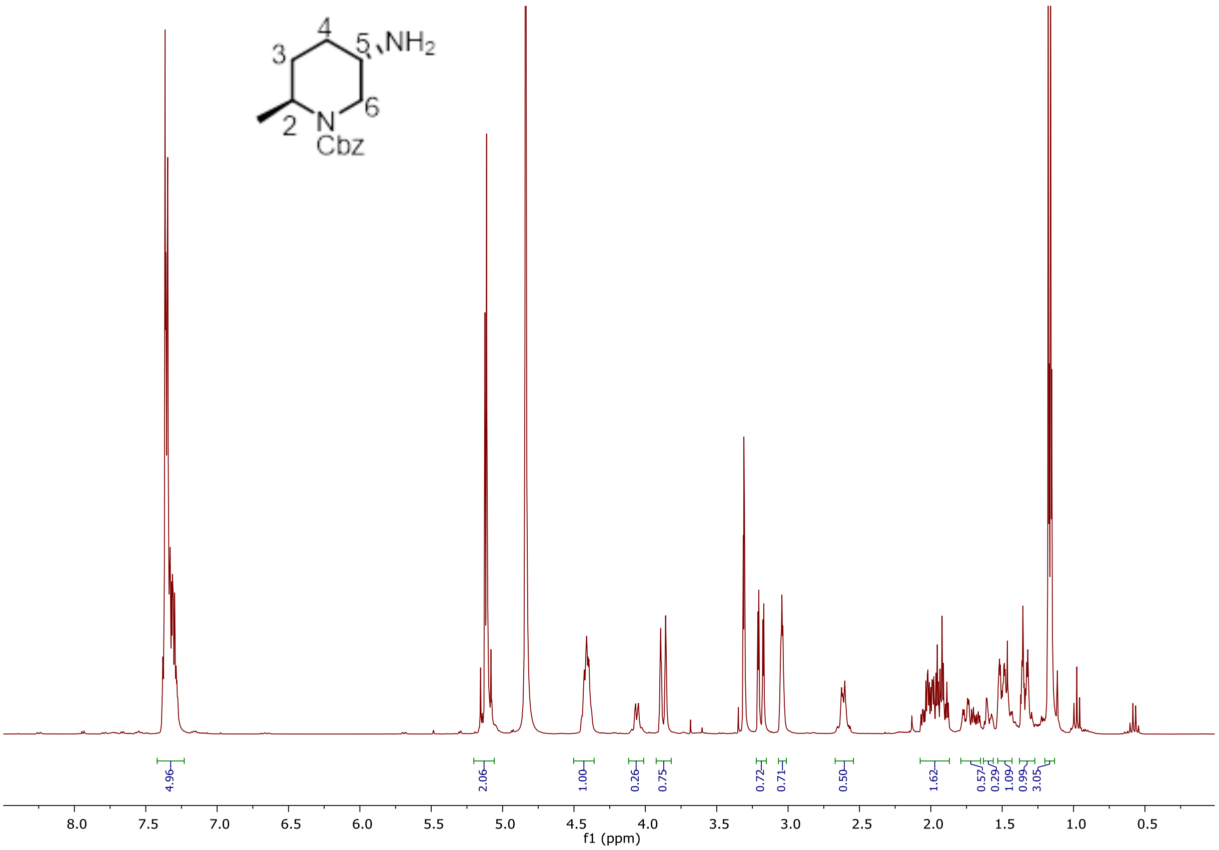

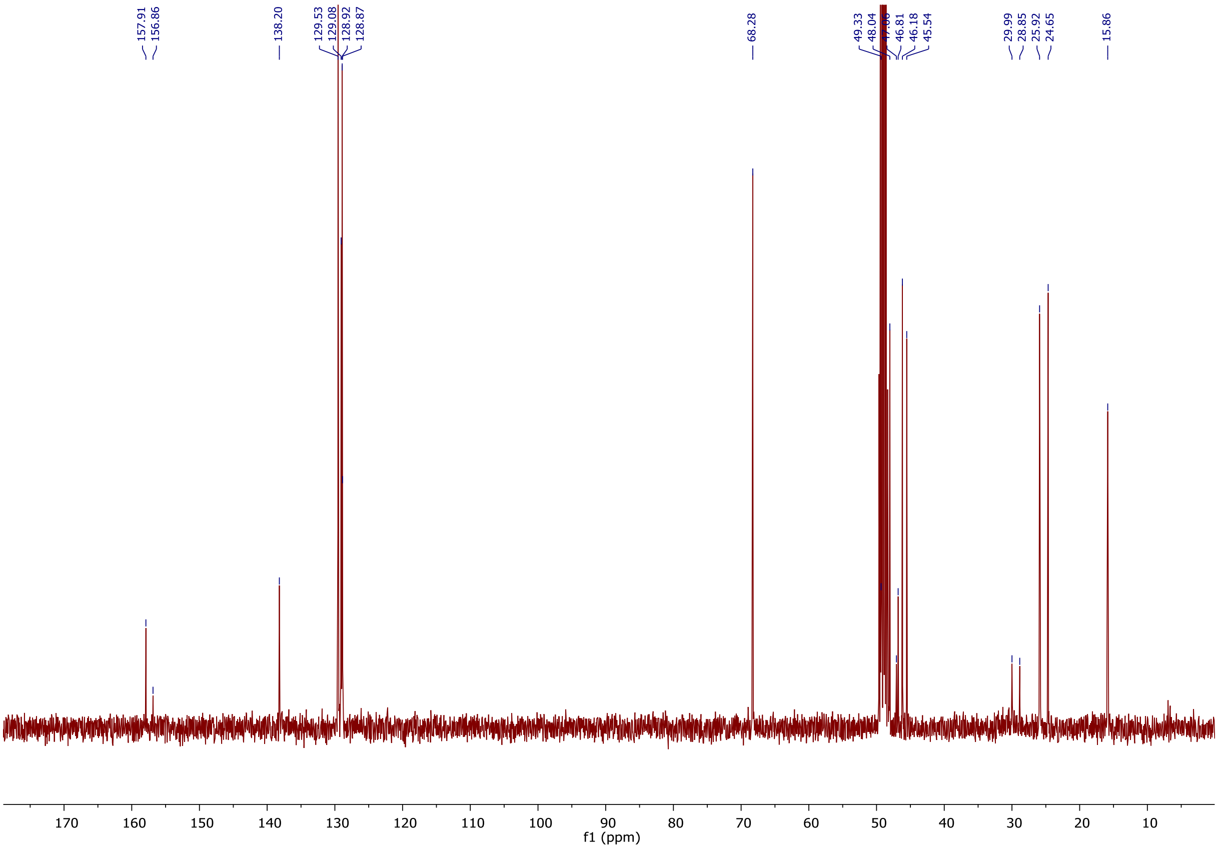

Benzyl 3a-aminooctahydro-2*H*-isoindole-2-carboxylate (**7j**)

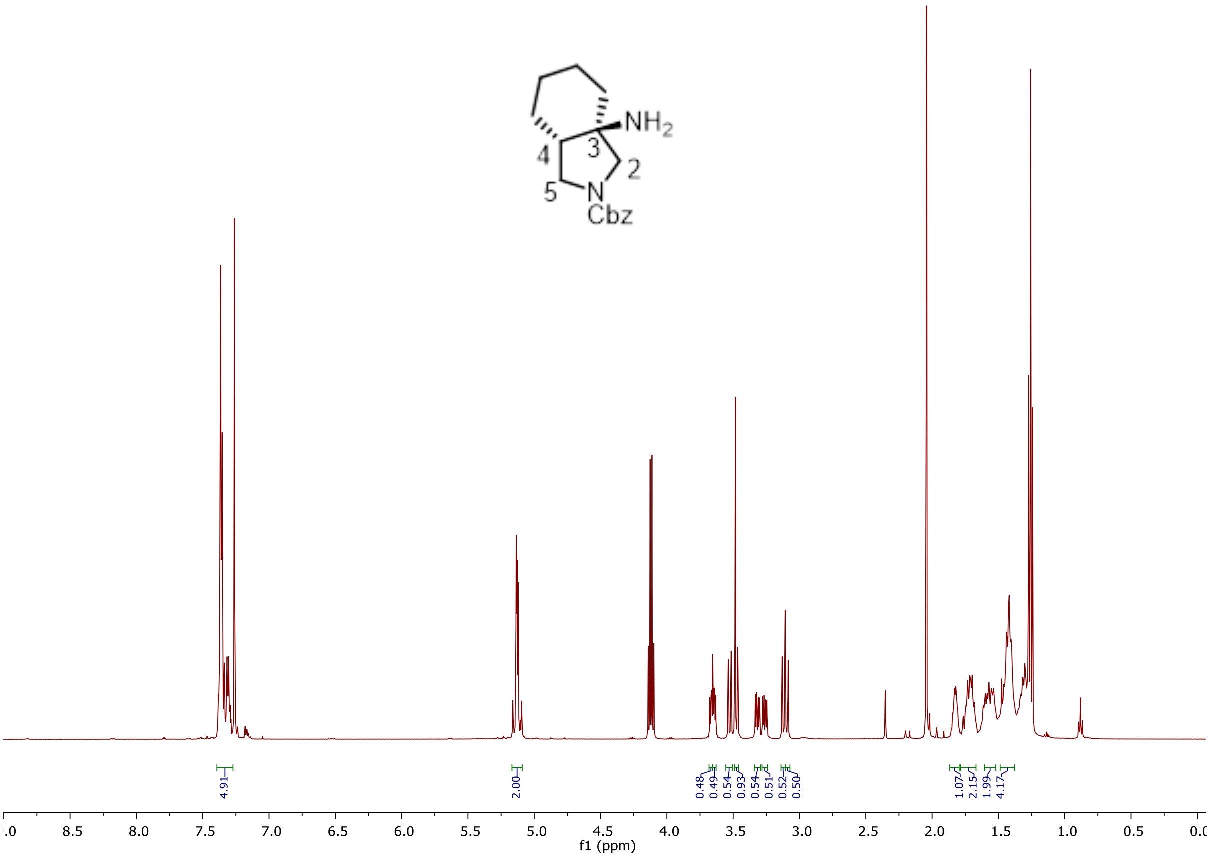

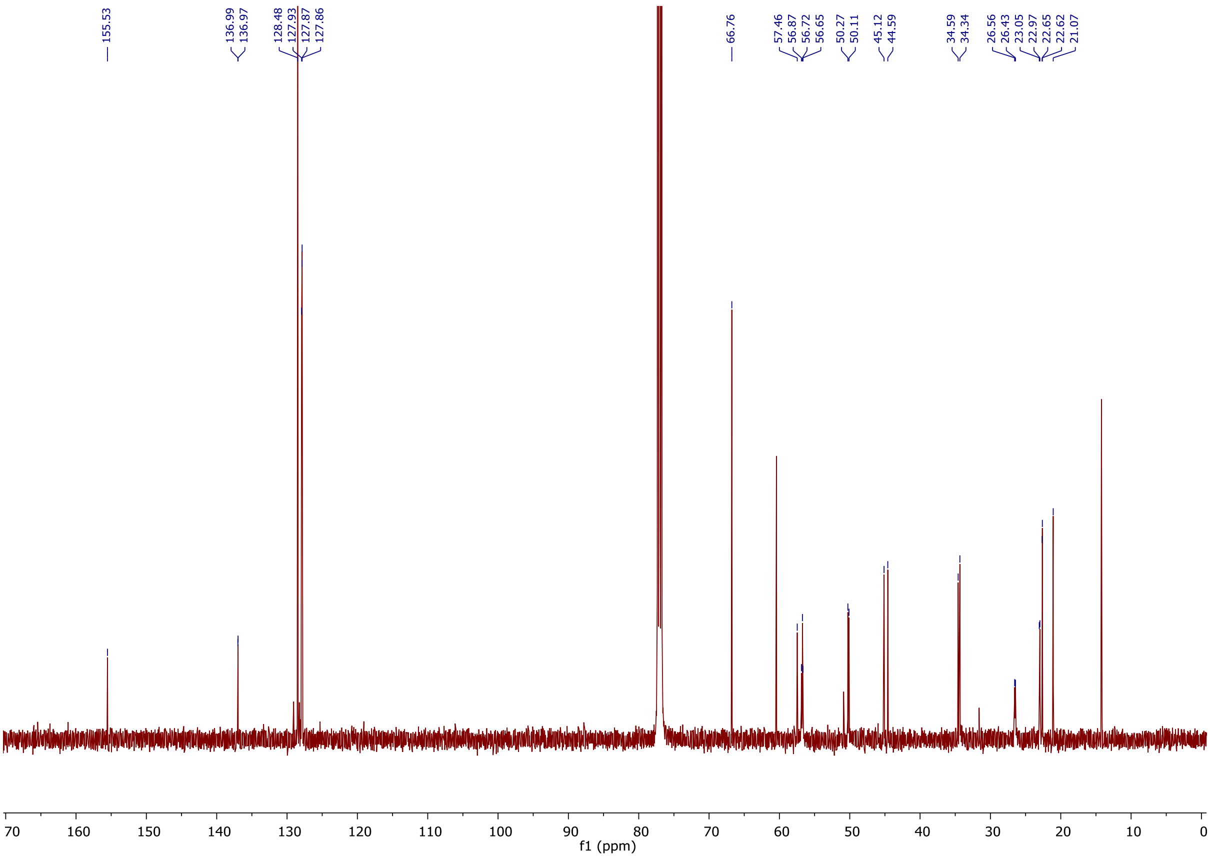

Benzyl 3-((*tert*-butoxycarbonyl)amino)pyrrolidine-1-carboxylate (**8**)

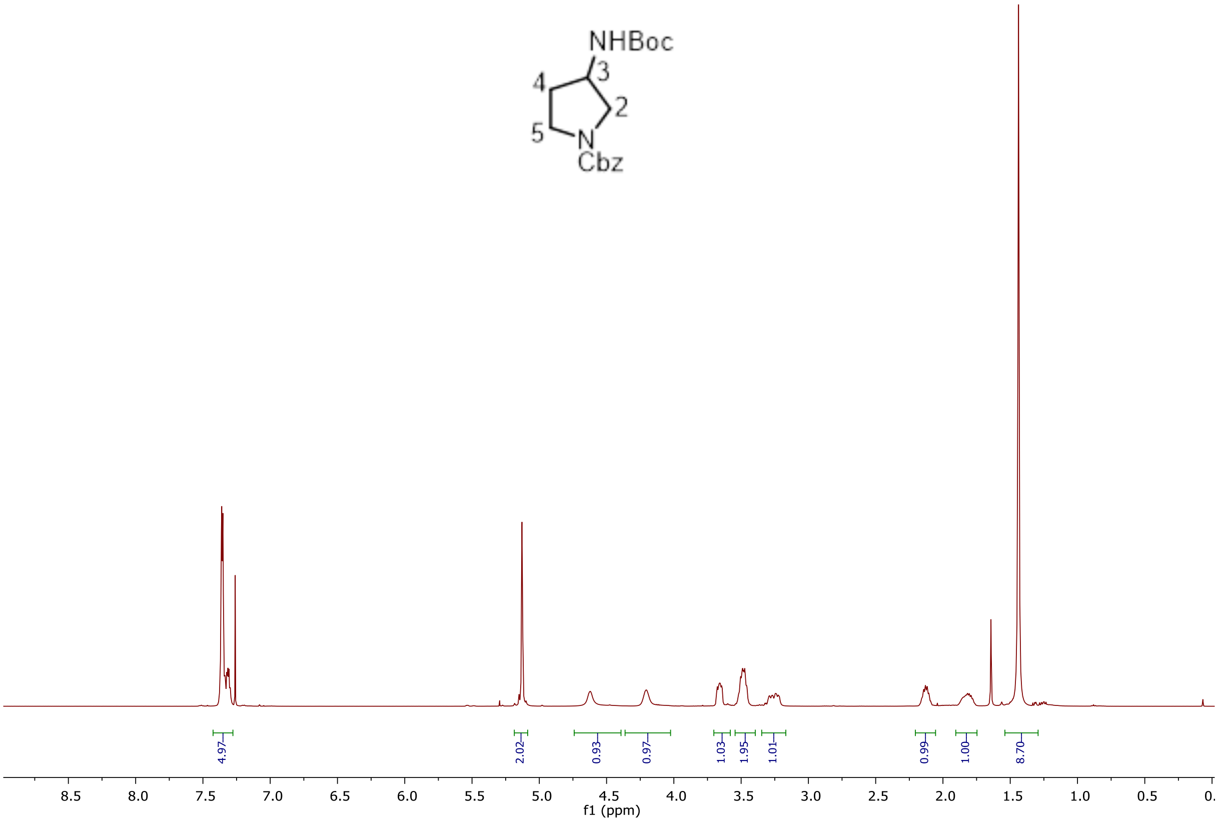

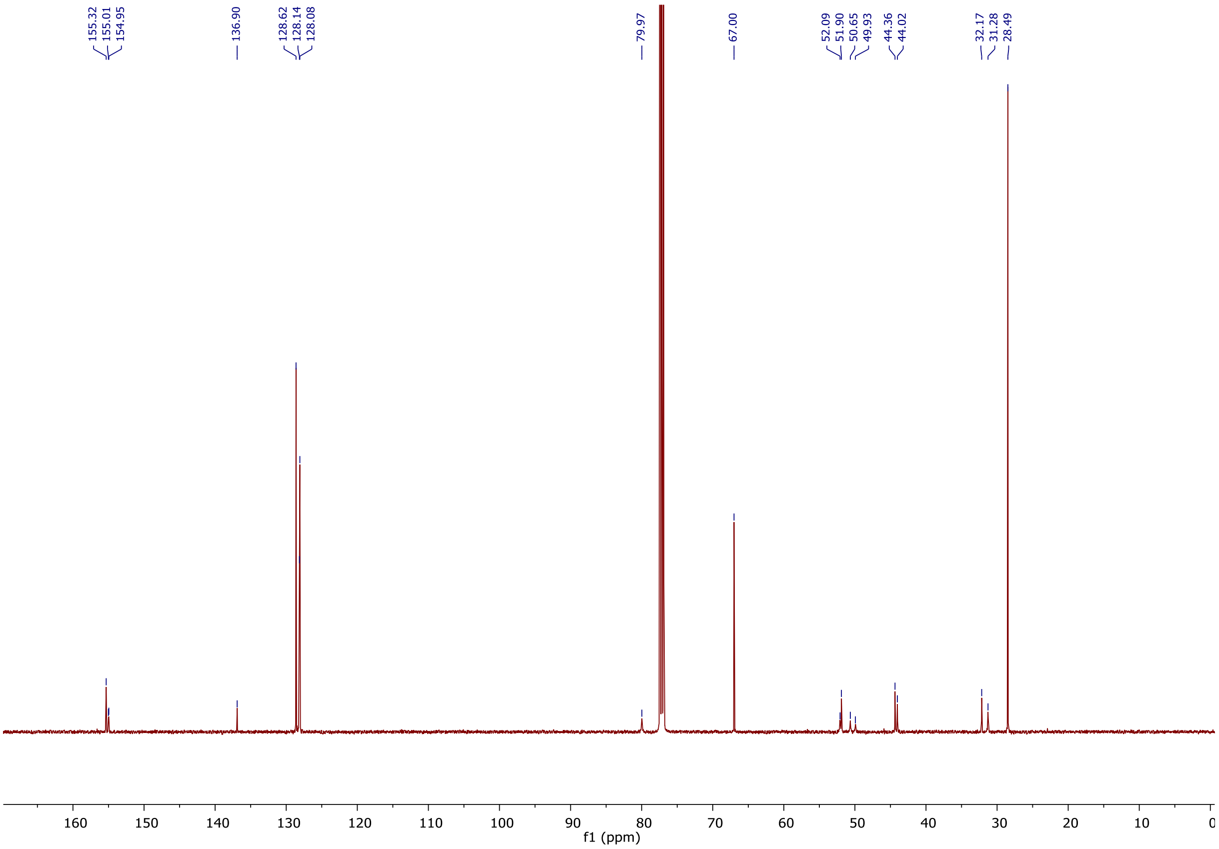

Benzyl 3-(furan-2-carboxamido)pyrrolidine-1-carboxylate (**9**)

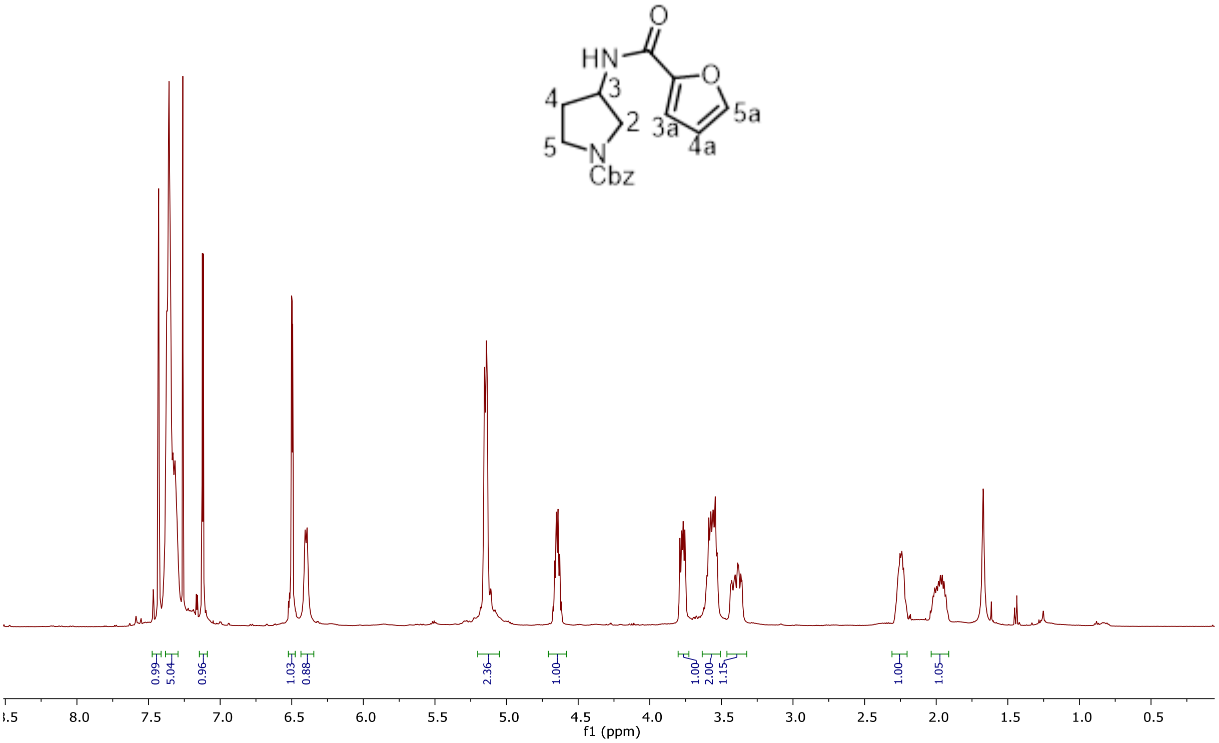

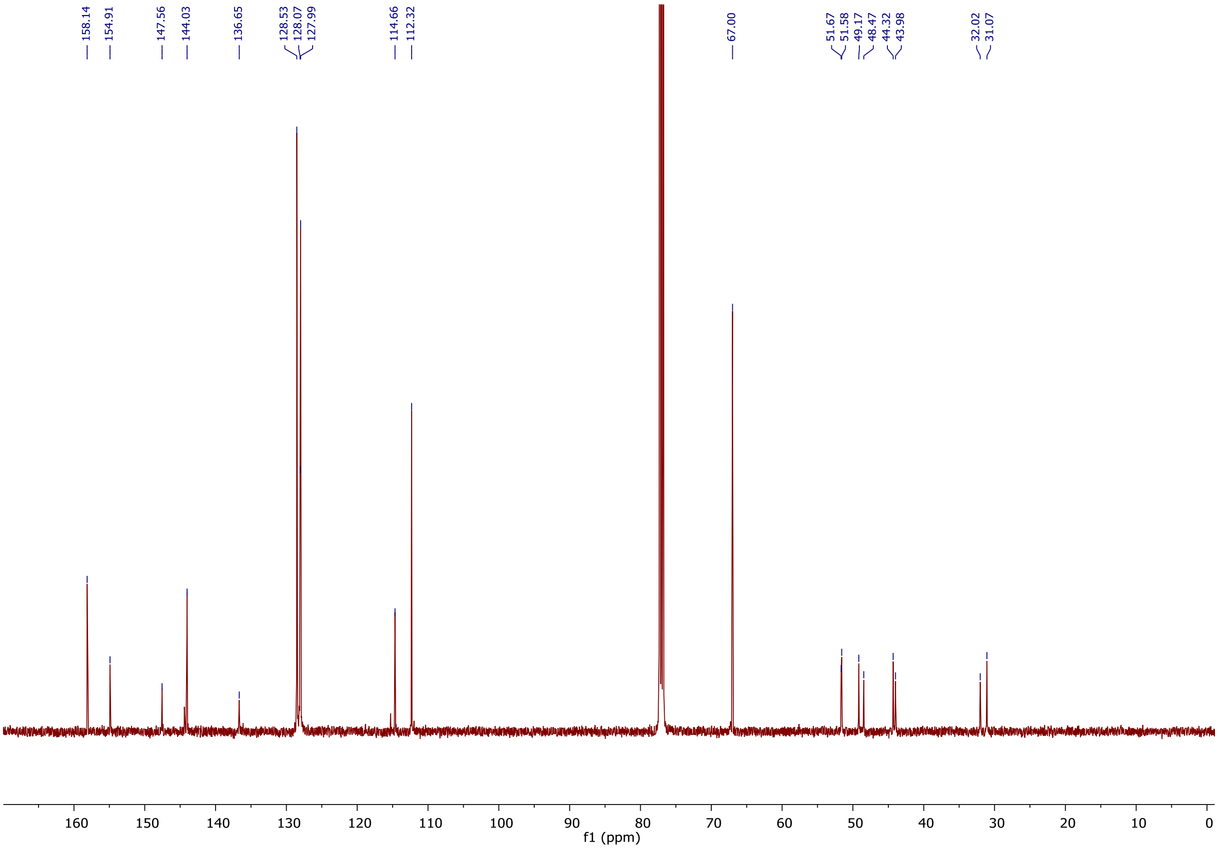

Benzyl 3-(3-(3-fluorophenyl)ureido)pyrrolidine-1-carboxylate (**10**)

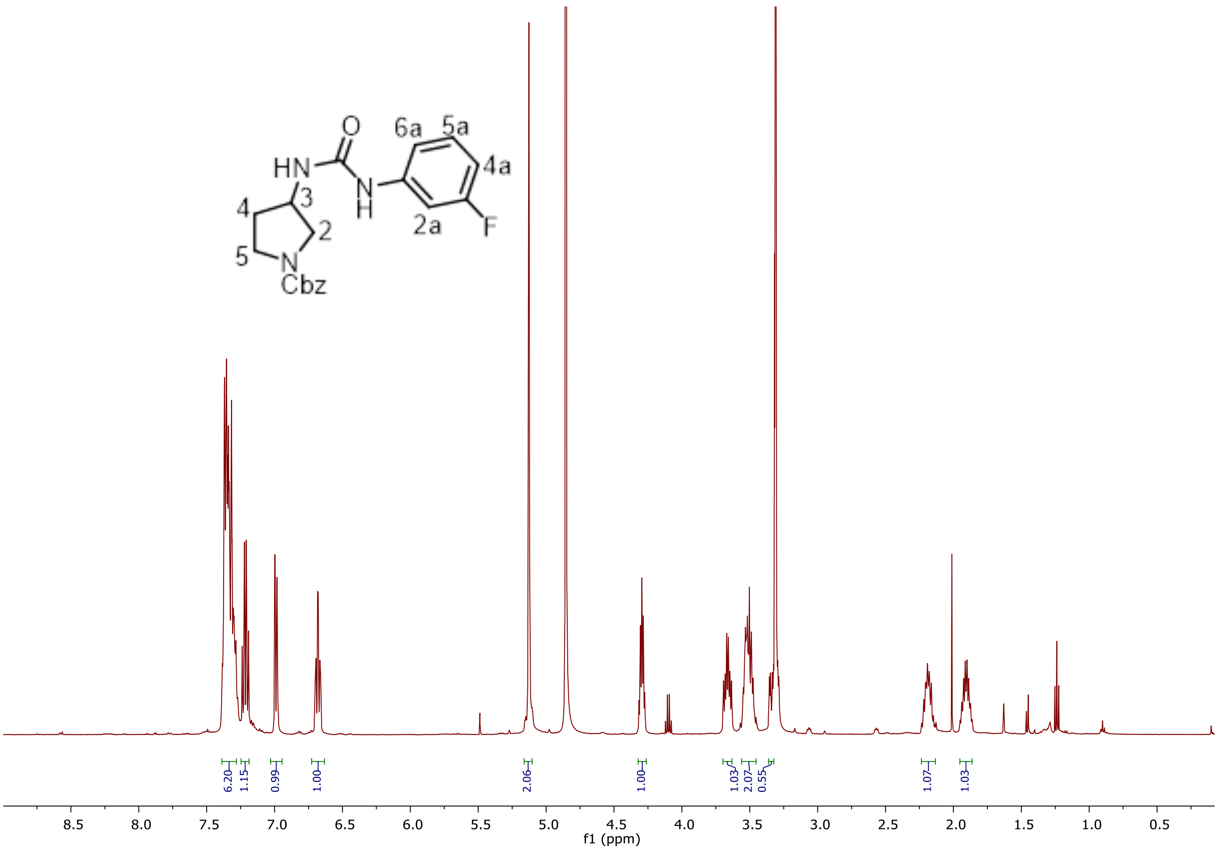

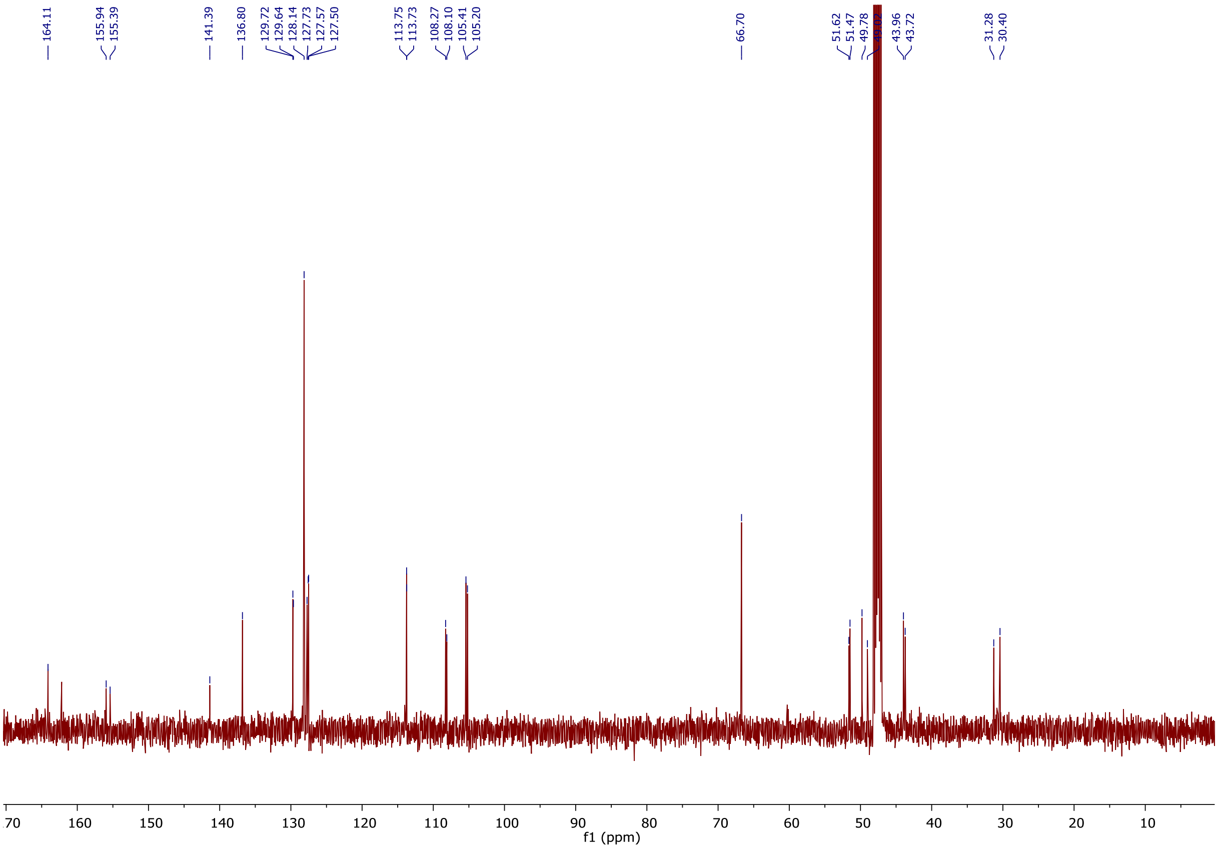

Benzyl 3-(pyridine-3-sulfonamido)pyrrolidine-1-carboxylate (**11**)

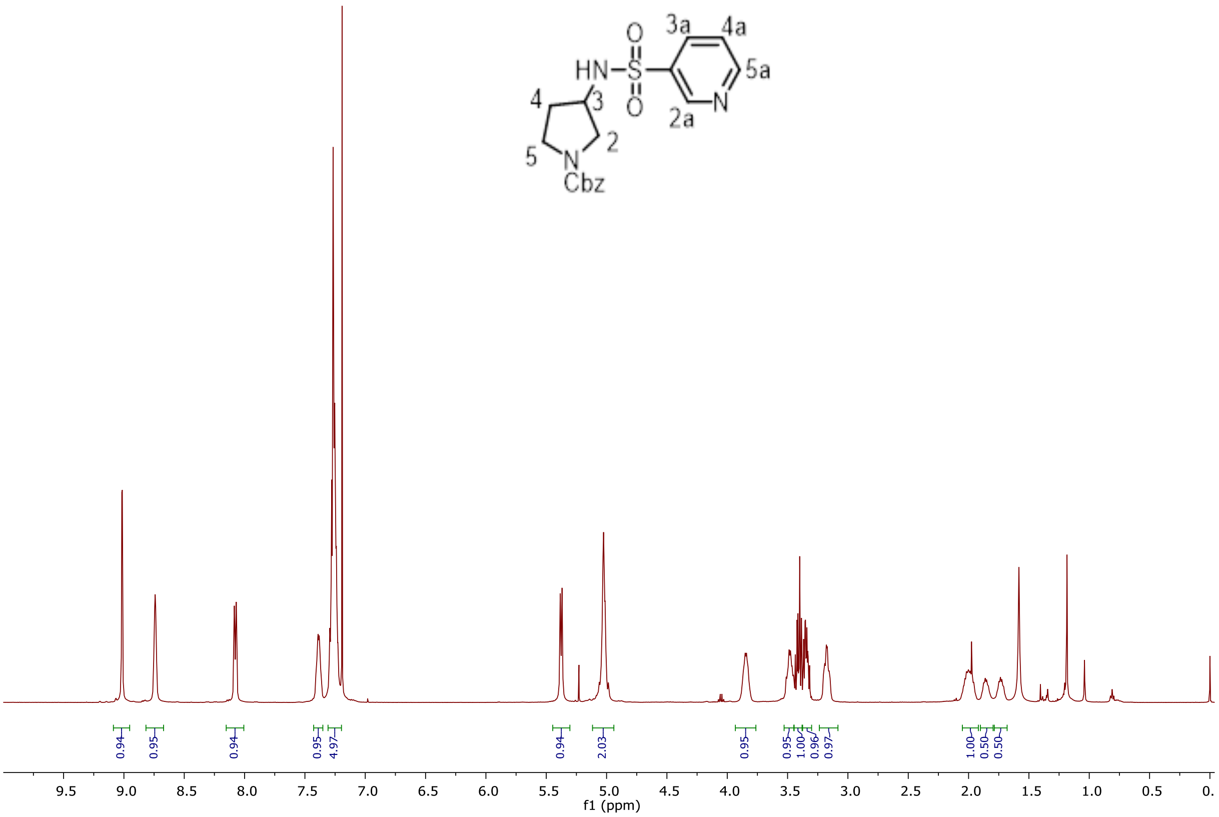

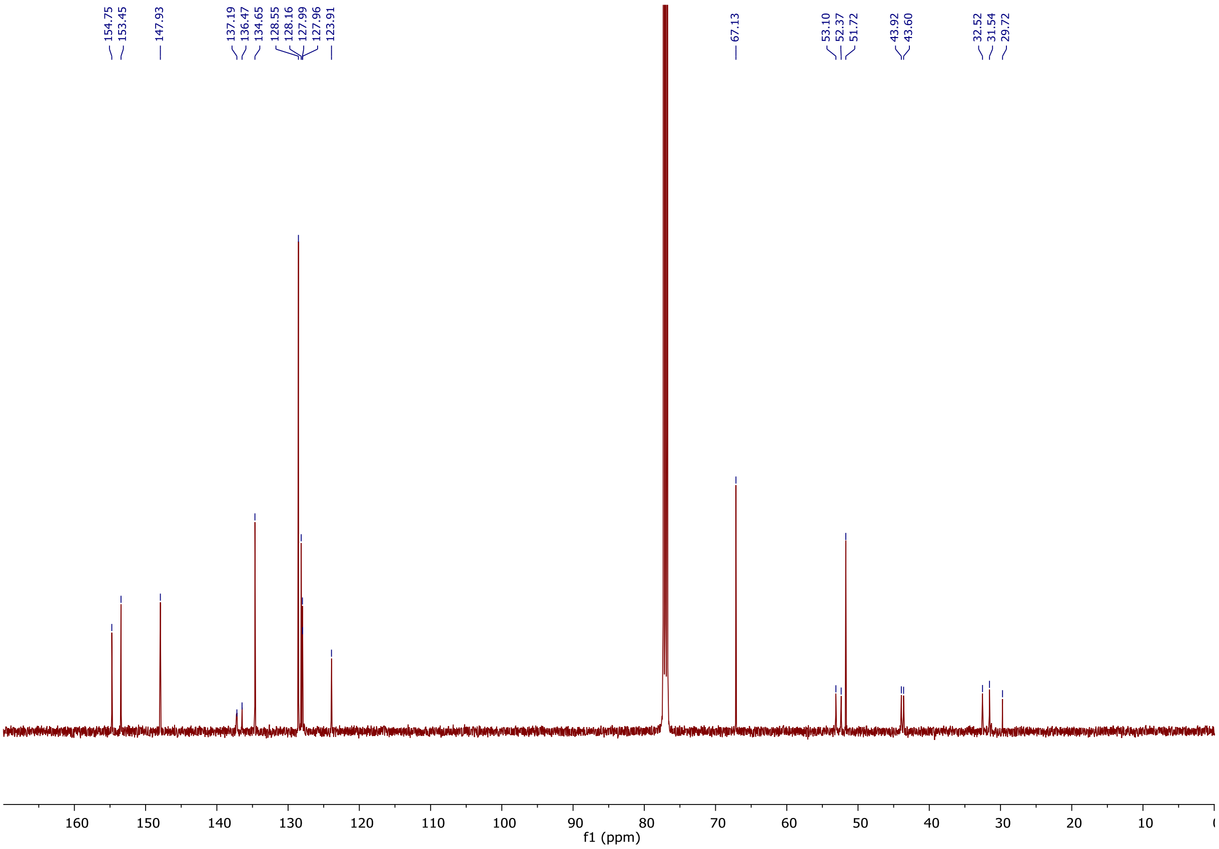

Benzyl 3-(phenylamino)pyrrolidine-1-carboxylate (**12a**)

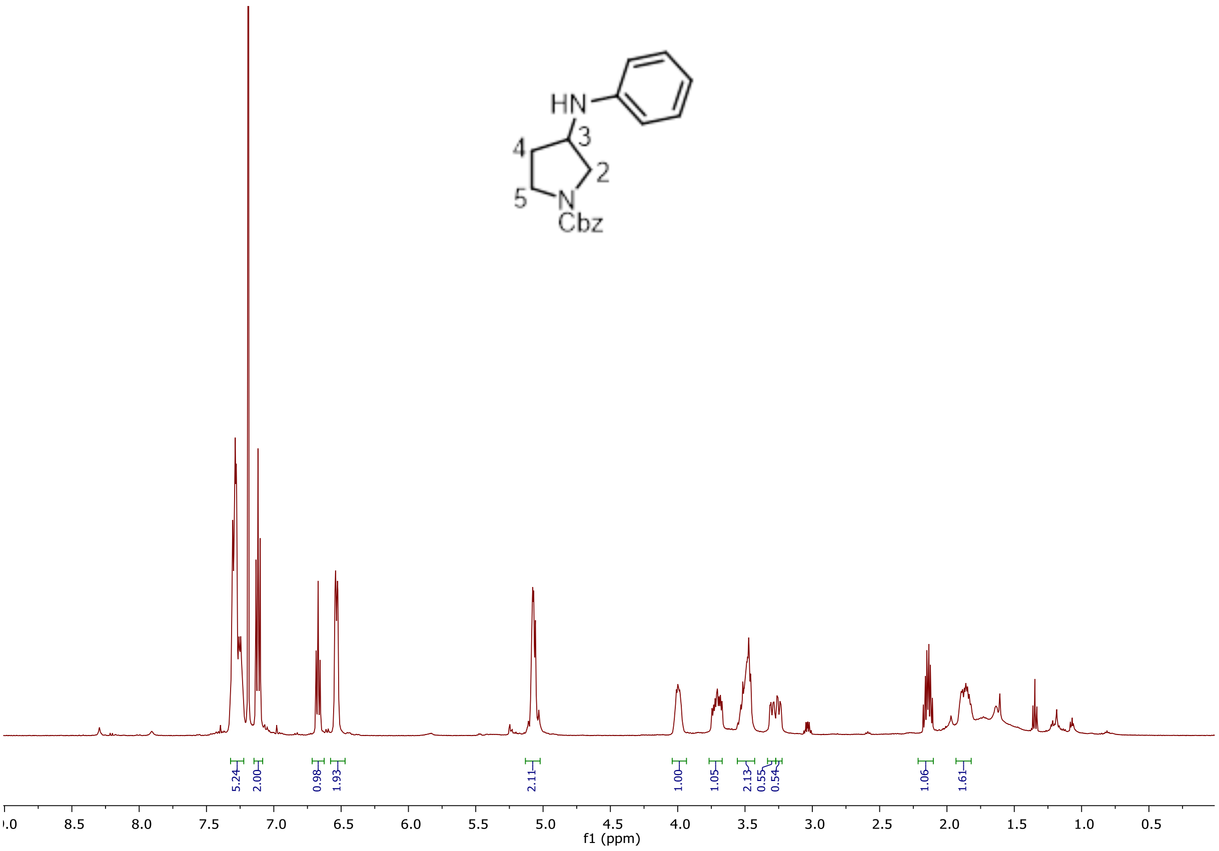

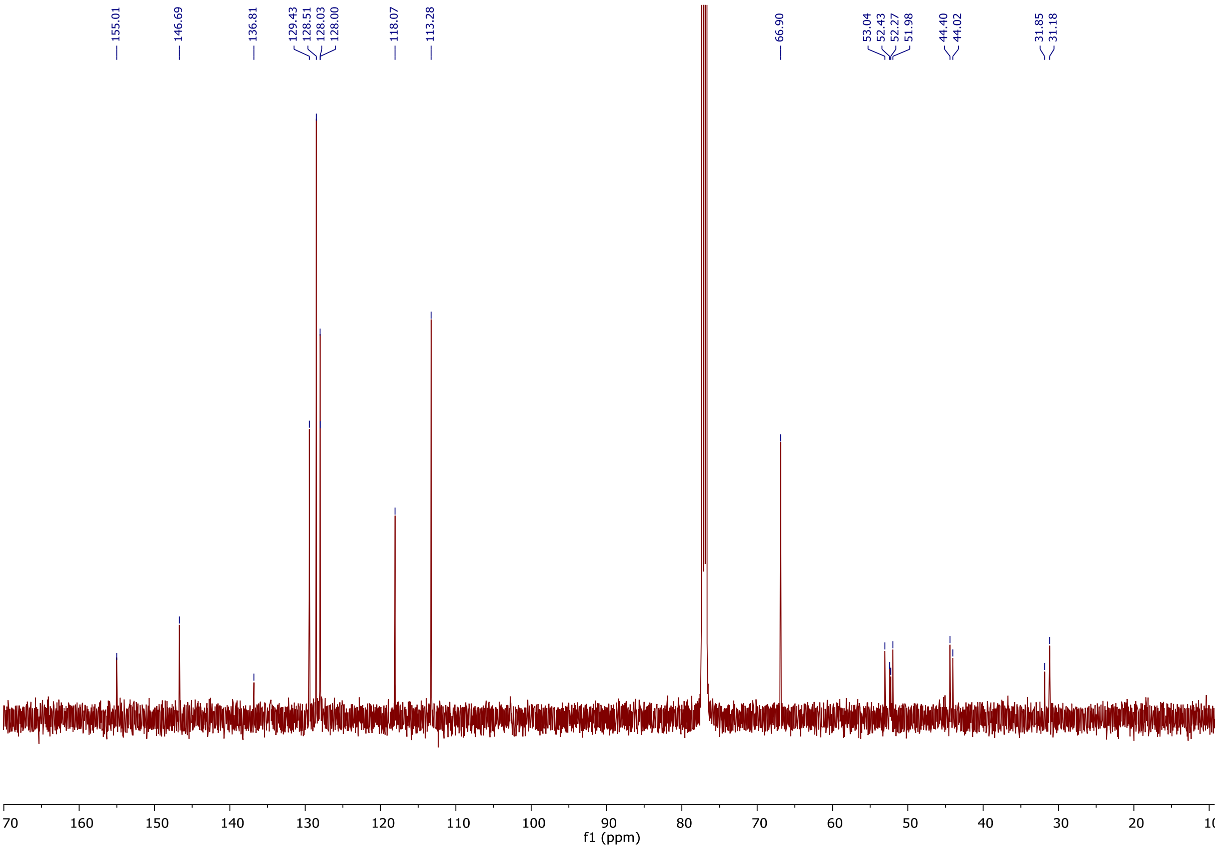

Benzyl 3-((3-(trifluoromethyl)phenyl)amino)pyrrolidine-1-carboxylate (**12b**)

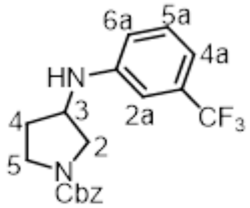

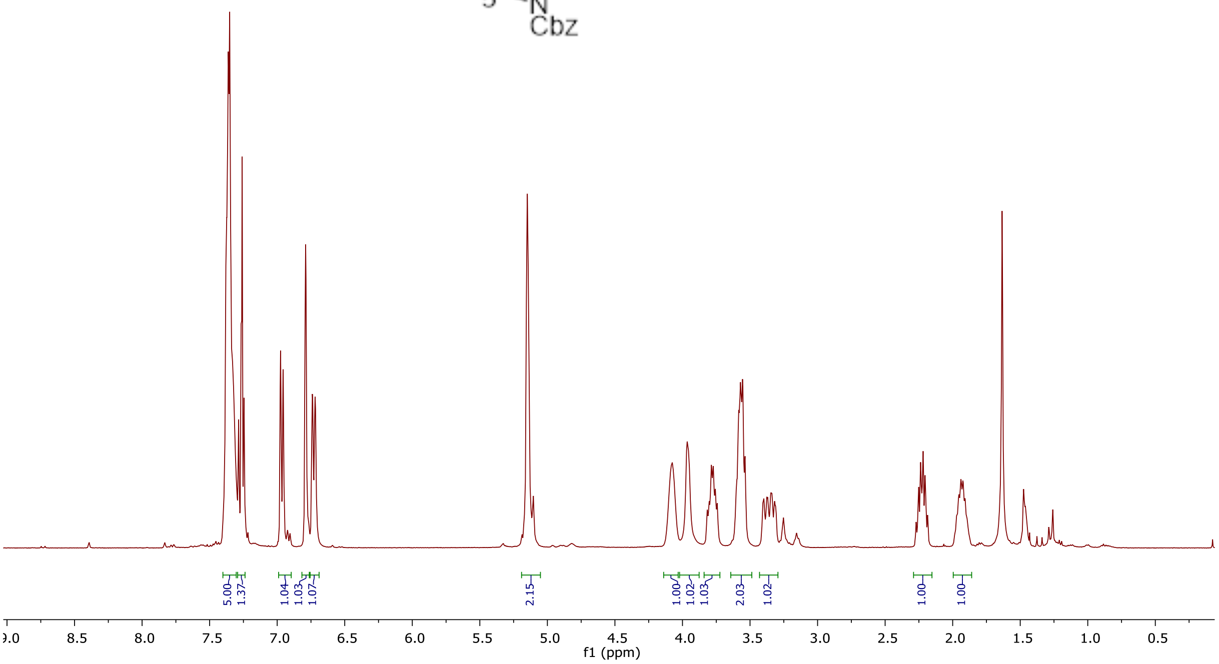

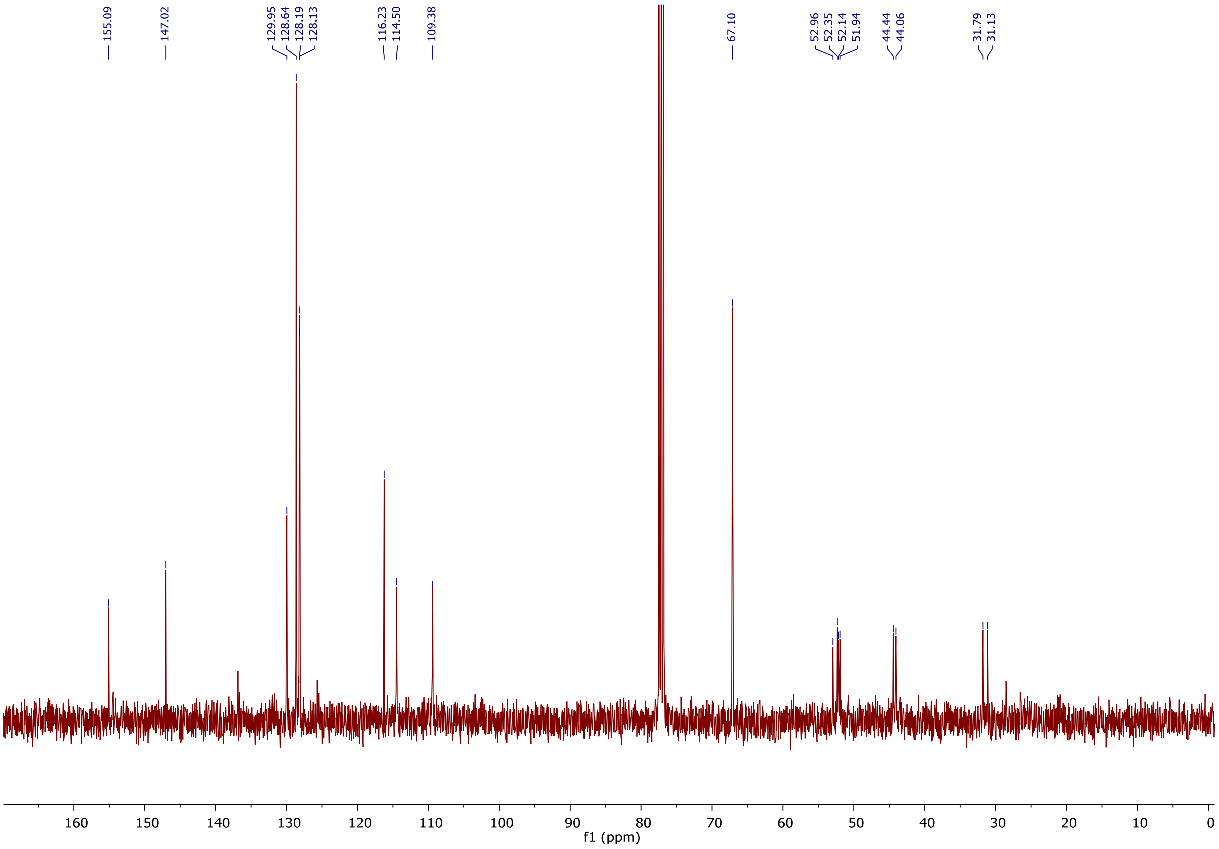

Benzyl 3-(pyridin-2-ylamino) pyrrolidine-1-carboxylate (**12c**)

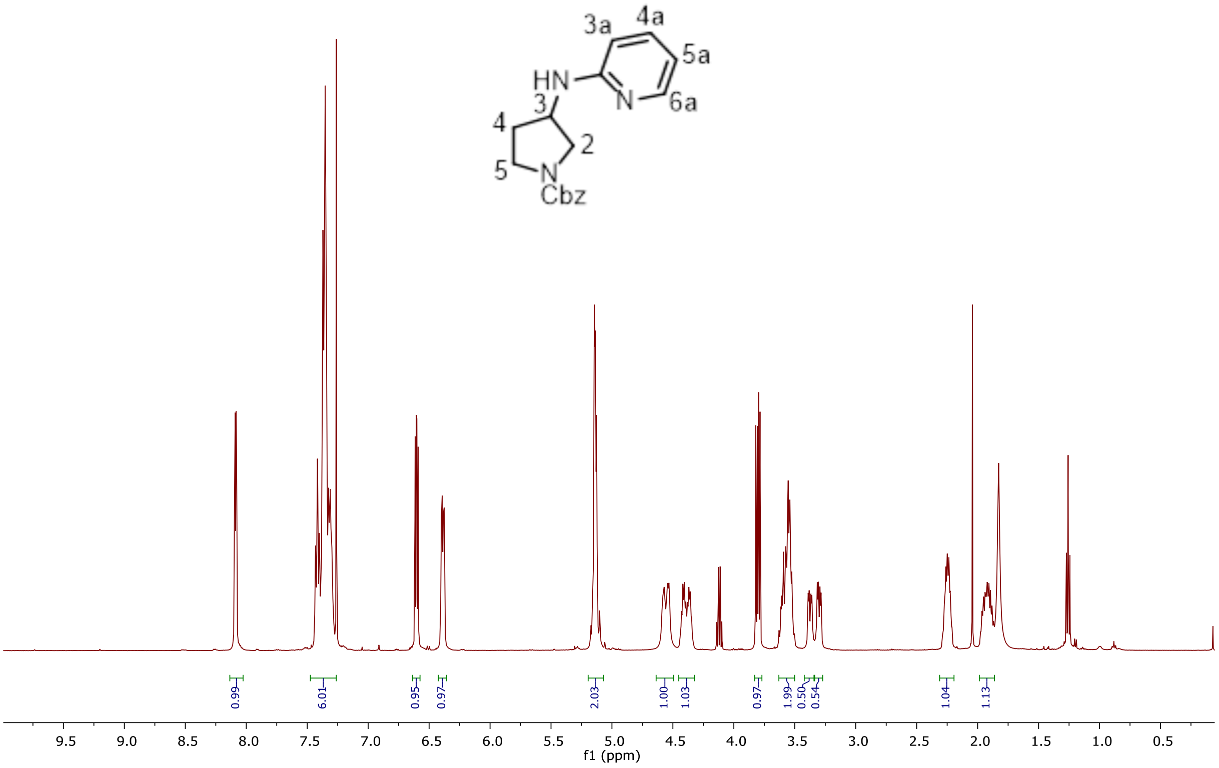

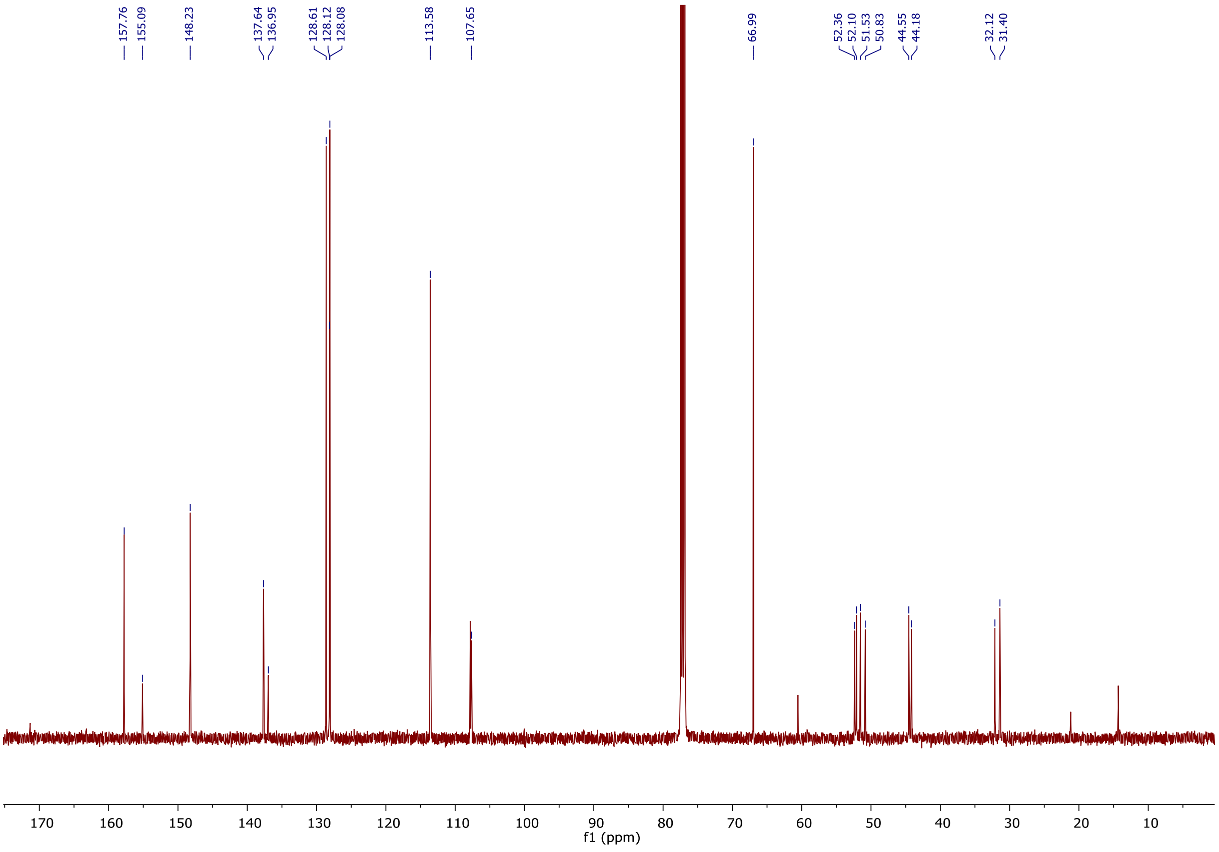

Benzyl 3-(pyrimidin-2-ylamino)pyrrolidine-1-carboxylate (**12d**)

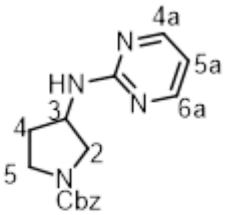

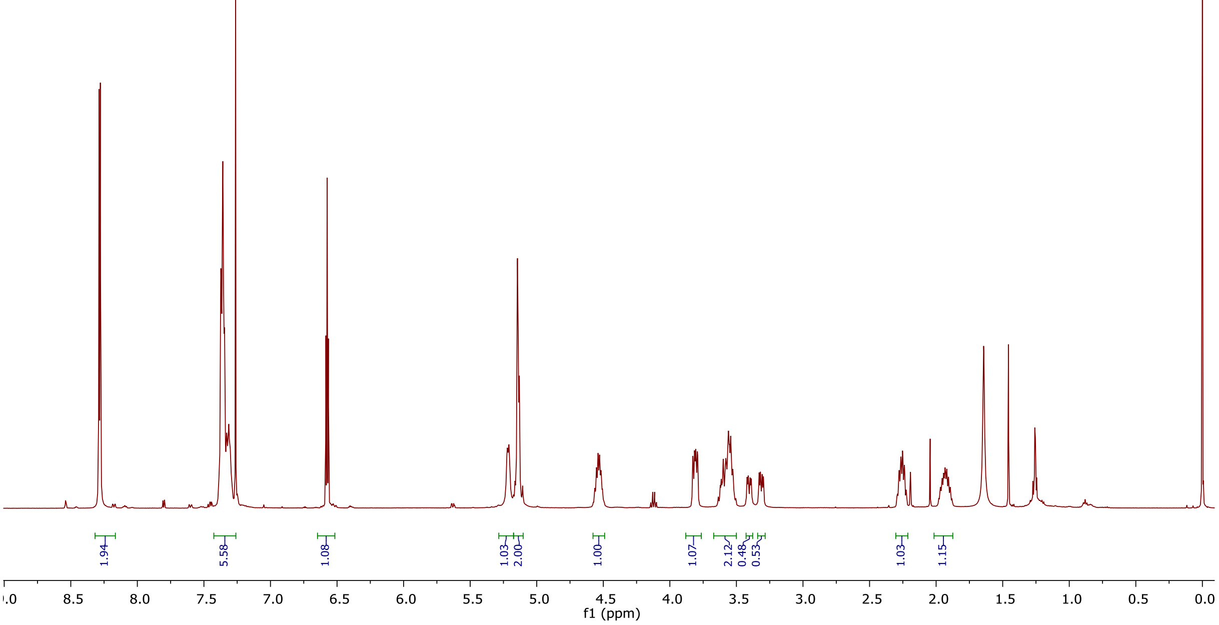

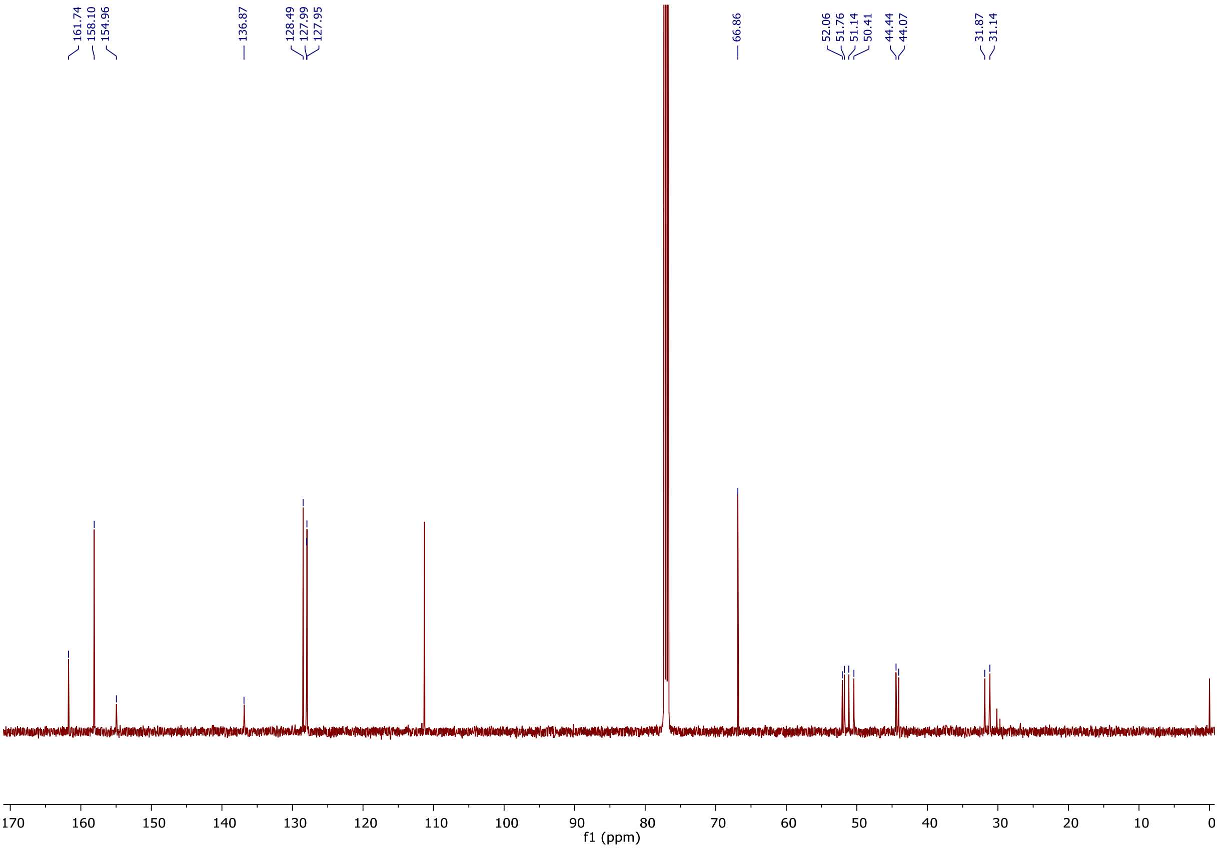

Benzyl 3-((6-methylpyridin-2-yl)amino)pyrrolidine-1-carboxylate (**12e**)

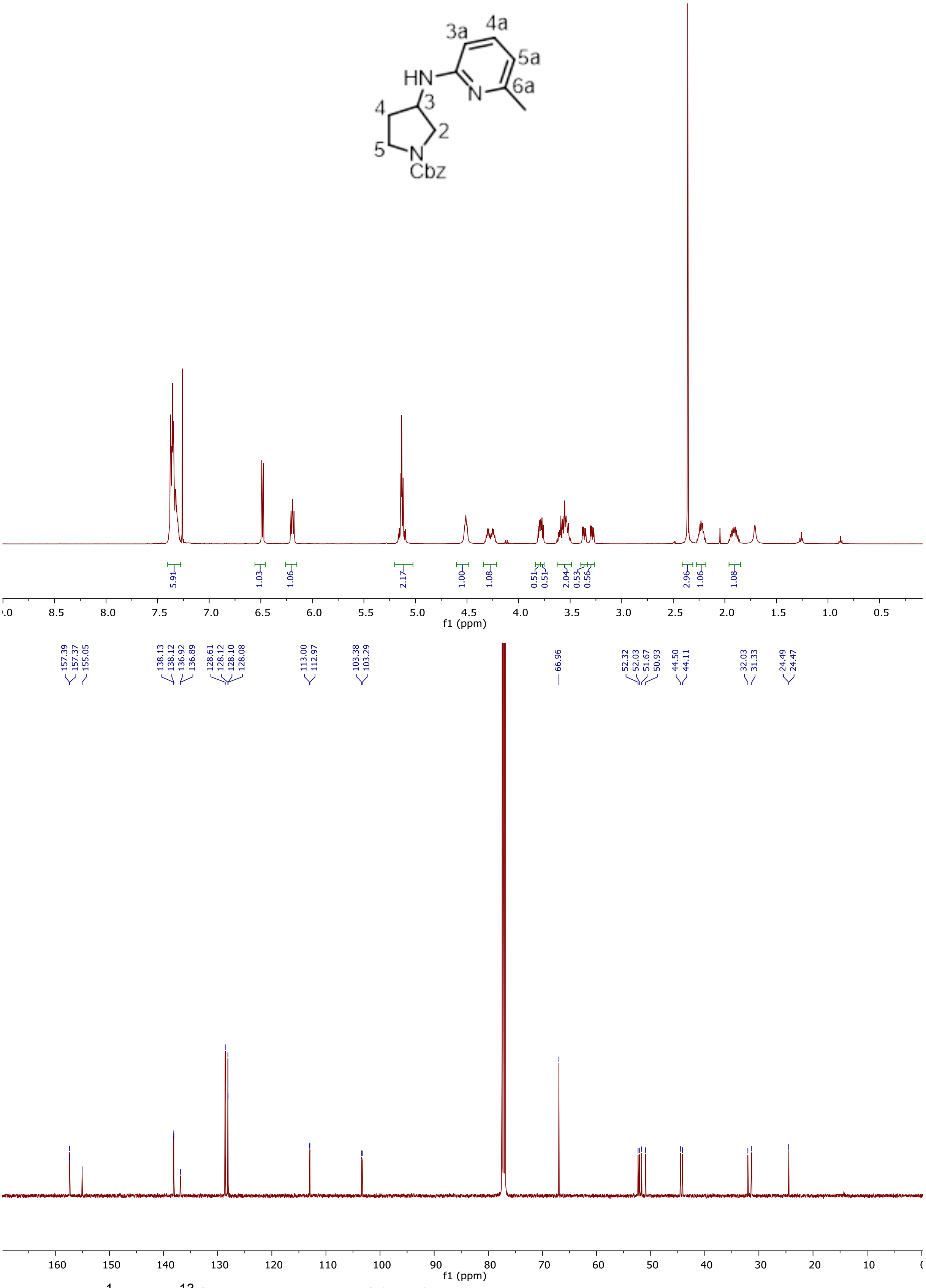

Figure X: ^{1}H and ^{13}C NMR spectra of (**12e**) 423a

Benzyl 2,2-dimethyl-4-(pyridin-2-ylamino)pyrrolidine-1-carboxylate (**12f**)

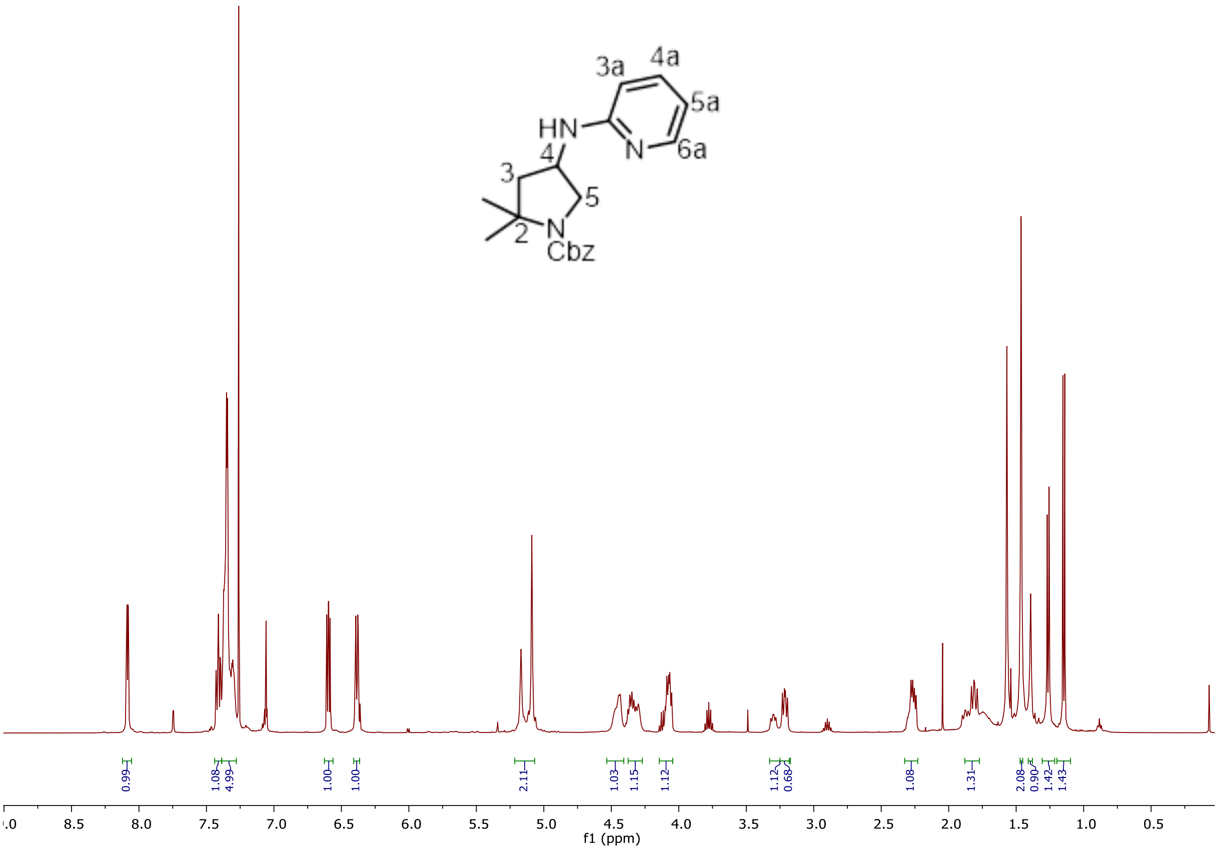

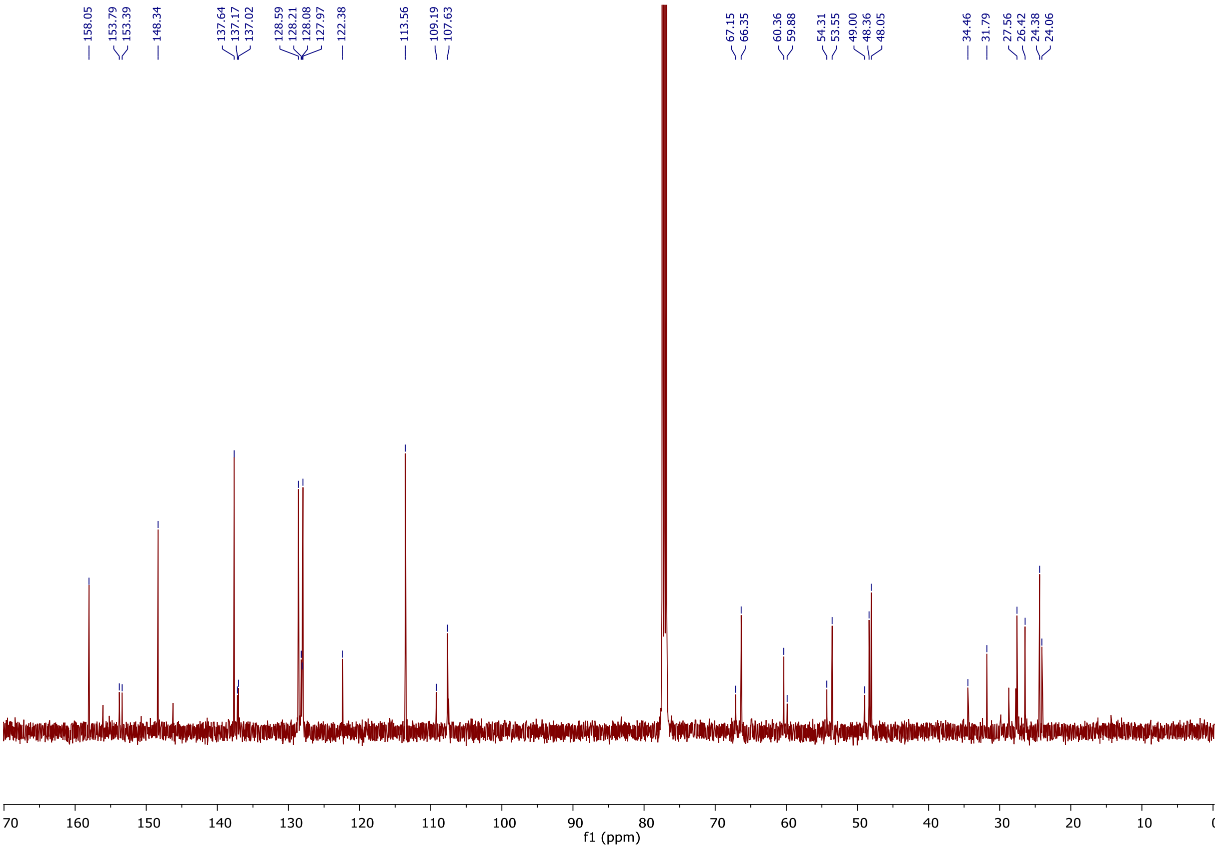

Benzyl 3-(pyridin-2-ylamino)piperidine-1-carboxylate (**12g**)

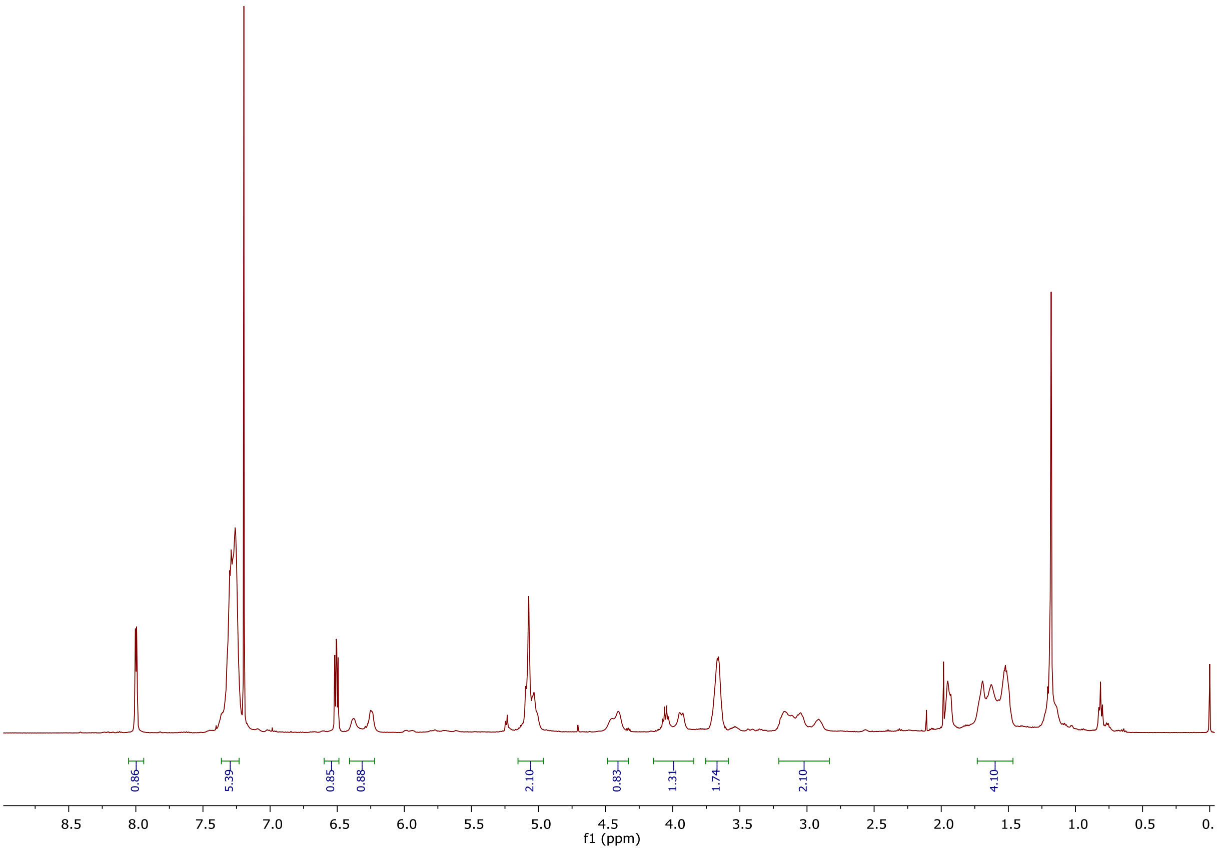

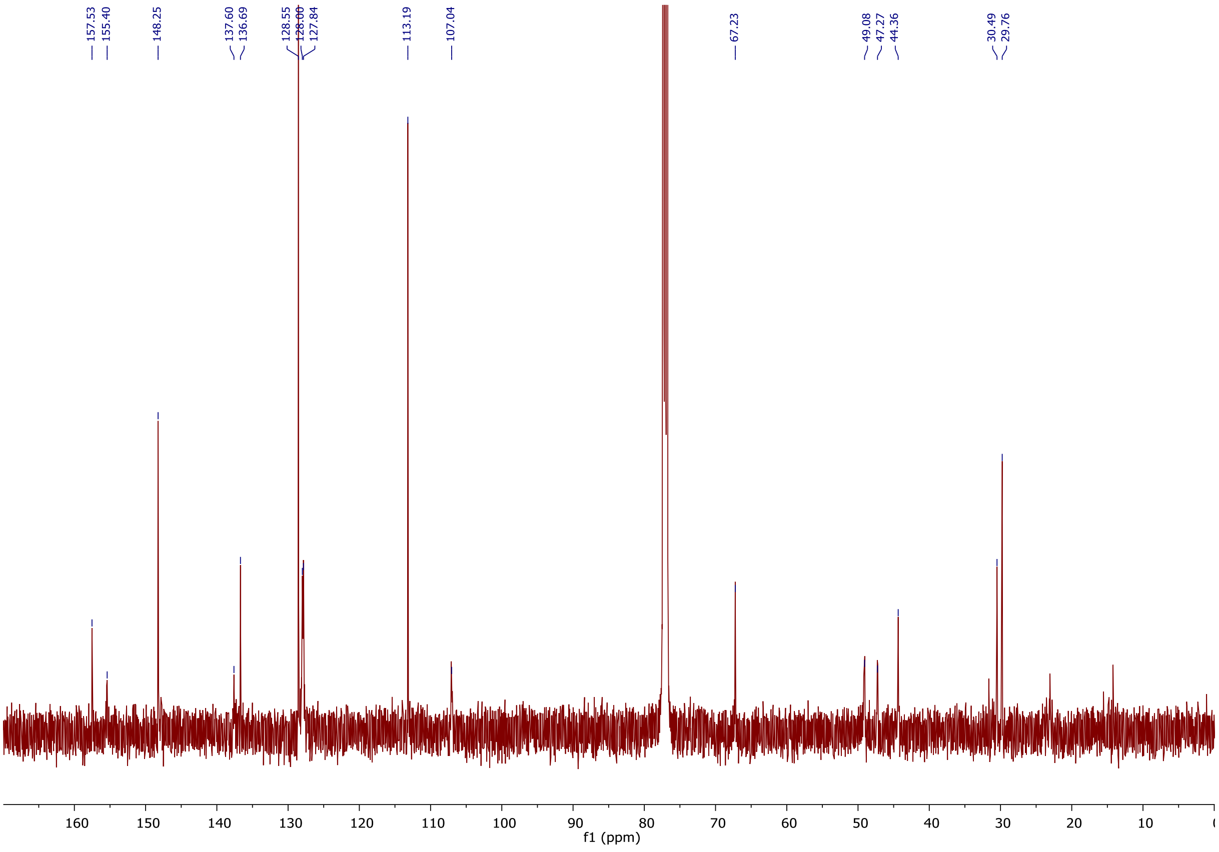

Benzyl 3-(1*H*-pyrazol-1-yl)pyrrolidine-1-carboxylate (**13a**)

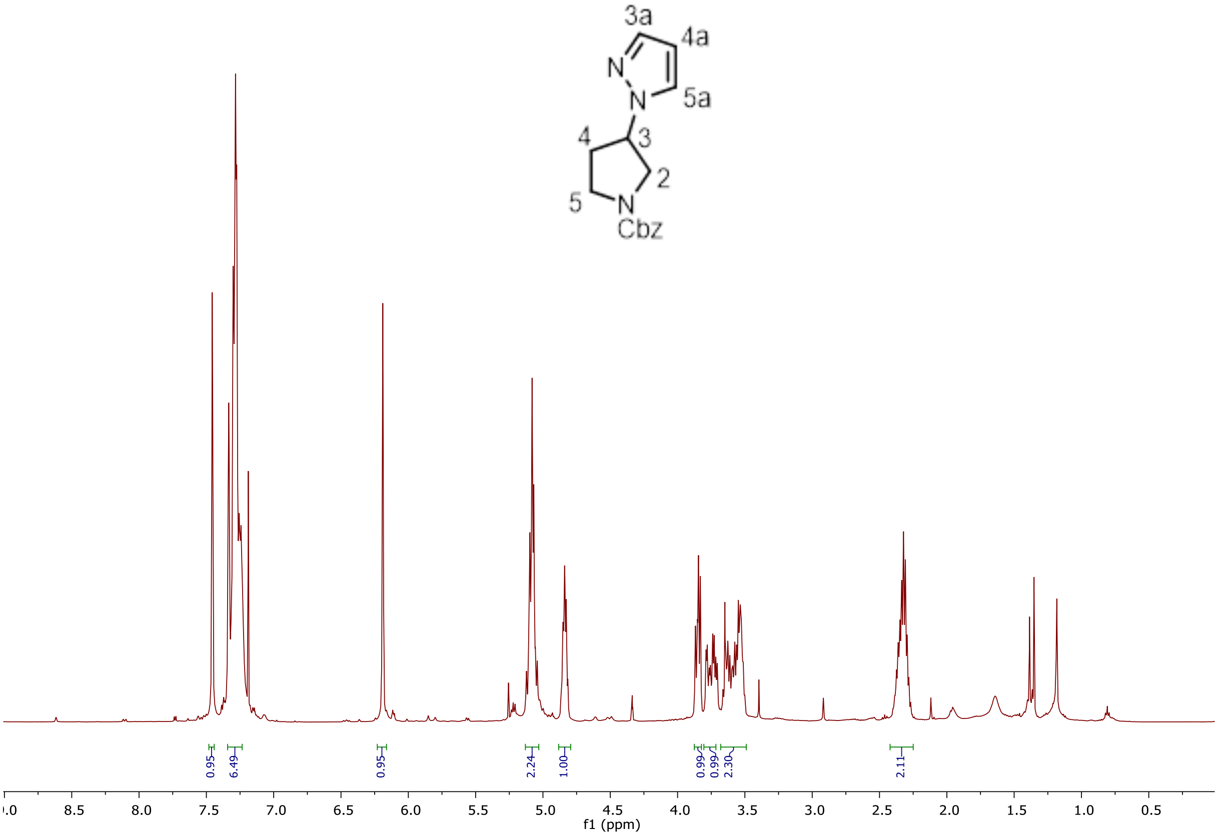

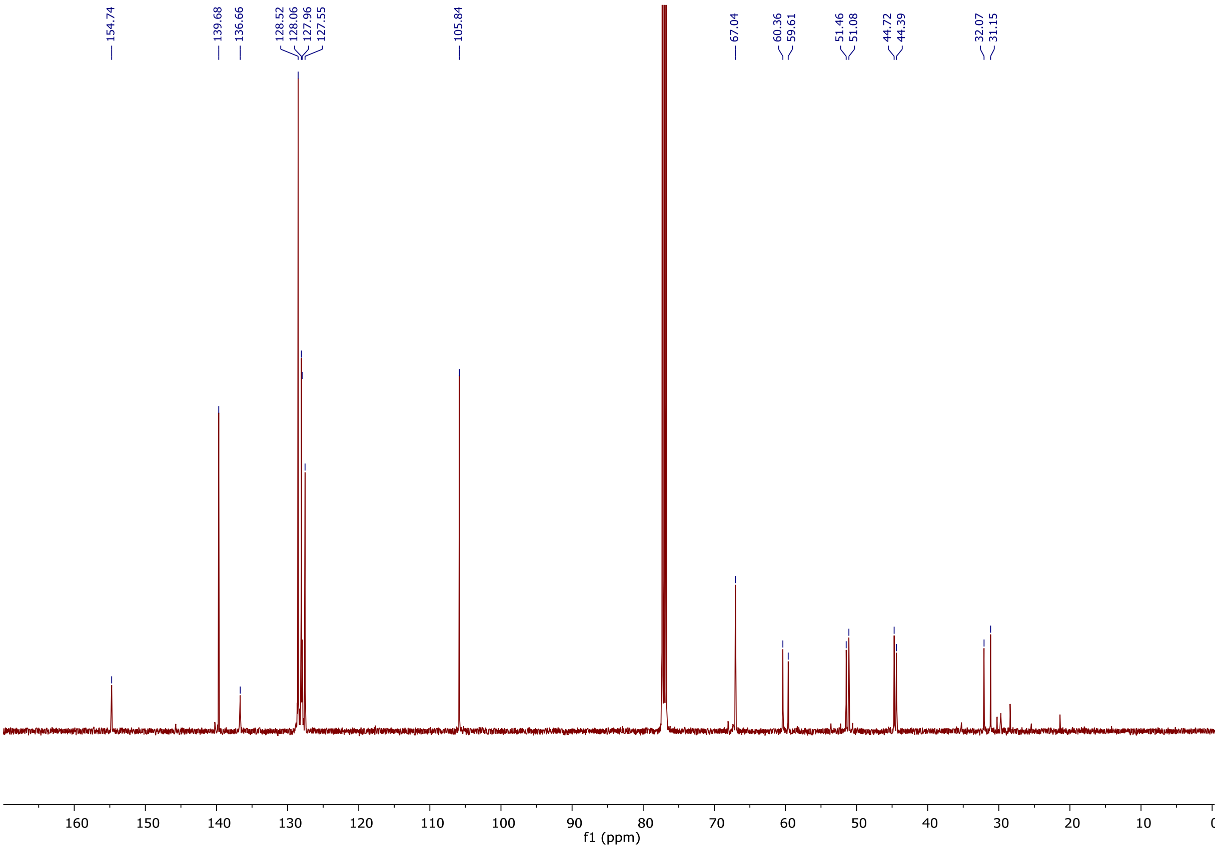

Benzyl 3-(1*H*-imidazol-1-yl)pyrrolidine-1-carboxylate (**13b**)

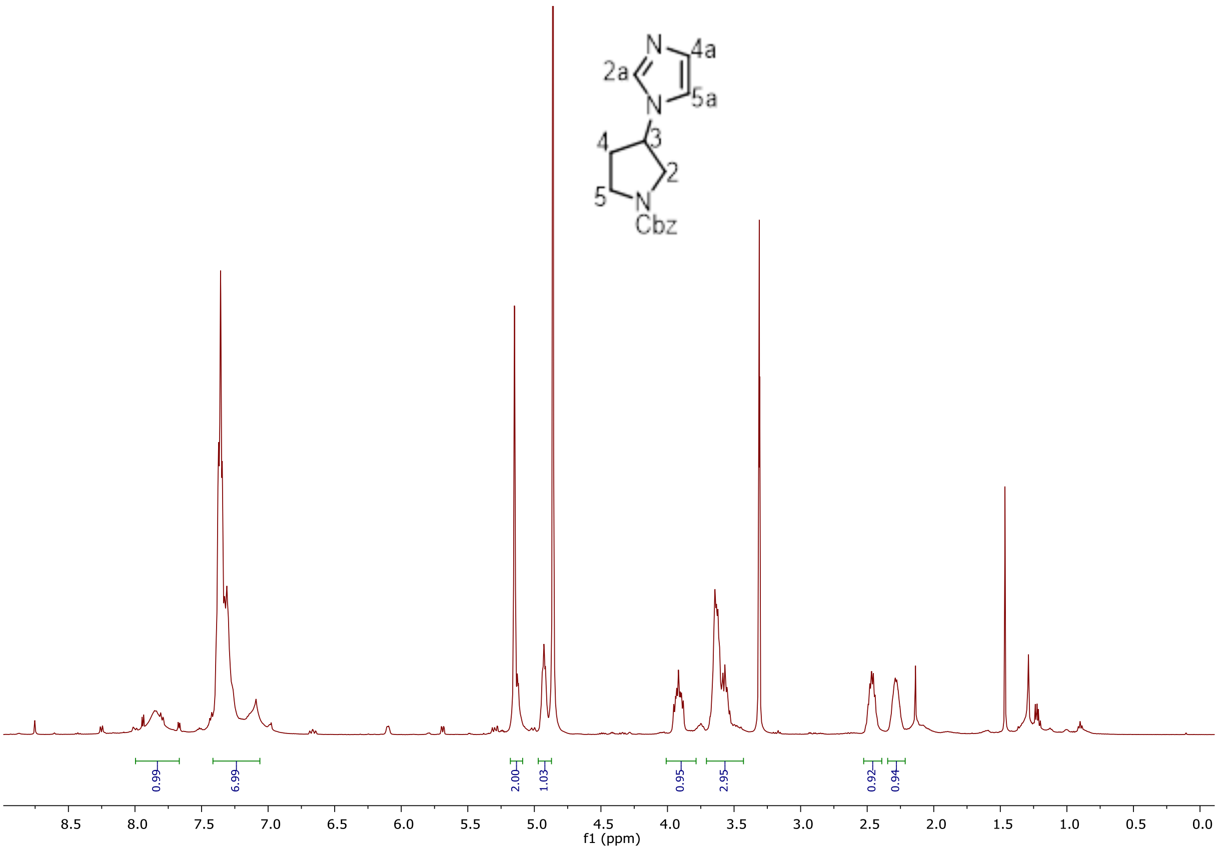

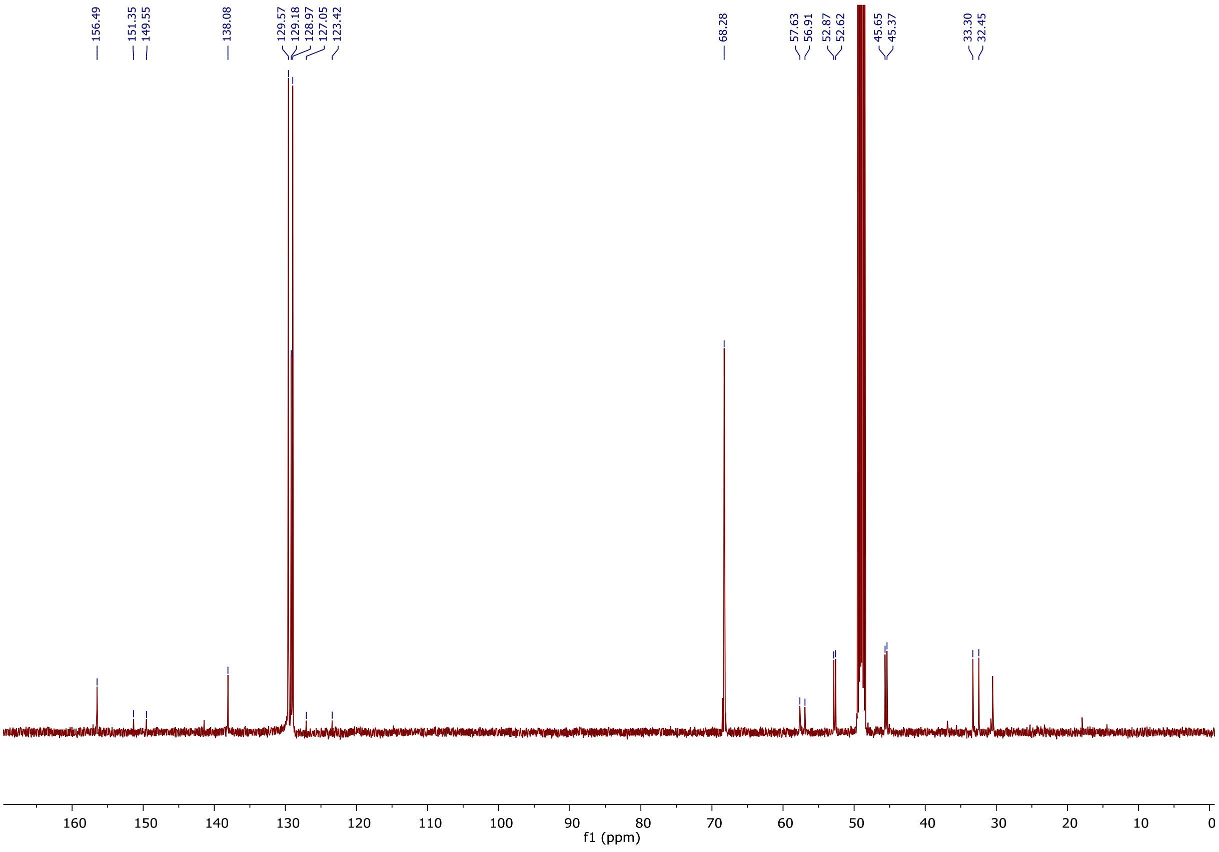

Benzyl 3-(1*H*-1,2,4-triazol-1-yl)pyrrolidine-1-carboxylate (**13c**)

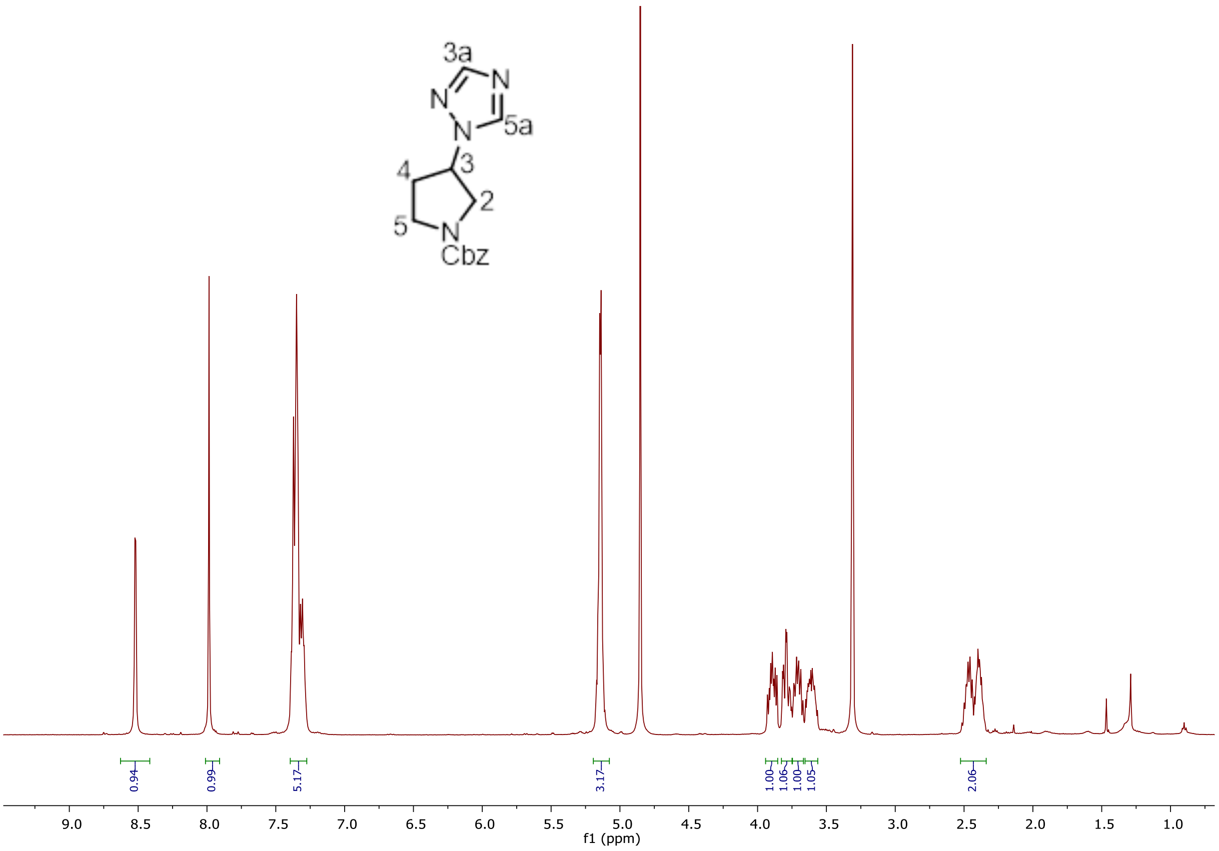

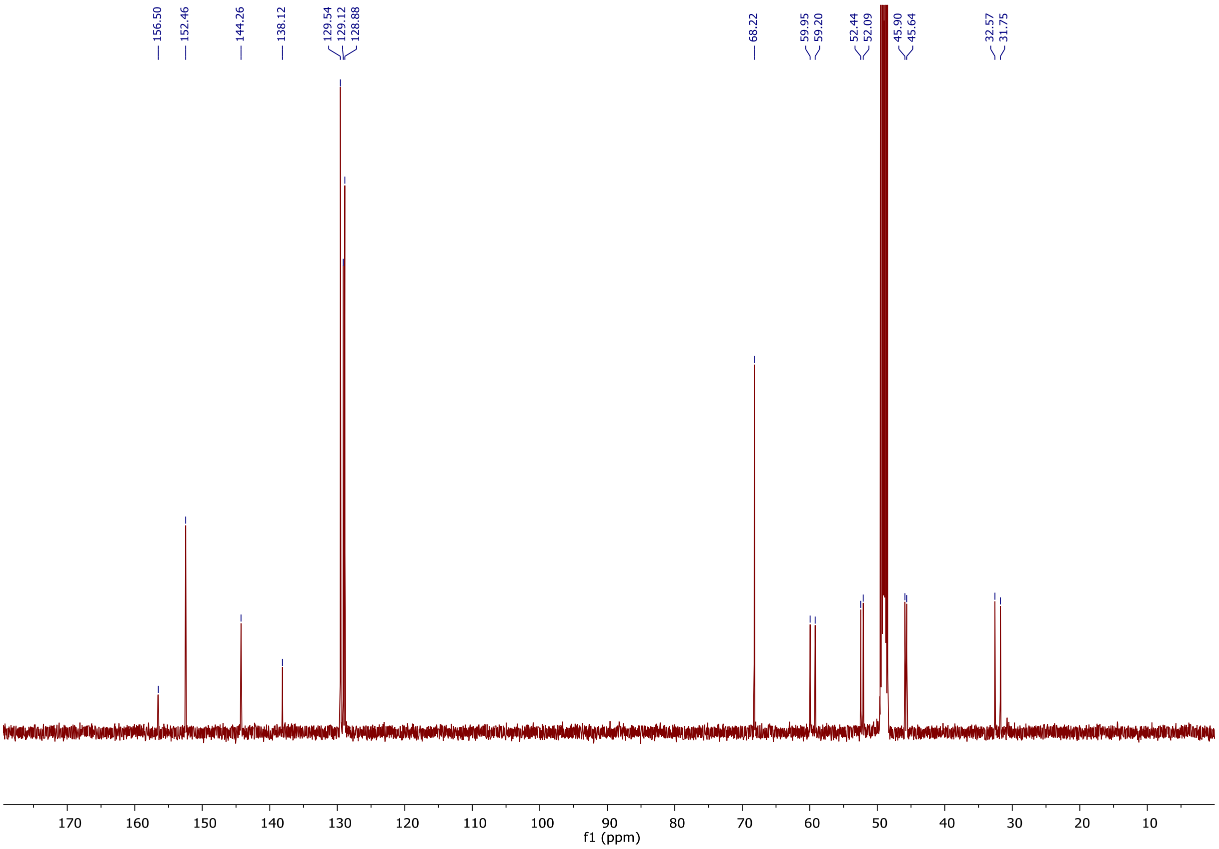

Benzyl 3-(1*H*-pyrazol-1-yl)piperidine-1-carboxylate (**13d**)

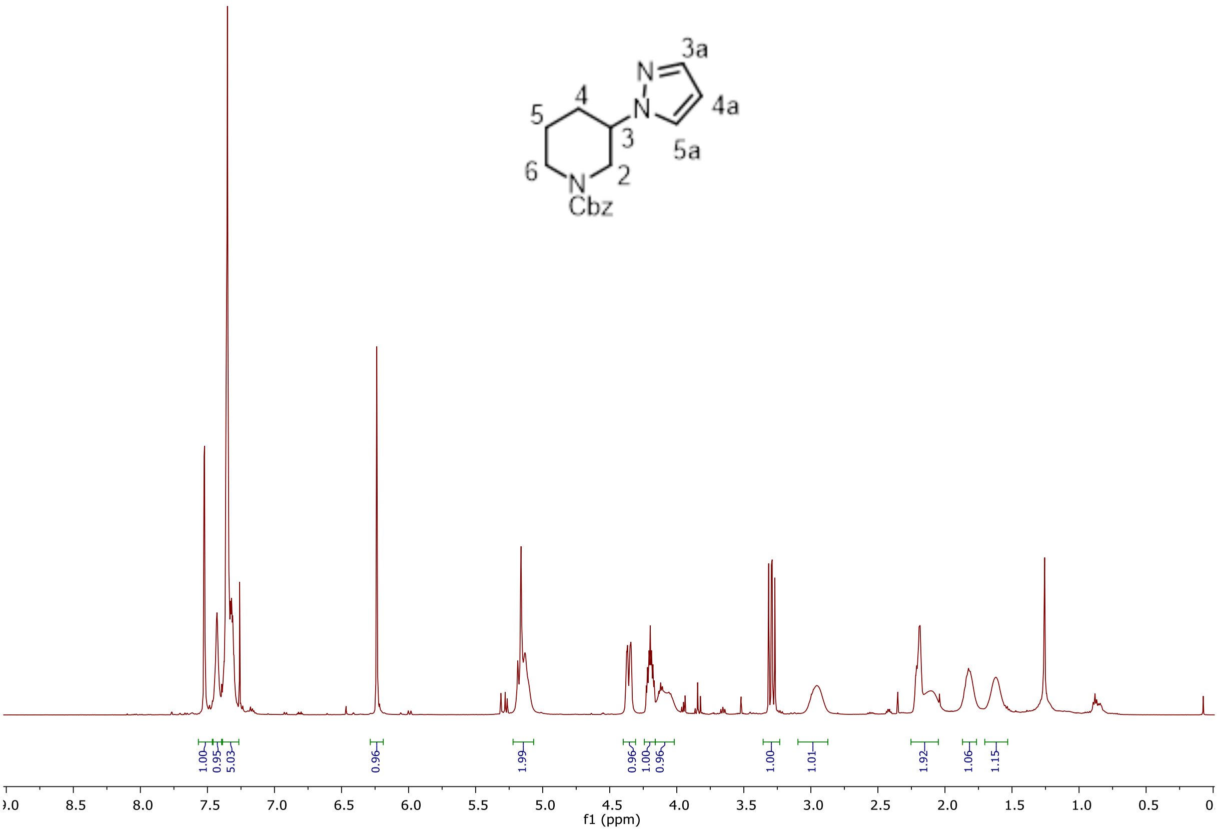

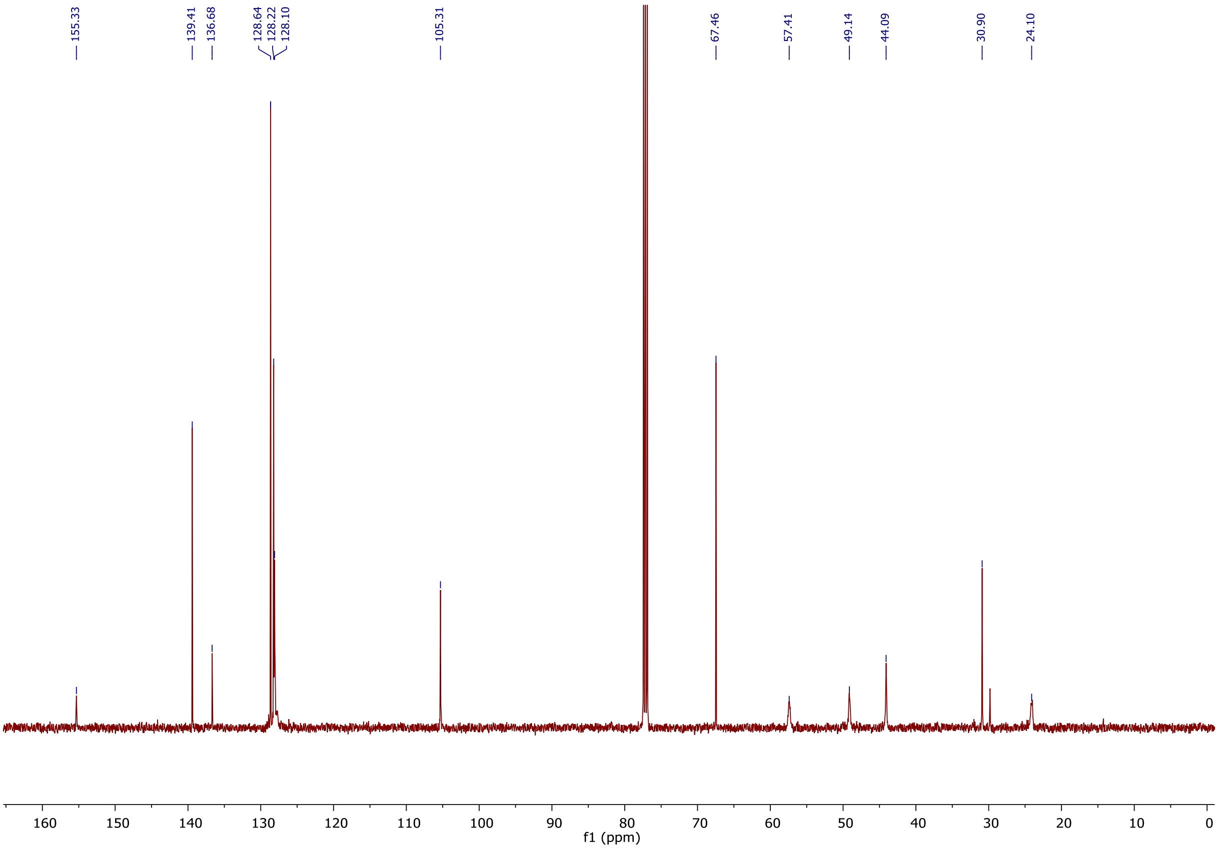

Benzyl 3-(1*H*-indazol-1-yl)pyrrolidine-1-carboxylate (**13e**)

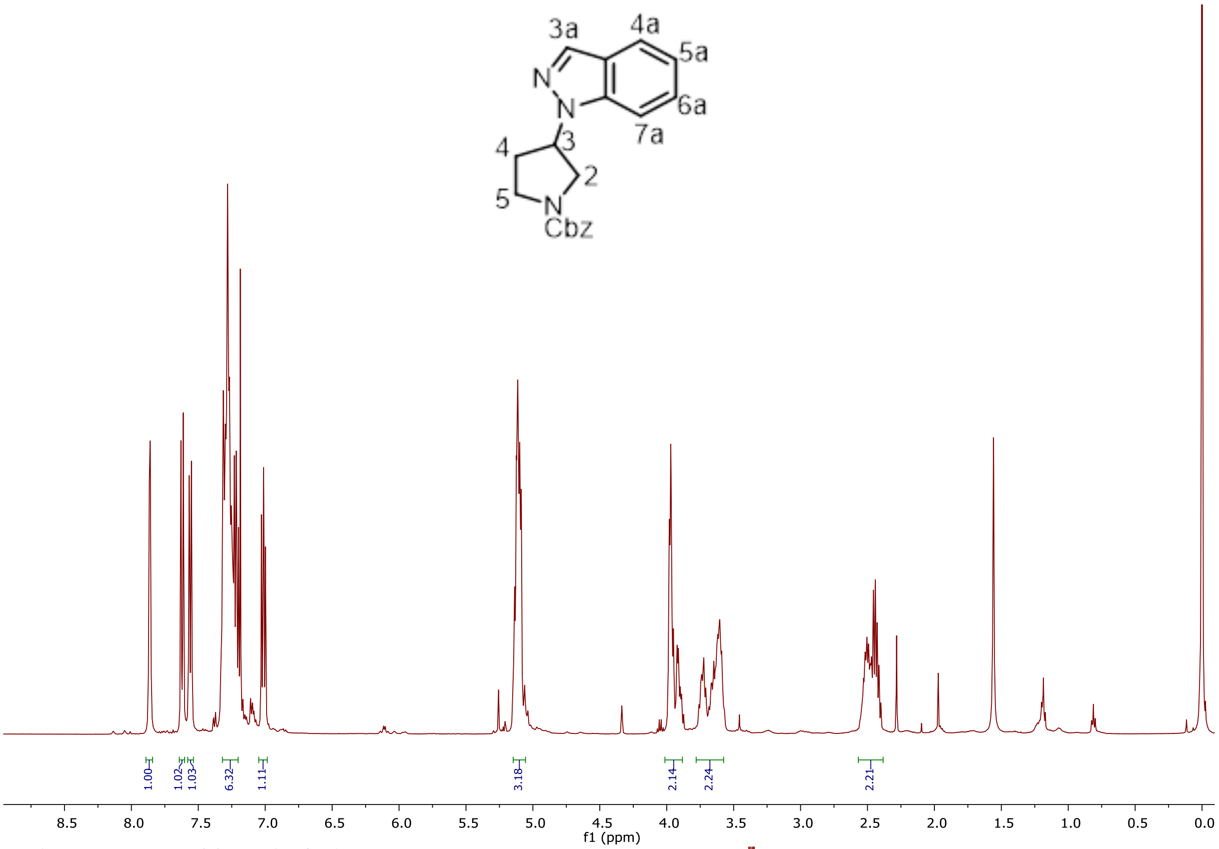

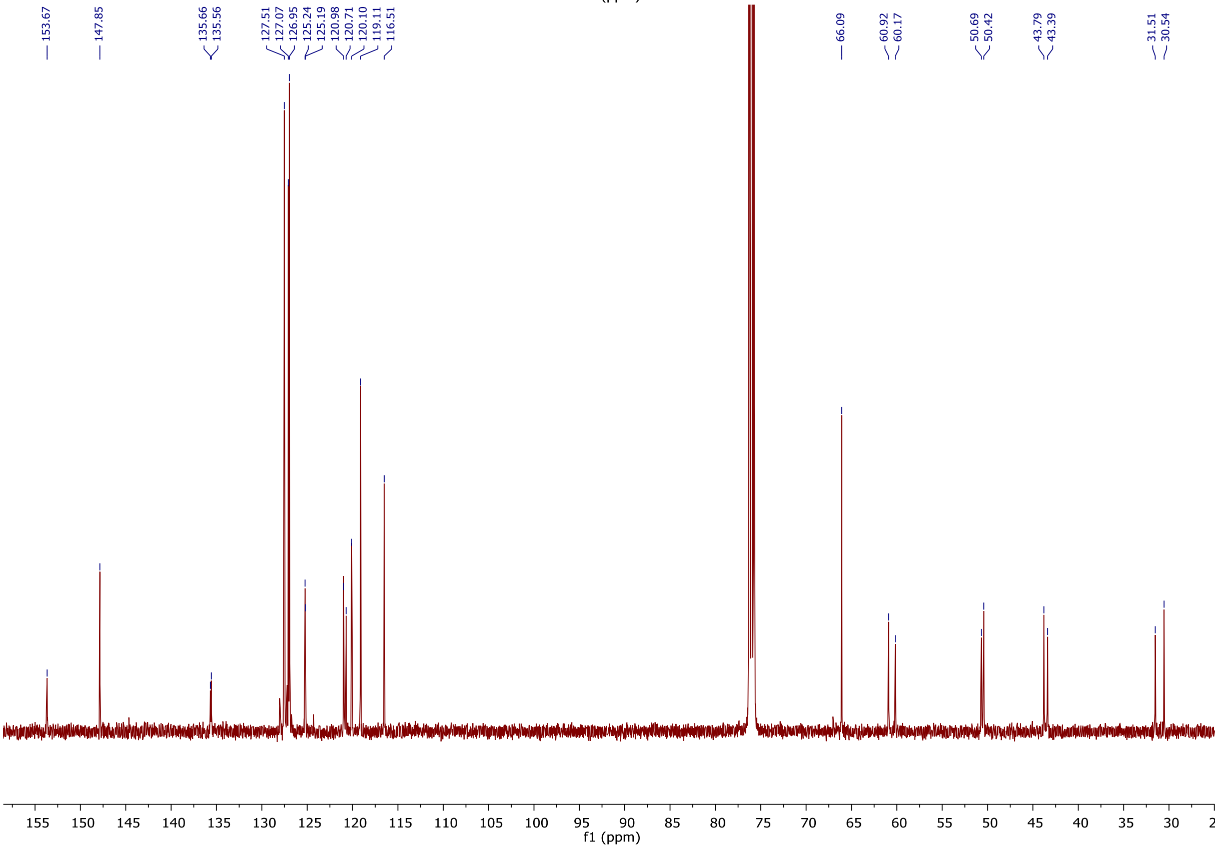

Supplement: Supplementary file 1 — Supplementary [file CHEM-26-14861-s001.pdf]
